# Deep learning and predictive modelling for generating normalised muscle function parameters from signal images of mandibular electromyography

**DOI:** 10.1007/s11517-024-03047-6

**Published:** 2024-02-20

**Authors:** Taseef Hasan Farook, Tashreque Mohammed Haq, Lameesa Ramees, James Dudley

**Affiliations:** Adelaide Dental School, The University of Adelaide, Adelaide, SA 5000 Australia

**Keywords:** Mastication, Range of motion, Variational autoencoder, Signal processing, Clustering

## Abstract

Challenges arise in accessing archived signal outputs due to proprietary software limitations. There is a notable lack of exploration in open-source mandibular EMG signal conversion for continuous access and analysis, hindering tasks such as pattern recognition and predictive modelling for temporomandibular joint complex function. To Develop a workflow to extract normalised signal parameters from images of mandibular muscle EMG and identify optimal clustering methods for quantifying signal intensity and activity durations. A workflow utilising OpenCV, variational encoders and Neurokit2 generated and augmented 866 unique EMG signals from jaw movement exercises. k-means, GMM and DBSCAN were employed for normalisation and cluster-centric signal processing. The workflow was validated with data collected from 66 participants, measuring temporalis, masseter and digastric muscles. DBSCAN (0.35 to 0.54) and GMM (0.09 to 0.24) exhibited lower silhouette scores for mouth opening, anterior protrusion and lateral excursions, while K-means performed best (0.10 to 0.11) for temporalis and masseter muscles during chewing activities. The current study successfully developed a deep learning workflow capable of extracting normalised signal data from EMG images and generating quantifiable parameters for muscle activity duration and general functional intensity.

**Graphical Abstract:**

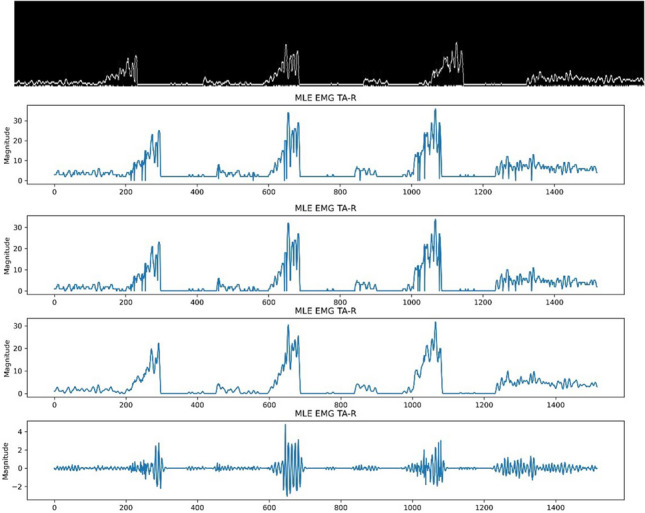

**Supplementary Information:**

The online version contains supplementary material available at 10.1007/s11517-024-03047-6.

## Introduction

Medical signal analyses, spanning routine electrocardiograms (ECG), electromyography (EMG), to case-specific neural electroencephalogram (EEG) and sleep cycle analyses, play an important role in diagnostic healthcare. EMGs are electromagnetic signals generated from muscle contraction, particularly for muscle intensity and activity duration.

Masticatory muscles are specialised groups facilitating mandibular movement for speech, chewing and facial expressions [1]. These versatile muscles adapt to varying masticatory forces throughout life, influenced by changing dental status. Irregular occlusion, tooth extraction and parafunctional habits like bruxism can affect masticatory forces, leading to issues like temporomandibular joint dysfunction and myofascial pain dysfunction syndrome [2, 3].

### Standardisation and image archival

Specialist practices and hospitals observe mandibular muscle activity and temporomandibular joint function, generating extensive signals over extended treatment periods. This results in a substantial collection of longitudinal data per patient. These signals, obtained during various jaw movements like mouth opening, lateral excursion, protrusion and chewing, are typically relative values [2]. Although relative and subjectively interpreted, two pivotal features in diagnosing musculoskeletal dysfunction of the head-neck region involve overall muscle activity durations and the average intensity of muscle response following activation [4]. EMG images created for reporting integrate signals set at a specific magnification to emphasise clinically significant features. This standardisation process precedes image storage to ensure consistency across a broad sample population, aiding in referrals and facilitating future research endeavours. In Australia, medical data, including biomedical signal images, are archived for at least seven years post-treatment for an adult according to Privacy laws. Archival is the conversion of medical data into low storage-consuming, easy-to-view files that can be preserved over cloud storage for long-term accessibility of crucial patient-centric information in healthcare research [5]. Medical data is archived upon treatment completion for legal compliance requirements, training, research, education and to serve as reference for future diagnoses [6, 7].

### The current state of research on image-to-signal analysis

Data normalisation maintains consistency in medical signal datasets, such as electrocardiogram (ECG) outputs, across different clinics and devices, minimising redundancy and data dependency, thus preserving data integrity [8]. Recent exploration in this domain focuses on applying data normalisation to regions of interest with EMGs, demonstrating the shear modulus of large muscles in extremities [9]. Addressing baseline wander, a common issue in EMG data acquisition, is often achieved through baseline correction during data acquisition, as it is less complex than corrections made in post-processing [10].

Modern post-processing techniques that apply deep learning, like generative adversarial networks (GAN), have successfully managed noise and baseline corrections. This methodology has been demonstrated on electroencephalogram (EEG) signals and variational encoders [11]. Various mathematical models, including wavelet transform, time–frequency approaches, Fourier transform, Wigner-Ville Distribution (WVD), statistical measures and higher-order statistics, are employed for signal analysis. AI approaches for signal recognition involve artificial neural networks (ANN), dynamic recurrent neural networks (DRNN) and fuzzy logic systems for mapping EMG inputs to desired hand actions [12].

Before visualisation, it is important to correct baseline drifts across the signal. The application of a thresholding algorithm using the moving average method has demonstrated applicability for various signals, such as magnetocardiography (MCG) [13]. Open-source signal correction toolkits like Neurokit2 have been widely acclaimed and proven effective for EMG, electrocardiography (ECG) and EEG data [14]. In models trained by limited medical imaging datasets, data augmentation is the practiced norm [15]. To generate synthetic signals, GAN-based synthesisers and Variational Autoencoder synthesisers have been documented as effective approaches [16, 17].

High-dimensional data, defined by significantly more features or dimensions than samples or observations, poses specific challenges. Recent research demonstrated the effectiveness of feature extraction libraries such as Time Series Feature Extraction Library (TSFEL) in extracting temporal and spatial data for multidimensional signal feature extraction [18]. The Gaussian mixture model (GMM) with principal component analysis (PCA) has found successful implementation in clustering and diagnosing knee osteoarthritis from acoustic emission signals [19]. Additionally, the K-nearest neighbour method remains a popular choice for clustering and classification in EMG data [20]. DBSCAN, when coupled with Lidar technology, is effective in handling variable point density, identifying clusters of arbitrary shapes in three-dimensional point clouds [21]. This algorithm excels at noise removal, distinguishing meaningful structures from outliers, and is adaptable to variable density environments. Its efficient memory usage makes it well-suited for processing large Lidar datasets in applications like autonomous vehicles, biomedical signals and geospatial analysis [21].

### Rationale

In the current research landscape, several gaps have been identified:

1. Despite the widespread application of data normalisation and synthetic signal processing in various medical signal-based datasets, the normalisation of EMG signals from masticatory muscles for broad applicability and correlation with similar image datasets from different facilities, devices and varying timeframes remains unexplored [22].
2. The development of a image-to-signal conversion system is necessary for mandibular EMG, but it must ensure that the converted signal features are classifiable through clustering. This classification is crucial for tasks such as pattern recognition, exploratory analysis, anomaly detection and predictive modelling of temporomandibular joint complex function [23]. Nevertheless, there is currently a lack of a documented method to identify suitable clustering methods for mandibular EMG images.
3. Although digital images of biomedical signals are commonly stored in hospital and dental practices, accessing archived signal outputs years later can be challenging due to proprietary software limitations [24]. Continuous access to proprietary software may be limited for various practitioners and researchers [25]. Although there have been advancements in image-to-signal conversion for cardiac and neural signals, the exploration of open-source muscle EMG signal conversion is notably limited.

### Research objectives

To address the research gaps stated in the rationale above, the following objectives were formulated:

1. To develop an opensource deep learning workflow to extract normalised signal data from EMG images of mandibular elevator and depressor muscle activity during mouth opening, lateral excursion, anterior protrusion and chewing activities.
2. To identify the most appropriate methods and parameters for clustering the extracted data to generate cluster-centric signals and normalised quotients for signal intensity and muscle activity durations.

## Materials and methods

The study adhered to the minimum information for the clinical artificial intelligence modelling (MI-CLAIM) checklist [26]. The codes used to generate models in the current study adhered to the PEP-8 guidelines. Data was obtained from 66 participants in South Australia using BioEMG III (Bioresearch Inc, USA) between June and August 2023. All data were anonymised, ensuring no identifiable information was utilised. The collected data included EMG signals of the temporalis, masseter and digastric muscles, recorded during maximum mouth opening, lateral jaw excursion, anterior protrusion and chewing. The study, approved by the University of Adelaide Human Research Ethics Committee (HREC-2022–185), involved the following steps described in their respective subsections:

1. Isolating ROI from archived images
2. Extracting signals from ROI
3. Baseline correction
4. Further correction with moving average
5. Signal pre-processing
6. Feature extraction and data augmentation
7. Visualising high-dimensional data
8. Normalisation through clustering
9. Generating cluster-centric data
10. Determining signal intensity and duration through normalised quotients

### Isolating regions of interest from archived images

**Fig. 1:**
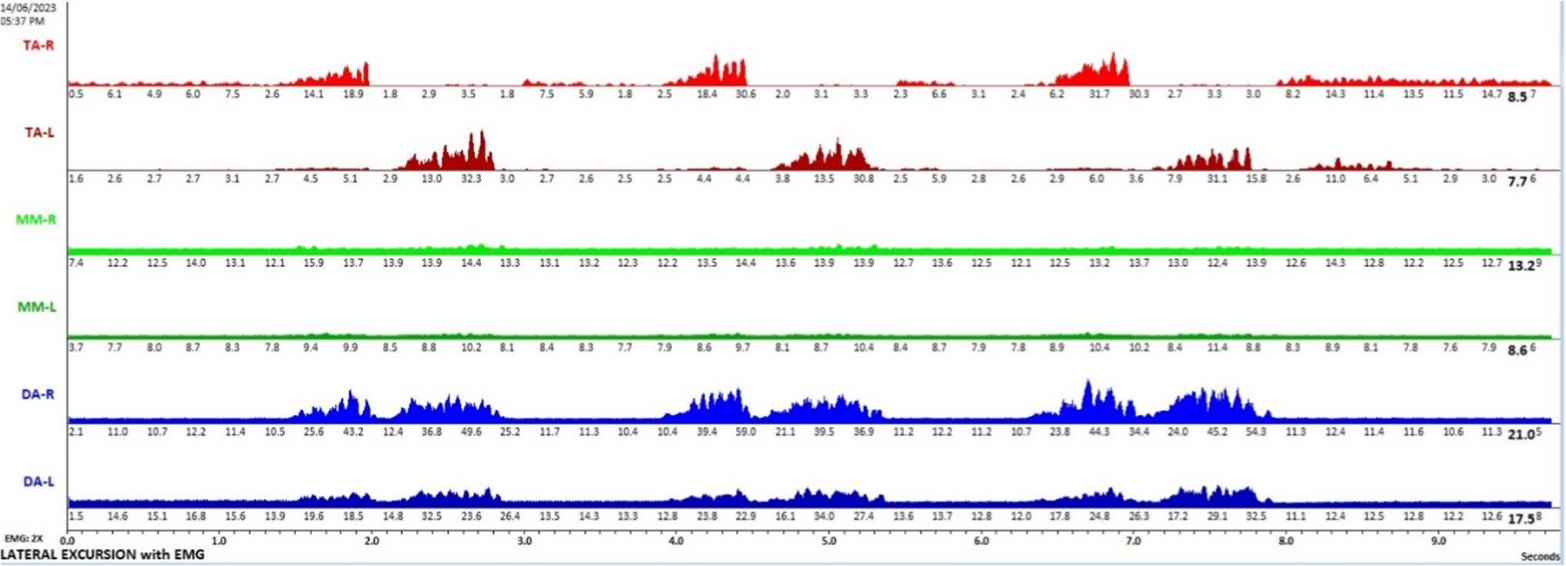
A sample of the archived image of a signal sweep for temporalis, masseter and digastric muscles when performing lateral excursion

Each subject’s images, representing specific EMG activities, was stored as JPEG files. The EMG activities included maximum mouth opening (MMO), maximum lateral excursion (MLE), maximum anterior protrusion (MAP) and chewing. The signals for each of the six muscles involved in the activities were retrieved in the form of a JPEG image. Figure 1 illustrates a sample of the original image file stored in the database.

Initially, the ‘Canny edge detection’ function from OpenCV was employed to convert each image into black and white, ensuring that the background was black and the signal edges were white. The parameters for Canny edge detection included a minimum threshold of 0.66 times the average pixel value and a maximum threshold of 1.33 times the average pixel value. To isolate the six regions of interest (the six muscle signals), pixel boundaries were manually established based on visual inspection to determine a single, universally applicable boundary rule for all signals and muscle activities. Despite variable sizes, the images were made consistent by truncating the minimum number of horizontal pixels (labelled ‘m_ph’) and vertical pixels (labelled ‘m_pv’) across all images, resulting in a resolution of m_pv x m_ph. The images showcased varying ranges in pixel counts, ranging between 1604 × 579 and 1617 × 590 pixels.

**Fig. 2:**
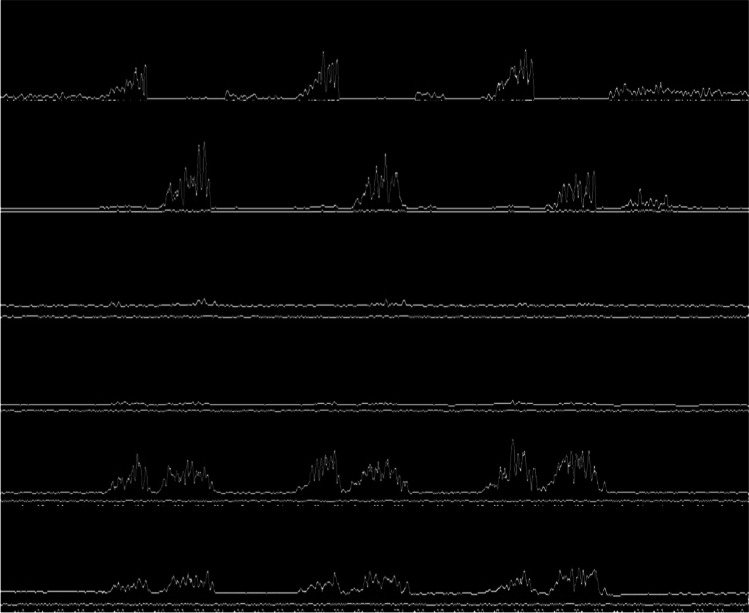
Signal edge detection for temporalis, masseters, and digastrics when performing lateral excursion

The following rules were then applied to Isolate the six regions of Interest (ROI) In each Image: left pixels truncated = 85 (ignoring the first 85 columns), top offset = 20 (ignoring the first 20 rows for every image), and each of the six regions was given a height of (89 – top offset). These parameters emerged through a process of trial and error, undertaken with the aim of achieving uniformity across images of diverse dimensions. Figure 2 illustrates the signal images converted from the original archived images following the mentioned method.

### Extracting the signals from the regions of interest

**Fig. 3:**
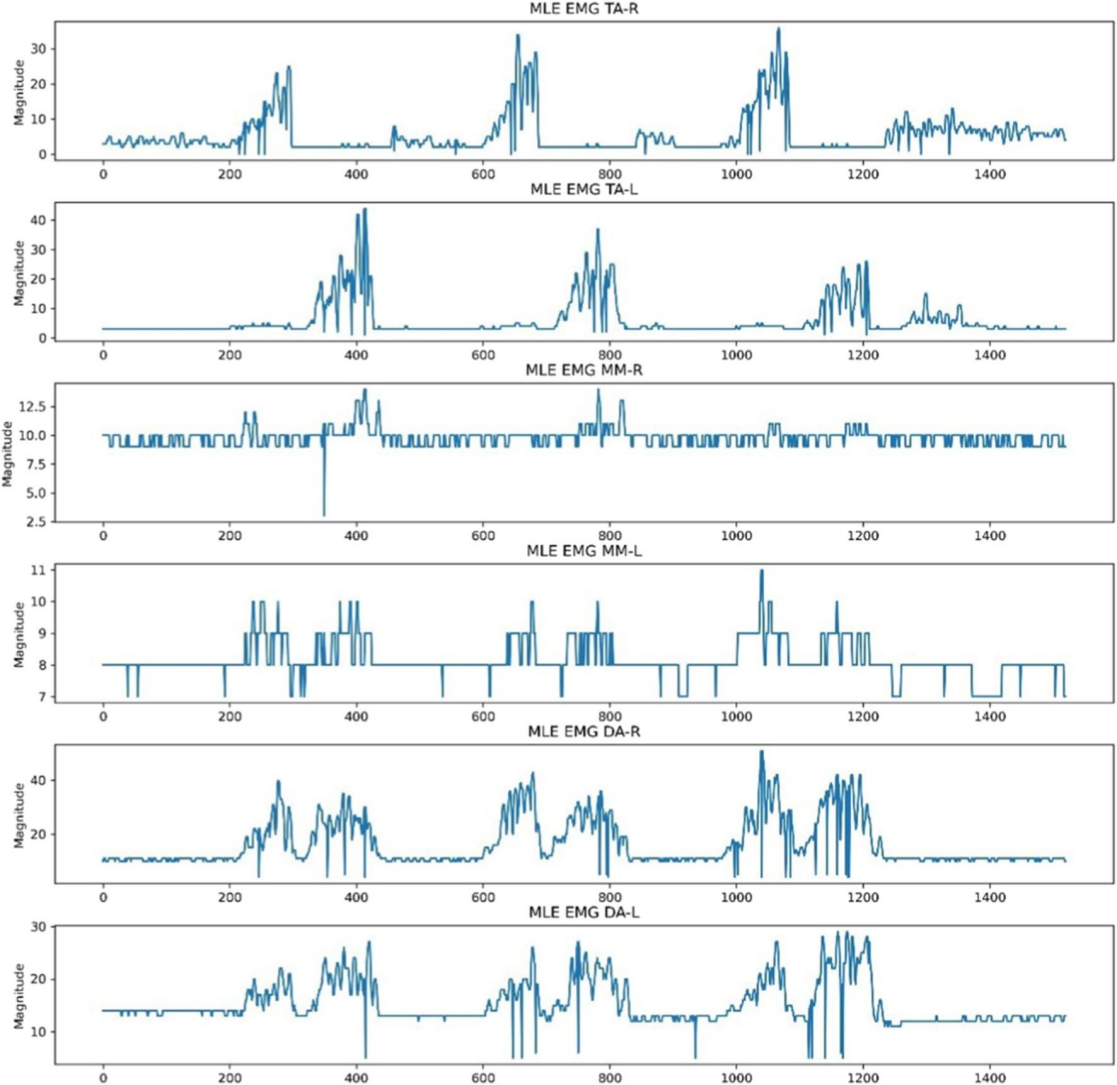
Signal extraction from detected edges

Pixel values were utilised to extract the actual signal points from each segmented image of EMG signal. Given that the images were black and white, with signal edges in white and the background in black, the extraction process involved iterating through each column. For every row index, the white pixel values were extracted. If there were one or more white values per pixel, the pixel row difference (number of rows – last encountered white row index) was considered as the signal point. If no white row values were detected in the column, the signal point was considered as 0. Figure 3 demonstrates an example of signal extraction in the current workflow. Notice that the baselines are not normalised in this step and would require additional correction.

### Baseline correction

**Fig. 4:**
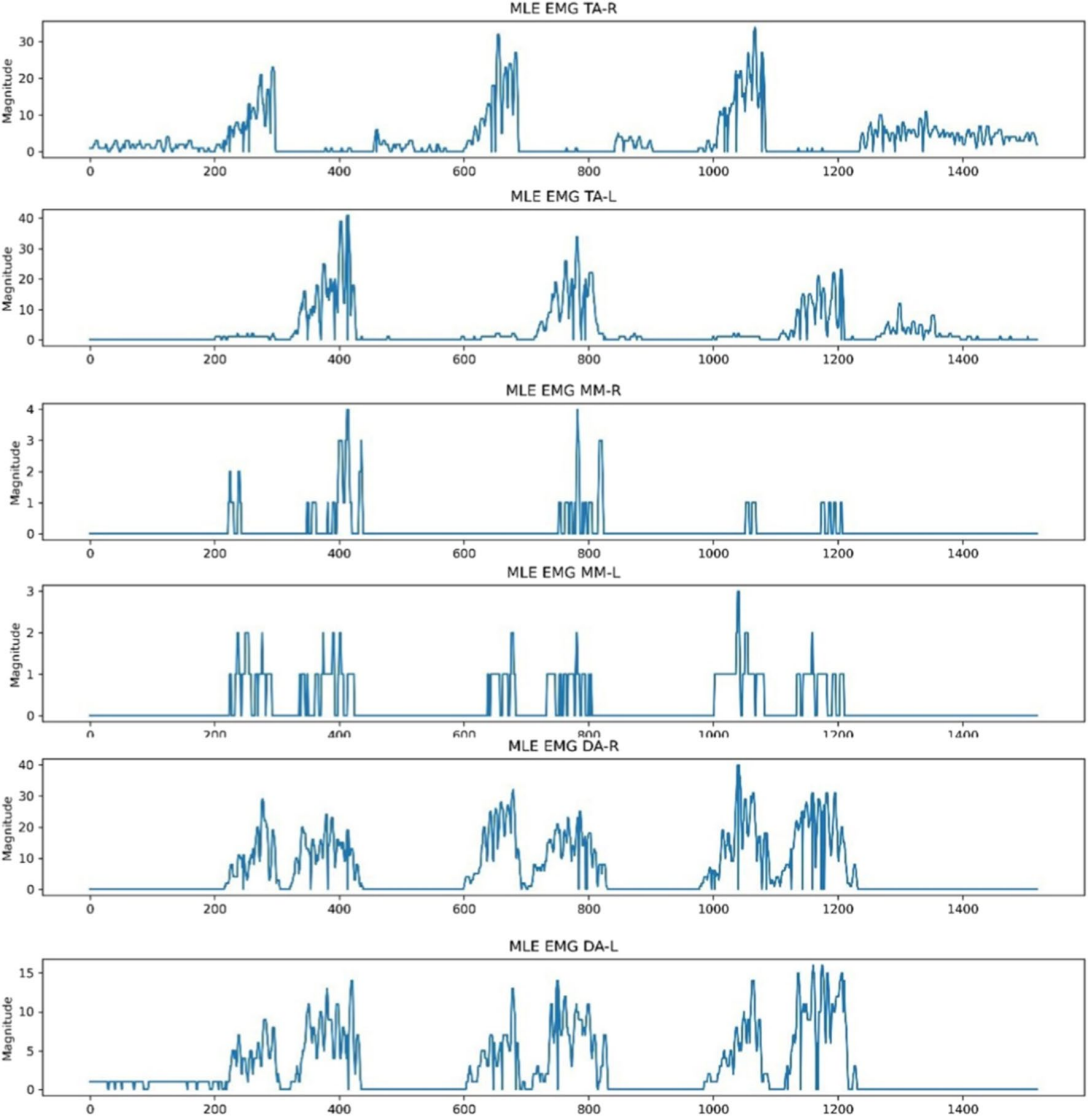
Baseline correction of extracted signals

Each extracted signal underwent baseline correction, which involved subtracting the most frequently occurring signal point across all pixel rows. Subsequently, any resulting signal points that produced negative values were replaced with 0 and the graph was redrawn. Figure 4 displays the six extracted signals after baseline correction.

### Further correction with moving average

**Fig. 5:**
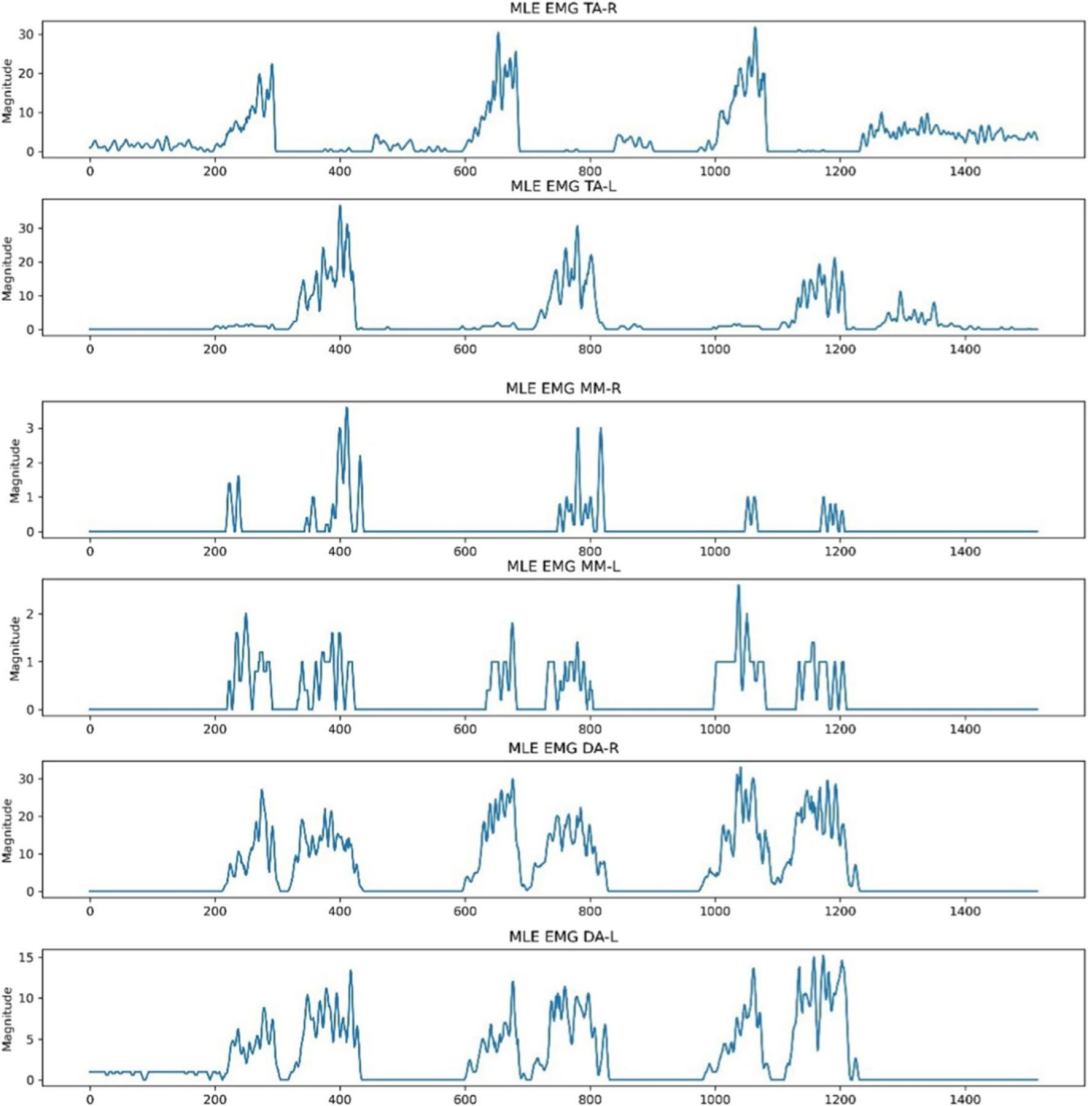
‘Moving average’ function applied to the extracted signals following baseline correction

The baseline, previously adjusted to a magnitude of 0, still exhibited sharp and abrupt drops due to the absence of white pixel values, indicating a signal point. This was a result of edges not being detected in certain columns by OpenCV, attributed to signal point clipping from the thresholds used to isolate the six regions of interest. To address this, the ‘moving average’ function was implemented on each extracted signal using a window size of 5. This was done by performing convolution between the signal and a sequence of five points, each with a value of 1/5. Padding was executed to ensure that the resulting signal had a length of max(M, 5) – min(M, 5) + 1, with M being the number of points in the signal. Values outside the signal boundary did not influence the moving average output. Figure 5 illustrates the six signals after the application of the moving average.

### Signal pre-processing

**Fig. 6:**
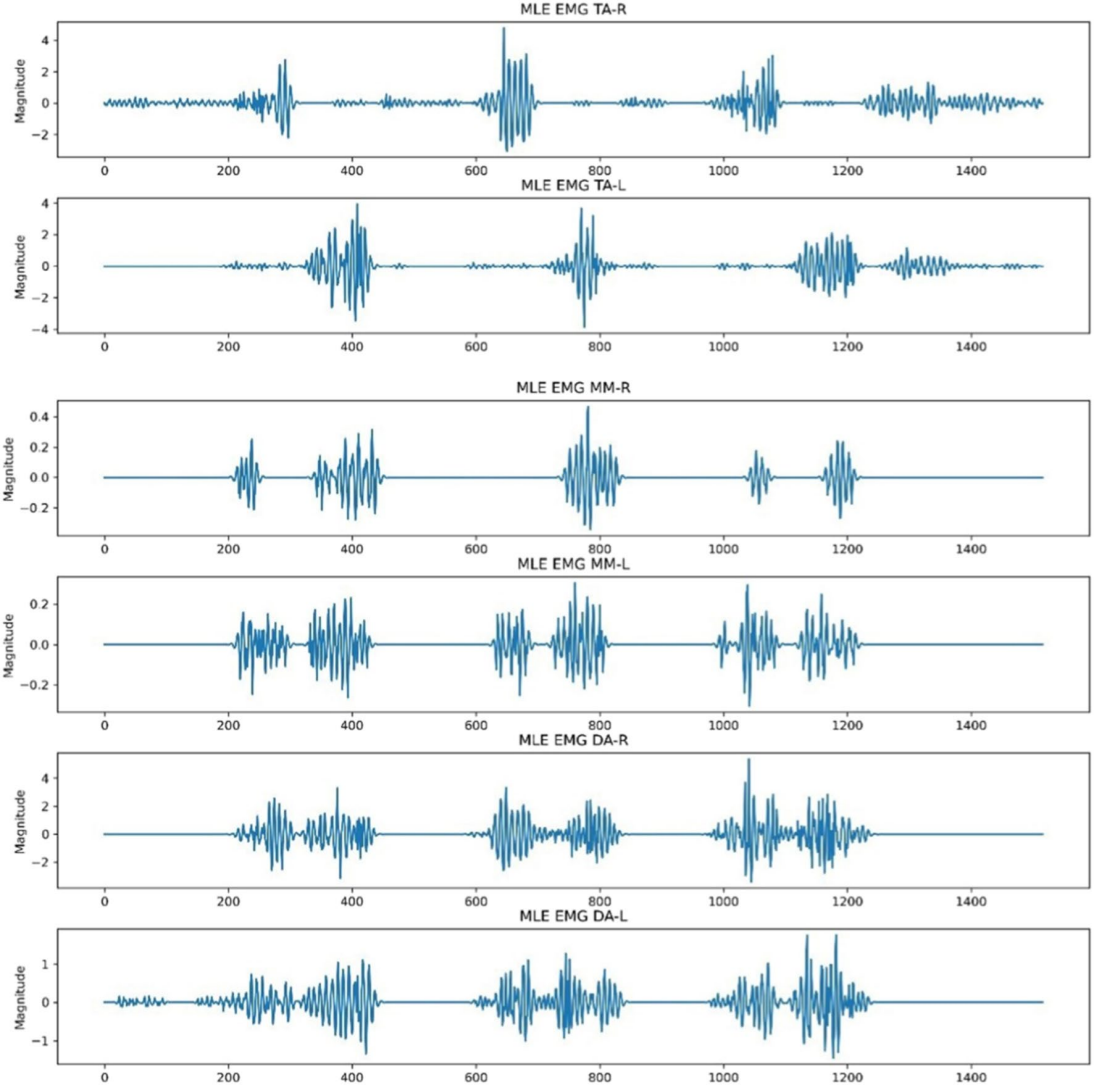
Signal conversion following moving average using the Neurokit2 signal pre-processing tool

The NeuroKit2 Python toolbox was then employed for signal pre-processing [14]. NeuroKit2 is an open-source package dedicated to neurophysiological signal processing, encompassing various body signals like EMG, electrocardiography and more [14]. Presented below in Fig. 6 are the same six signals after undergoing pre-processing for MLE. This identical procedure was applied to other activities like MMO, MAP and Chewing EMG signal images for each subject. The resultant signals were then exported as CSV files, ensuring six distinct CSVs for each subject, each representing the EMG of a specific muscle.

### Feature extraction and data augmentation

Temporal (time domain based) and spectral (frequency domain based) features were extracted for each signal using the python library Time Series Feature Extraction Library (TSFEL), which facilitates fast exploratory data analysis and feature extraction from multidimensional time series signals [18]. Each signal was normalised to a range of 0–1, and TSFEL’s feature extraction was applied, resulting in 382 features for every signal. Any missing values were imputed with the median, and features with zero variance were discarded, leading to 369–372 features based on the muscle type.

Due to the limited size of the dataset, the utilisation of data augmentation was deemed necessary. Synthetic data based on the extracted features was generated using the python library Synthetic Data Vault (SDV), which includes various synthetic data generation tools, including classical statistical techniques and deep learning approaches [27]. Two different deep learning models, the CTGAN = Conditional Generative Adversarial Network (CTGAN) synthesiser and Triplet-Based Variational Autoencoder (TVAE) synthesiser were considered for synthetic data generation.

For each type of EMG activity, hyperparameter tuning was performed for both CTGAN and TVAE. For CTGAN, the tuned parameters included embedding dimensions (256, 512), generator dimensions (256, 512), and discriminator dimensions (128, 256). For TVAE, the tuned parameters were embedding dimensions (128, 256), compress dimensions (128, 256) and decompress dimensions (128, 256). Each model was trained for 500 epochs on the extracted features, generating 200 synthetic observations per training. The average of two performance scores, column shapes and column pair trends were saved. TVAE consistently produced higher quality synthetic data across all muscle types and was therefore used moving forward.

For each muscle, the best-performing model was run on the original extracted features to generate 1000 new synthetic observations. A dataset for each muscle, consisting of synthetic and original observations was exported as CSV files. This process was repeated for all muscle activities.

### Visualising high dimensional data

For each EMG activity and muscle, a subset of 800 synthetic observations was combined with the original observations, creating a dataset of 866 observations. To address the ‘curse of dimensionality’ prior to data visualisation, principal component analysis (PCA), a widely used dimensionality reduction technique, reduced the number of features from 368–372 to 50, and the data was standardised [28]. To visualise this data, t-SNE, a dimensionality reduction technique for visualising high-dimensional data in 2 or 3 dimensions to reveal potential clusters, was subsequently used [29]. The key parameter in t-SNE is perplexity that determines how points are grouped. A lower perplexity emphasises local relationships, while a higher perplexity can reveal global relationships. After repeated visual evaluation, a perplexity of 30 provided a reasonable visualisation that captured both global and local relationships.

### Normalisation through clustering

The evaluation of clustering techniques involved applying three distinct methods: K-Means clustering, GMM clustering and DBSCAN clustering. To determine the optimal number of clusters and assess clustering performance, multiple metrics were used. The elbow method entailed testing various cluster numbers (k), examining the sum of squared distances between each point and its cluster centre and identifying an ‘elbow’ point on the graph, indicating the optimal cluster number. Silhouette scores were employed to measure the quality of clusters based on their cohesion and separation, with higher scores indicating more distinct clusters [30]. The Davies Bouldin Index was utilised to assess clustering quality by considering both compactness within clusters and separation between clusters, using a lower value to signify superior clustering performance.

#### K-means clustering

The analysis began with the K-means clustering algorithm. K-means clustering partitions data into a predefined number of clusters by iteratively assigning points to the nearest cluster centre and updating centroids to minimise the ‘within-cluster’ sum of squares. Elbow plots were generated over a range of 2 to 20 clusters with 1-unit increments. K-means was applied to the initial PCA output for each cluster count and repeated thrice to create three distinct elbow plots. Silhouette scores were subsequently graphed. The Silhouette scores were visualised across a range of 2 to 20 clusters with 1-unit increments. K-means was run on the initial PCA output for each cluster count and replicated three times, producing three Silhouette score plots.

#### GMM clustering

Gaussian mixture models (GMM) is a probabilistic model that assumes the data is generated from a mixture of Gaussian distributions. It assigns data points to multiple Gaussian distributions, allowing for more flexible cluster shapes and capturing uncertainties in cluster assignments through soft clustering or probability distributions for each point. It used two graphical tools for identifying the optimal K value: Silhouette and BIC scores. GMM clustering was applied to the PCA output across a cluster range of 2 to 20 with increments of 1, executed in 3 repetitions to generate separate graphs for Silhouette and BIC (Bayesian Information Criterion) scores.

#### DBSCAN clustering

DBSCAN (Density-Based Spatial Clustering of Applications with Noise) identifies clusters based on the density of data points. It distinguishes core, border and noise points, defining clusters as regions of high density separated by areas of low density, and is effective in discovering clusters of arbitrary shapes while being robust against noise and outliers. DBSCAN has two crucial parameters: Epsilon and MinSamples. Epsilon defines the maximum distance allowed between two points within the same cluster, while MinSamples determines the minimum points required to form a cluster. The parameters were fine-tuned following the methodology outlined in a specific reference [31]. The MinSamples value was set at 2 times the number of dimensions [32]. To establish Epsilon, the average distance between each data point and its ‘n’ nearest neighbours (where n = MinSamples) was calculated, producing a graph of sorted distances versus points. Davies Bouldin Index, a metric to calculate the ratio between the sums of intra- and inter-cluster similarities, was applied.

### Generating cluster-centric signals

The original observations were extracted from the clusters derived by the best-performing algorithm to create two normal EMG signal cohorts. These signals were normalised to a range of − 1 to 1. The process involved setting the first signal as the initial reference, then aligning subsequent signals with this reference using cross-correlation through convolution, calculating the average between each signal and the reference, and updating the reference signal to be the new averaged signal. This iterative averaging procedure aimed to adjust for the varying alignments between the signals, ensuring that no single signal was considered exclusive throughout the process.

### Determining signal intensity and duration through normalised quotients

Quotients were computed using the NeuroKit2 tool discussed prior subsections. The tool generated masks with 1 s indicating the presence of activity. For the intensity quotient, the points of the original pre-processed signal with masks of 1 were averaged to obtain ‘I_o’. The same process was repeated for the generated normal EMG signal to obtain I_n. The intensity quotient (q_i) was then calculated using the formula *q_i* = *abs(I_o – I_n)*.

Similarly, for the duration quotient, the number of signal points with masks of 1 in the original pre-processed signal were combined to obtain ‘n_1’, divided by the number of masks (‘n_m’), resulting in d_o = n_1 / n_m. The process was replicated for the generated normal EMG signal to obtain ‘d_n’, and the duration quotient (‘q_d’). This was calculated as *q_d* = *abs(d_o – d_n)*.

In the two contexts, ‘I_o’ denotes original intensity, ‘I_n’ denotes normal intensity, ‘q_i’ signifies quotient of intensity, ‘abs’ represents the absolute value, ‘d_o’ refers to the duration of original signal, ‘n_m’ stands for number of masks, ‘q_d’ denotes ‘quotient of duration’ and ‘d_n’ represents duration of normal.

## Results

### Clustering performance across all muscle groups and activities

The described methods were uniformly applied to all six muscle classes and four distinct muscle activities. Elaborate findings and outcomes are available in the supplementary file. Table 1 provides a comprehensive record of the most effective clustering techniques for the temporalis, masseter and digastric muscles in relation to maximum mouth opening, maximum lateral excursion, maximum anterior protrusion and chewing. It specifies the respective parameters for these techniques.

**Table 1:** Optimum parameters and silhouette scores for each muscle for jaw movement activity

Jaw movement activity	Muscle	Side	Best-performing model	Optimum parameter	Silhouette score
Maximum mouth opening	Temporalis	Right	GMM	K = 2	0.14
		Left	DBSCAN	Eps = 13.8, MinPts = 51	0.47
	Masseter	Right	DBSCAN	Eps = 13.8, MinPts = 51	0.48
		Left	GMM	K = 2	0.15
	Digastric	Right	K-Means	K = 2	0.85
		Left	DBSCAN	Eps = 13.8, MinPts = 101	0.51
Maximum lateral excursion	Temporalis	Right	GMM	K = 3	0.13
		Left	GMM	K = 3	0.09
	Masseter	Right	DBSCAN	Eps = 13.8, MinPts = 91	0.37
		Left	DBSCAN	Eps = 13.8, MinPts = 71	0.31
	Digastric	Right	DBSCAN	Eps = 13.8, MinPts = 51	0.54
		Left	DBSCAN	Eps = 13.8, MinPts = 61	0.45
Maximum anterior protrusion	Temporalis	Right	GMM	K = 2	0.16
		Left	DBSCAN	Eps = 13.8, MinPts = 61	0.50
	Masseter	Right	DBSCAN	Eps = 13.8, MinPts = 51	0.35
		Left	DBSCAN	Eps = 13.8, MinPts = 51	0.43
	Digastric	Right	K-Means	K = 3	0.10
		Left	GMM	K = 2	0.24
Chewing	Temporalis	Right	K-Means	K = 2	0.12
		Left	DBSCAN	Eps = 13.8, MinPts = 81	0.35
	Masseter	Right	DBSCAN	Eps = 13.8, MinPts = 51	0.36
		Left	K-Means	K = 2	0.11
	Digastric	Right	DBSCAN	Eps = 13.8, MinPts = 51	0.50
		Left	DBSCAN	Eps = 13.8, MinPts = 91	0.41

*Eps*, Epsilon; *MinPts*, minimum samples/Minsamples

### Cluster data output

**Fig. 7:**
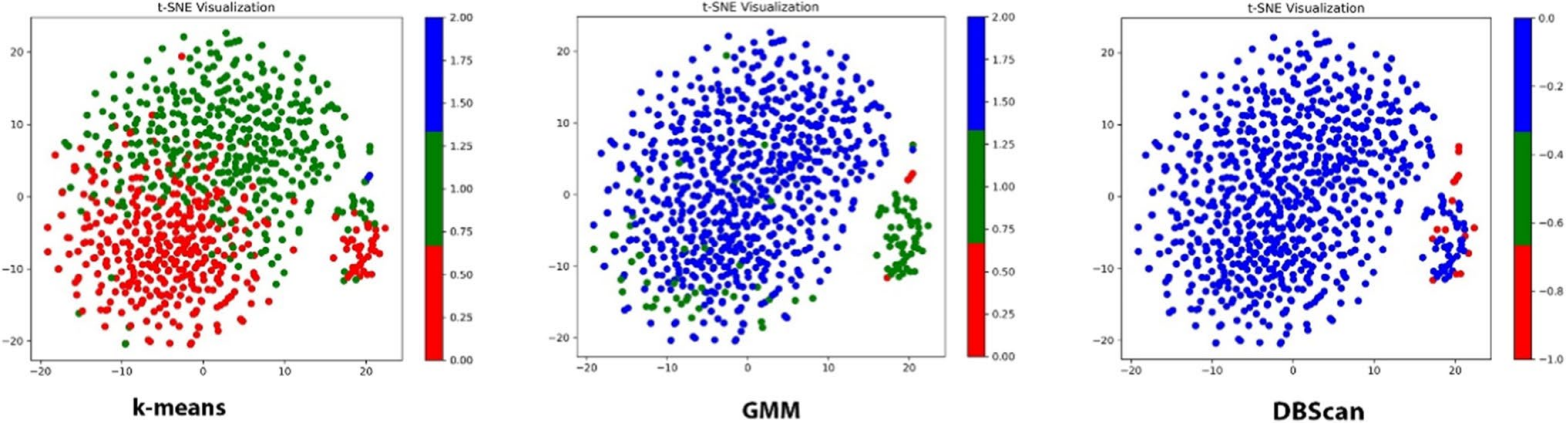
Clustering outputs for right temporalis for lateral excursion across the three methods employed

**Fig. 8:**
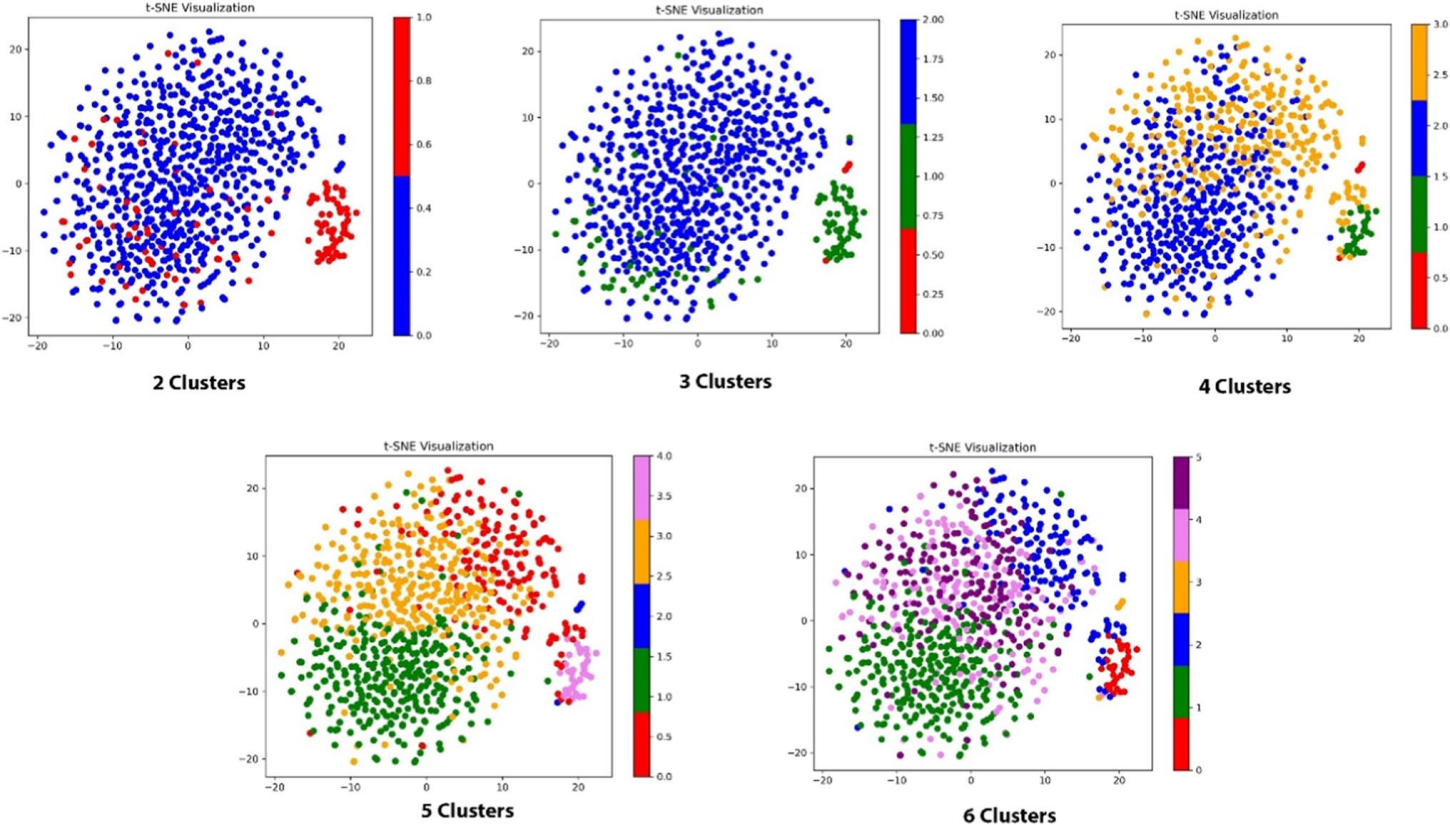
Sample outputs of different levels of GMM clustering for right temporalis during lateral excursion

An example of clustering outputs across the three methods are depicted in Fig. 7, showcasing GMM as the most effective for temporalis during lateral excursion. To visually explore the effectiveness of clustering, GMM was executed for different cluster values of 2, 4, 5 and 6, represented in Fig. 8. Cluster data outputs for each individual muscle across the different activities have been documented within the supplementary file.

### Cluster-centric signal output

**Fig. 9:**
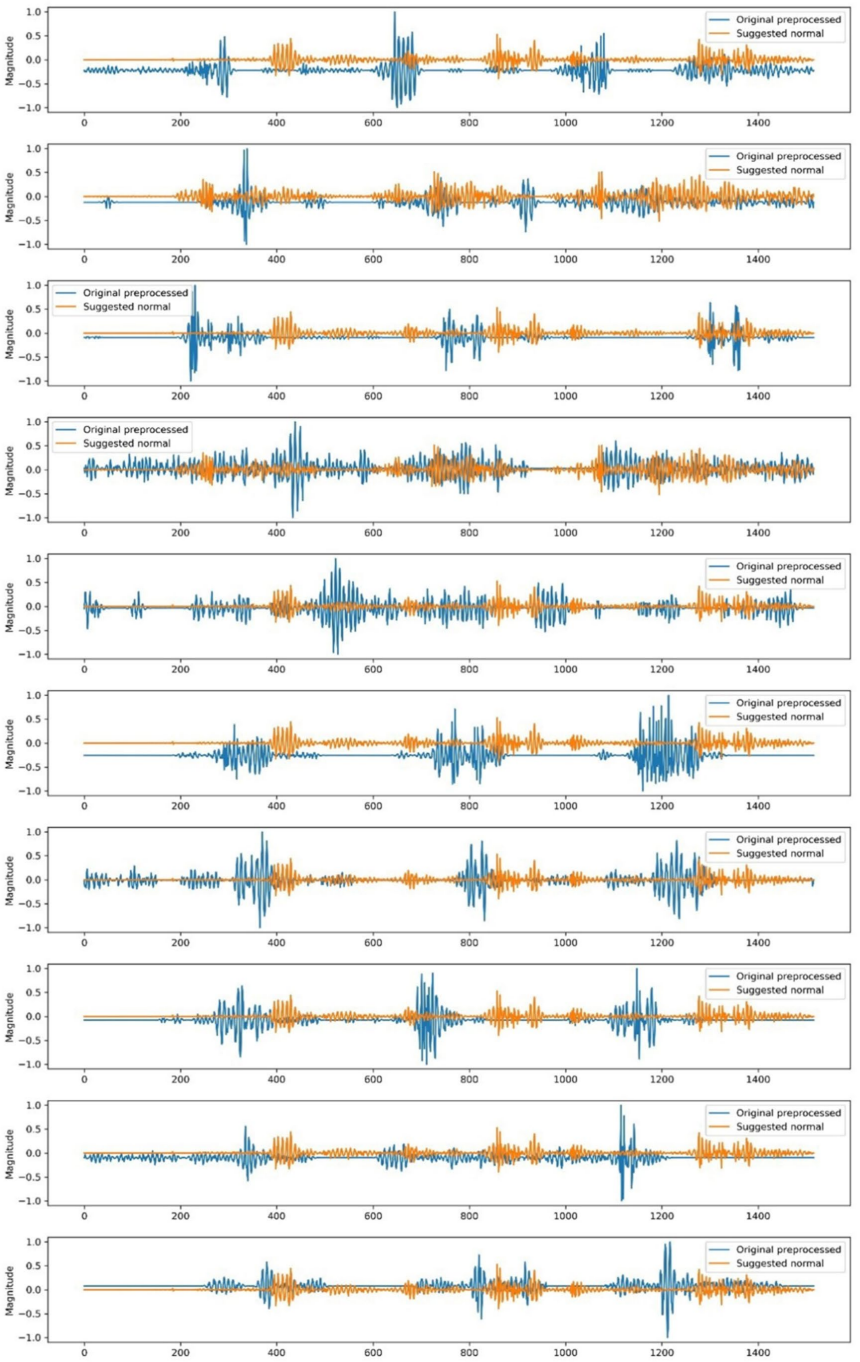
comparison between the GMM cluster-centric signals (orange) and their original unprocessed counterparts (blue) for the right temporalis muscle during maximum lateral excursion of the jaw across 10 participants

**Fig. 10:**
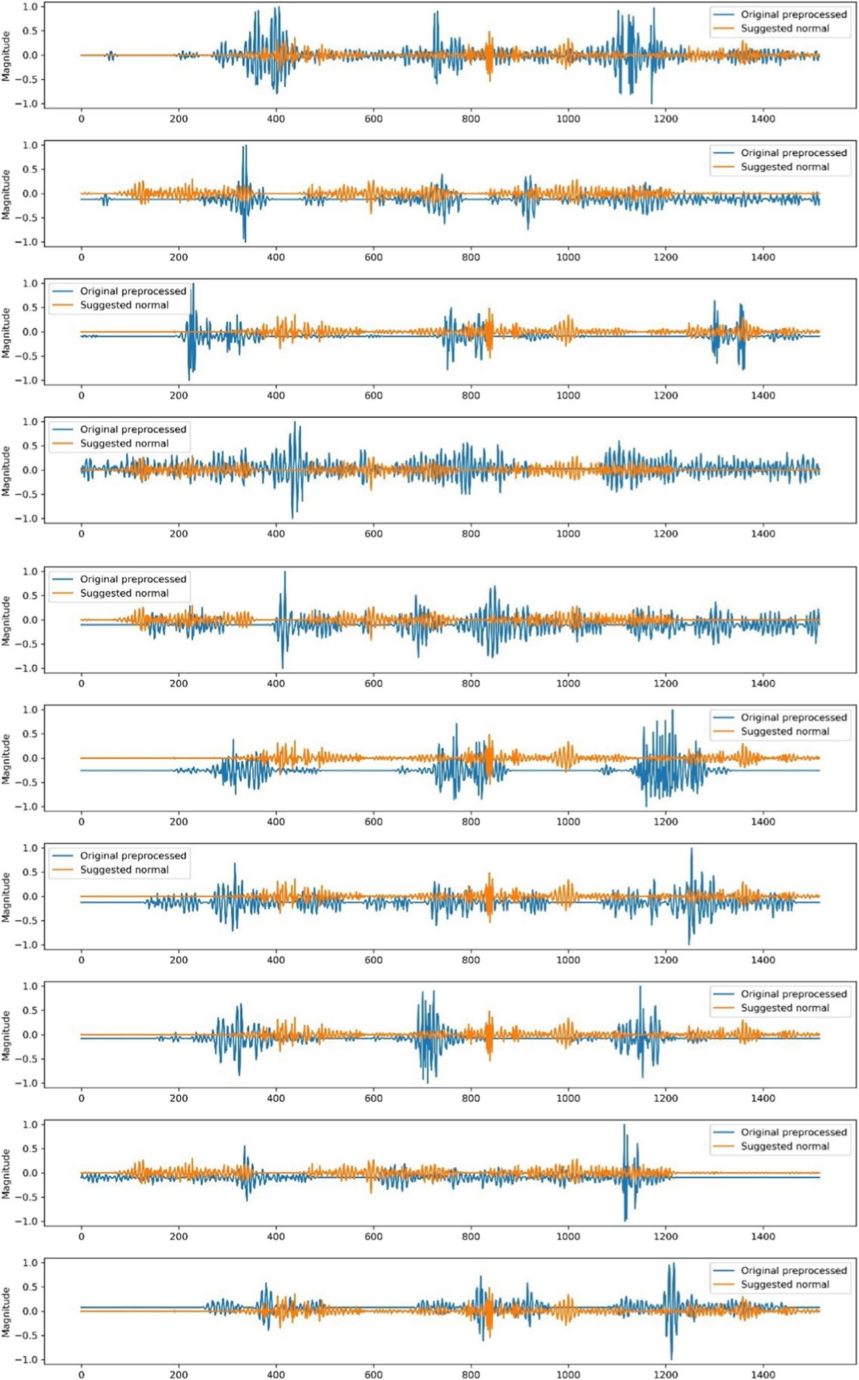
comparison between the DBSCAN-based cluster-centric signals (orange) and their original unprocessed counterparts (blue) for the right masseter muscle during maximum lateral excursion of the jaw across 10 participants

Figure 9 displays the normalised signals created for the right temporalis muscle of 10 participants during lateral excursion using GMM with three clusters of GMM, which was deemed the best-performing model for the said group of muscles on lateral excursion. A similar DBSCAN-based cluster-centric signal was generated for the right masseter muscle of the 10 participants for the same lateral excursion which has been demonstrated in Fig. 10. The visual presents the comparison between the normalised signals generated following clustering and their corresponding original signals obtained and refined from the archived images.

### Generation of normalised signal intensity and activity duration

Table 2 demonstrates the normalised signal intensity and activity duration for the 66 participants. The cluster-centric signals were derived from the best-performing models as described in Table 1.

**Table 2:** Normalised signal intensity and duration quotients validated from data of 66 South Australian participants

Jaw movement activity	Muscle	Side	Muscle signal intensity quotient	Muscle activity duration quotient
Maximum mouth opening	Temporalis	Right	0.149 ± 0.049	55.96 ± 44.80
		Left	0.134 ± 0.048	46.62 ± 44.24
	Masseter	Right	0.117 ± 0.047	72.59 ± 52.10
		Left	0.122 ± 0.048	49.58 ± 35.20
	Digastric	Right	0.120 ± 0.047	51.46 ± 41.37
		Left	0.119 ± 0.452	57.14 ± 40.22
Maximum lateral excursion	Temporalis	Right	0.107 ± 0.052	112.47 ± 84.72
		Left	0.129 ± 0.061	126.31 ± 112.77
	Masseter	Right	0.133 ± 0.069	173.85 ± 126.33
		Left	0.107 ± 0.052	115.81 ± 113.45
	Digastric	Right	0.127 ± 0.058	113.72 ± 107.34
		Left	0.121 ± 0.054	463.63 ± 337.73
Maximum anterior protrusion	Temporalis	Right	0.147 ± 0.058	197.60 ± 171.71
		Left	0.145 ± 0.058	134.45 ± 169.37
	Masseter	Right	0.106 ± 0.492	100.84 ± 152.29
		Left	0.113 ± 0.047	93.43 ± 156.01
	Digastric	Right	0.119 ± 0.050	158.95 ± 188.06
		Left	0.116 ± 0.050	163.87 ± 138.99
Chewing	Temporalis	Right	0.087 ± 0.077	415.07 ± 465.11
		Left	0.090 ± 0.071	621.44 ± 437.69
	Masseter	Right	0.086 ± 0.067	755.86 ± 572.48
		Left	0.091 ± 0.071	684.24 ± 566.80
	Digastric	Right	0.084 ± 0.040	962.92 ± 507.15
		Left	0.087 ± 0.040	824.28 ± 460.01

## Discussion

This study accomplished the successful development and implementation of an open-source deep learning workflow for predictive modelling and the extraction of normalised signal data from EMG images of mandibular muscles. The validity of the workflow was established through the analysis of normal signal intensities and durations in 66 participants engaging in specific muscle activities, revealing distinct differences across each exercise.

### Clinical implications derived from current image-to-signal conversion

Some clinical implications emerge from the examples obtained during the validation of the current workflow. Notably, among the 66 South Australian participants, the right temporalis muscle consistently showed higher intensity, while the masseter muscles exhibited greater durations of activity during mouth opening. These observations could indicate potential parafunctional habits or environmental modifiers, necessitating further correlation with medical history and subsequent investigations. An example is the average output of the left digastric muscle during maximum lateral excursion, where greater soft tissue displacement of the neck in the elderly, and facial hair stubble in men could lead to electrode noise during movement. Nevertheless, the currently proposed method allows for the evaluation of trends in muscle activities for tasks such as chewing. For instance, increased durations of left temporalis activity during chewing were noted in the current report without a substantial difference in signal intensity compared to its right-sided counterpart. This suggests that muscle fatigue may not be a significant factor, making occlusal discrepancies, TMJ dysfunction and parafunctional habits more likely. The normalisation process also helps exclude possible causes such as anatomical variations.

### Feasibility and scalability

The proposed method enables the extraction of normalised signal data from EMG signal images, offering an alternative solution in scenarios where proprietary software might not be available. The choice of K-means was based on its widespread use and computational efficiency. GMM was introduced based on proven effectiveness in handling clusters of varying shapes and densities, offering soft clustering with probabilistic assignments [33]. DBSCAN is a model known for its ability to automatically manage clusters of diverse shapes using a density-based approach [33]. However, the application of K-means for clustering yielded mostly unsatisfactory outcomes, likely because it assumes spherical-shaped clusters that did not align with the actual distribution of data points. In comparison, GMM demonstrated relatively better performance in the current study than K-means by accommodating both spherical and elliptical-shaped clusters. Nonetheless, GMM also exhibited some misplacement of points in the current study, particularly in clusters where the point distribution did not conform to an elliptical form. Enhancements in model performance are anticipated through the scaling of original datasets. Future advancements in clustering methods will necessitate a process of visual inspection, trial and error, and the analysis of evaluation metrics to discern and select the most suitable model for mandibular electromyography.

Each group of muscle activities is inherently unique, making direct comparisons challenging. The absence of a standardised reference for EMG signals, coupled with the limited dataset of only 66 subjects per muscle activity, posed challenges for clustering due to data scarcity. Data augmentation was applied to counteract the data scarcity. Augmentation is a commonly established practice in medical imaging when the dataset quantity provided is insufficient to train a robust AI model [15]. Initially, the EMG-GAN library, based on a variation of Deep Convolutional Generative Adversarial Networks (DCGANs), was explored for the purpose of augmentation, but the outdated codes required substantial debugging, impeding its effective utilisation [34]. This underscores the importance of continuous updates for the long-term viability of a model, a task that can be challenging for less popular open-source models.

### Limitations

With a relatively small sample size of 66 subjects, a considerable amount of synthetic data had to be generated to mirror the original dataset, potentially limiting the representation of outliers or diverse distributions not captured in the synthesised data. The synthetic data contained signal features rather than complete signals, leading to constraints in generating cluster-centric signals after clustering, as only the original observations with real EMG signals were considered. Extreme values in the original dataset, where masks for NeuroKit2 were undetectable, resulted in very high values in muscle activity duration outputs.

The initially generated signals exhibited baseline shifts post-baseline correction due to introduced lag for alignment. Zero padding was added for signals with baseline amplitude below zero, a necessity for algorithms requiring fixed input sizes [35]. Signals varied in length, requiring padding with zeros for uniform input in machine learning training. However, this caused a sudden baseline amplitude increase, addressed by Neurokit2. Discrepancies between original EMG and generated cluster-centric signals arose from inconsistent muscle activity intervals, causing misalignment during cross-correlation-based generation. This resulted in slight variations in muscle activity intervals in the generated signals compared to the originals.

### Future recommendations

Future research can enhance the current workflow scope in three key areas:

1. Addressing the observed negative impact of significantly different resolution formats necessitates incorporating a more varied dataset with diverse digital image properties. Building a comprehensive dataset with diverse image properties is vital for establishing a robust model for signal conversion and normalisation.
2. An alternative approach to the current study involves attempting to generate entire synthetic EMG signals rather than single signal features as observations. This approach has the potential to increase the volume of signals available during cluster-centric signal generation.
3. Integration of newer clustering methods, such as OPTICS [36] and Hierarchical DBSCAN [37], LSTM [38], and a fuzzy logic expert system [39, 40] could be explored to improve the versatility of the current workflow.

## Conclusion

The current study established an effective deep learning workflow, extracting normalised signal data from electromyography (EMG) images and generated quotients for muscle activity duration and functional intensity. Serving as an open-source alternative in the absence of proprietary software, the workflow permits modularity by incorporating diverse clustering algorithms for comparative analysis. This flexibility aids in identifying optimal models for evaluating maxillofacial conditions via mandibular electromyography. However, the incorporation of synthetic data, containing signal features instead of complete signals, limited the generation of post-clustering cluster-centric signals, with periodic misalignments between original EMG observations and their corresponding normalised signals.

### Supplementary Information

Below is the link to the electronic supplementary material.

Supplementary file1 (PDF 5491 KB)

Supplementary file2 - codes_Augmenting_signals (IPYNB 8760 KB)

Supplementary file3 - codes_clustering (IPYNB 2175 KB)

Supplementary file4 - codes_comparisons (IPYNB 850 KB)

Supplementary file5 - codes_extracting_ROI_EMG (IPYNB 327 KB)

Supplementary file6 - codes_generating_quotients (IPYNB 7 KB)

## Data Availability

All data constituting the metadata for the current study has been consolidated into a single Supplementary File. The source codes have also been provided as a supplementary document.

## Abbreviations

*EMG*: Electromyography
*MMO*: Maximum mouth opening
*MLE*: Maximum lateral excursion
*MAP*: Maximum anterior protrusion
*ROI*: Regions of Interest
*Px*: Pixels
*m_ph*: Minimum number of horizontal pixels
*m_pv*: Minimum number of vertical pixels
*Pixel Row Difference*: Number of pixel rows in an extracted image—last encountered white pixel in the black row index
*Moving average*: The process of calculating the average value of pixel intensities in a local neighbourhood around each pixel and replacing the original pixel intensity with this average value
*Padding*: The addition of extra pixels or values around the borders of an image or input data
*CSV*: Comma-separated value
*TSFEL*: Time Series Feature Extraction Library
*Imputation*: The process of estimating or filling in missing values in a dataset
*SDV*: Synthetic Data Vault
*CTGAN*: Conditional Generative Adversarial Network
*TVAE*: Triplet-Based Variational Autoencoder
*t-SNE*: T-distributed Stochastic Neighbour Embedding
*GMM*: Gaussian Mixture Models
*BIC*: Bayesian Information Criterion
*DBSCAN*: Density-Based Spatial Clustering of Applications with Noise
*OPTICS*: Ordering points to identify the clustering structure
*LSTM*: Long short-term memory

## References

[1] Farook TH, Rashid F, Ahmed S, Dudley J (2023) Clinical machine learning in parafunctional and altered functional occlusion: A systematic review. J Prosthet Dent 10.1016/j.prosdent.2023.01.013 36801145

[2] Farook TH, Rashid F, Alam MK, Dudley J (2022) Variables influencing the device-dependent approaches in digitally analysing jaw movement—a systematic review. Clin Oral Investig 1–16 10.1007/s00784-022-04835-w 36577849

[3] Farook TH, Dudley J (2023) Automation and deep (machine) learning in temporomandibular joint disorder radiomics. A systematic review. J Oral Rehabil 10.1111/joor.13440 36843391

[4] Østensvik T, Veiersted KB, Nilsen P (2009). A method to quantify frequency and duration of sustained low-level muscle activity as a risk factor for musculoskeletal discomfort. J Electromyogr Kinesiol.

[5] Rashi T, Yom-Tov E (2023). Ethics of medical archival internet research data. J Med Internet Res.

[6] Wolf SM, Crock BN, Van Ness B, Lawrenz F, Kahn JP, Beskow LM, Cho MK, Christman MF, Green RC, Hall R (2012). Managing incidental findings and research results in genomic research involving biobanks and archived data sets. Genet Med.

[7] Hochberg I, Allon R, Yom-Tov E (2020). Assessment of the frequency of online searches for symptoms before diagnosis: analysis of archival data. J Med Internet Res.

[8] Wu Y, Rangayyan RM, Zhou Y, Ng S-C (2009). Filtering electrocardiographic signals using an unbiased and normalized adaptive noise reduction system. Med Eng Phys.

[9] Barron SM, Diaz TO, Pozzi F, Vasilopoulos T, Nichols JA (2022). Linear relationship between electromyography and shear wave elastography measurements persists in deep muscles of the upper extremity. J Electromyogr Kinesiol.

[10] Jonkman AH, Warnaar RSP, Baccinelli W, Carbon NM, D’Cruz RF, Doorduin J, van Doorn JLM, Elshof J, Estrada-Petrocelli L, Graßhoff J (2024). Analysis and applications of respiratory surface EMG: report of a round table meeting. Crit Care.

[11] Mahmud S, Chowdhury MEH, Kiranyaz S, Al Emadi N, Tahir AM, Hossain MS, Khandakar A, Al-Maadeed S (2024). Restoration of motion-corrupted EEG signals using attention-guided operational CycleGAN. Eng Appl Artif Intell.

[12] Reaz MBI, Hussain MS, Mohd-Yasin F (2006). Techniques of EMG signal analysis: detection, processing, classification and applications. Biol Proced Online.

[13] Chen M, Cheng Q, Feng X, Zhao K, Zhou Y, Xing B, Tang S, Wang R, Duan J, Wang J (2024). Optimized variational mode decomposition algorithm based on adaptive thresholding method and improved whale optimization algorithm for denoising magnetocardiography signal. Biomed Signal Process Control.

[14] Makowski D, Pham T, Lau ZJ, Brammer JC, Lespinasse F, Pham H, Schölzel C, Chen SHA (2021) NeuroKit2: A Python toolbox for neurophysiological signal processing. Behav Res Methods 1–8 10.3758/s13428-020-01516-y 33528817

[15] Chlap P, Min H, Vandenberg N, Dowling J, Holloway L, Haworth A (2021). A review of medical image data augmentation techniques for deep learning applications. J Med Imaging Radiat Oncol.

[16] Hazra D, Byun Y-C (2020). SynSigGAN: Generative adversarial networks for synthetic biomedical signal generation. Biology (Basel).

[17] Nowroozilarki Z, Mortazavi BJ, Jafari R (2023) Variational autoencoders for biomedical signal morphology clustering and noise detection. IEEE J Biomed Health Inform 10.1109/JBHI.2023.3320585 PMC10984704 37768790

[18] Barandas M, Folgado D, Fernandes L, Santos S, Abreu M, Bota P, Liu H, Schultz T, Gamboa H (2020). TSFEL: time series feature extraction library. SoftwareX.

[19] Khan TI, Sakib N, Hassan MM, Ide S (2024). Gaussian mixture model in clustering acoustic emission signals for characterizing osteoarthritic knees. Biomed Signal Process Control.

[20] Çerçi Ç, Temeltaş H (2018) Feature extraction of EMG signals, classification with ANN and kNN algorithms, in: 2018 26th Signal Processing and Communications Applications Conference (SIU), IEEE, 1–4

[21] Farag W (2020). Road-objects tracking for autonomous driving using lidar and radar fusion. J Electr Eng.

[22] Farook TH, Ahmed S, Talukder MSI, Dudley J (2023). A 3D printed electronic wearable device to generate vertical, horizontal and phono-articulatory jaw movement parameters: A concept implementation. PLoS One.

[23] Paul R, Hoque ASML (2010) Clustering medical data to predict the likelihood of diseases, in: 2010 Fifth International Conference on Digital Information Management (ICDIM), IEEE, pp. 44–49

[24] Lehne M, Sass J, Essenwanger A, Schepers J, Thun S (2019). Why digital medicine depends on interoperability. NPJ Digit Med.

[25] Boulanger A (2005). Open-source versus proprietary software: is one more reliable and secure than the other?. IBM Syst J.

[26] Norgeot B, Quer G, Beaulieu-Jones BK, Torkamani A, Dias R, Gianfrancesco M, Arnaout R, Kohane IS, Saria S, Topol E (2020). Minimum information about clinical artificial intelligence modeling: the MI-CLAIM checklist. Nat Med.

[27] Patki N, Wedge R, Veeramachaneni K (2016) The synthetic data vault, in: 2016 IEEE International Conference on Data Science and Advanced Analytics (DSAA), IEEE, pp. 399–410

[28] Verleysen M, François D (2005) The curse of dimensionality in data mining and time series prediction, in: International Work-Conference on Artificial Neural Networks, Springer, pp. 758–770

[29] Van der Maaten L, Hinton G (2008) Visualizing data using t-SNE. J Mach Learn Res 9

[30] Rousseeuw PJ (1987). Silhouettes: a graphical aid to the interpretation and validation of cluster analysis. J Comput Appl Math.

[31] Rahmah N, Sitanggang IS (2016) Determination of optimal epsilon (eps) value on dbscan algorithm to clustering data on peatland hotspots in sumatra, in: IOP Conf Ser Earth Environ Sci, IoP Publishing, p. 012012

[32] Sander J, Ester M, Kriegel H-P, Xu X (1998). Density-based clustering in spatial databases: the algorithm gdbscan and its applications. Data Min Knowl Discov.

[33] Lyu B, Wu W, Hu Z (2021). A novel bidirectional clustering algorithm based on local density. Sci Rep.

[34] AnicetZanini R, Luna Colombini E (2020). Parkinson’s disease EMG data augmentation and simulation with DCGANs and style transfer. Sensors.

[35] Ozan W, Grammenos R, Darwazeh I (2020). Zero padding or cyclic prefix: evaluation for non-orthogonal signals. IEEE Commun Lett.

[36] Agrawal KP, Garg S, Sharma S, Patel P (2016). Development and validation of OPTICS based spatio-temporal clustering technique. Inf Sci (N Y).

[37] McInnes L, Healy J, Astels S (2017). hdbscan: Hierarchical density based clustering. J Open Source Softw.

[38] Liu Y, Bao Y (2023). Real-time remote measurement of distance using ultra-wideband (UWB) sensors. Autom Constr.

[39] Dobrić G, Žarković M (2021). Fuzzy expert system for metal-oxide surge arrester condition monitoring. Electr Eng.

[40] Ahmed HO (2021) 17.16 GOPS\W sustainable FLS-based wireless sensor network for surveillance system using FPGA, in: 2021 Integrated Communications Navigation and Surveillance Conference (ICNS), IEEE, pp. 1–10

