# Supplementary material for: Deep learning and predictive modelling for generating normalised muscle function parameters from signal images of mandibular electromyography
Source: Med Biol Eng Comput. 2024 Feb 20;62(6):1763–79. doi: 10.1007/s11517-024-03047-6 (PMC11076382; doi:10.1007/s11517-024-03047-6)
Supplement: Supplementary file 1 — Supplementary file1 (PDF 5491 KB) [file 11517_2024_3047_MOESM1_ESM.pdf]

Deep learning and predictive modelling for generating normalised muscle function intensity and activity duration data from archived signal images of mandibular electromyography

Contents

Maximum Mouth Opening 2

Right Temporalis 2

Left Temporalis 9

Right Masseter Muscle 16

Left Masseter Muscle 23

Right Digastric Muscle 30

Left Digastric Muscle 37

Maximum Anterior Protrusion 44

Right Temporalis 44

Left Temporalis 51

Right Masseter Muscle 58

Left Masseter Muscle 65

Right Digastric Muscle 72

Left Digastric Muscle 79

Chewing 86

Right Temporalis 86

Left Temporalis 93

Right Masseter Muscle 100

Left Masseter Muscle 107

Right Digastric Muscle 114

Left Digastric Muscle 121

Maximum Lateral Excursion 128

Right Temporalis 128

Left Temporalis 135

Right Masseter Muscle 142

Left Masseter Muscle 149

Right Digastric Muscle 156

Left Digastric Muscle 163

Normalised signal intensity quotient for muscle activity in different functions 170

Normalised muscle activity duration quotients for muscles during different exercises 174

Maximum Mouth Opening

Right Temporalis

Data visualization:

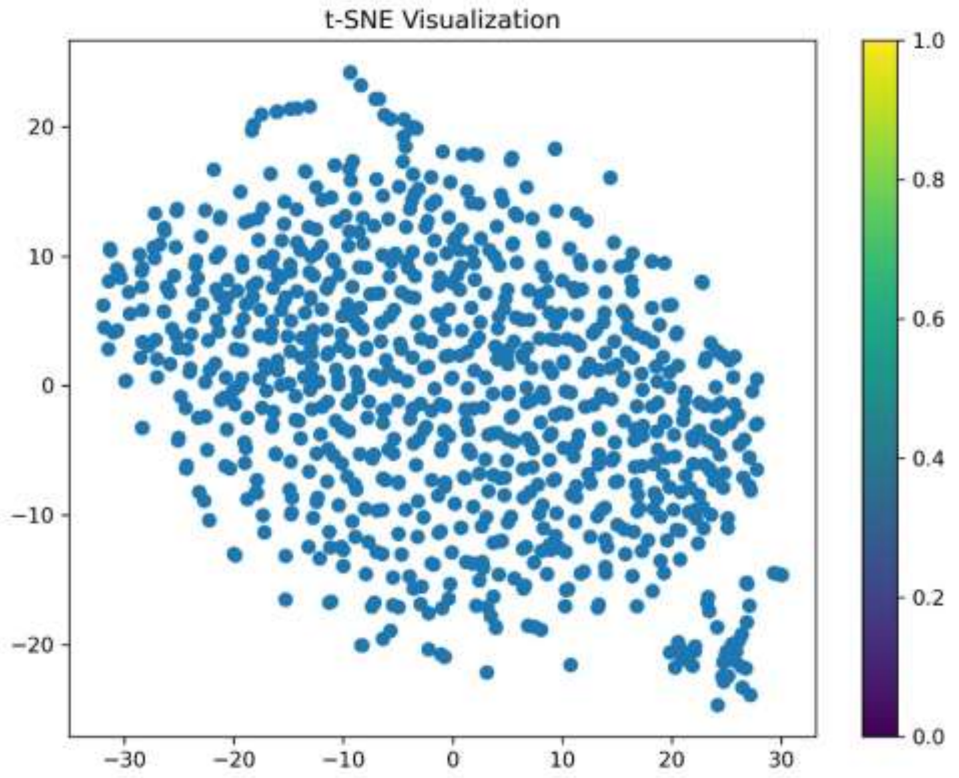

K-Means Elbow and Silhouette:

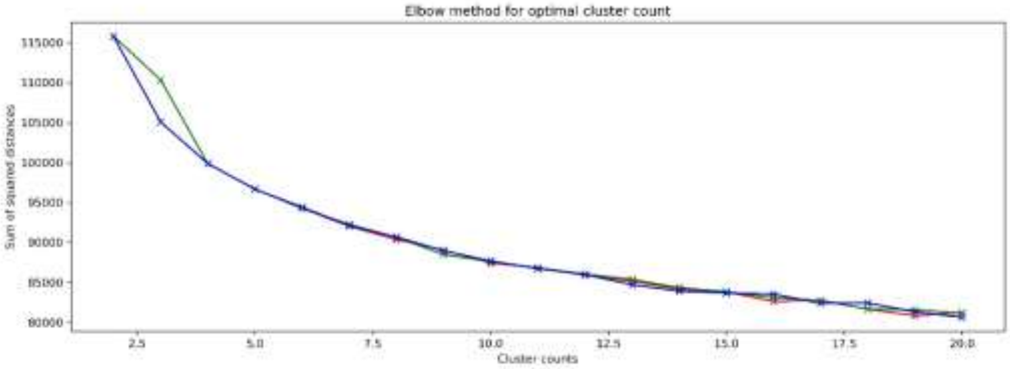

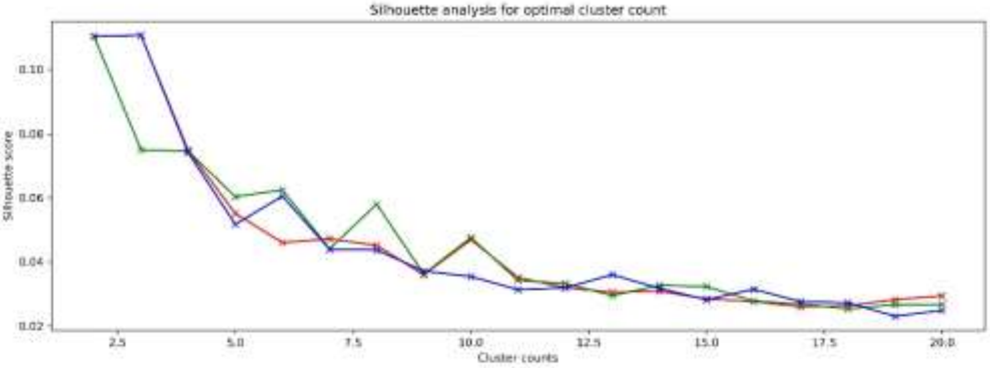

K-Means clustering:

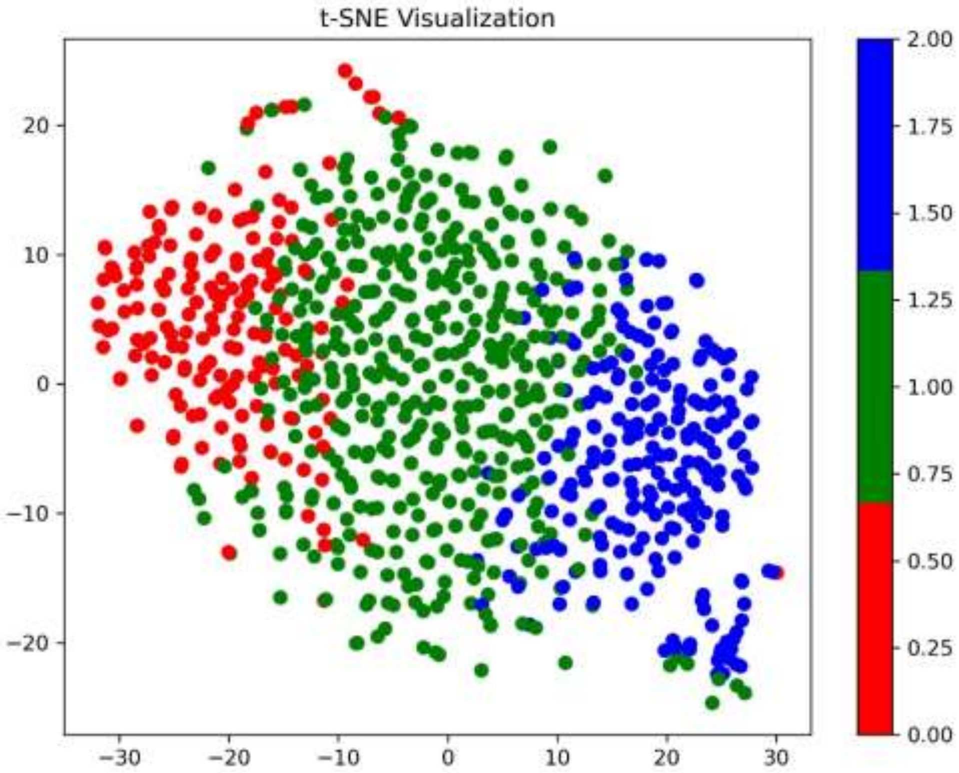

GMM Silhouette and BIC:

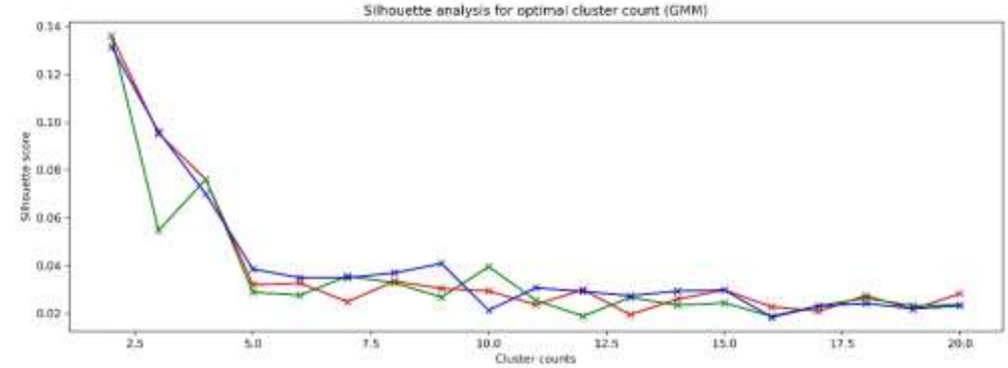

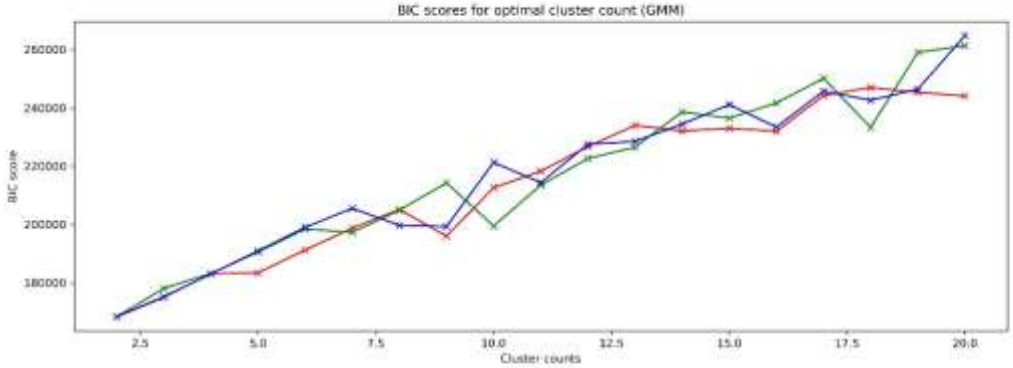

GMM Clustering:

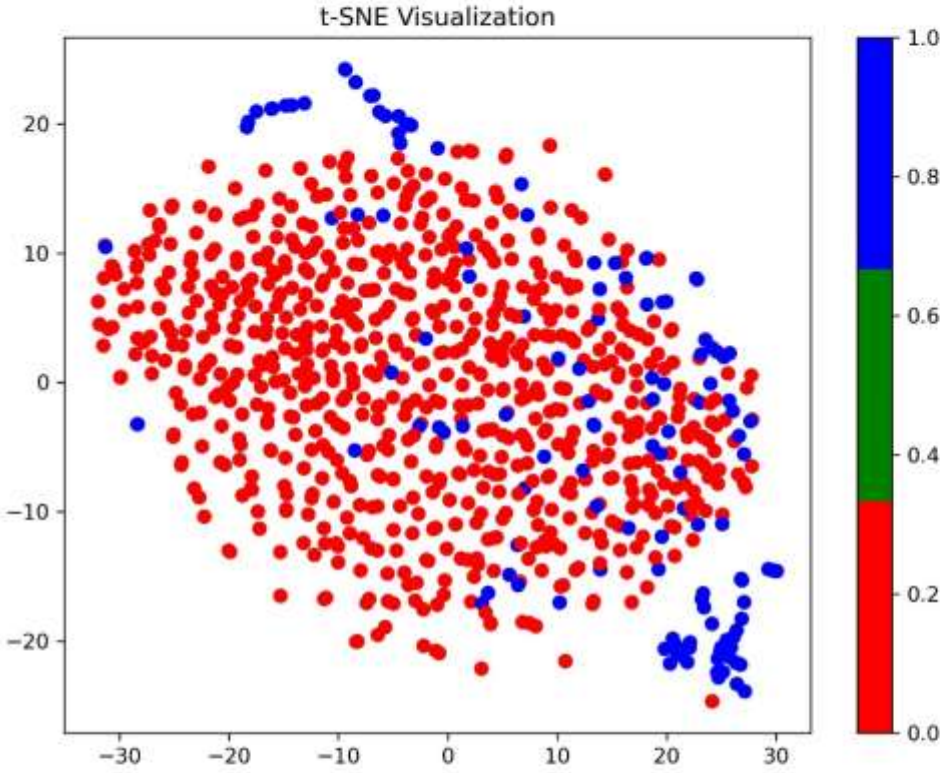

DBSCAN epsilon:

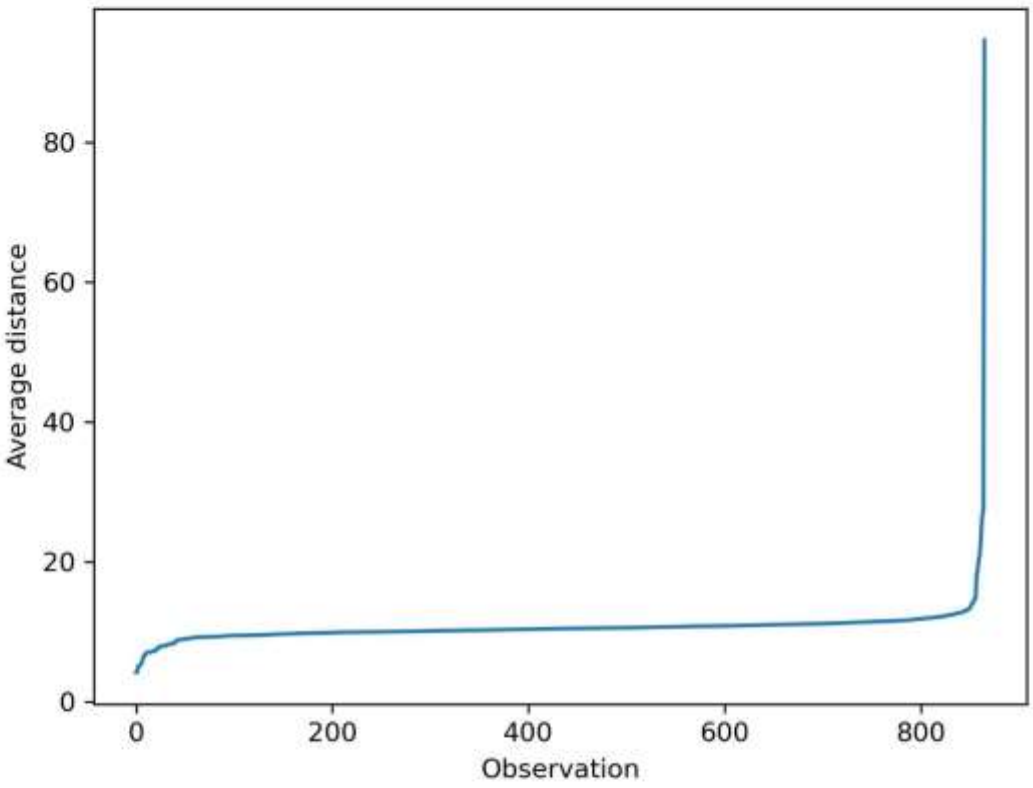

DBSCAN – best based on Davies Bouldin:

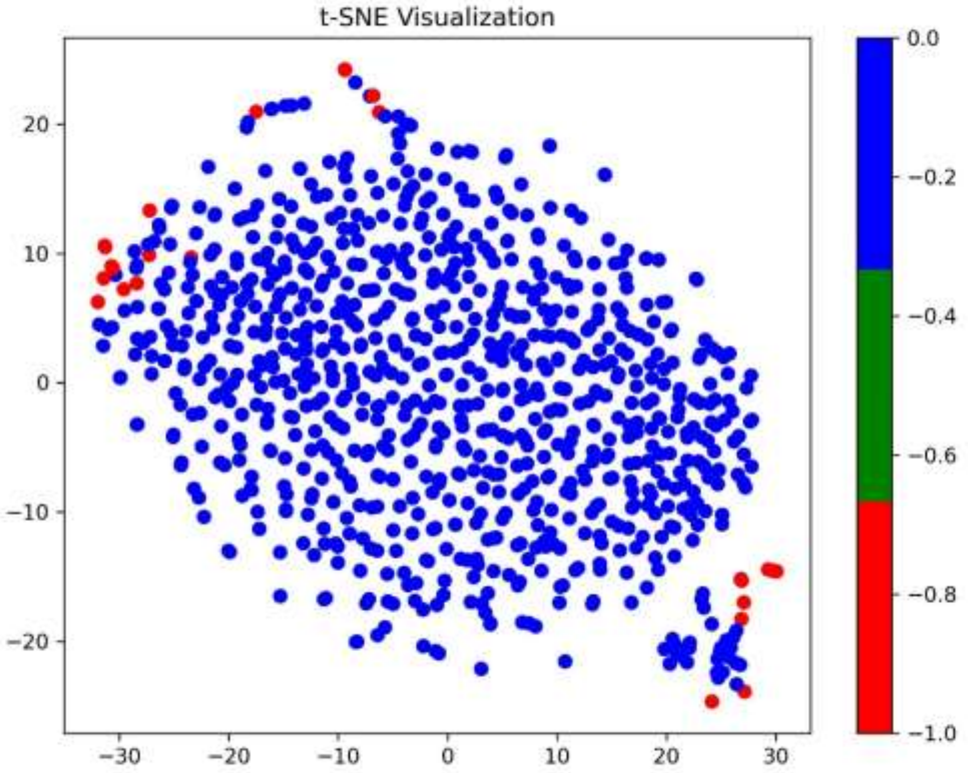

DBSCAN – Best based on Silhouette:

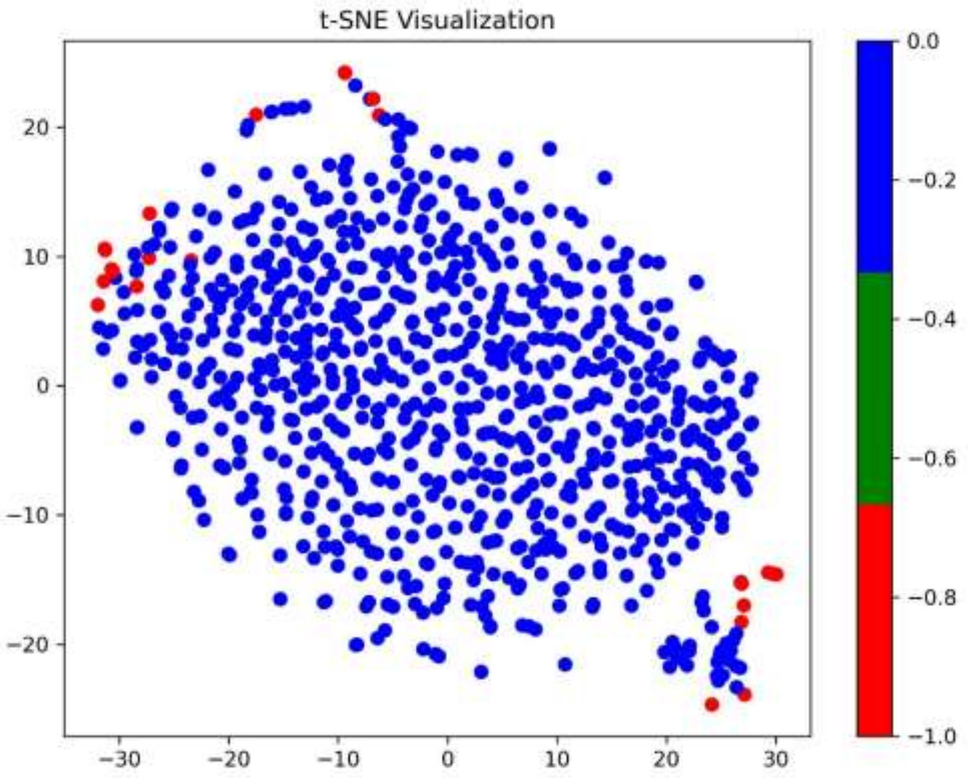

Best model – GMM with k = 2

Left Temporalis

Data visualization:

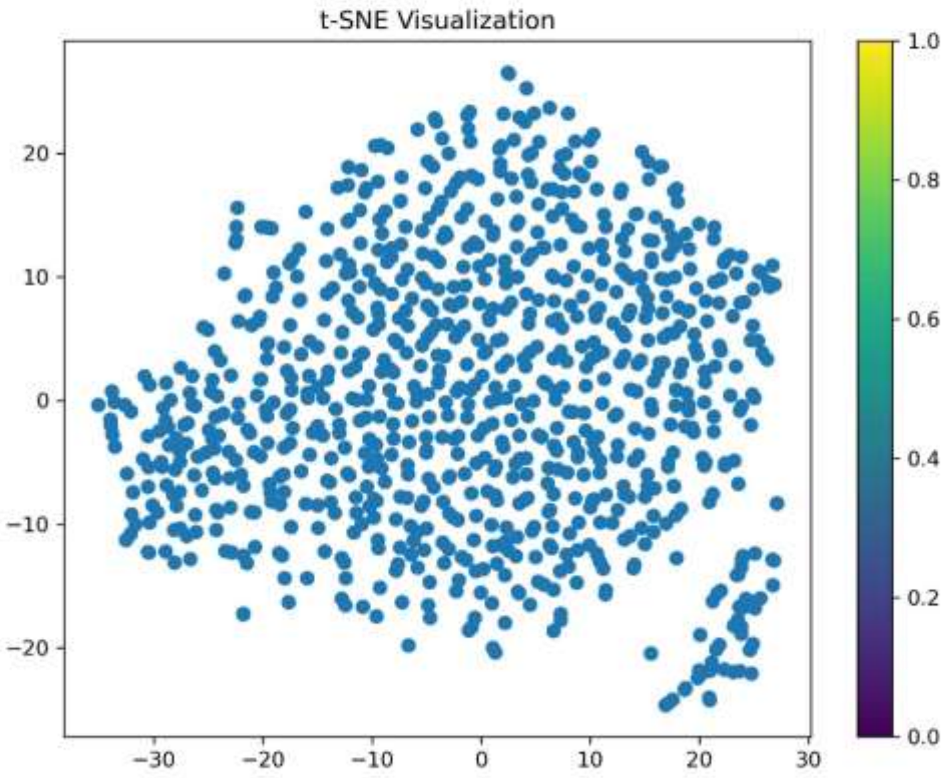

K-Means Elbow and Silhouette:

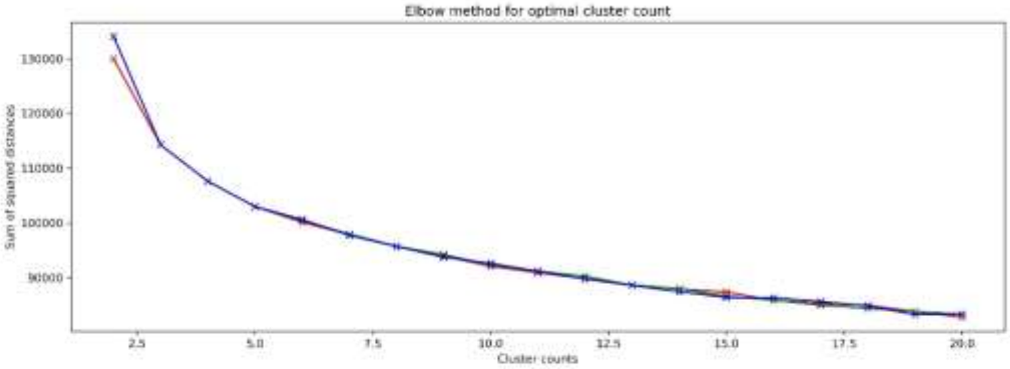

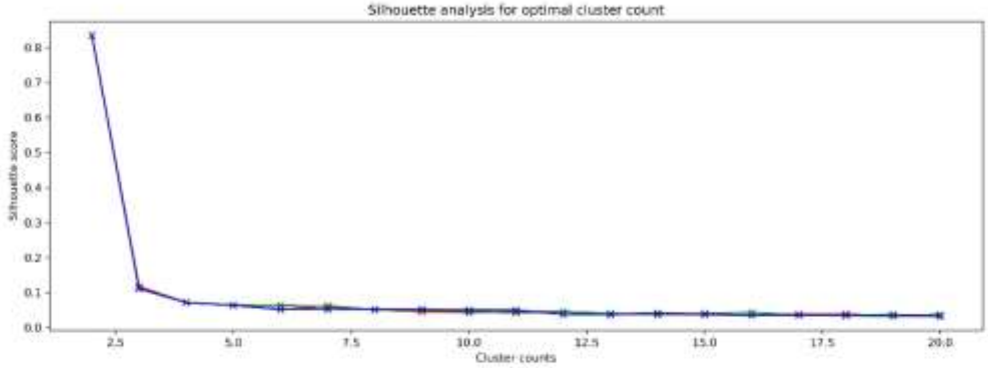

K-Means clustering:

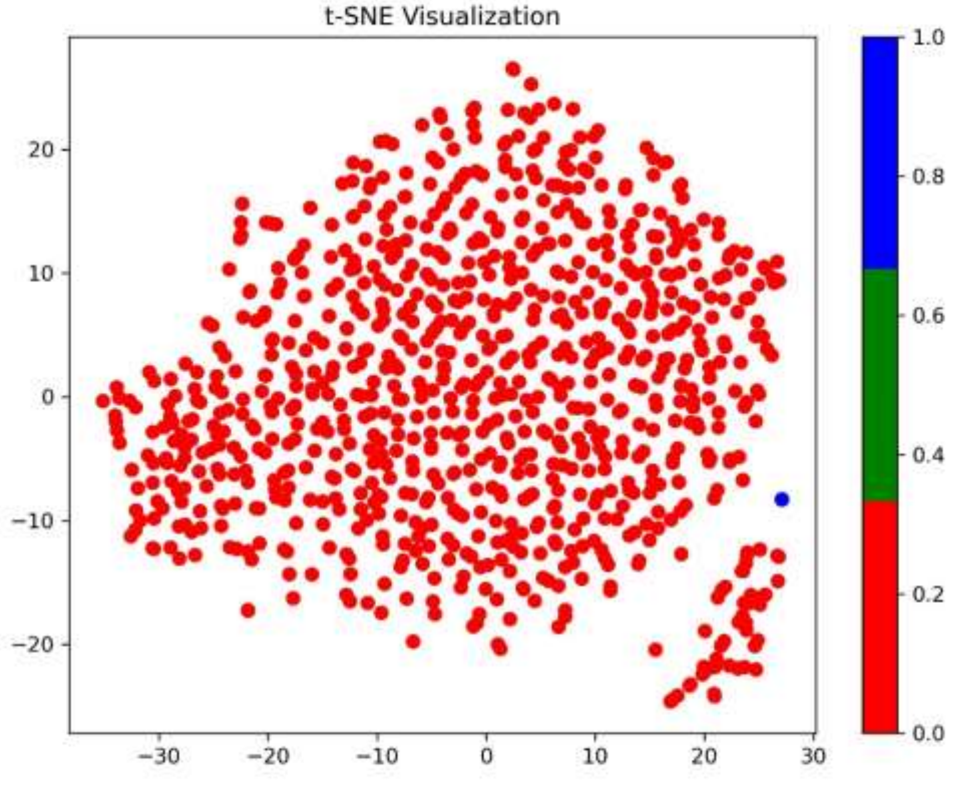

GMM Silhouette and BIC:

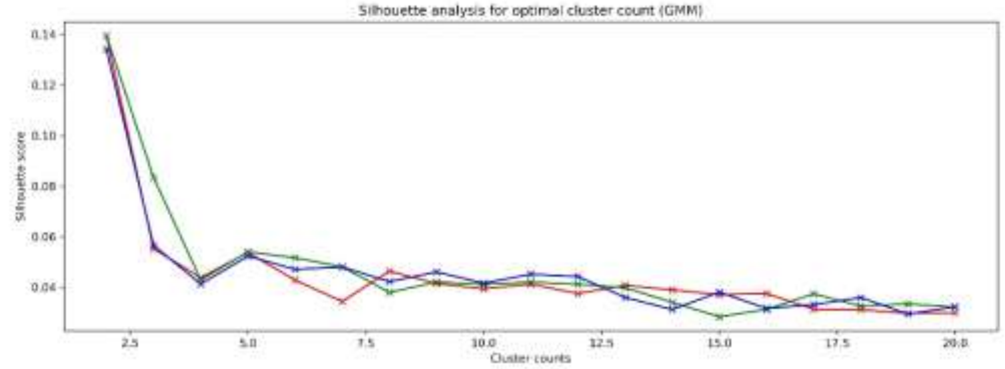

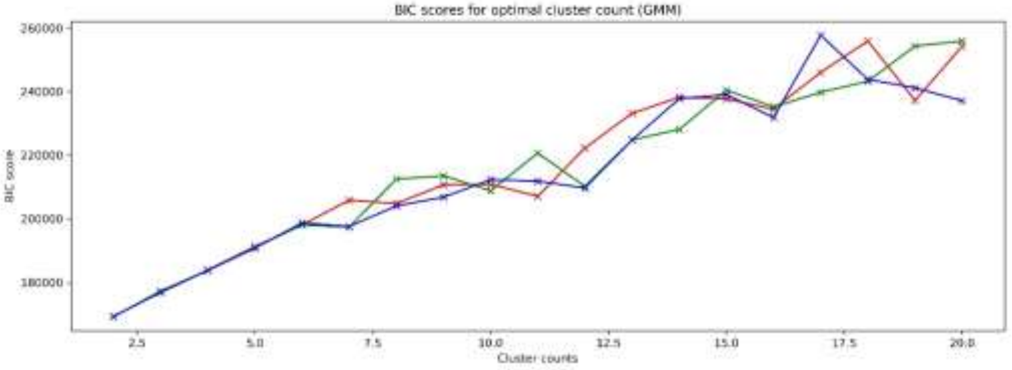

GMM Clustering:

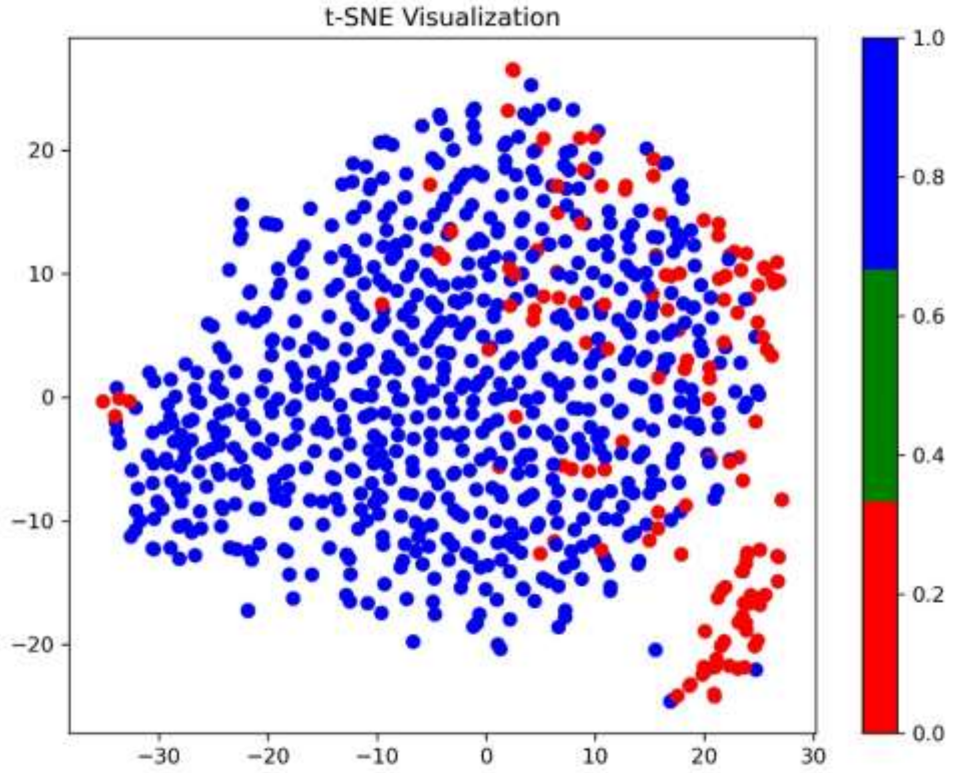

DBSCAN epsilon:

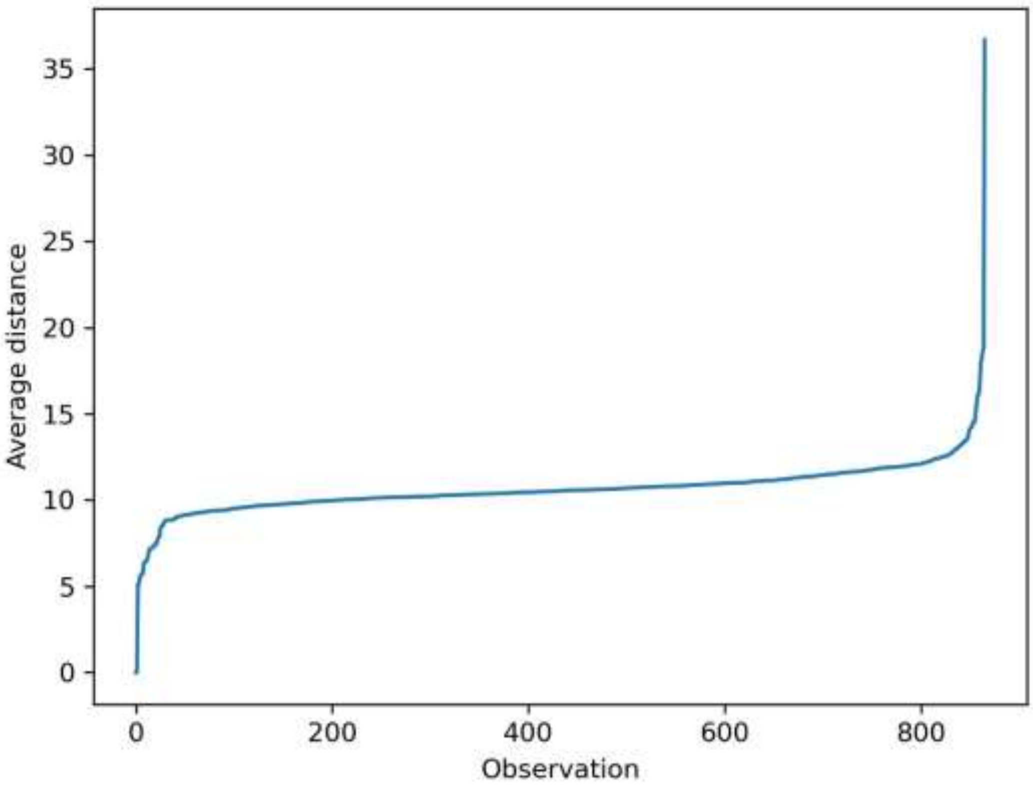

DBSCAN – best based on Davies Bouldin

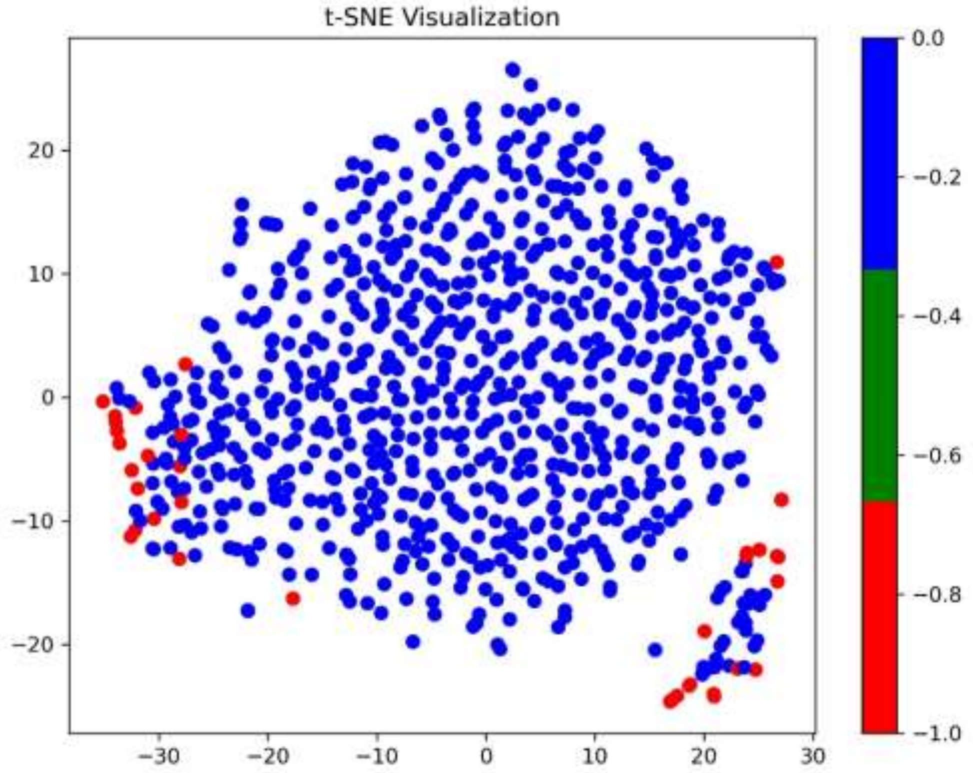

DBSCAN – Best based on Silhouette:

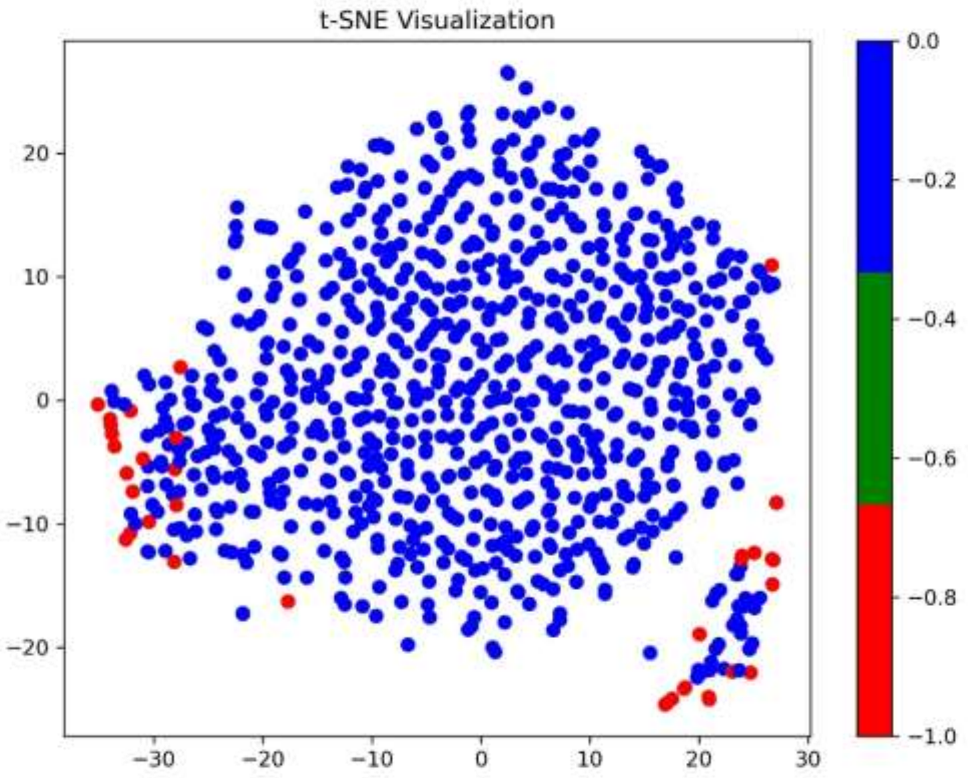

Best model – DBSCAN with Epsilon = 13.8, Min Points = 51.

Right Masseter Muscle

Data visualization:

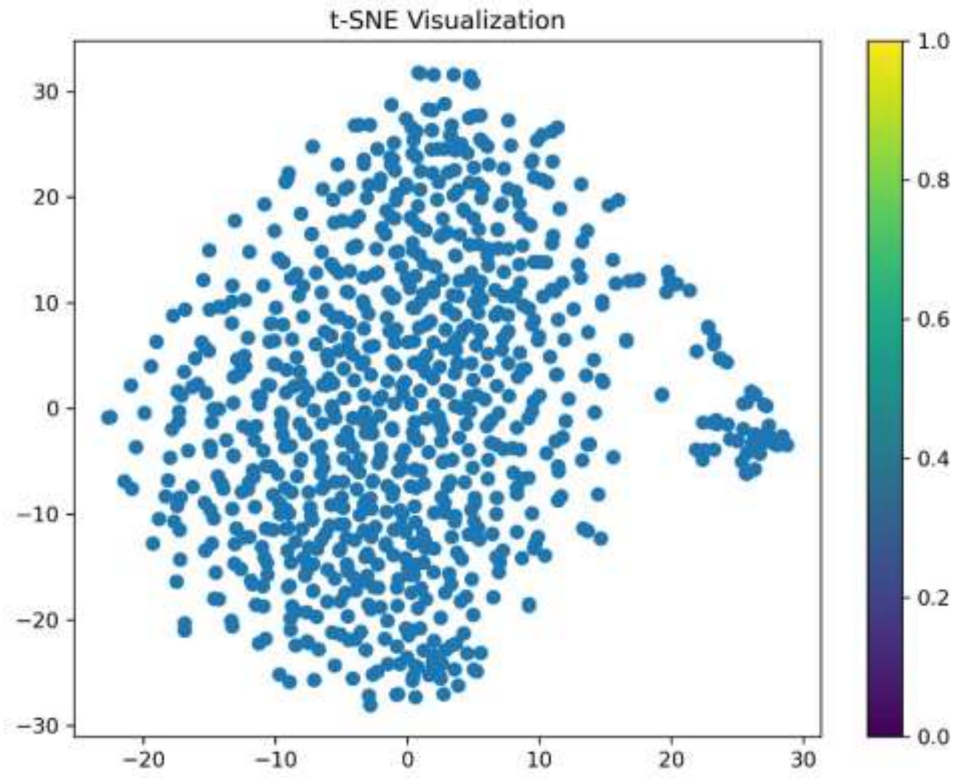

K-Means Elbow and Silhouette:

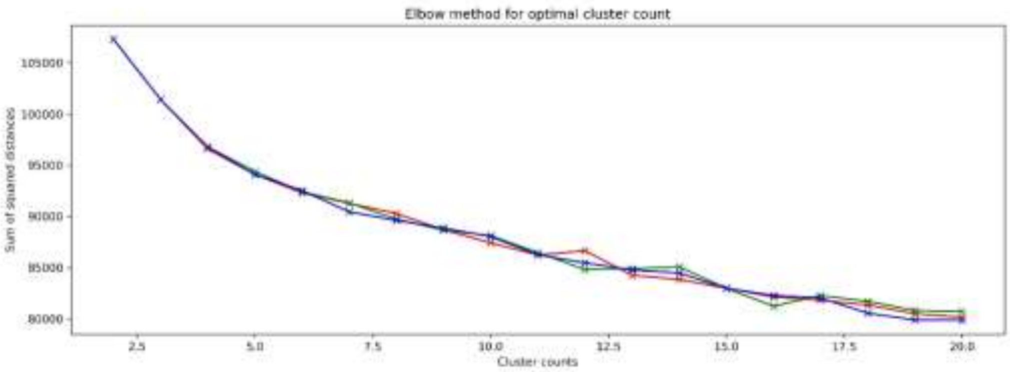

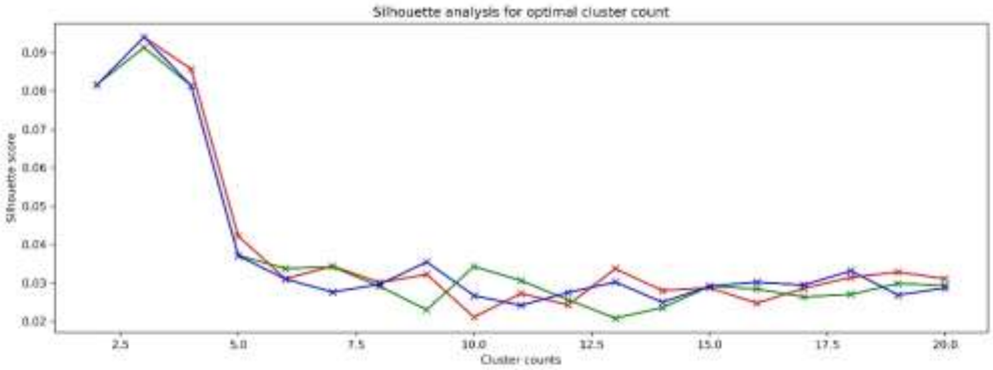

K-Means clustering:

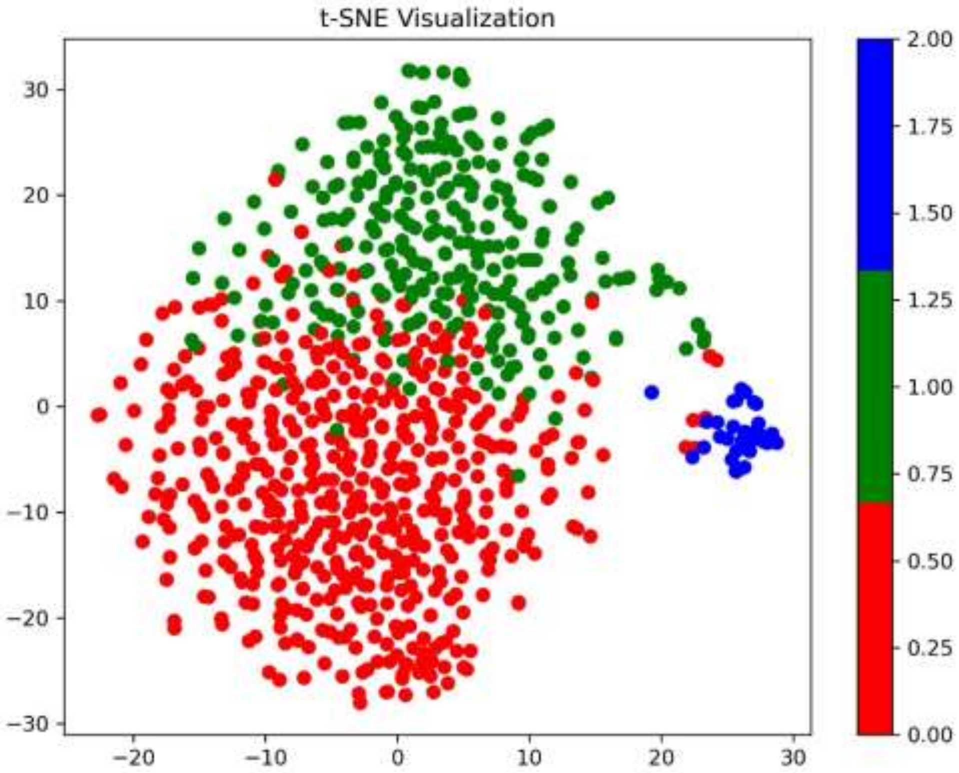

GMM Silhouette and BIC:

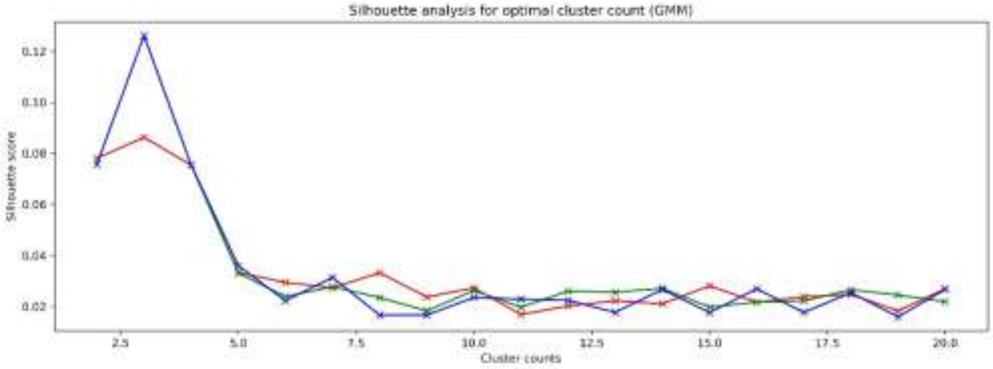

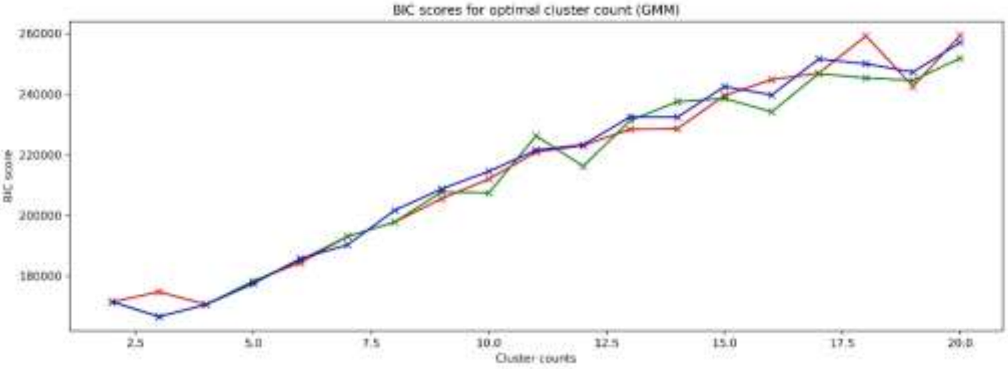

GMM Clustering:

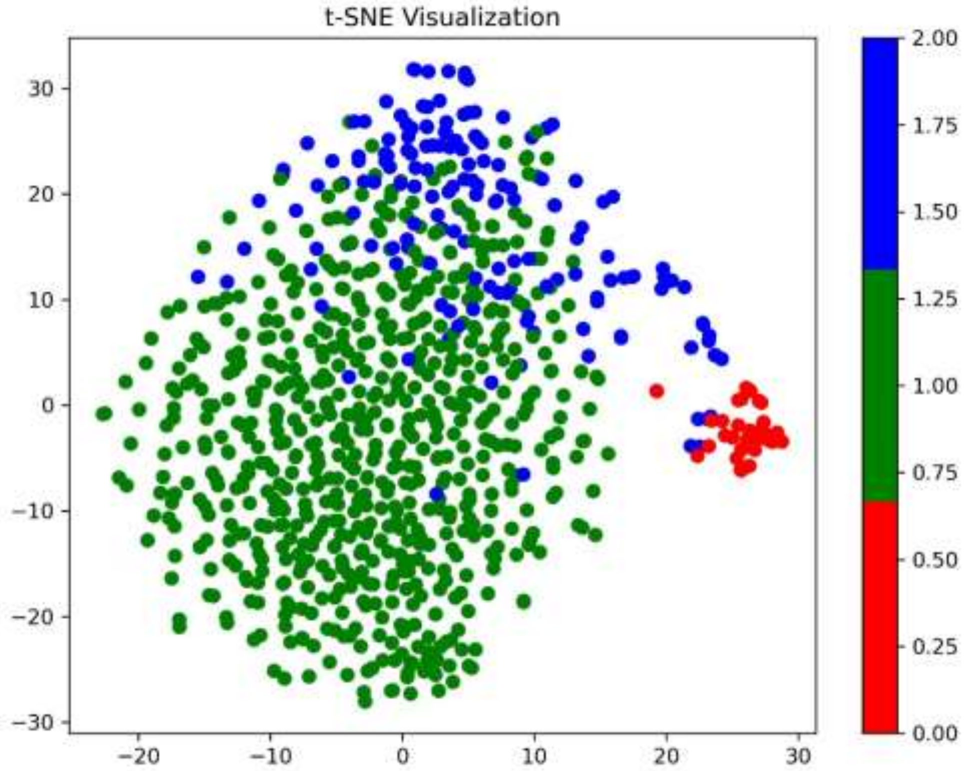

DBSCAN epsilon:

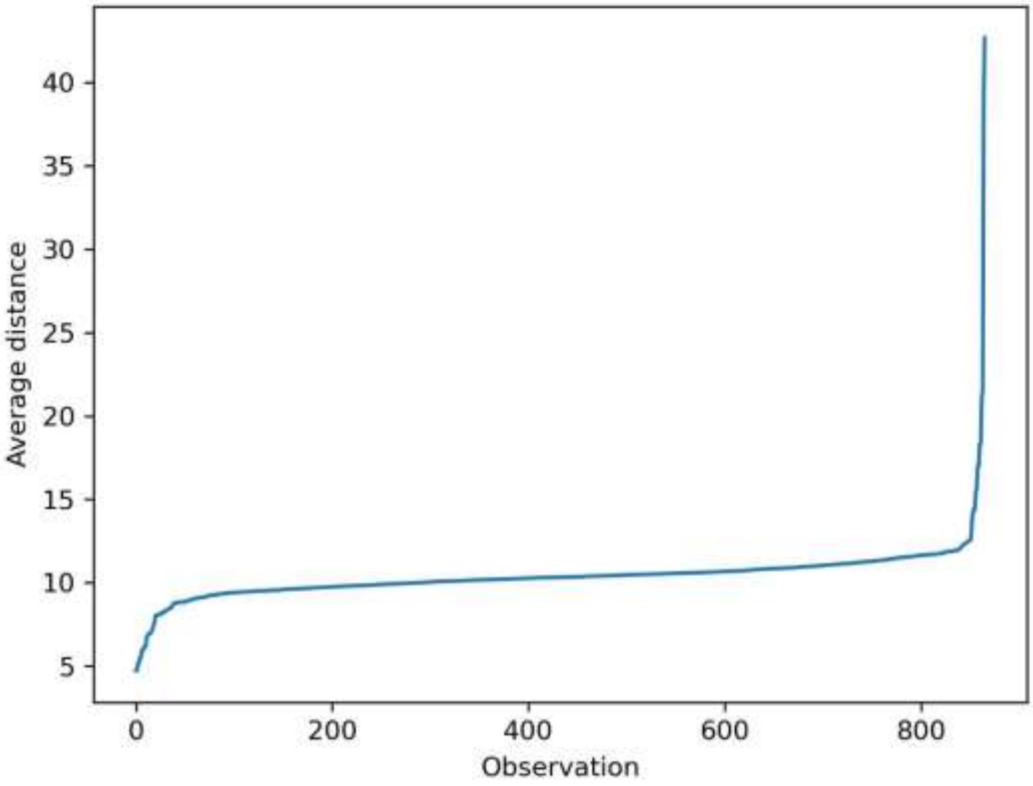

DBSCAN – best based on Davies Bouldin

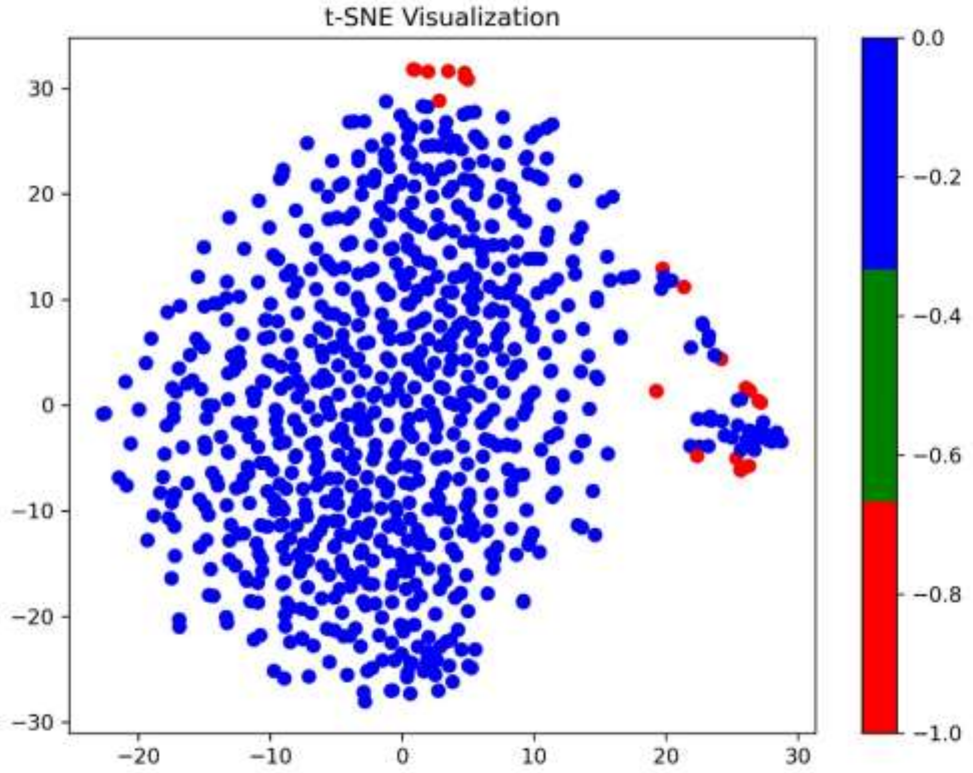

DBSCAN – Best based on Silhouette:

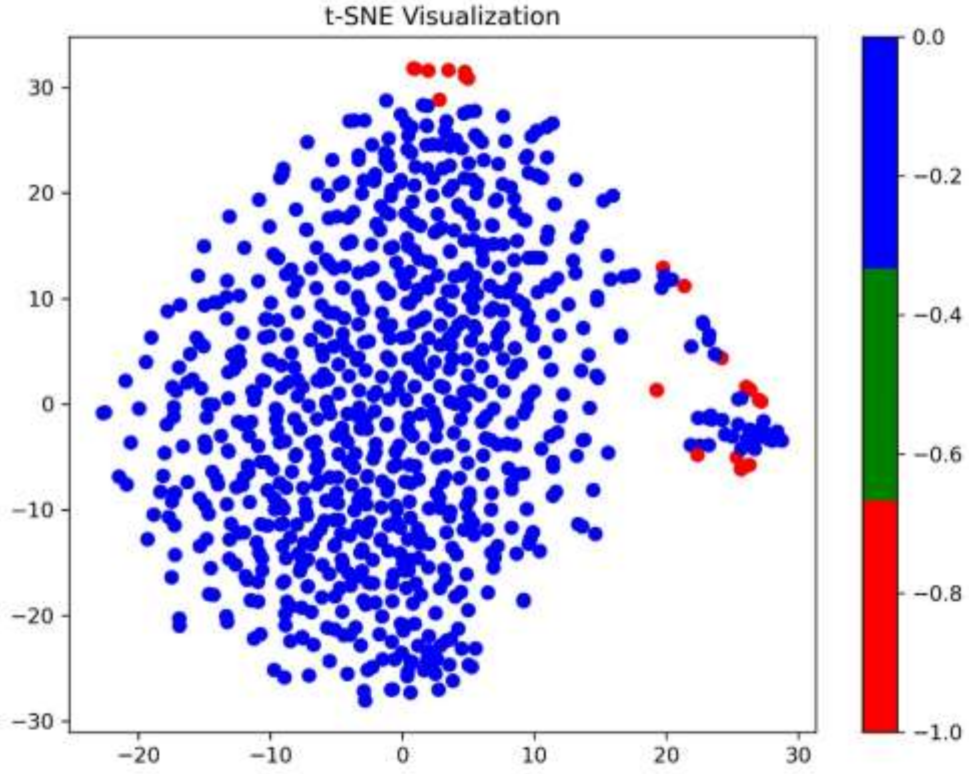

Best model – DBSCAN with Epsilon = 13.8, Min Points = 51.

Left Masseter Muscle

Data visualization:

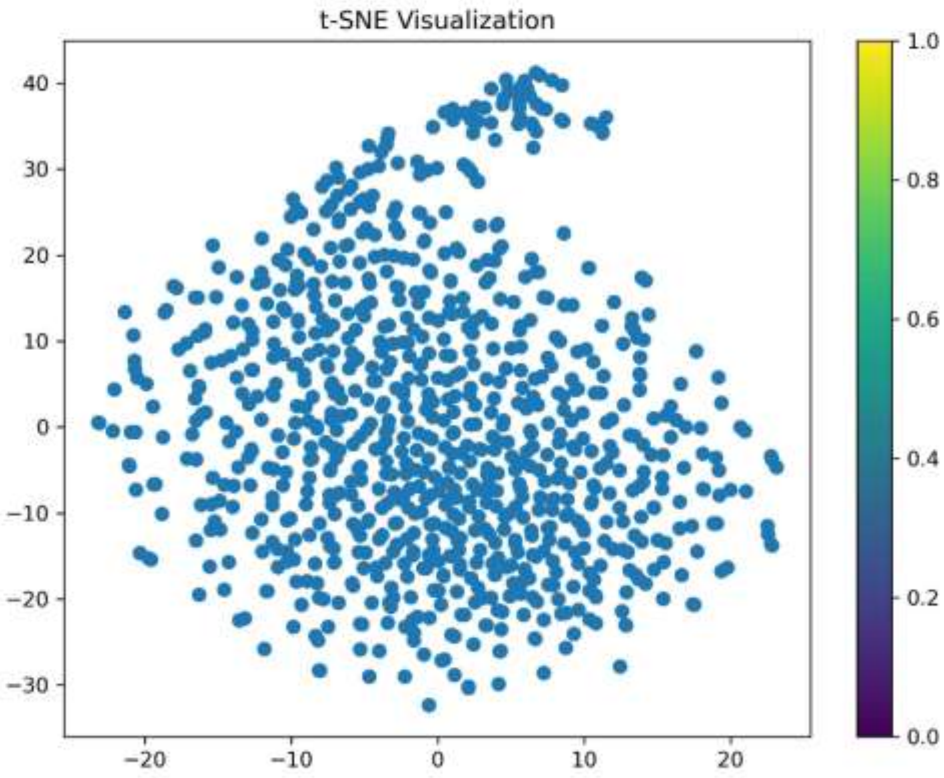

K-Means Elbow and Silhouette:

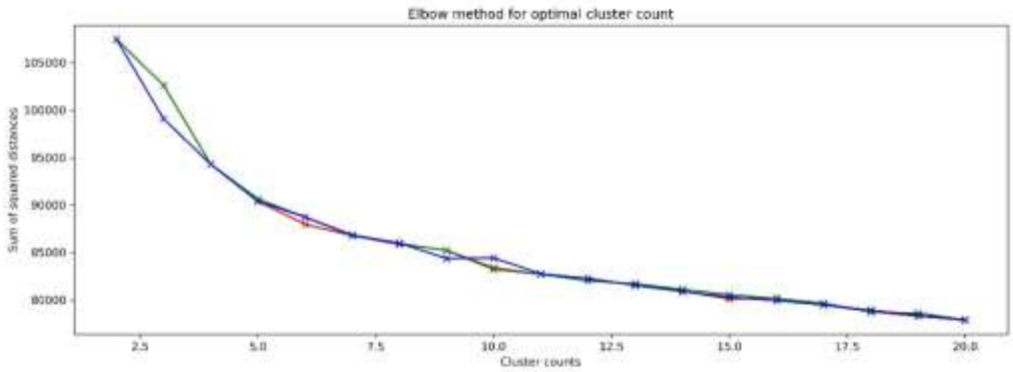

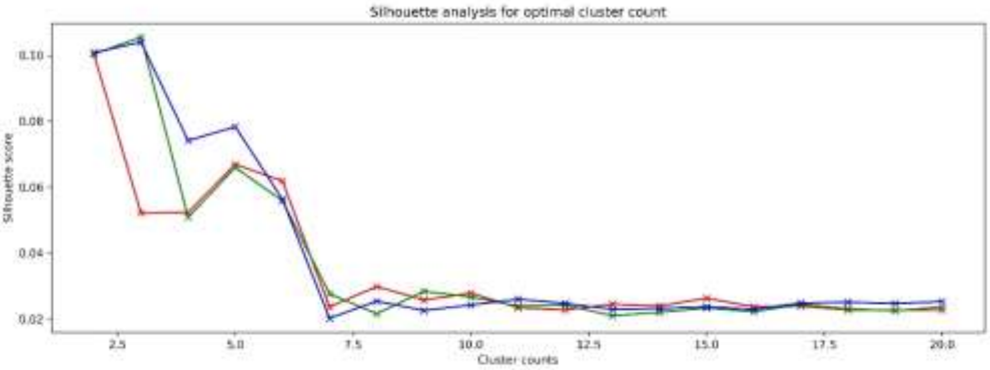

K-Means clustering:

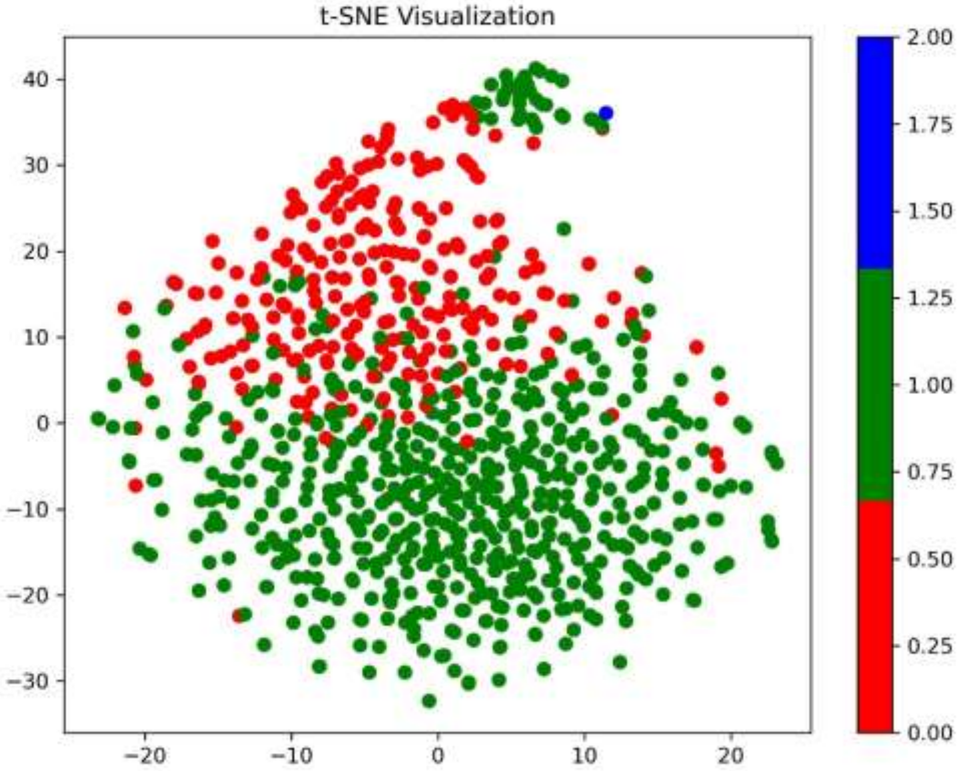

GMM Silhouette and BIC:

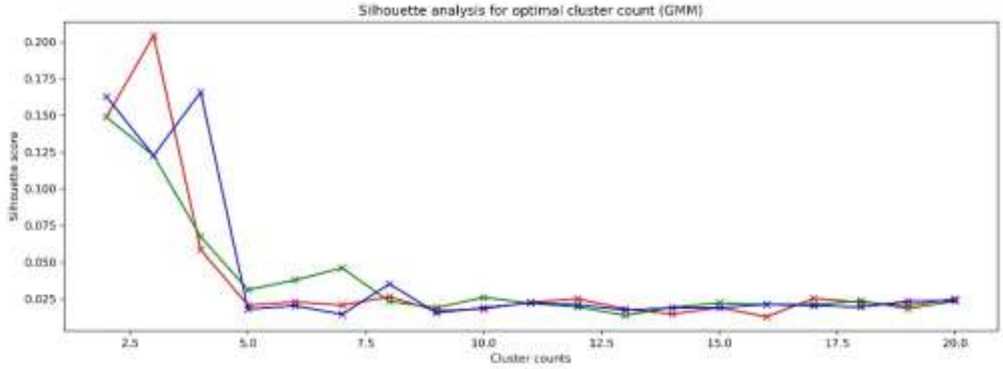

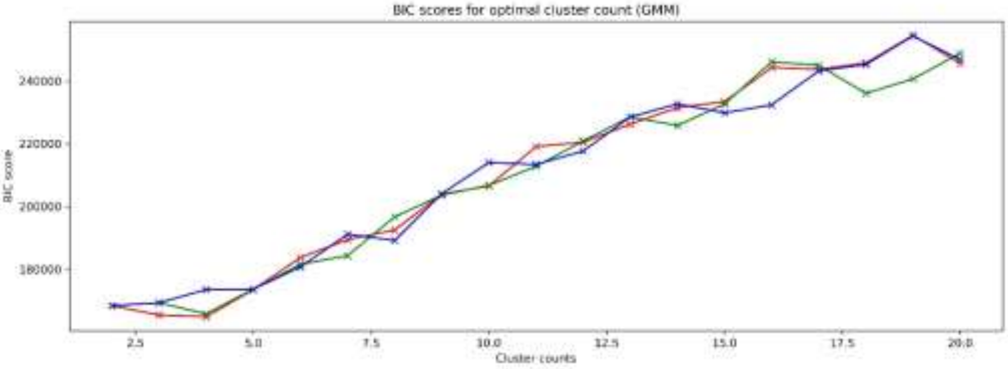

GMM Clustering:

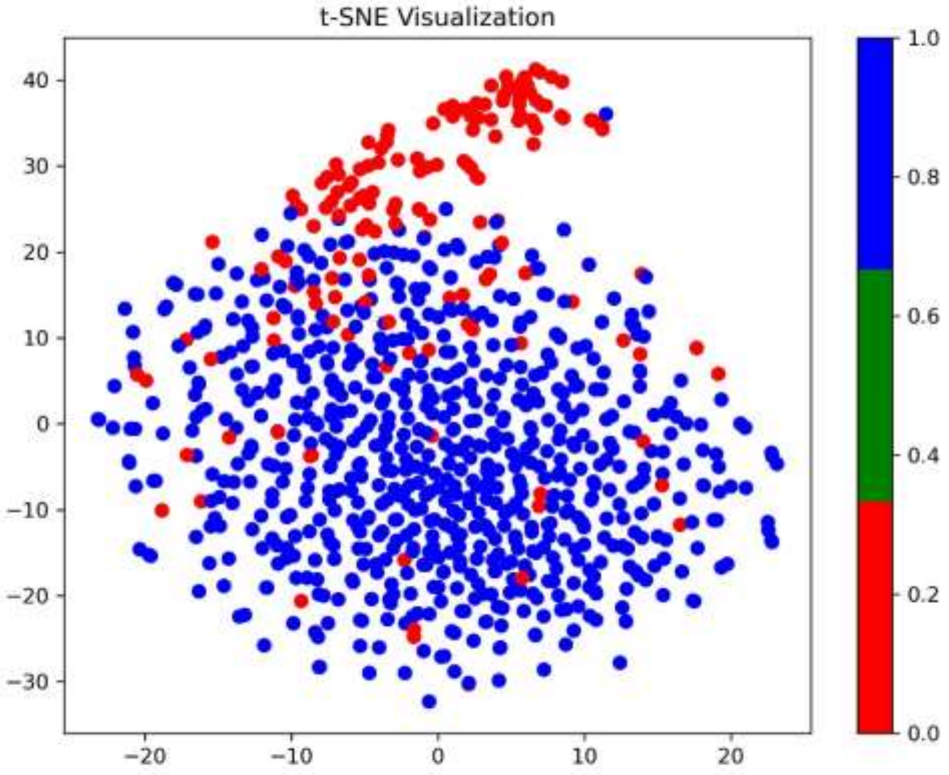

DBSCAN epsilon:

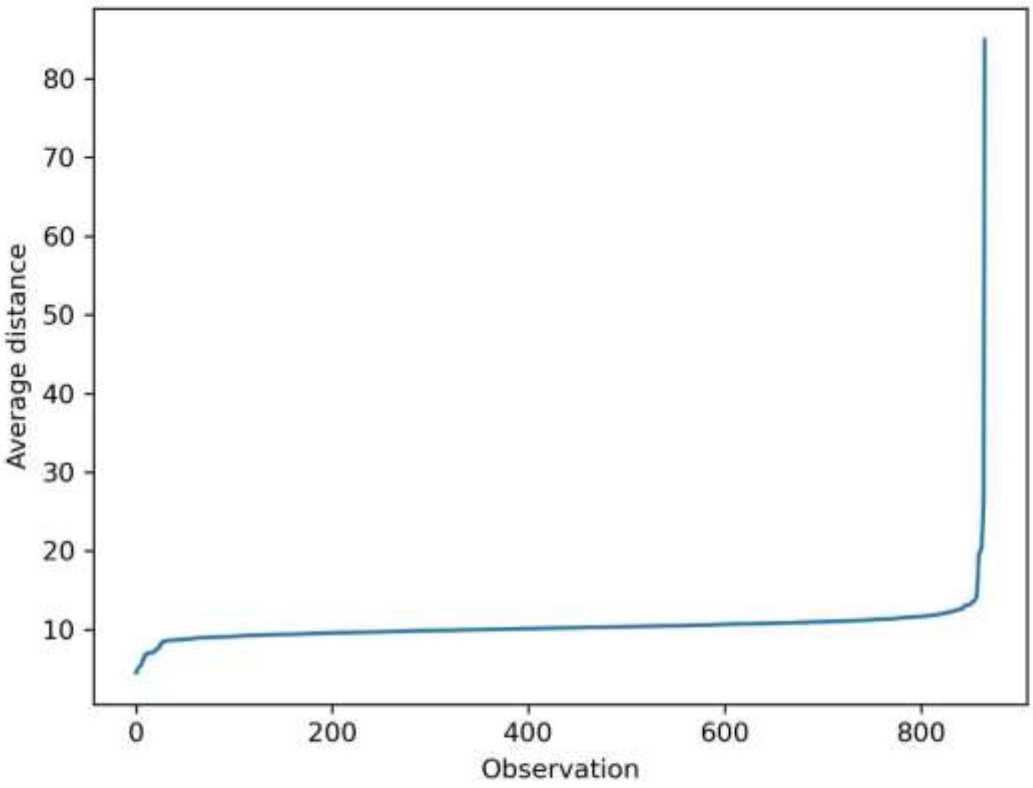

DBSCAN – best based on Davies Bouldin

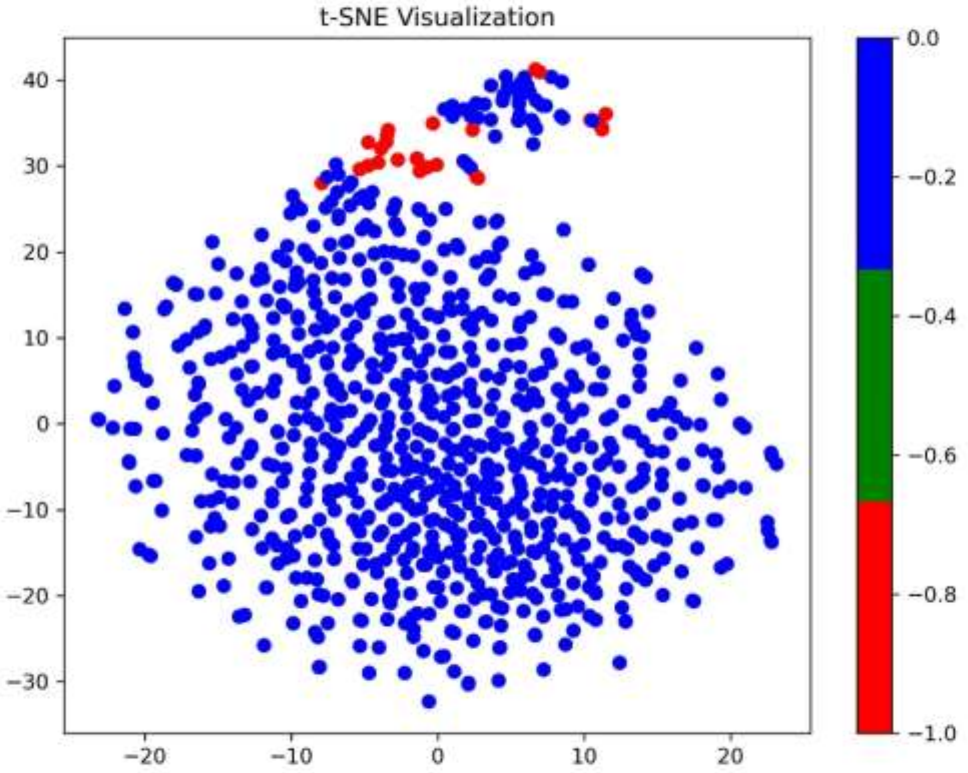

DBSCAN – Best based on Silhouette:

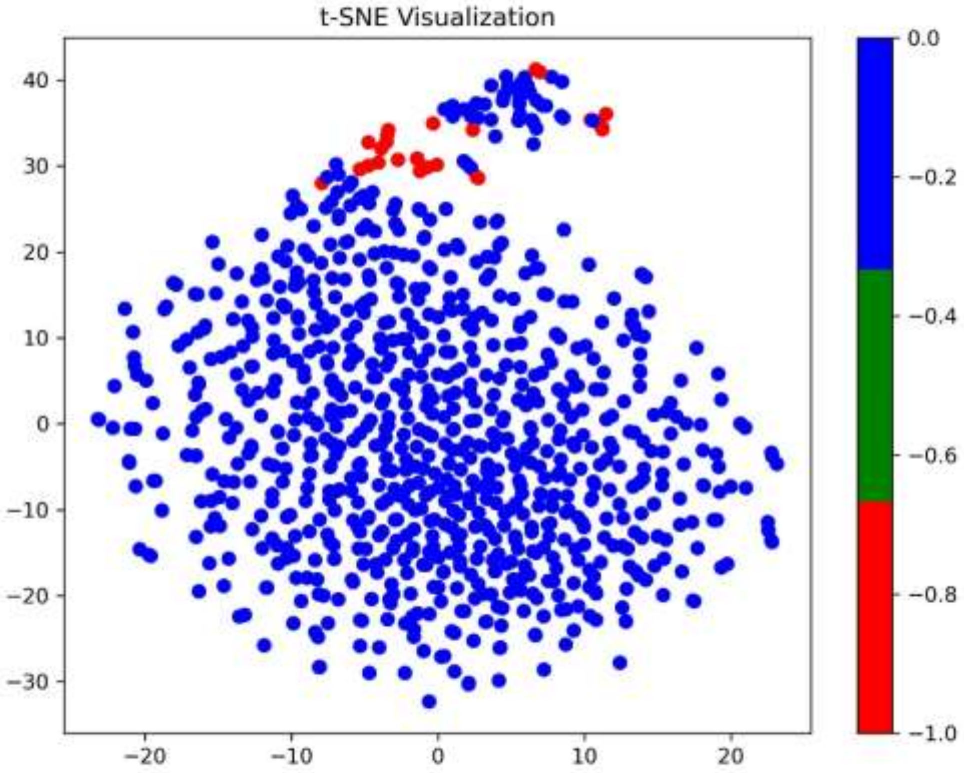

Best model – GMM with k = 2.

Right Digastric Muscle

Data visualization:

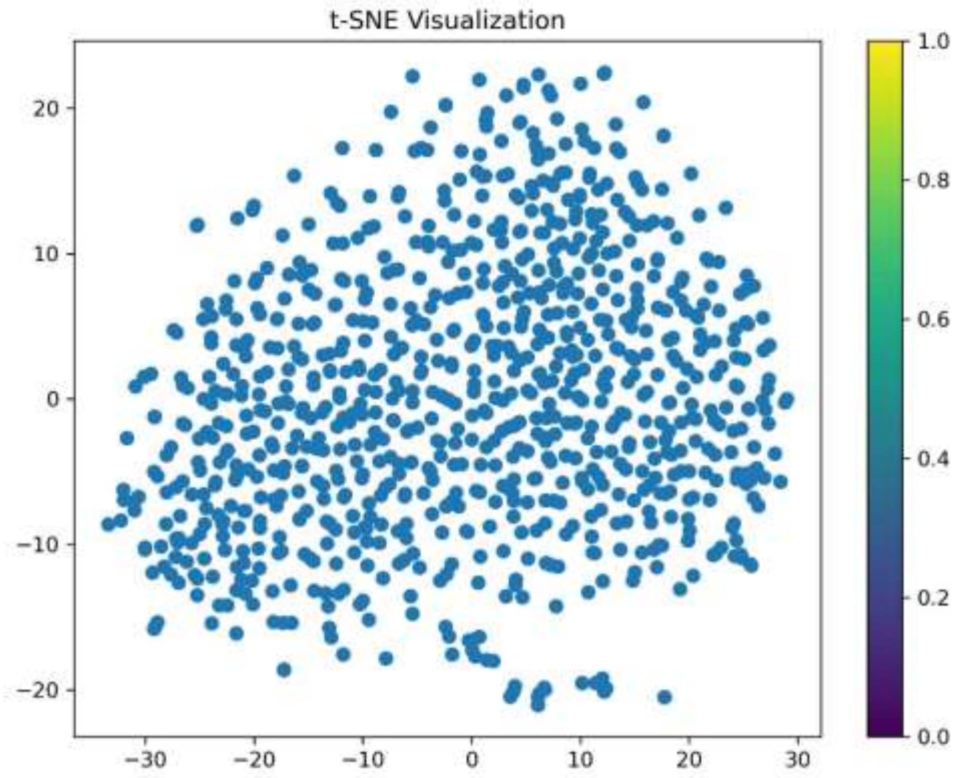

K-Means Elbow and Silhouette:

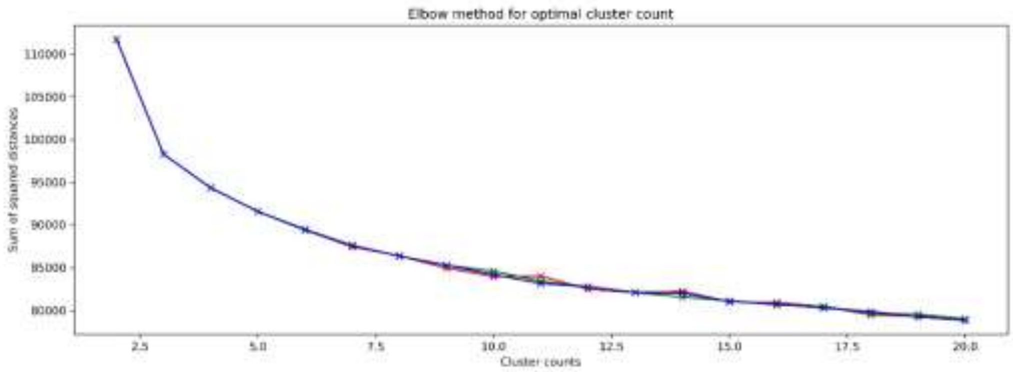

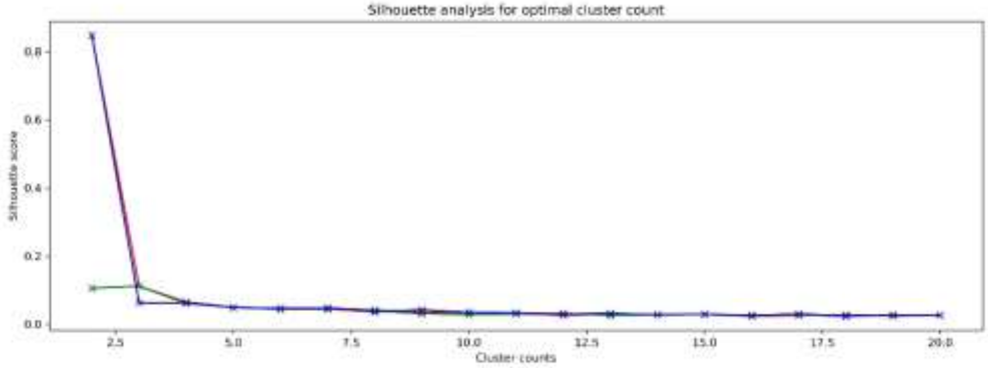

K-Means clustering:

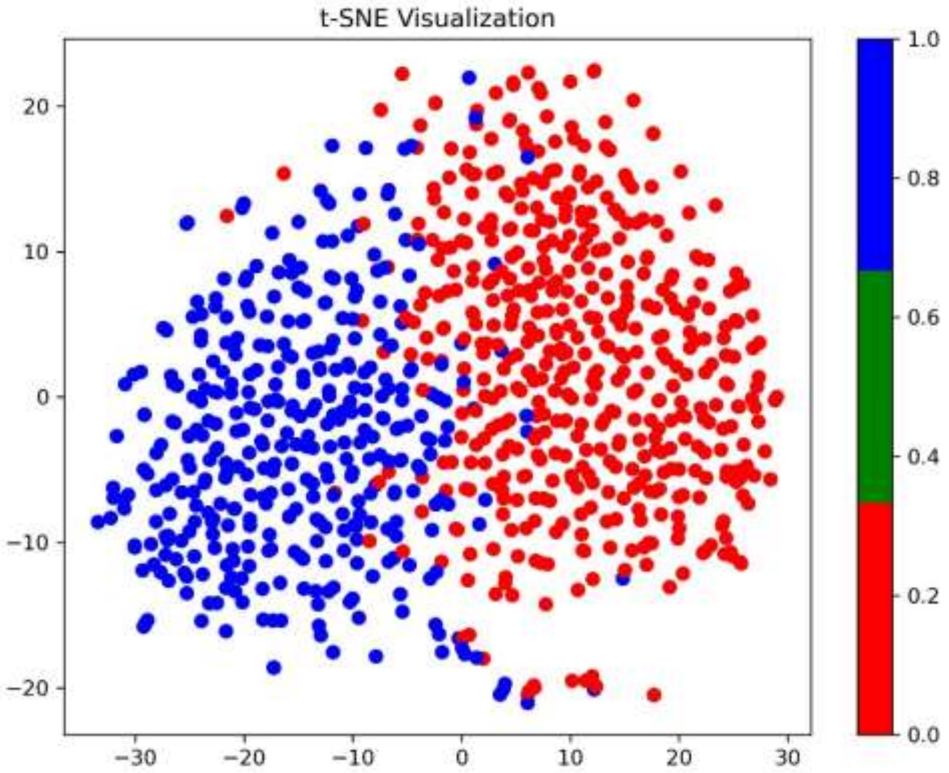

GMM Silhouette and BIC:

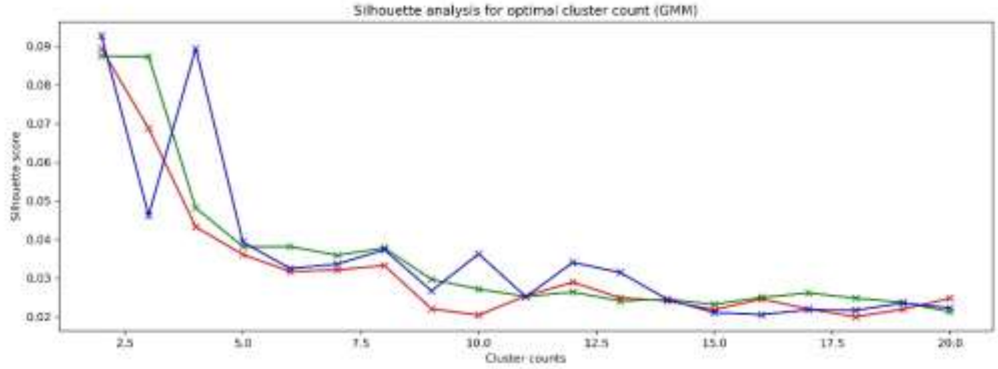

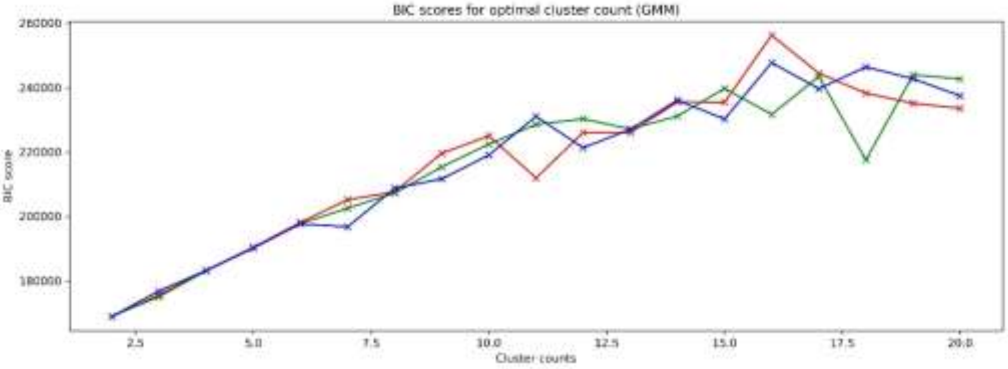

GMM Clustering:

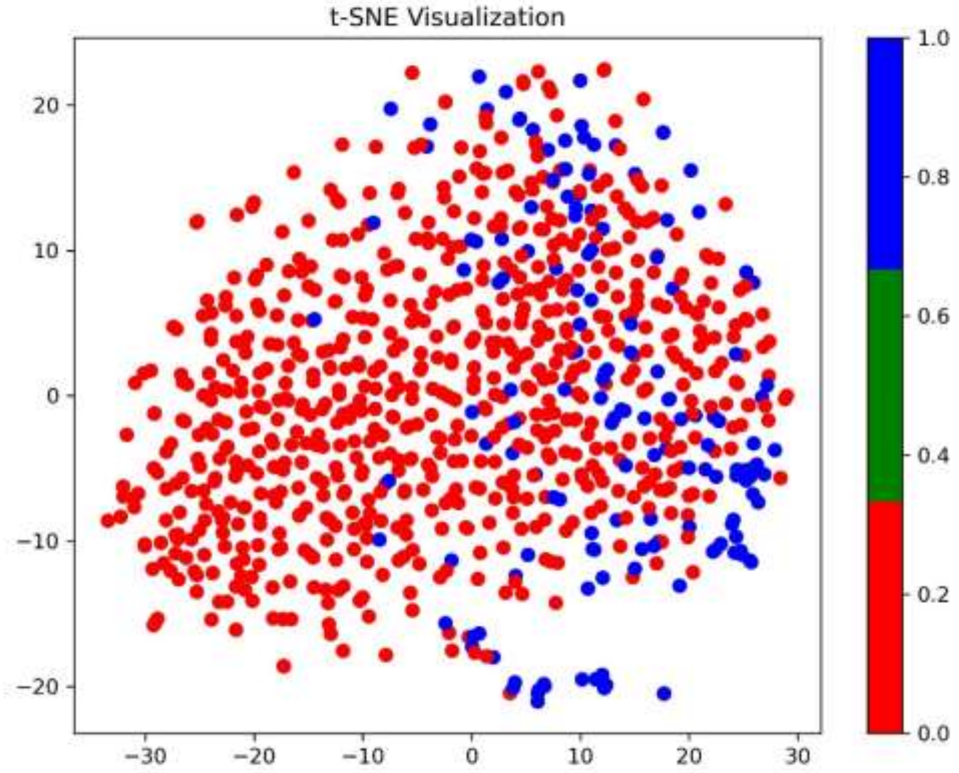

DBSCAN epsilon:

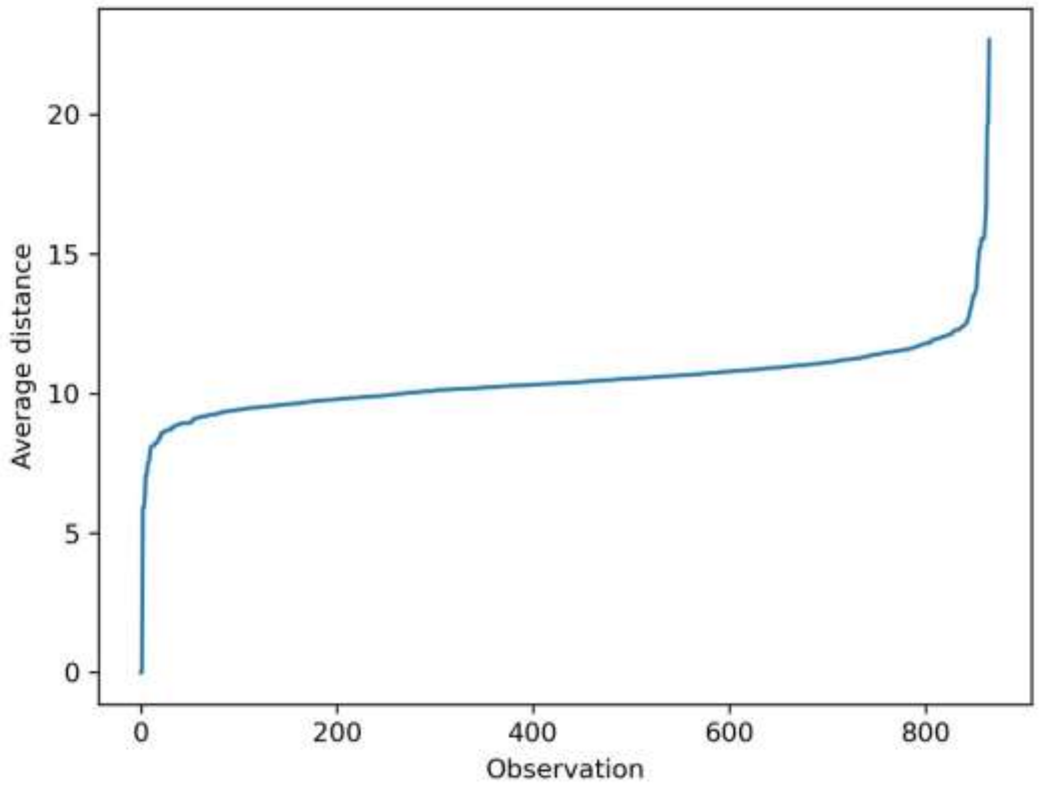

DBSCAN – best based on Davies Bouldin

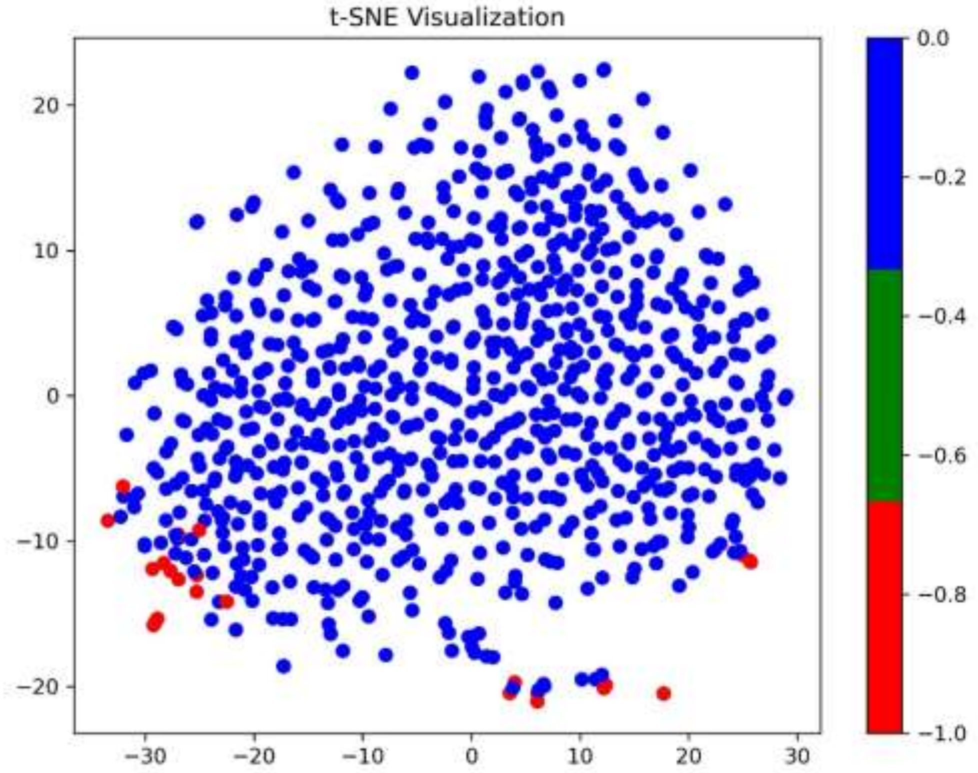

DBSCAN – Best based on Silhouette:

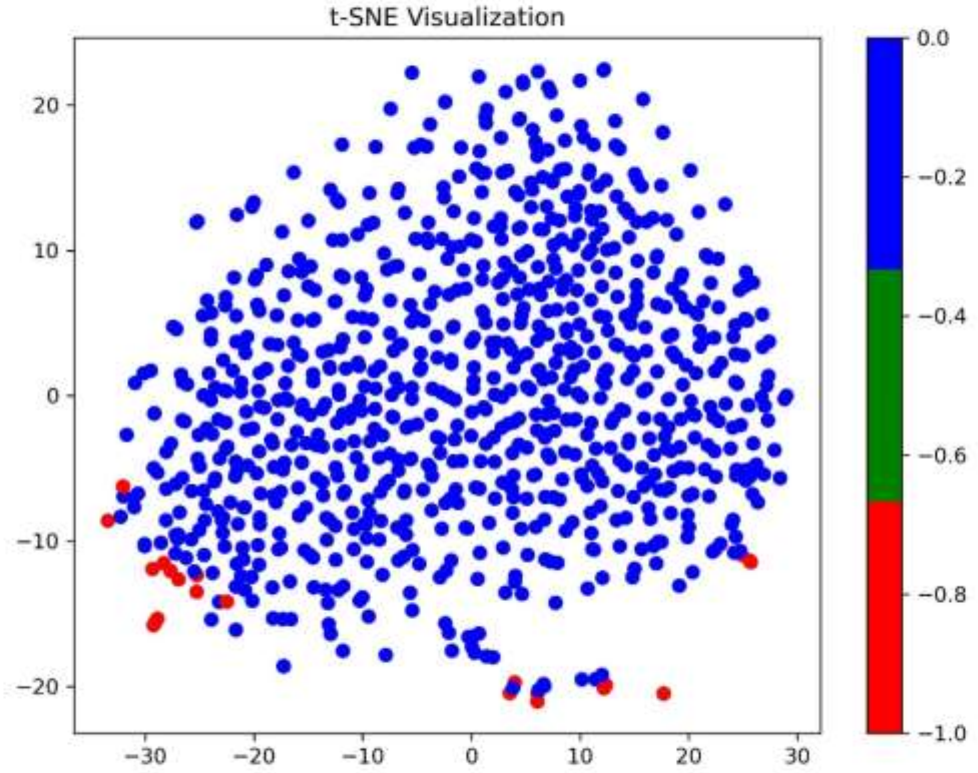

Best model – K-Means with k = 2.

Left Digastric Muscle

Data visualization:

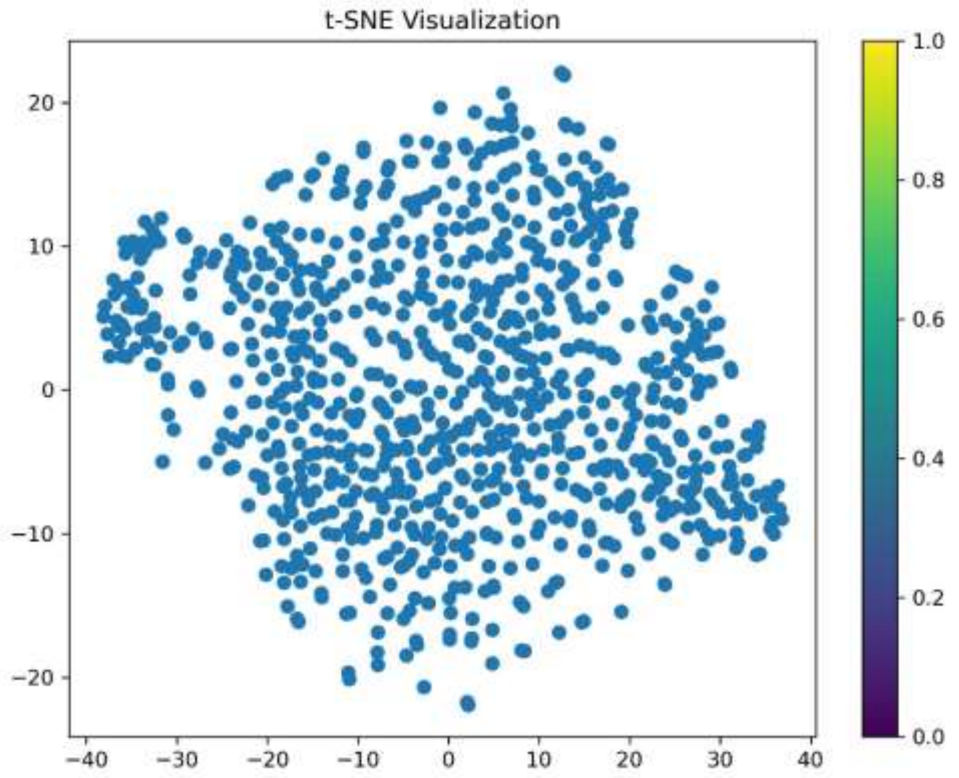

K-Means Elbow and Silhouette:

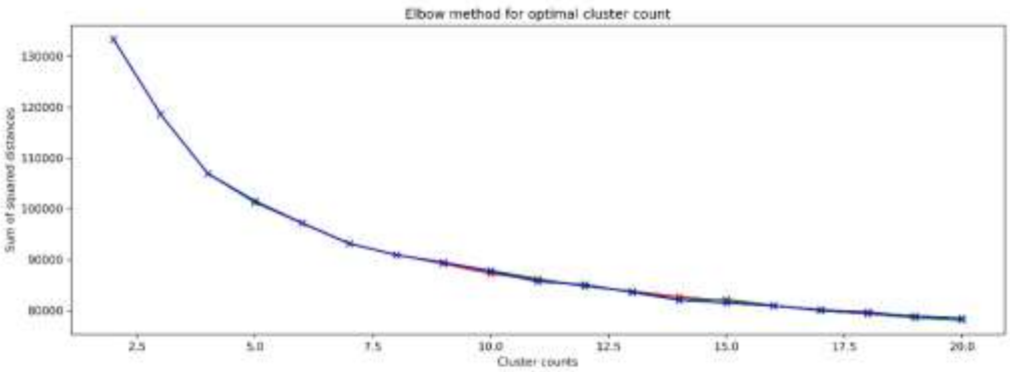

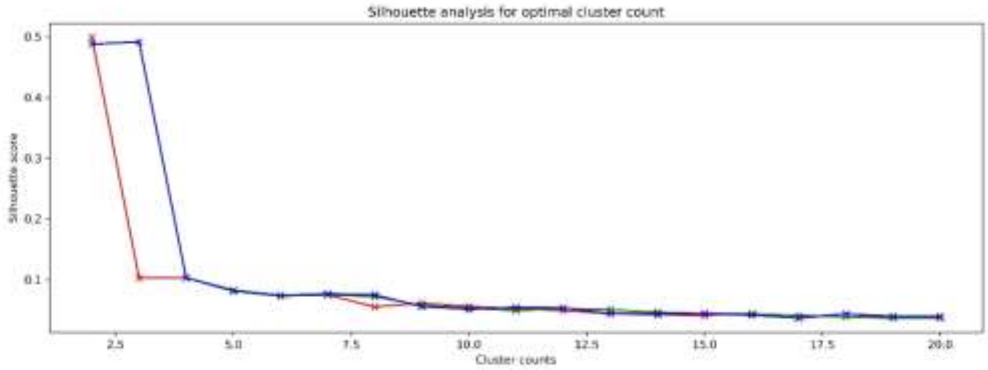

K-Means clustering:

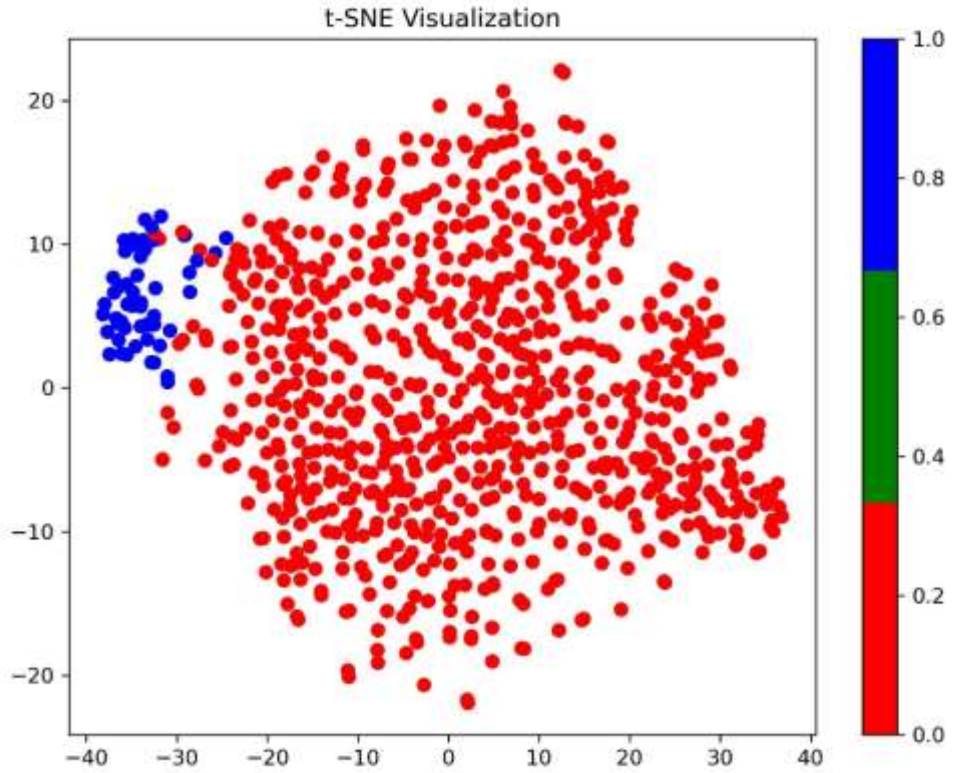

GMM Silhouette and BIC:

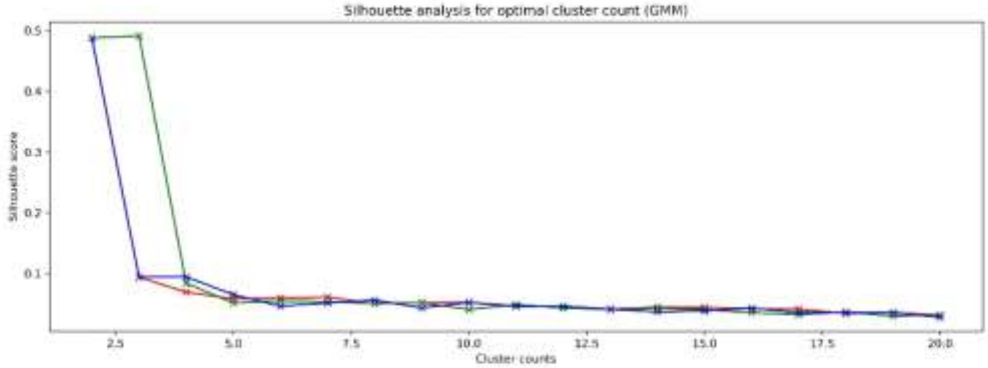

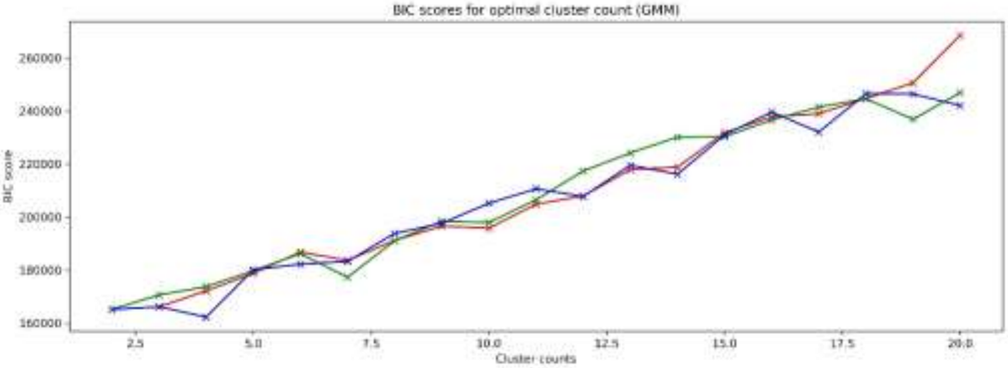

GMM Clustering:

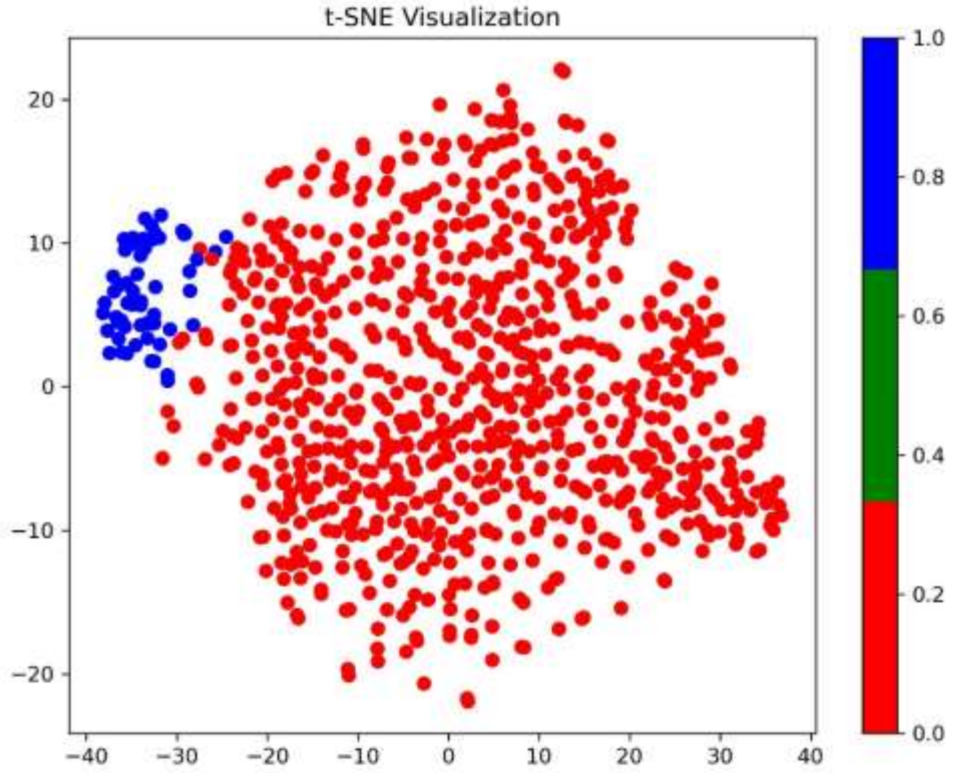

DBSCAN epsilon:

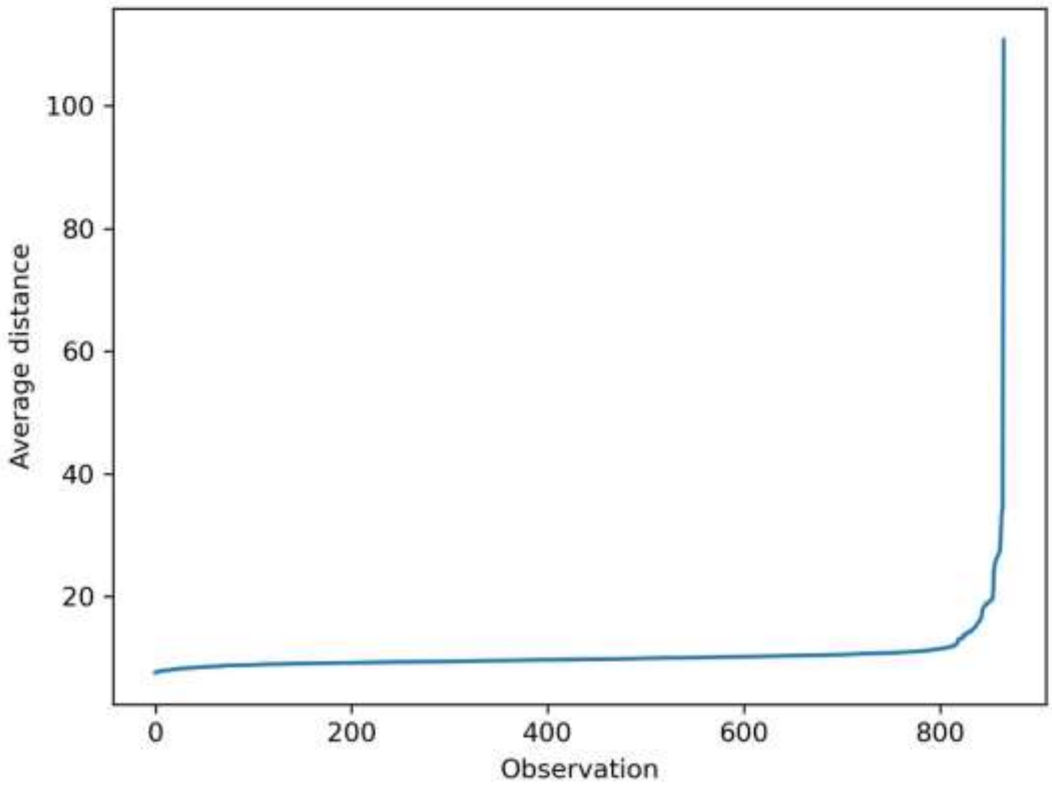

DBSCAN – best based on Davies Bouldin

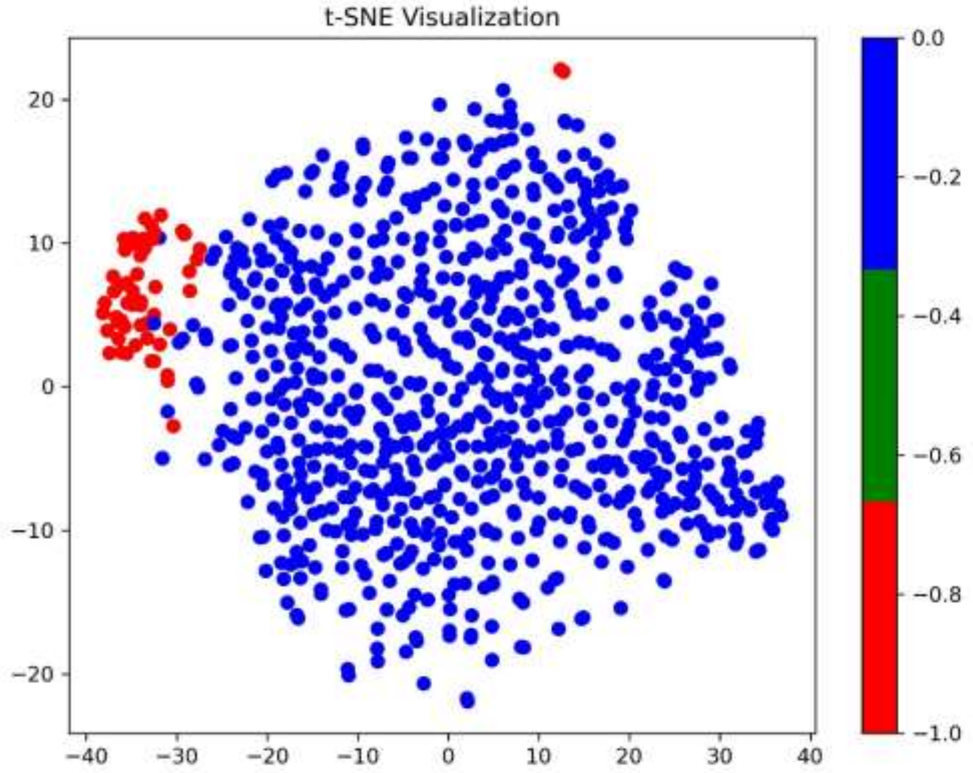

DBSCAN – Best based on Silhouette:

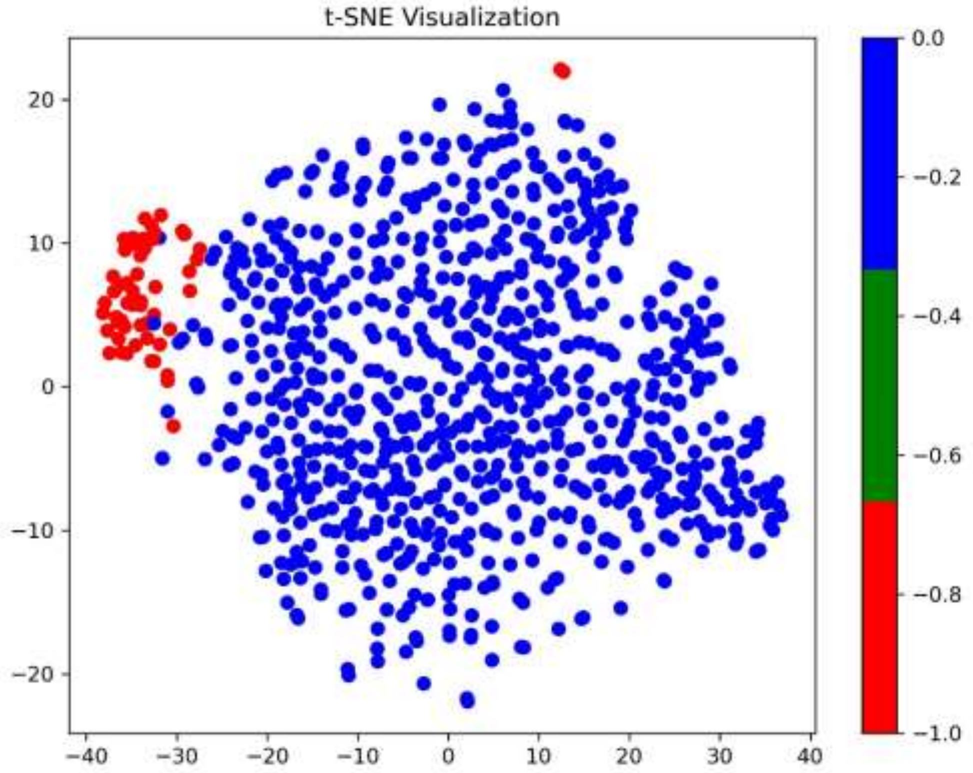

Best model – DBSCAN with Epsilon = 13.8, Min Points = 101.

Maximum Anterior Protrusion

Right Temporalis

Data visualization:

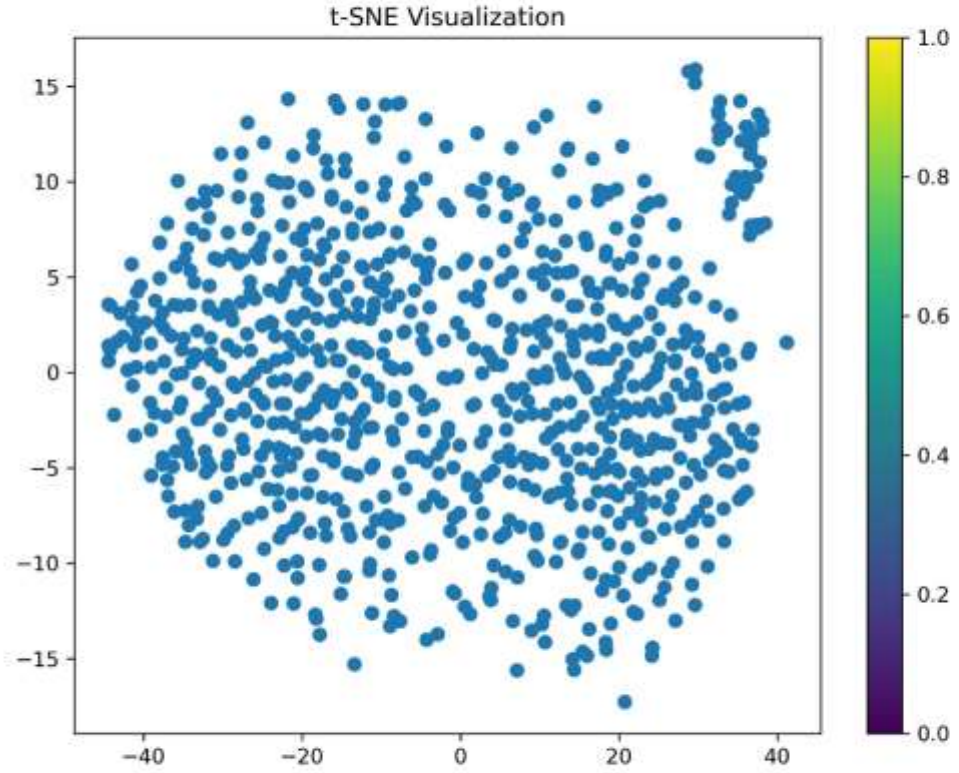

K-Means Elbow and Silhouette:

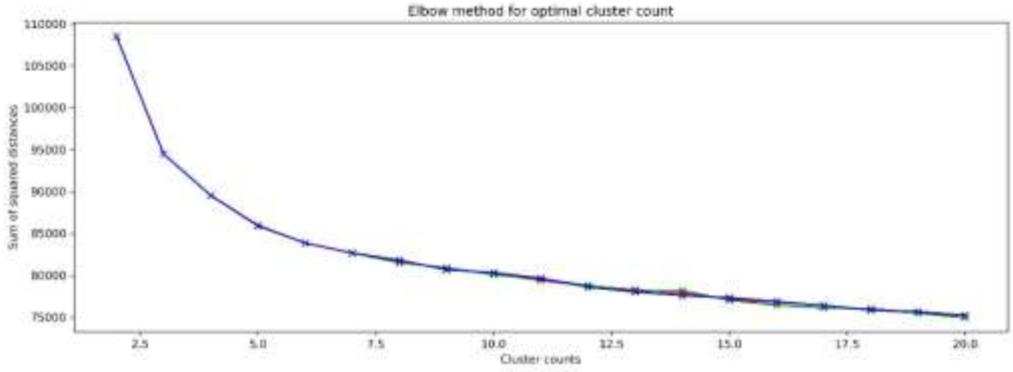

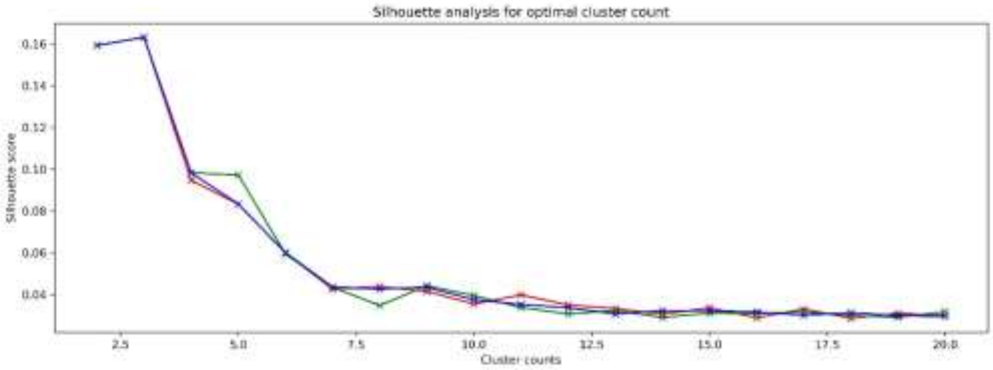

K-Means clustering:

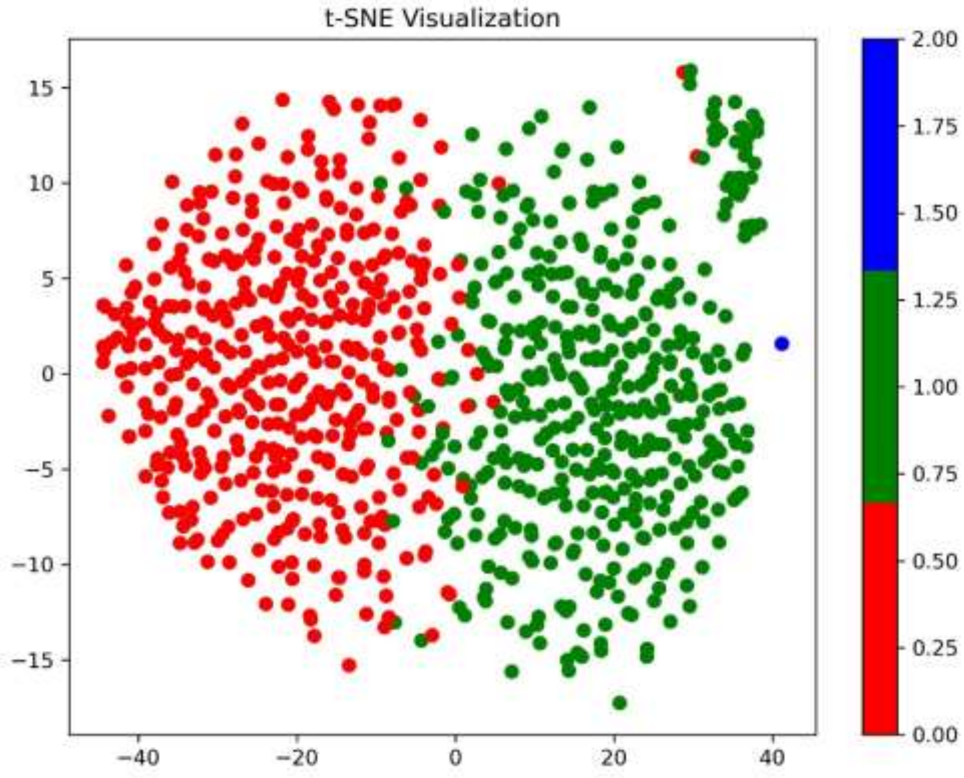

GMM Silhouette and BIC:

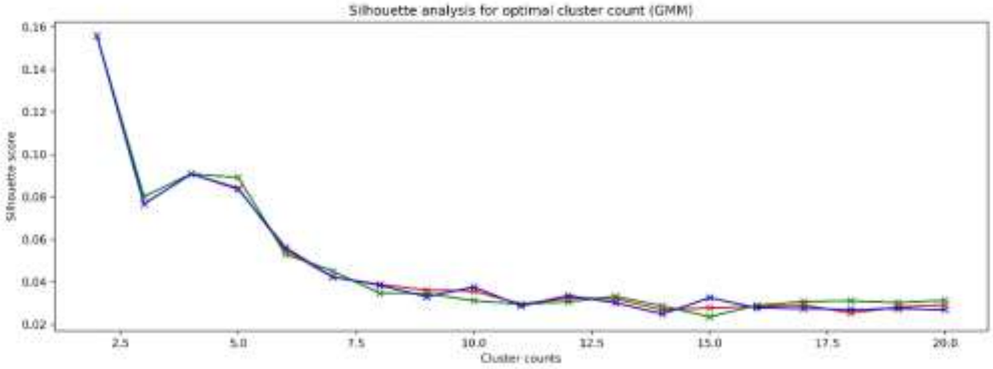

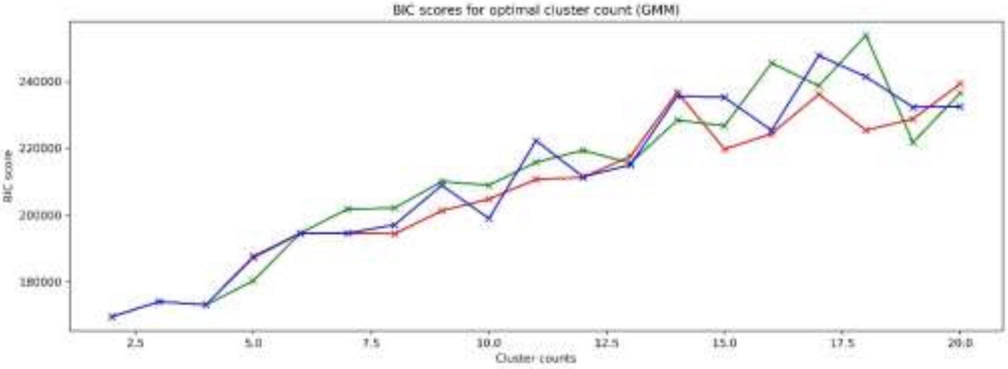

GMM Clustering:

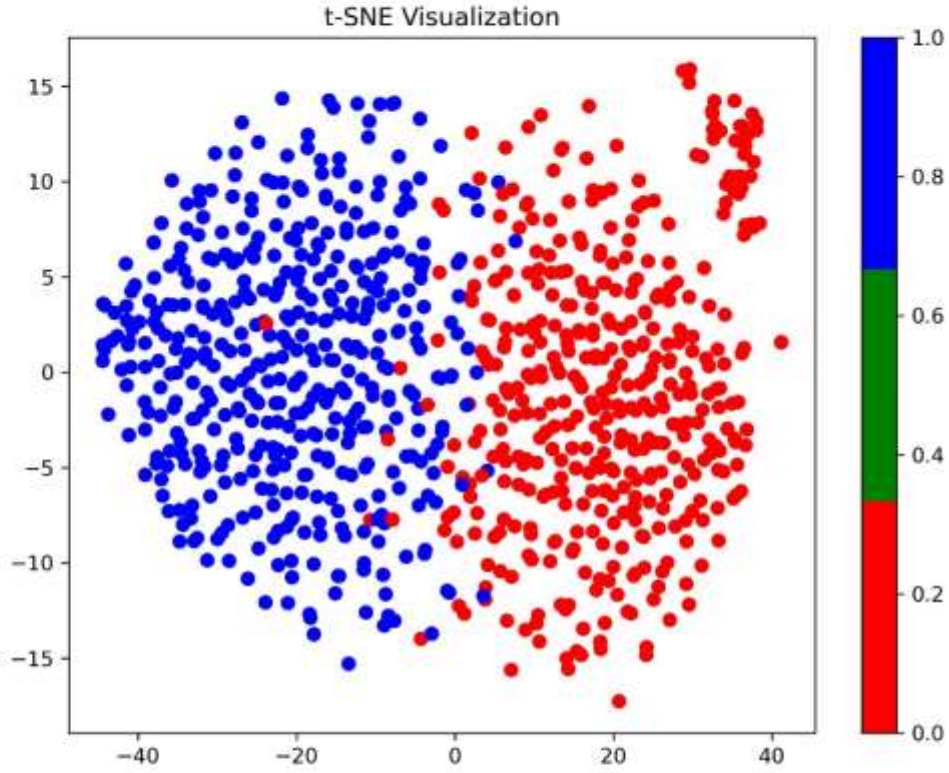

DBSCAN epsilon:

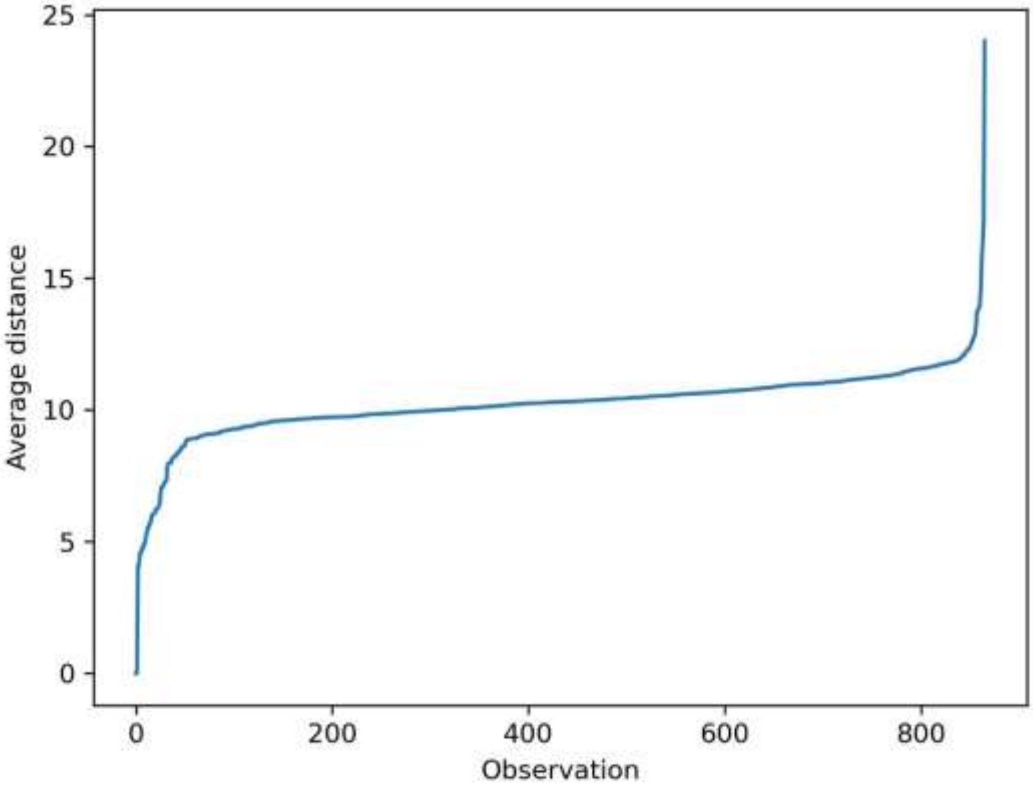

DBSCAN – best based on Davies Bouldin

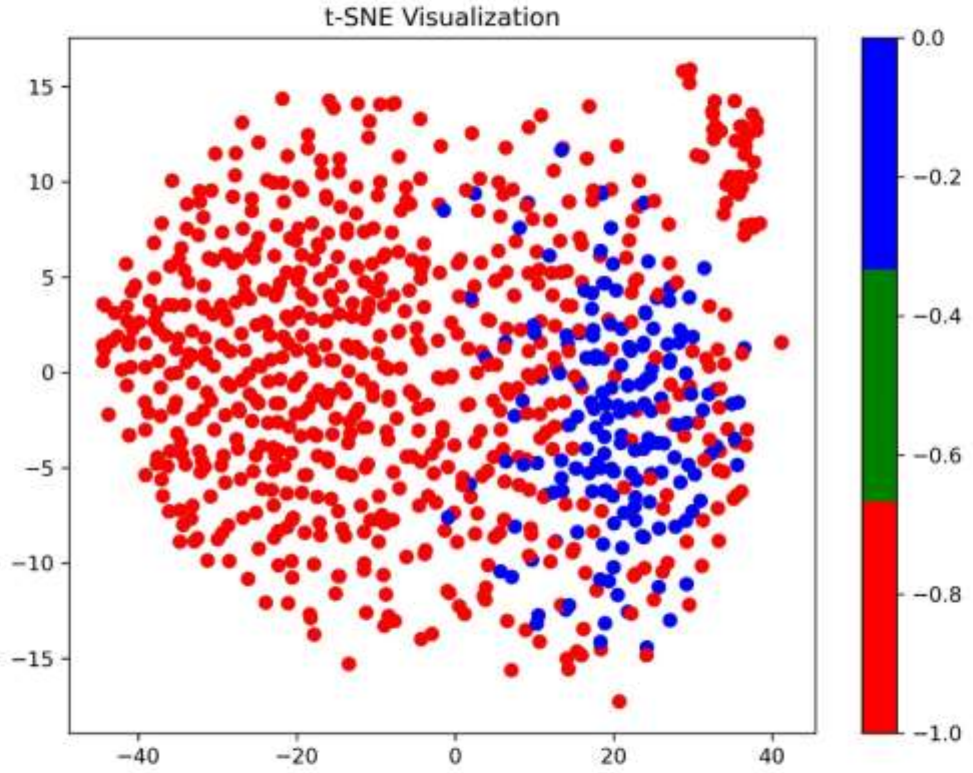

DBSCAN – Best based on Silhouette:

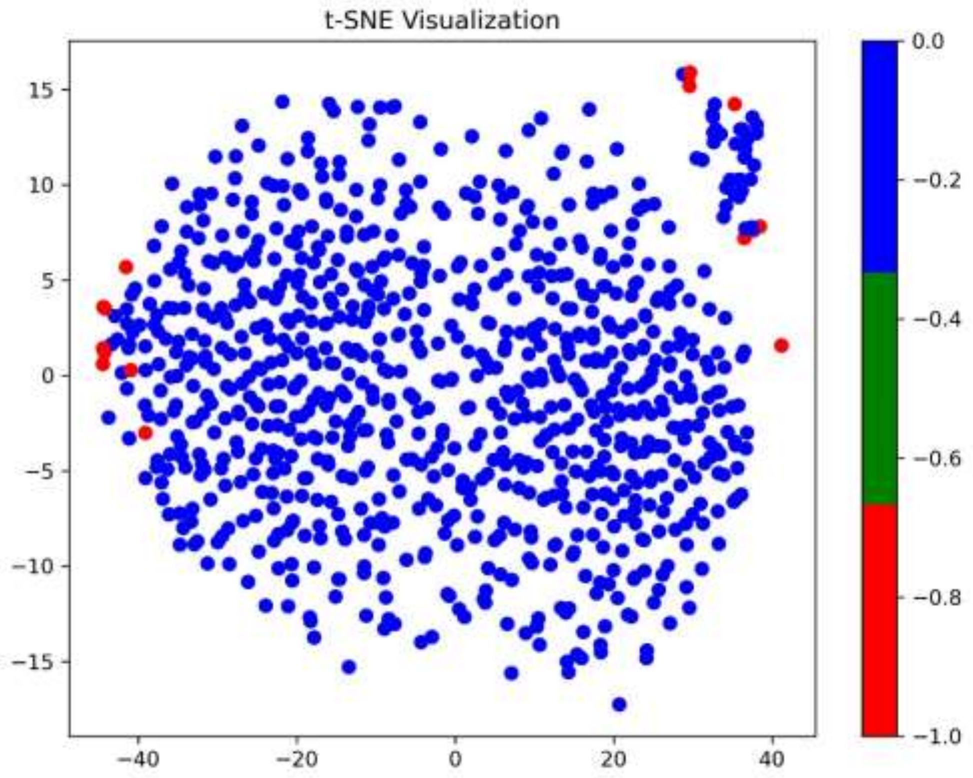

Best model – GMM with k = 2

Left Temporalis

Data visualization:

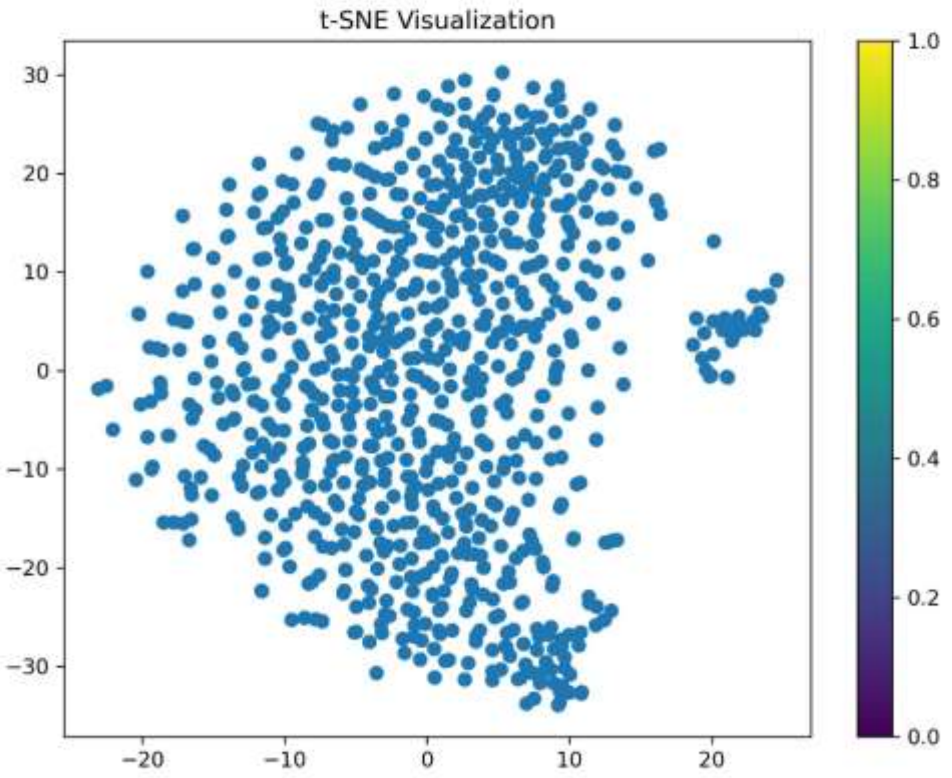

K-Means Elbow and Silhouette:

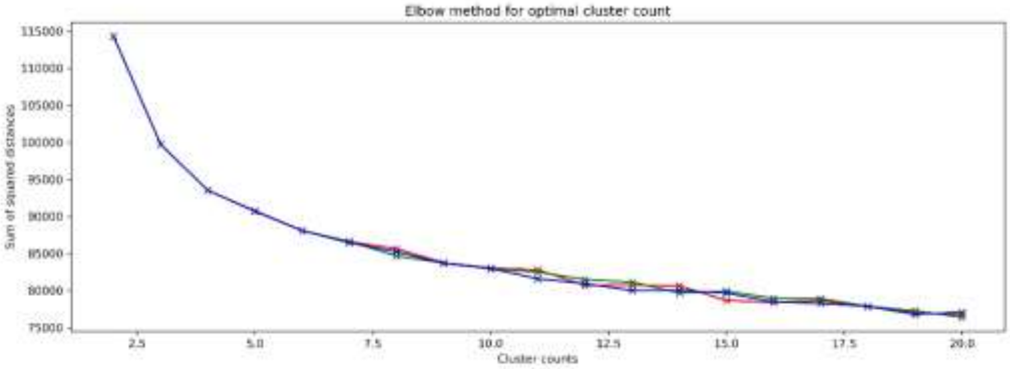

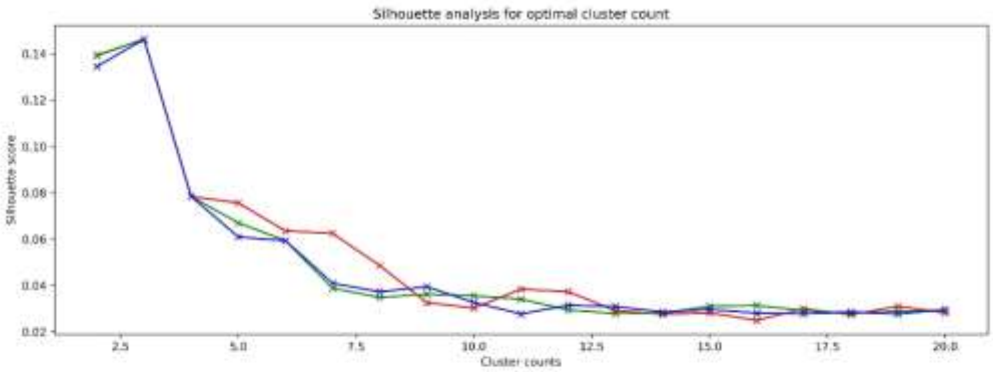

K-Means clustering:

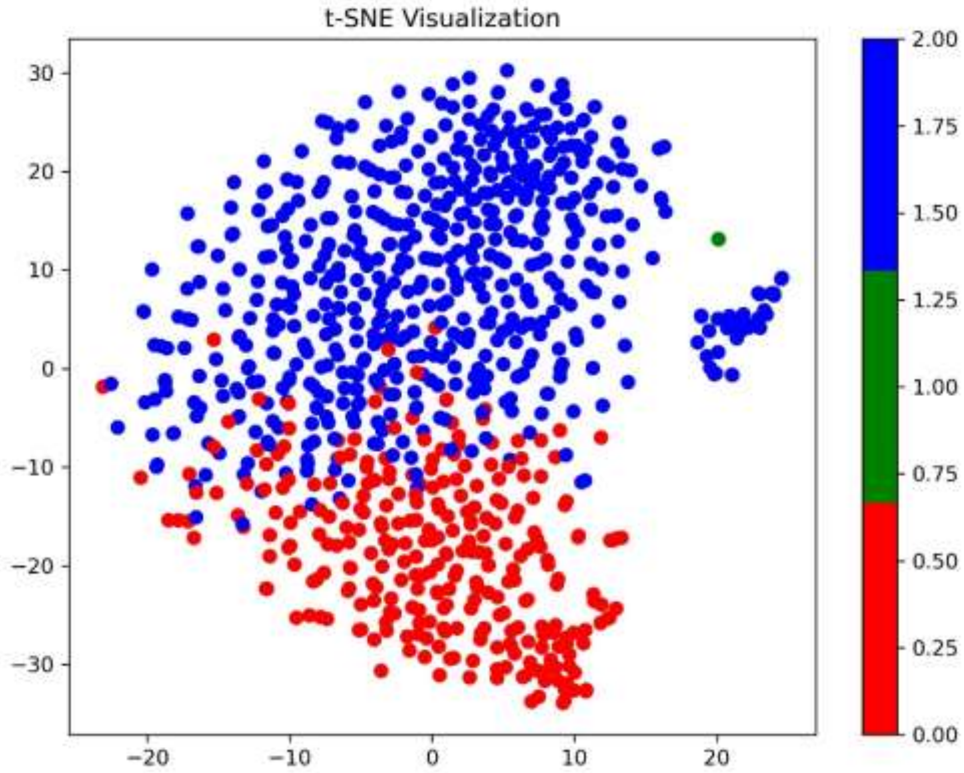

GMM Silhouette and BIC:

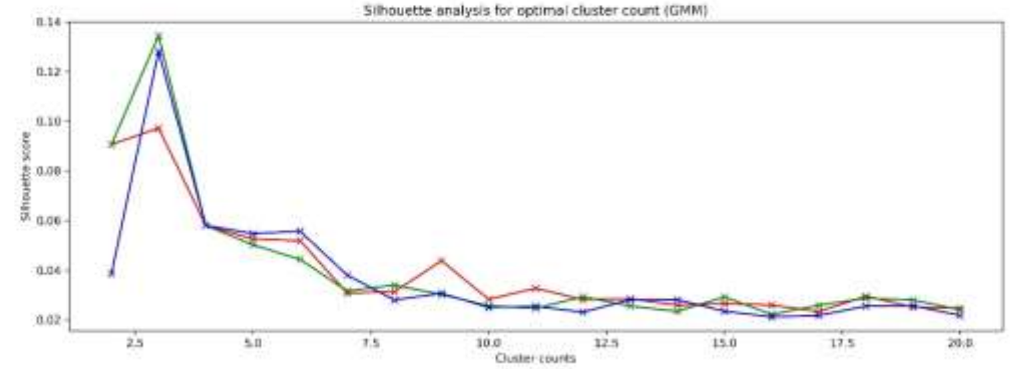

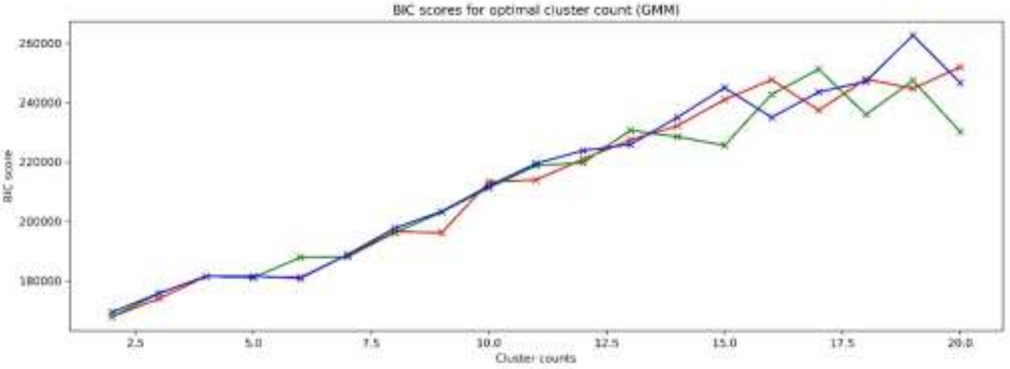

GMM Clustering:

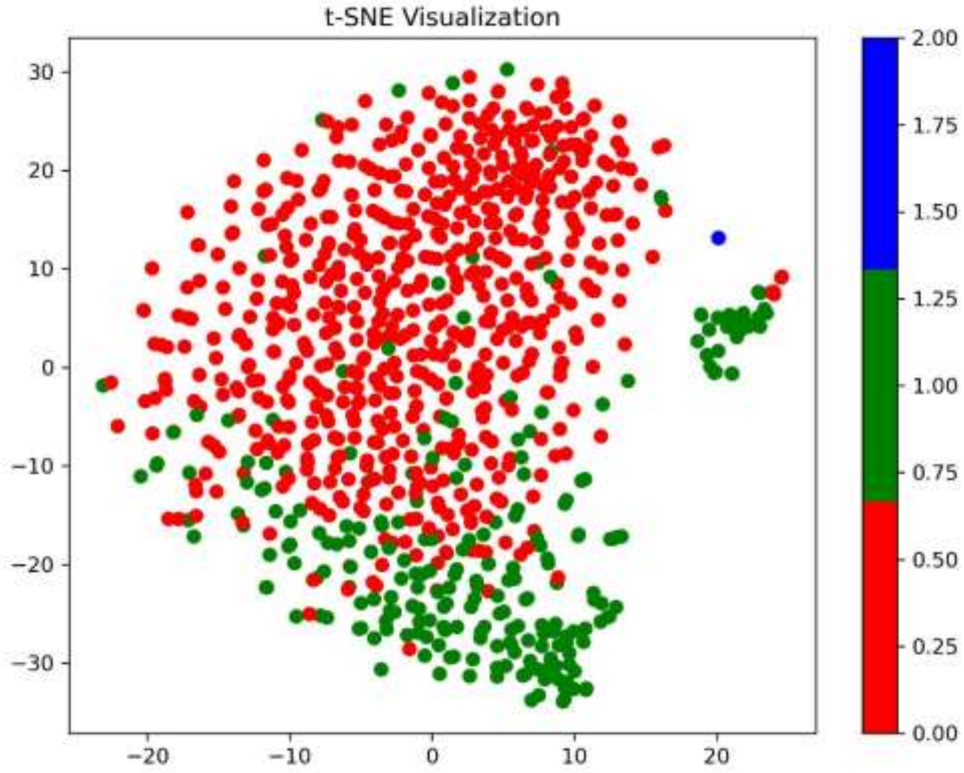

DBSCAN epsilon:

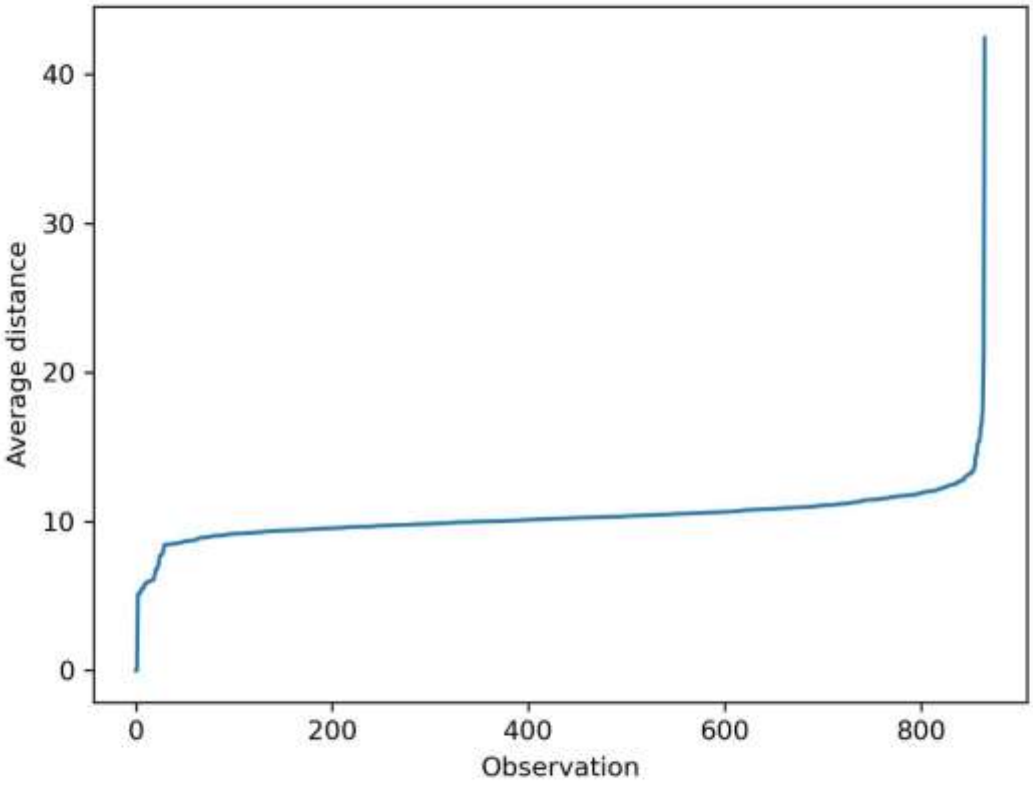

DBSCAN – best based on Davies Bouldin

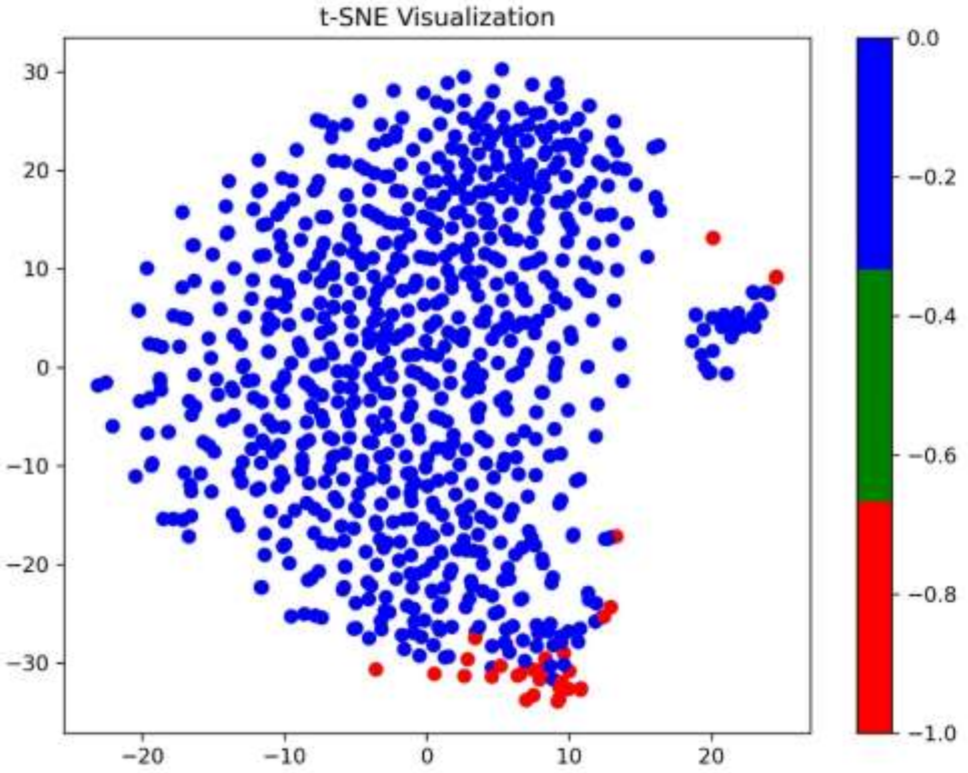

DBSCAN – Best based on Silhouette:

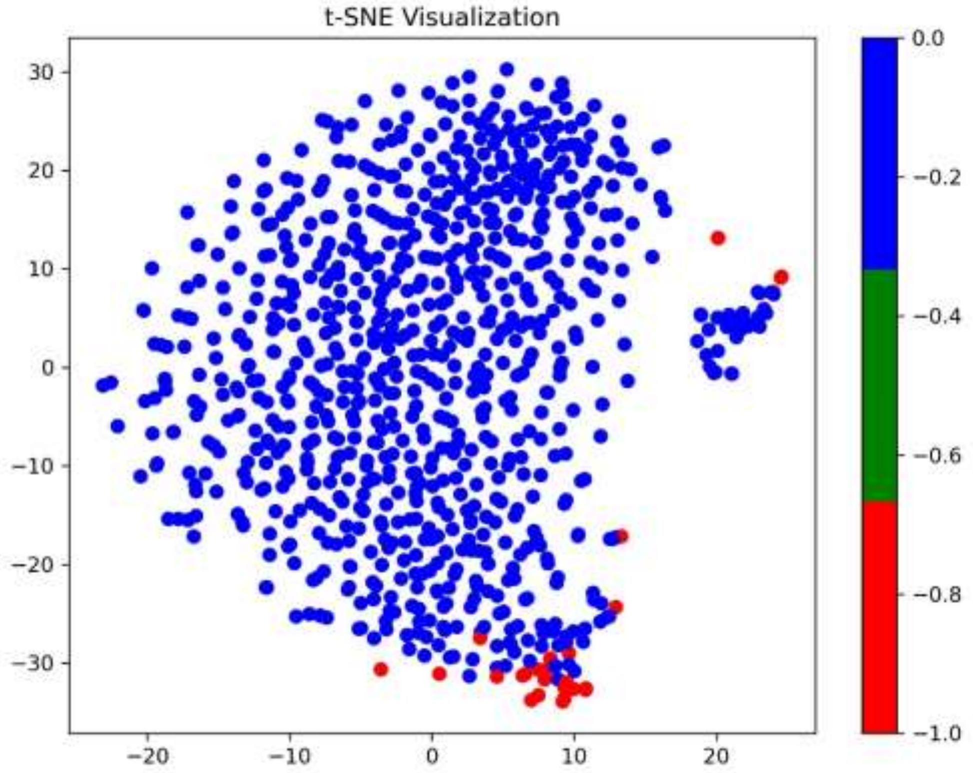

Best model – DBSCAN with Epsilon = 13.8, Min Points = 61.

Right Masseter Muscle

Data visualization:

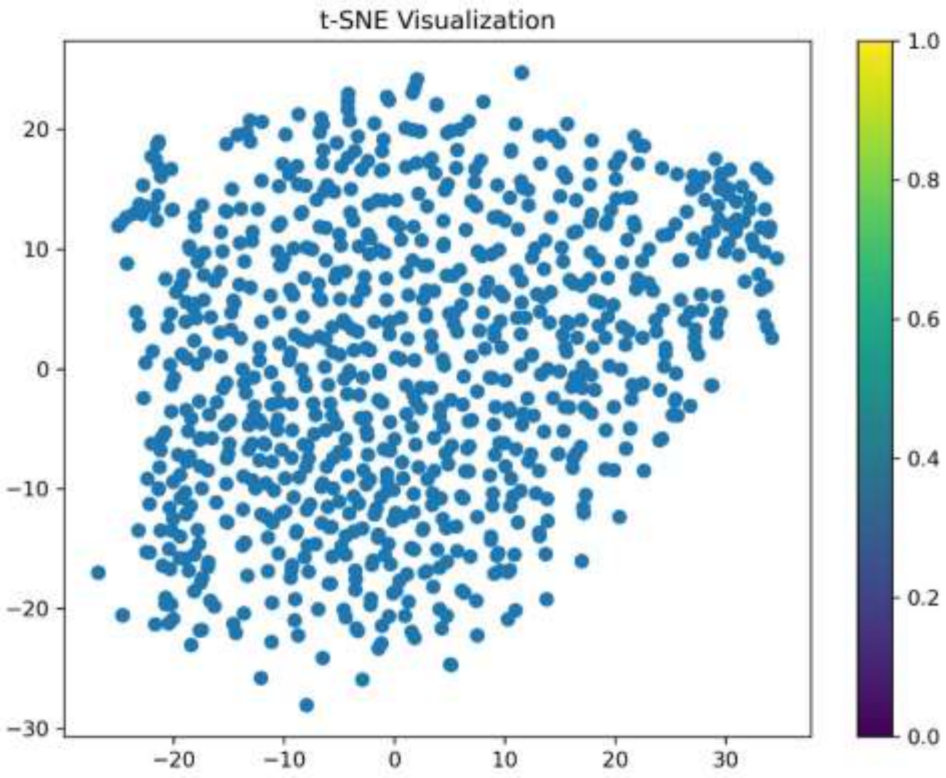

K-Means Elbow and Silhouette:

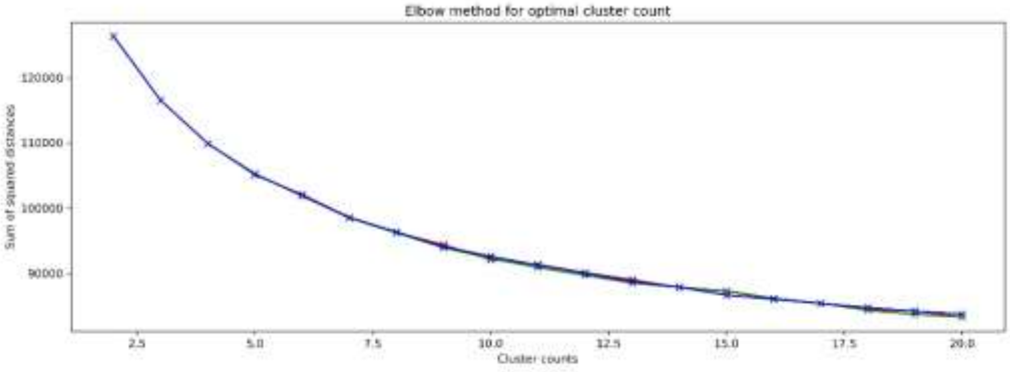

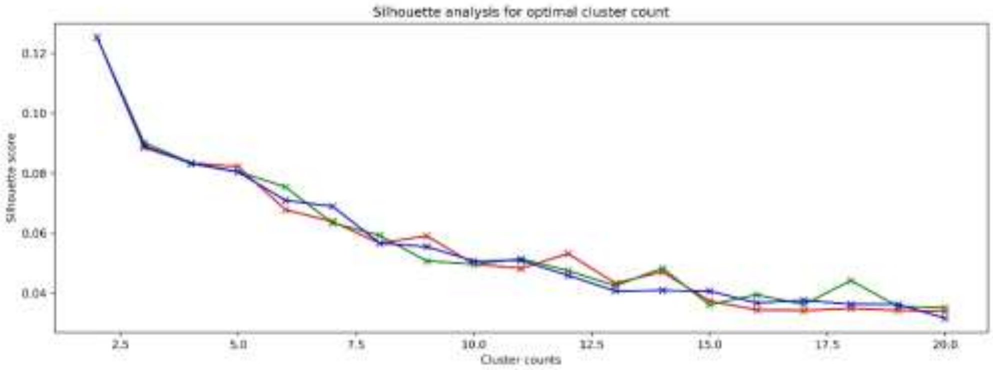

K-Means clustering:

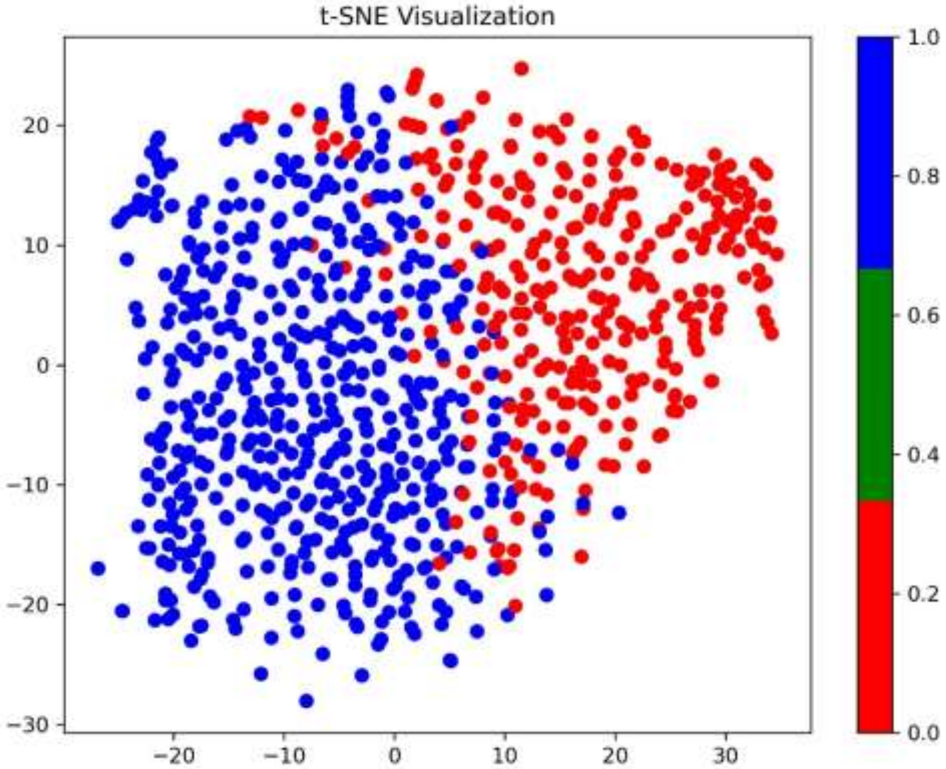

GMM Silhouette and BIC:

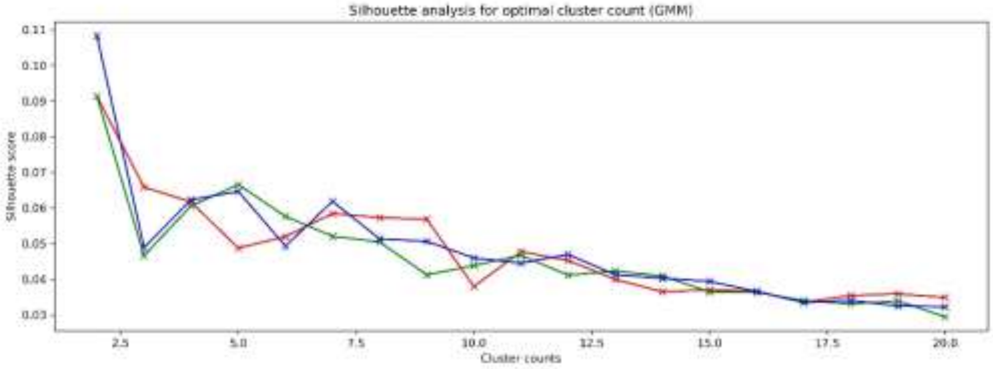

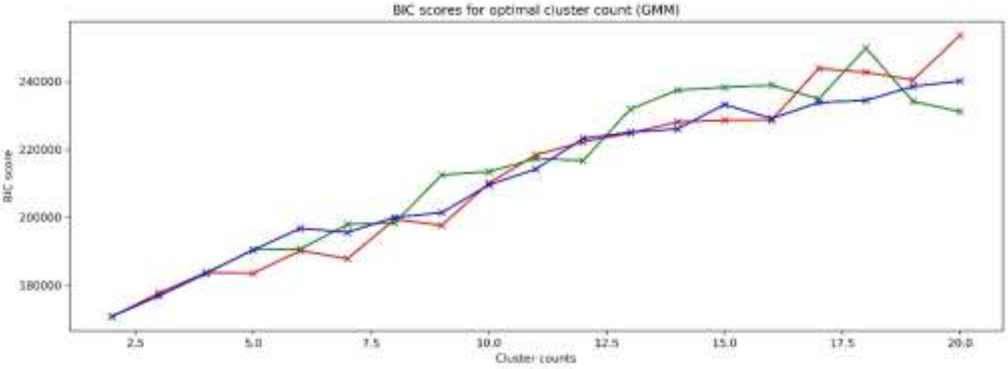

GMM Clustering:

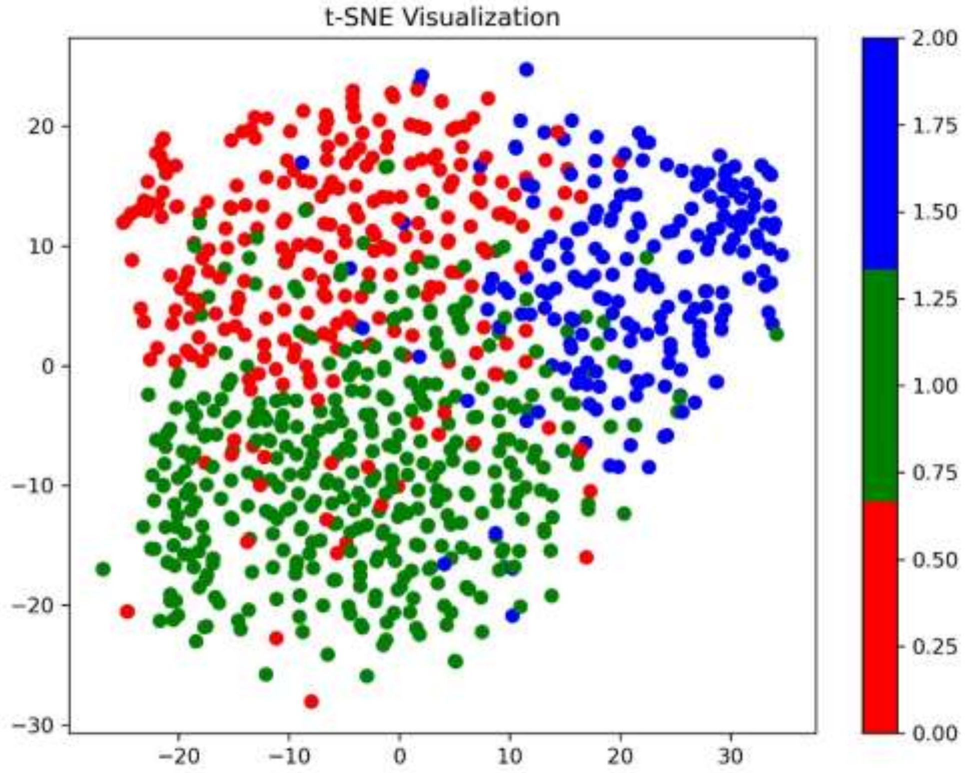

DBSCAN epsilon:

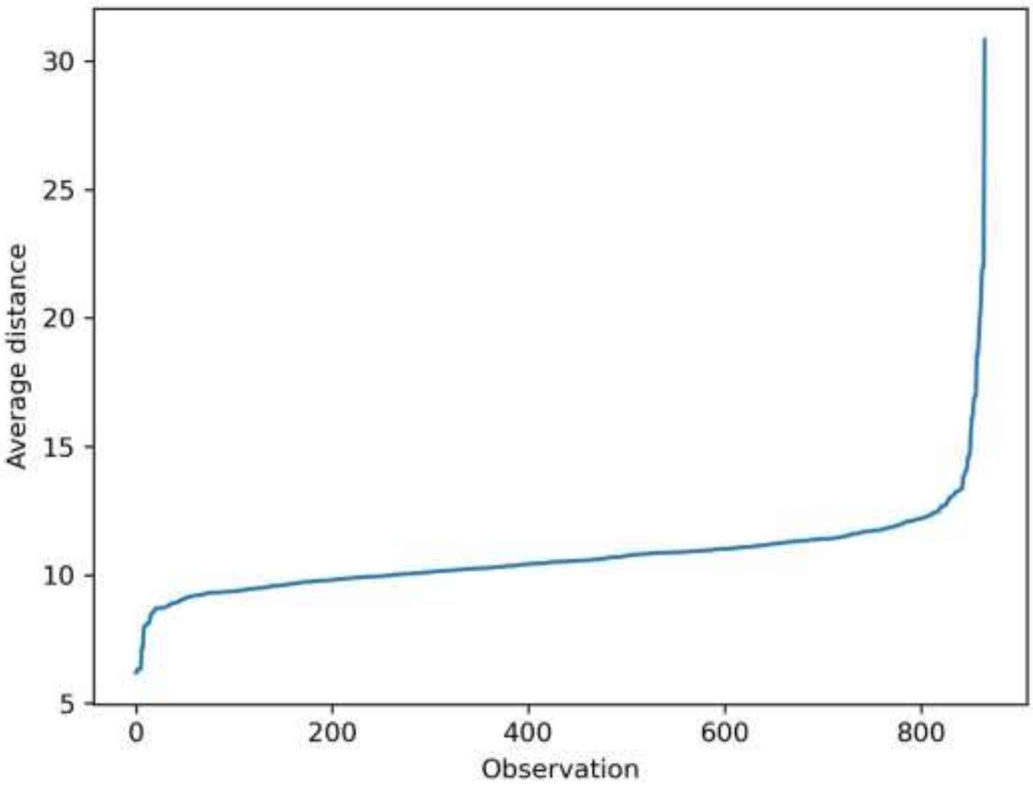

DBSCAN – best based on Davies Bouldin

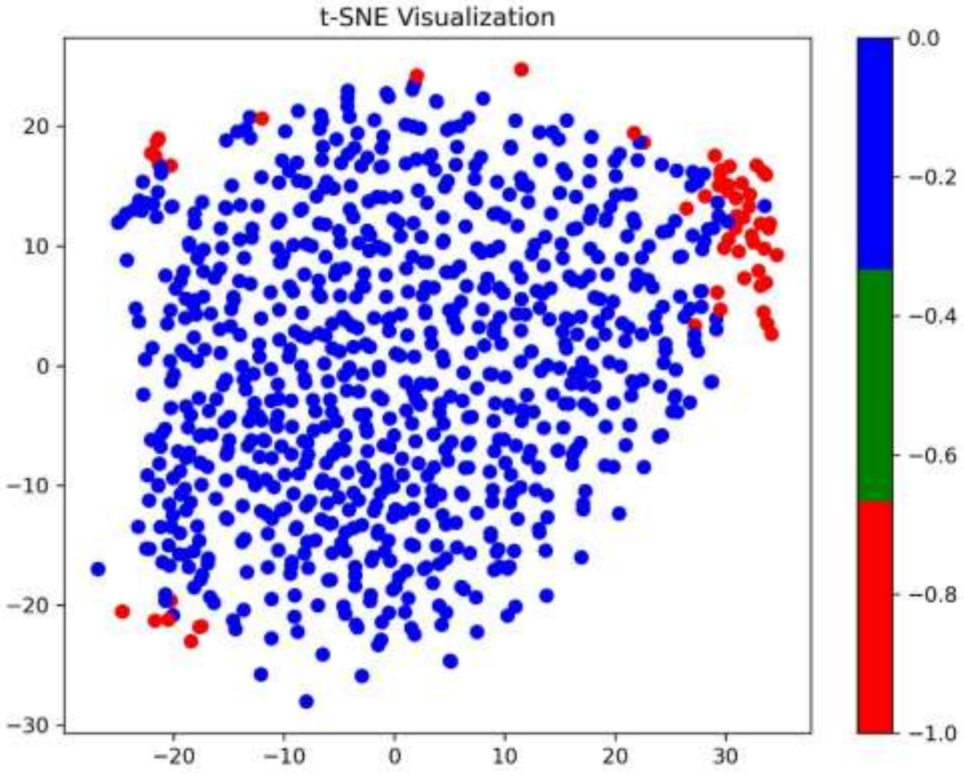

DBSCAN – Best based on Silhouette

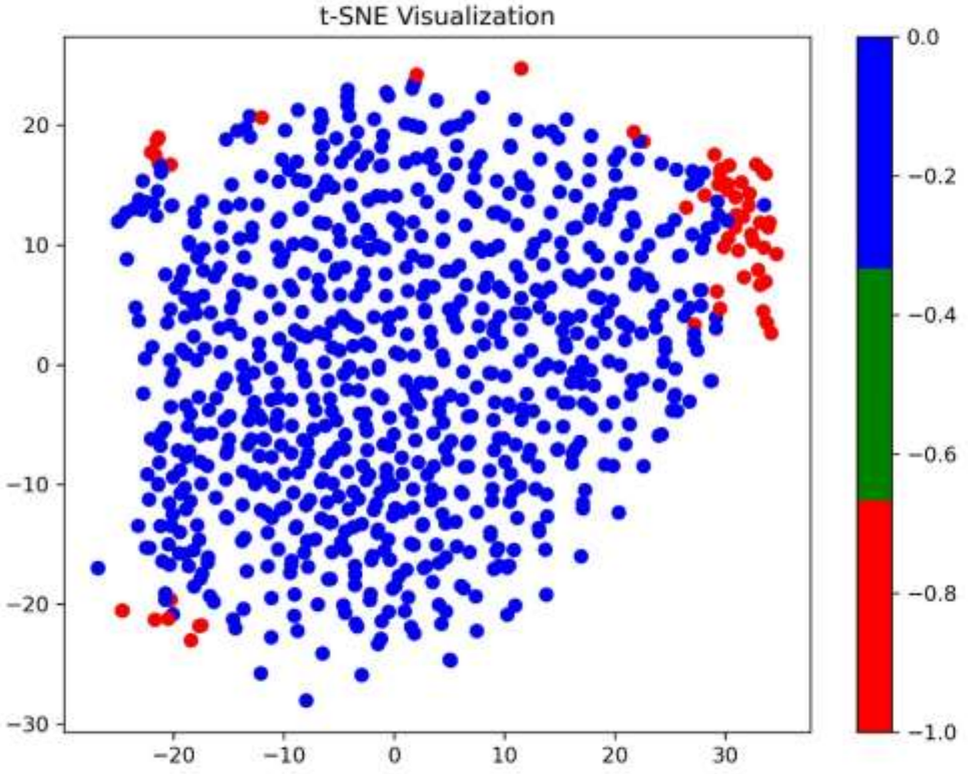

Best model – DBSCAN with Epsilon = 13.8, Min Points = 51.

Left Masseter Muscle

Data visualization:

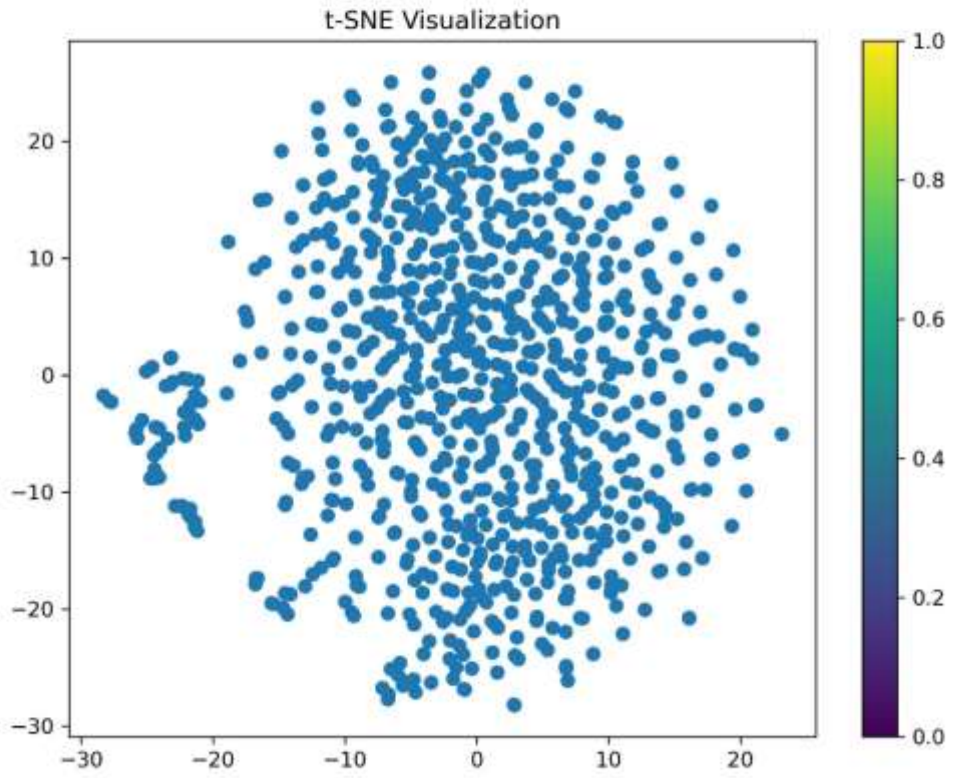

K-Means Elbow and Silhouette:

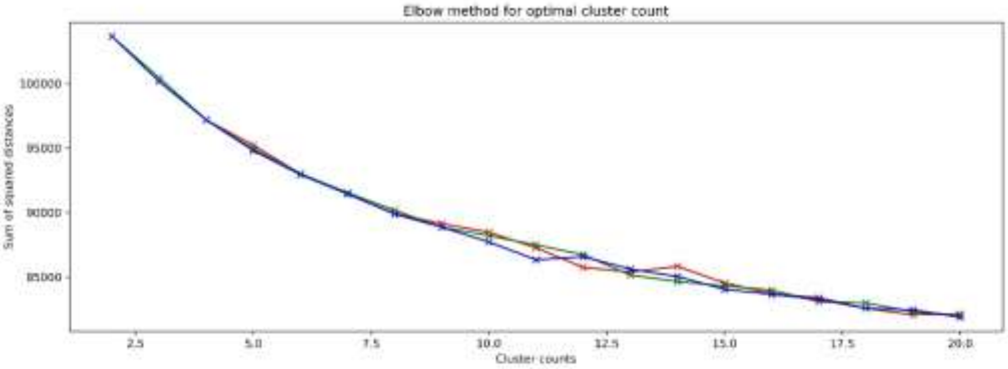

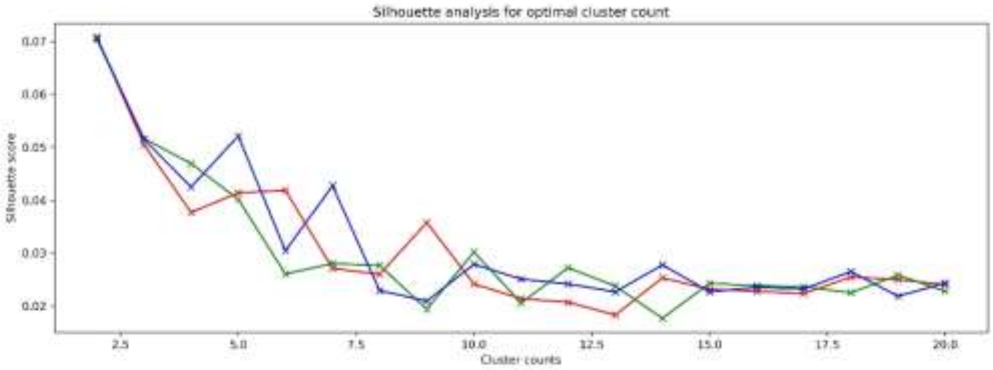

K-Means clustering:

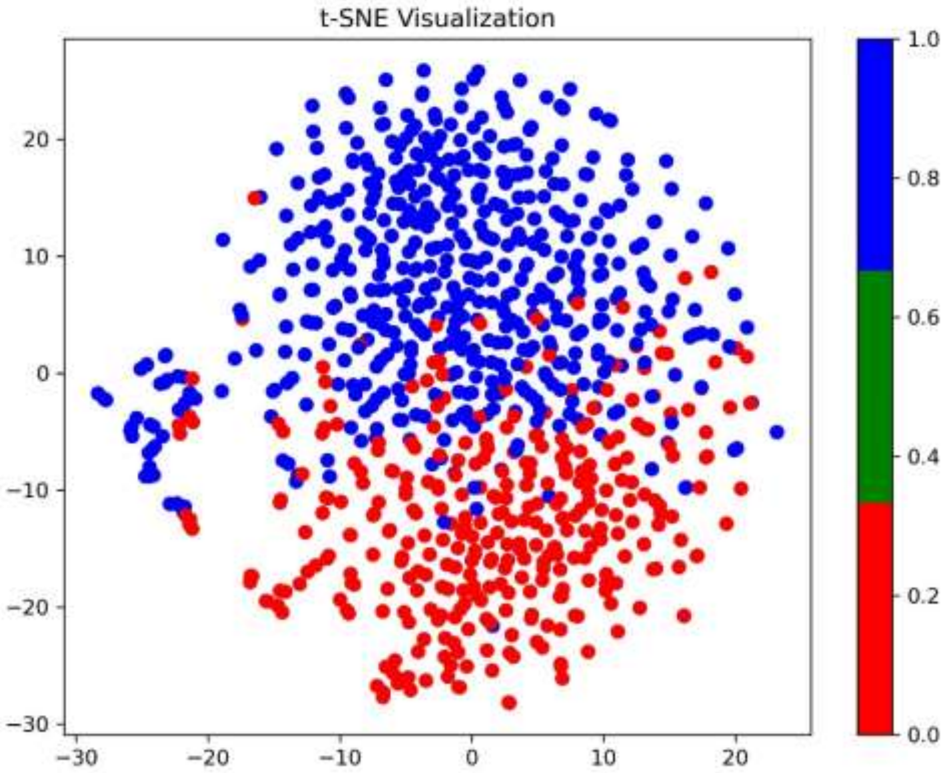

GMM Silhouette and BIC:

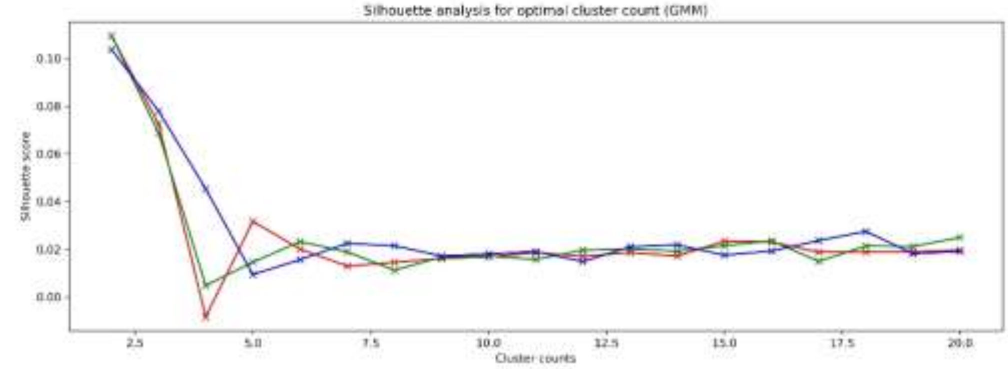

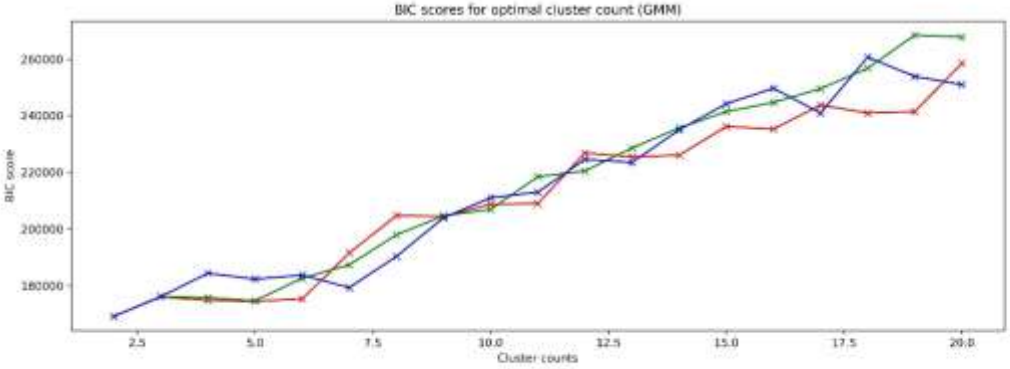

GMM Clustering:

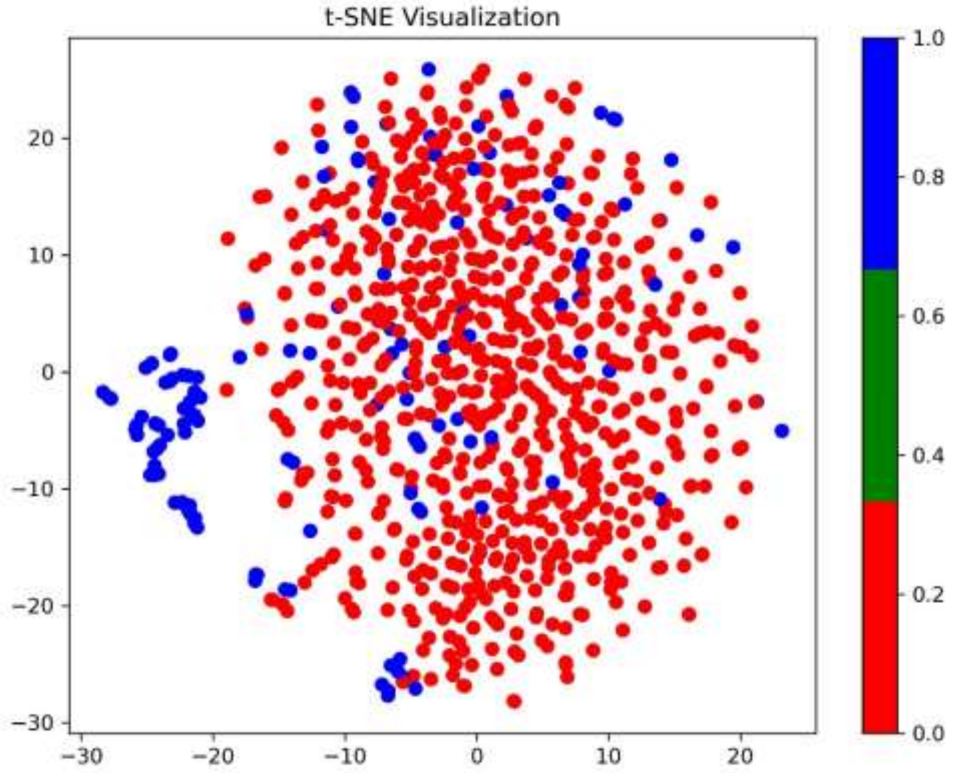

DBSCAN epsilon:

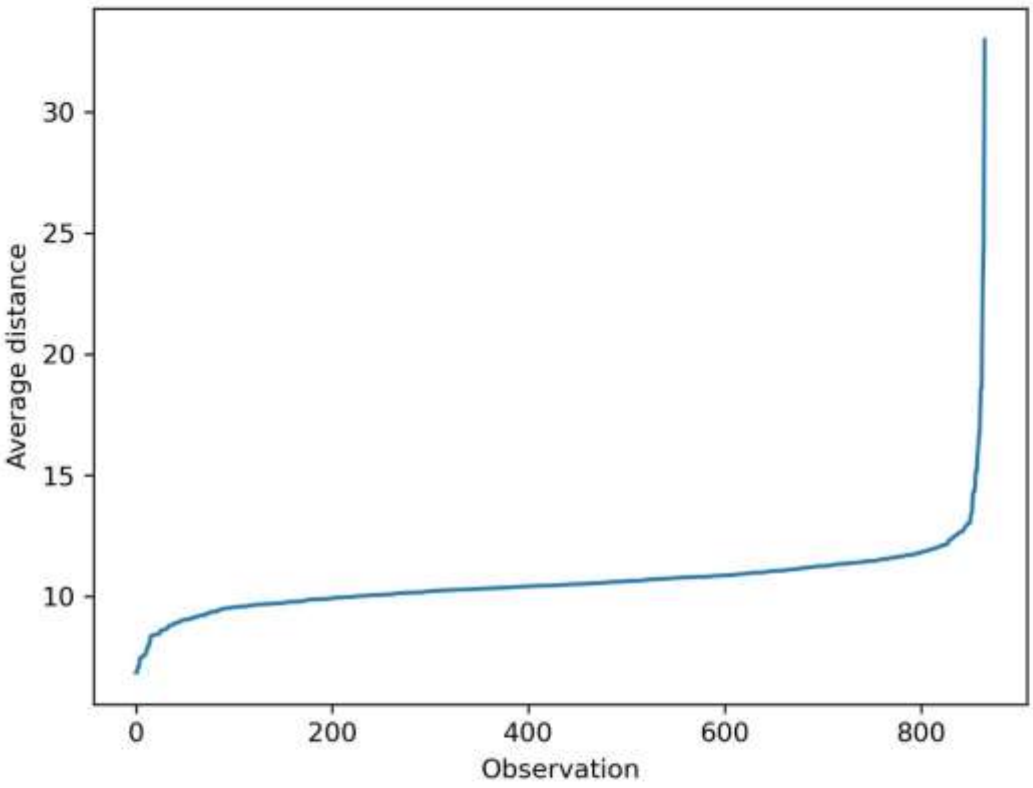

DBSCAN – best based on Davies Bouldin

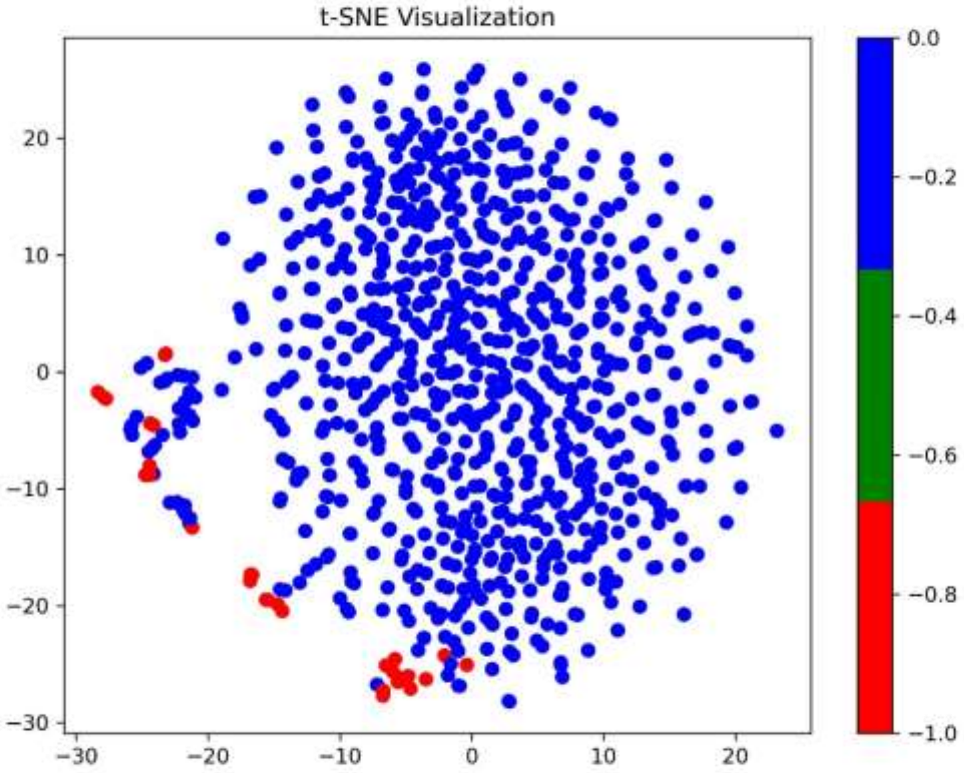

DBSCAN – Best based on Silhouette:

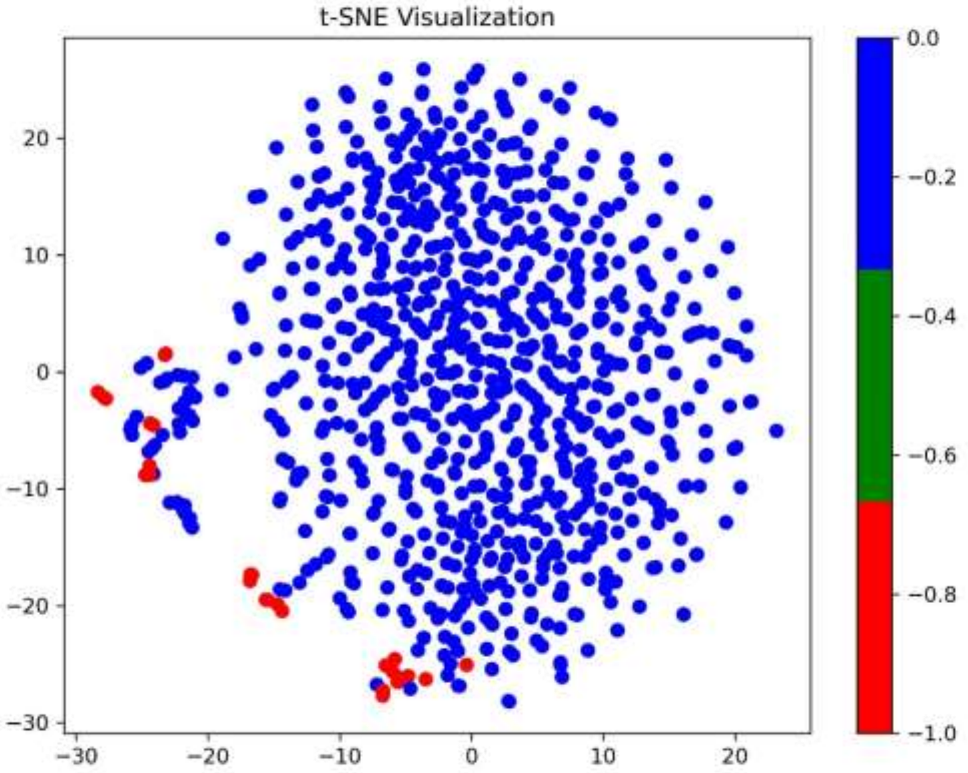

Best model – DBSCAN with Epsilon = 13.8, Min Points = 51.

Right Digastric Muscle

Data visualization:

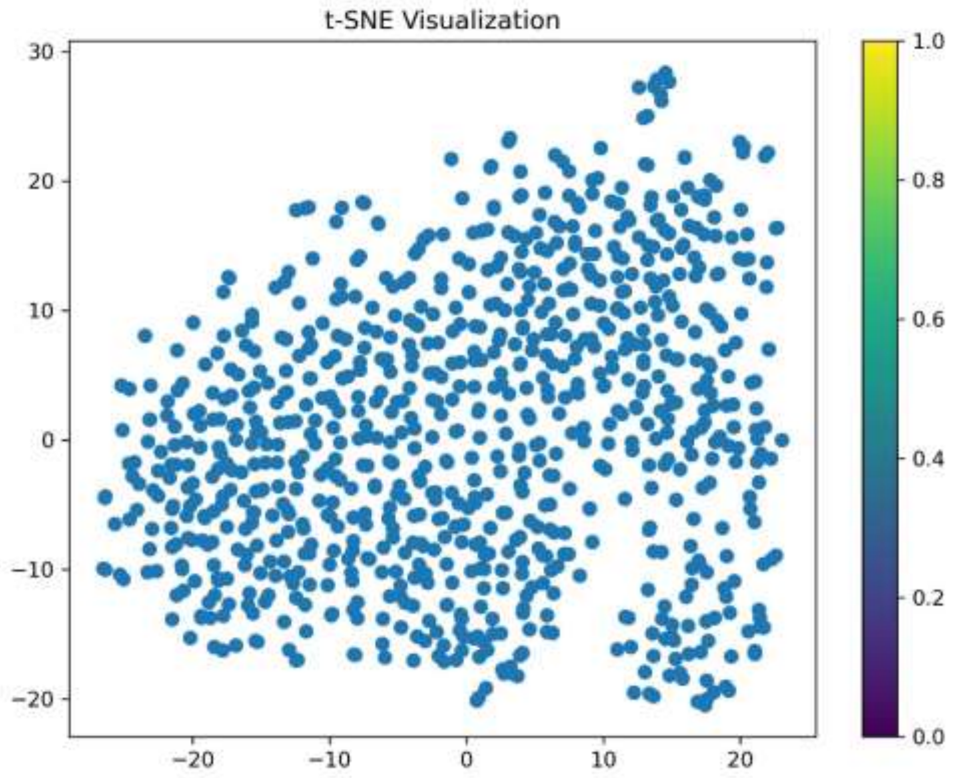

K-Means Elbow and Silhouette:

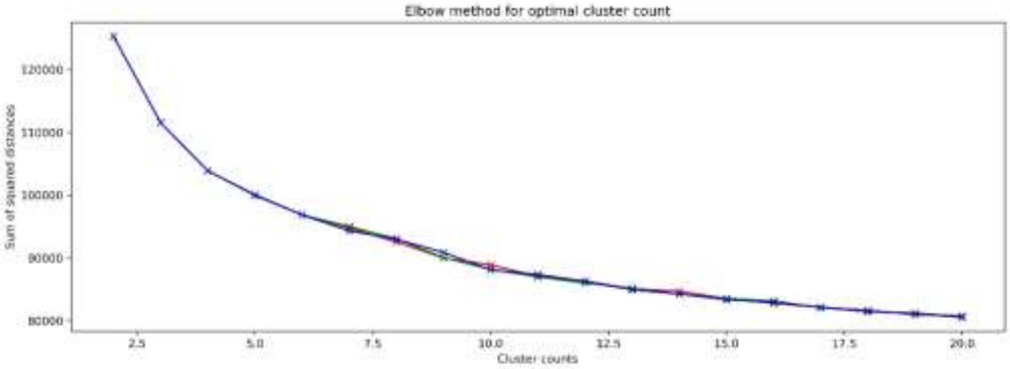

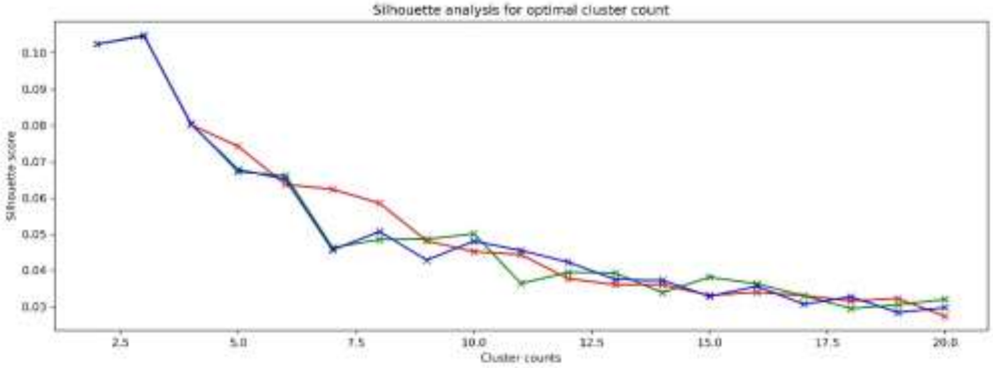

K-Means clustering:

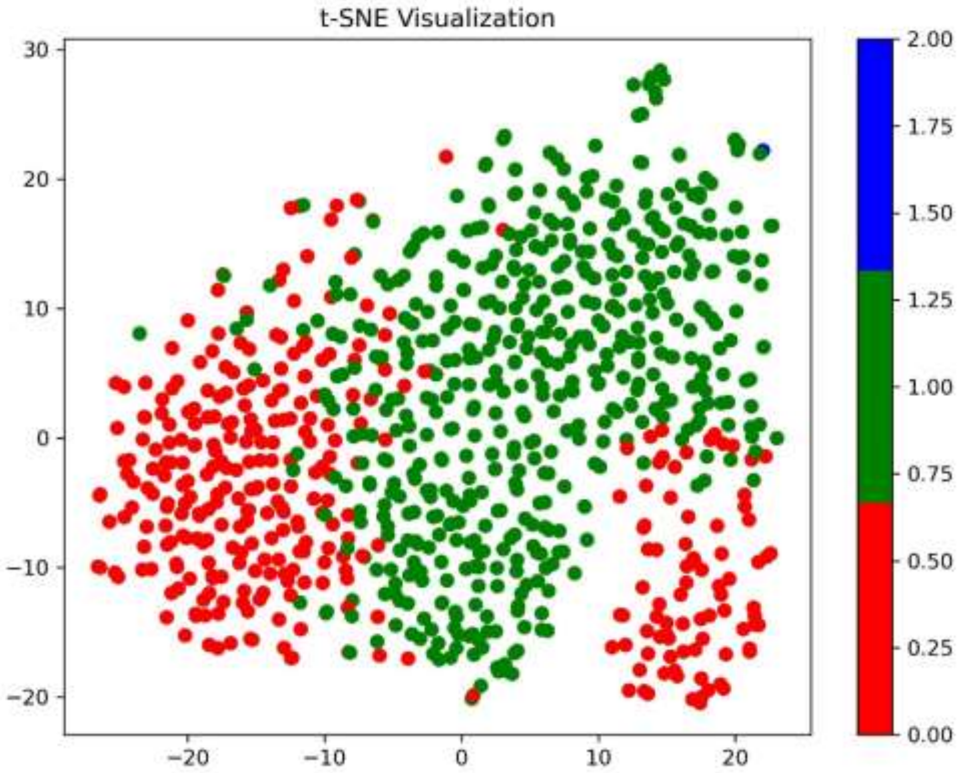

GMM Silhouette and BIC:

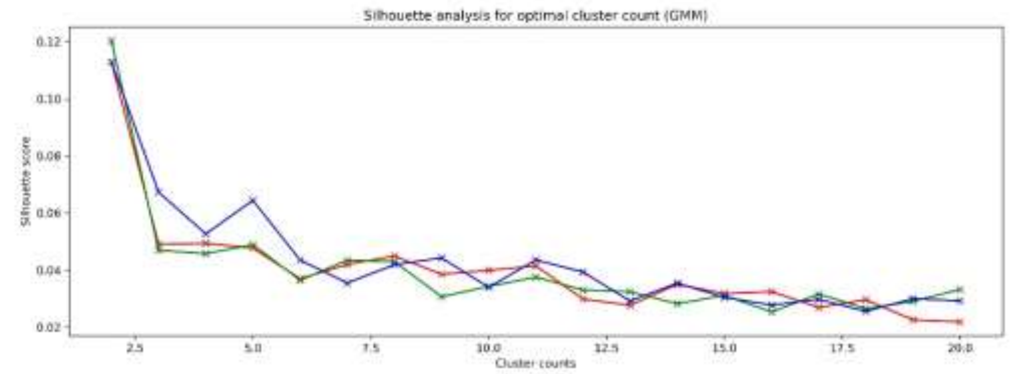

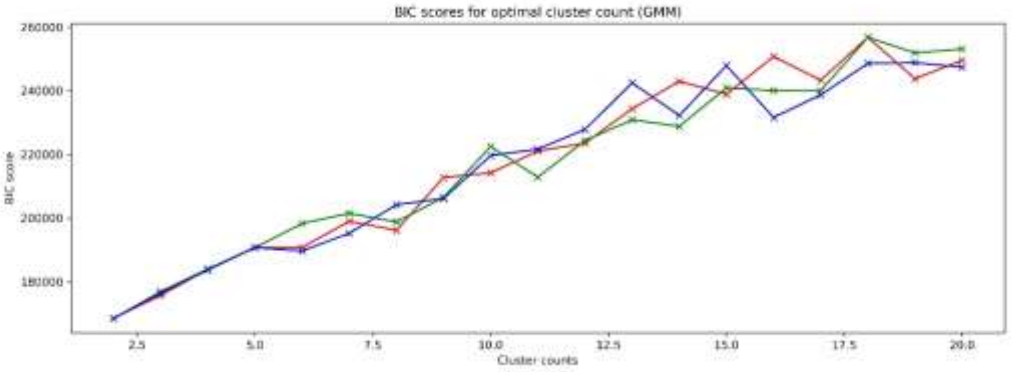

GMM Clustering:

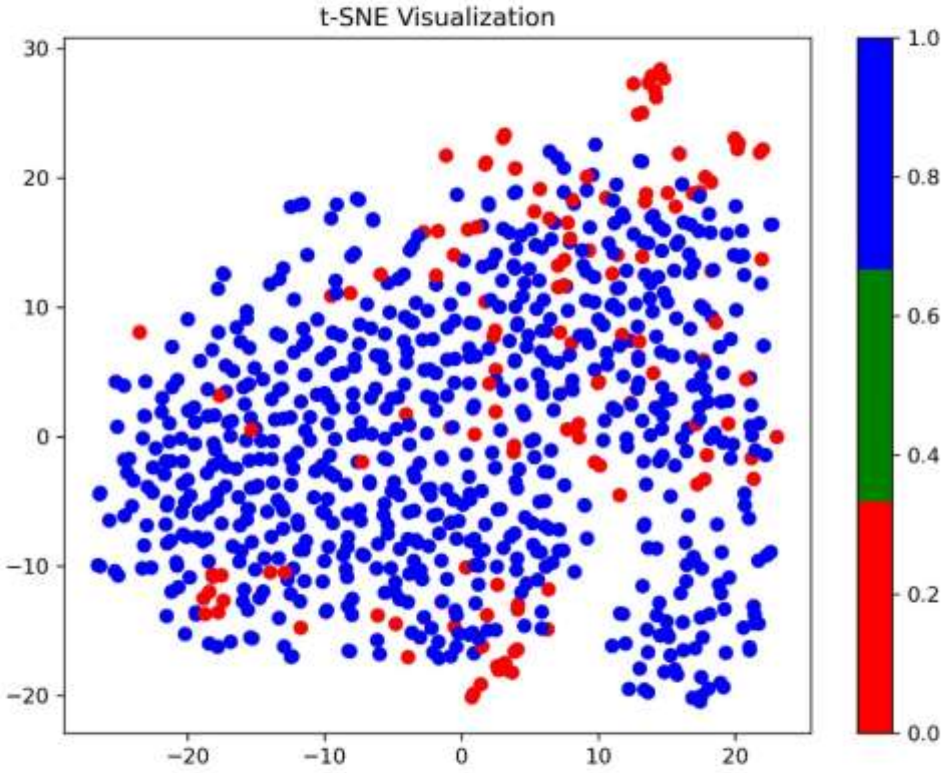

DBSCAN epsilon:

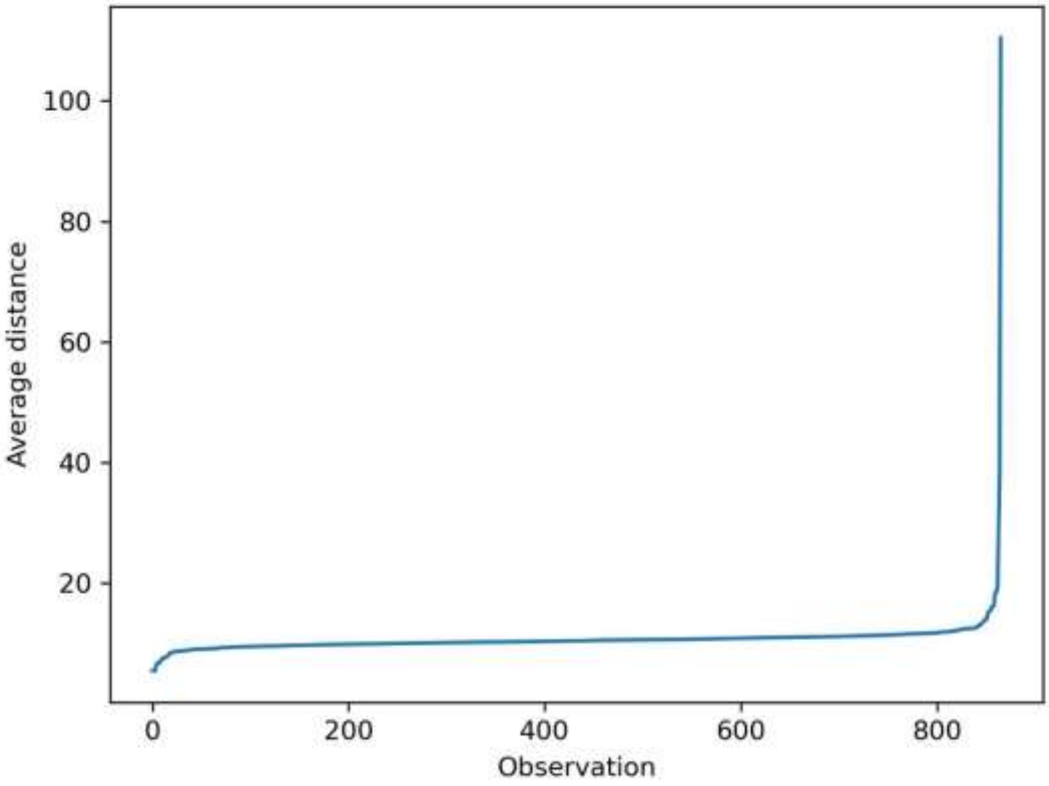

DBSCAN – best based on Davies Bouldin

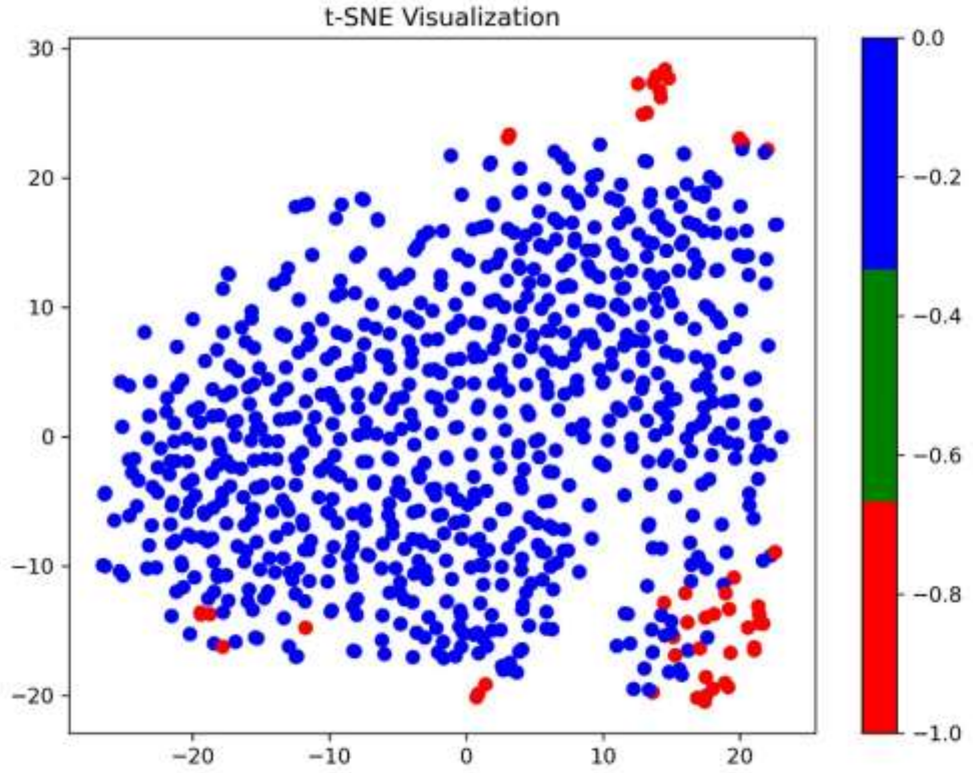

DBSCAN – Best based on Silhouette:

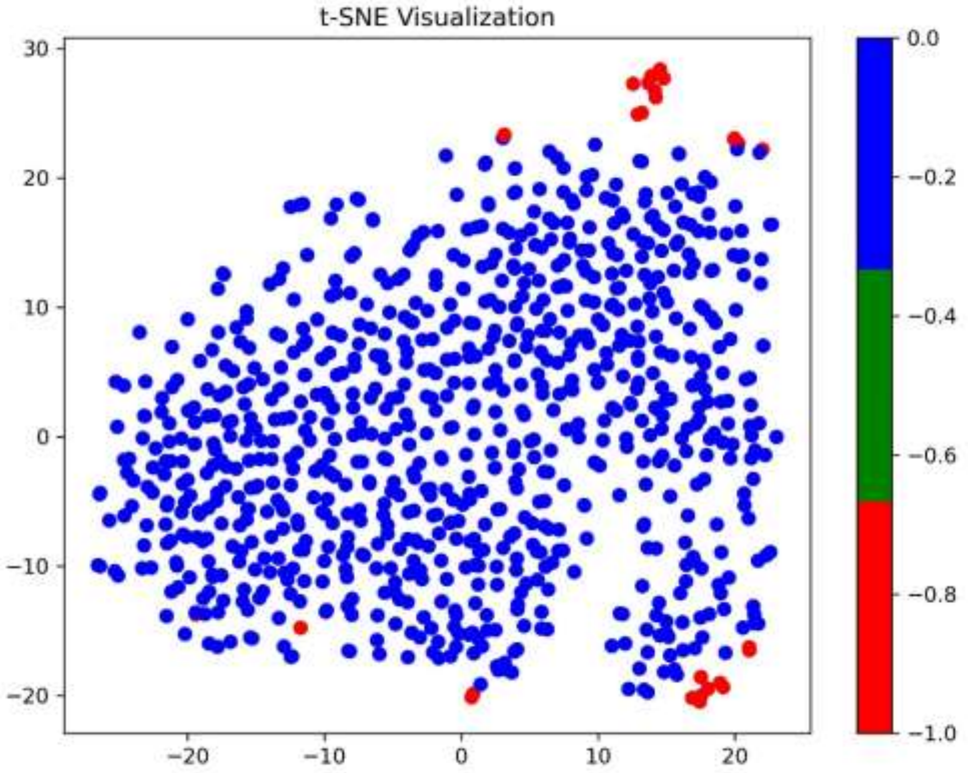

Best model – K-Means with k = 3.

Left Digastric Muscle

Data visualization:

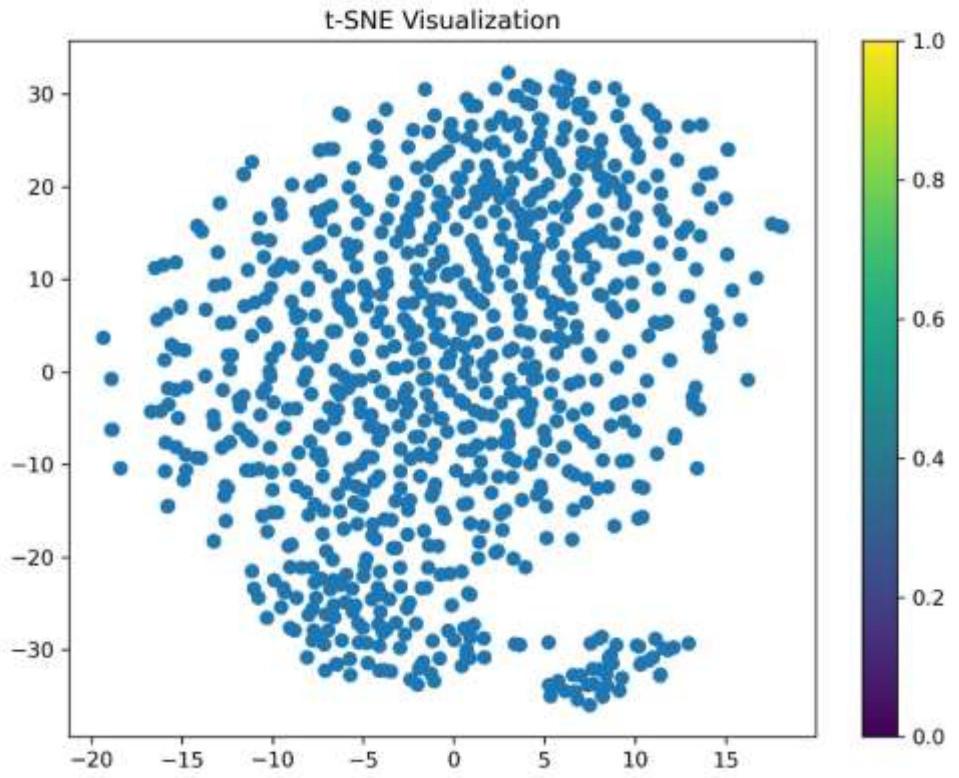

K-Means Elbow and Silhouette:

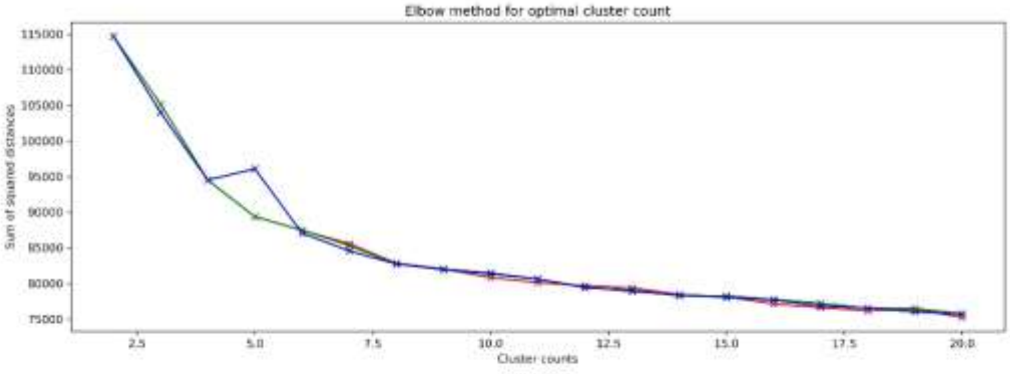

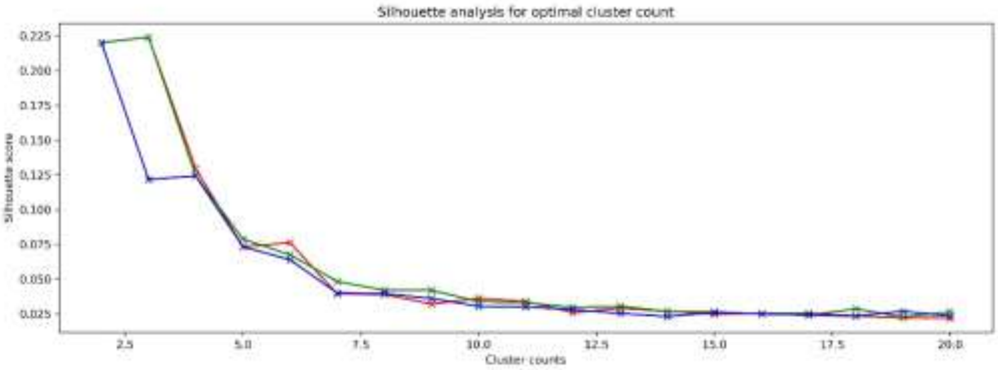

K-Means clustering:

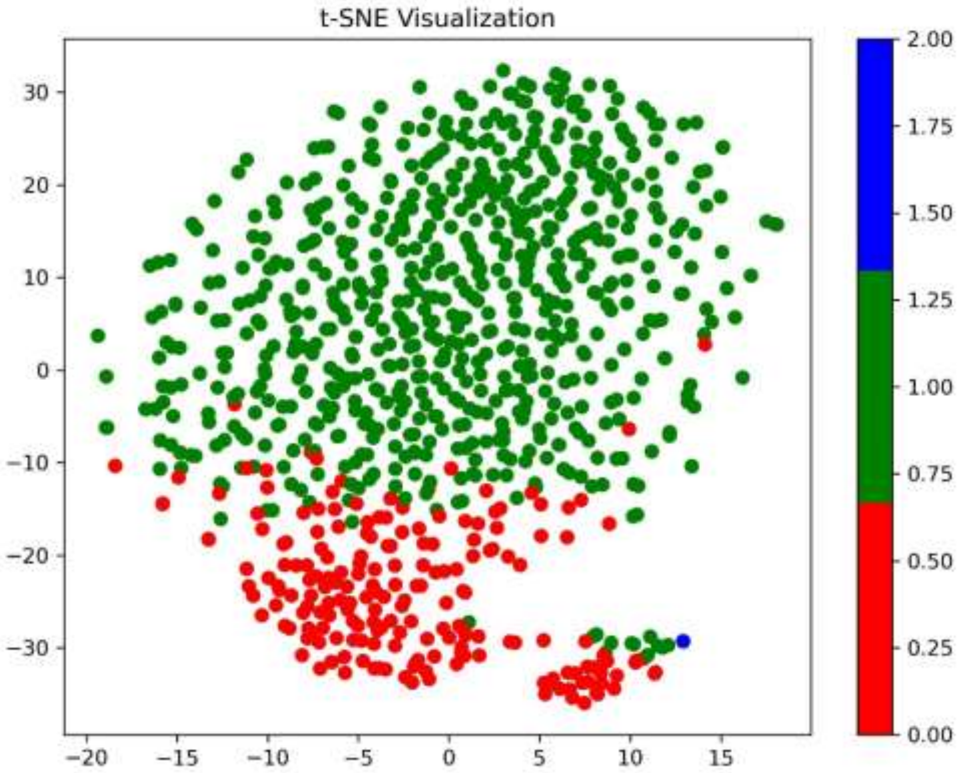

GMM Silhouette and BIC:

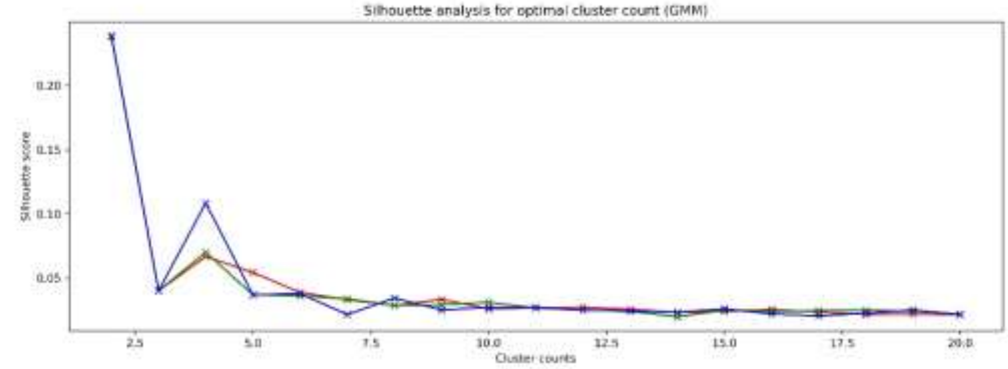

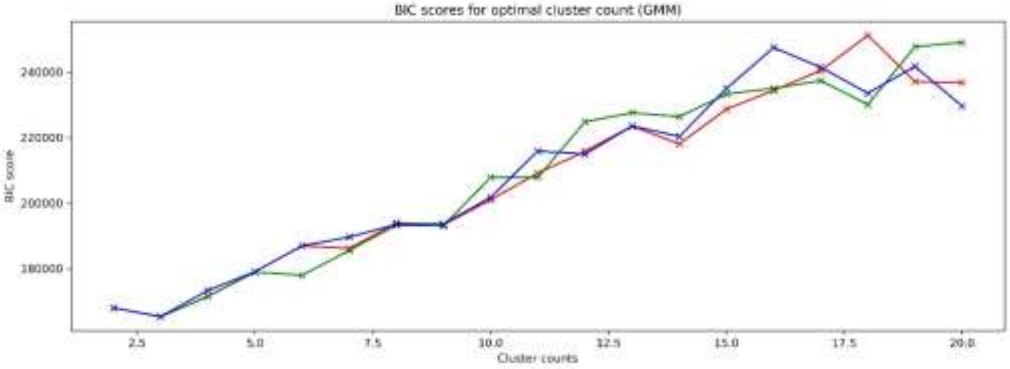

GMM Clustering:

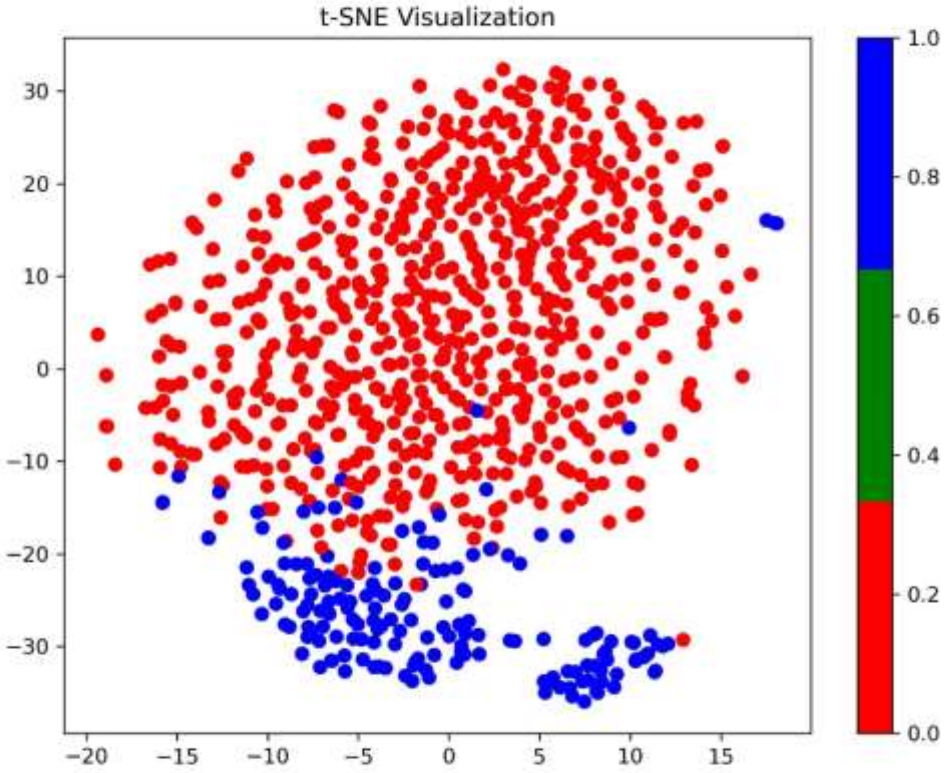

DBSCAN epsilon:

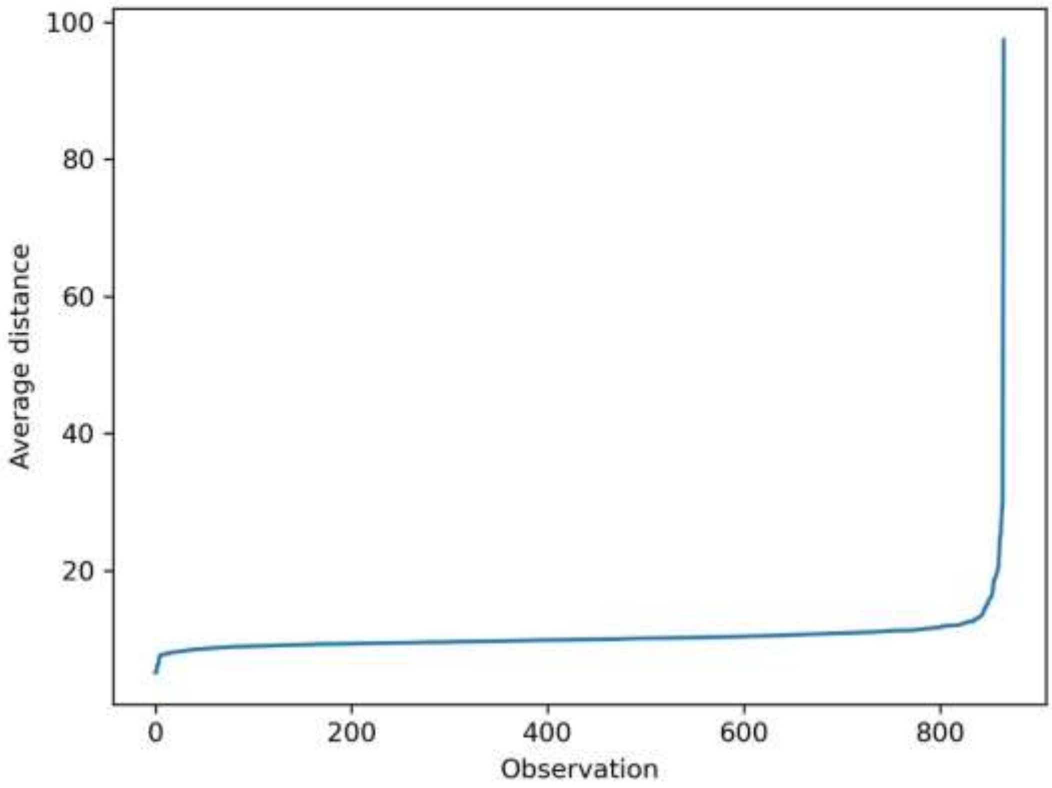

DBSCAN – best based on Davies Bouldin

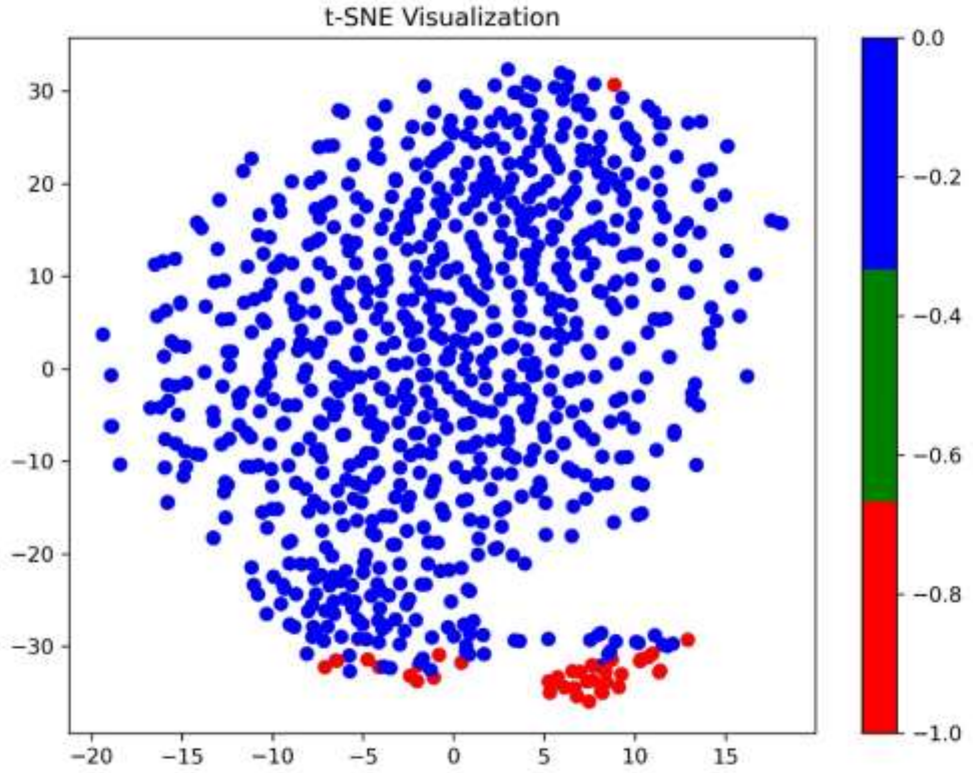

DBSCAN – Best based on Silhouette:

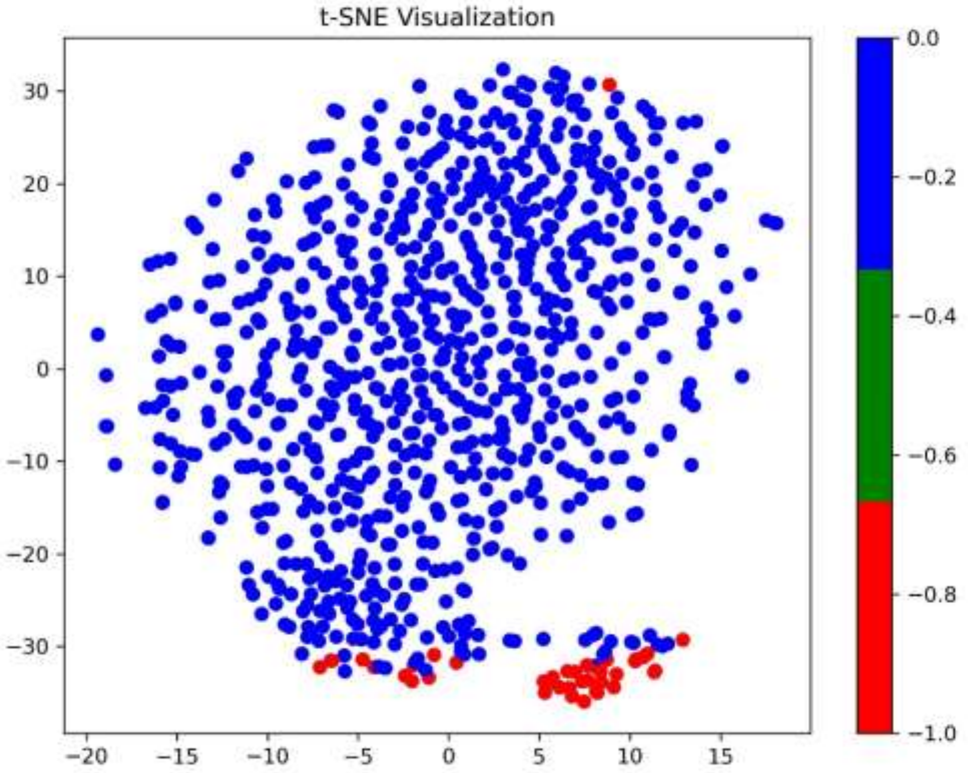

Best model – GMM with k = 2.

Chewing

Right Temporalis

Data visualization:

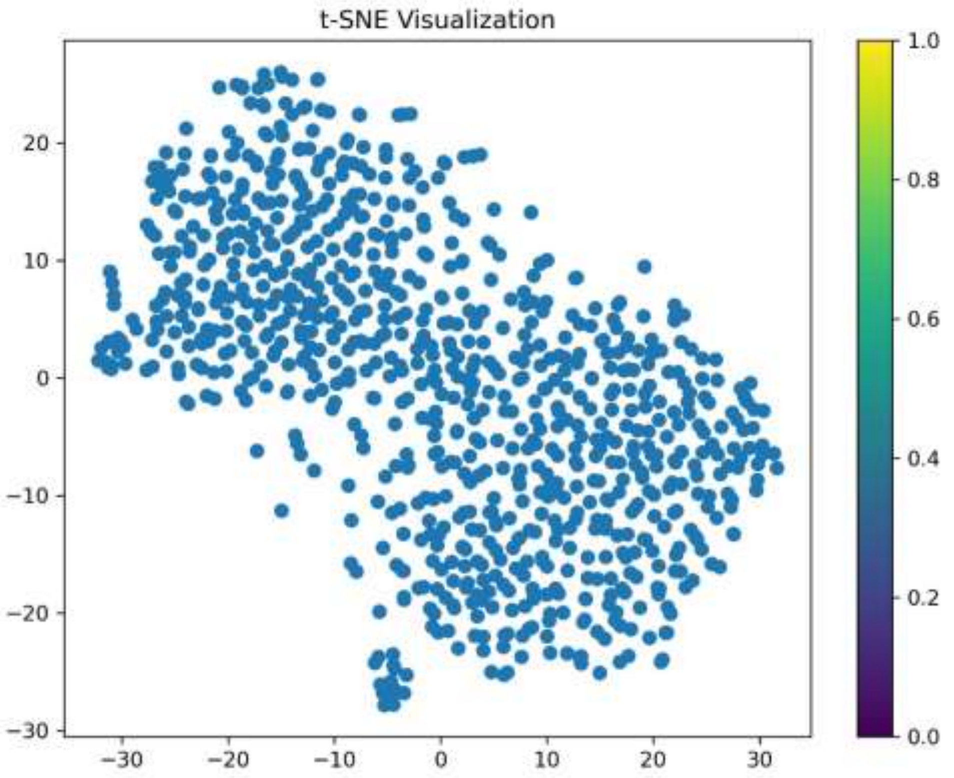

K-Means Elbow and Silhouette:

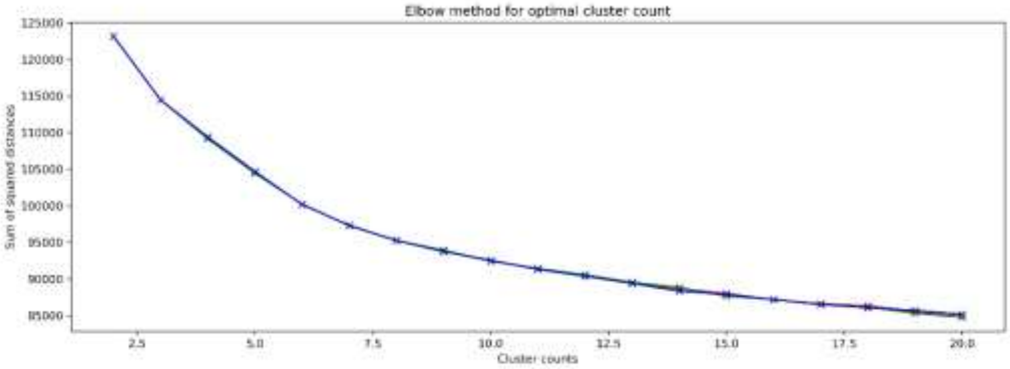

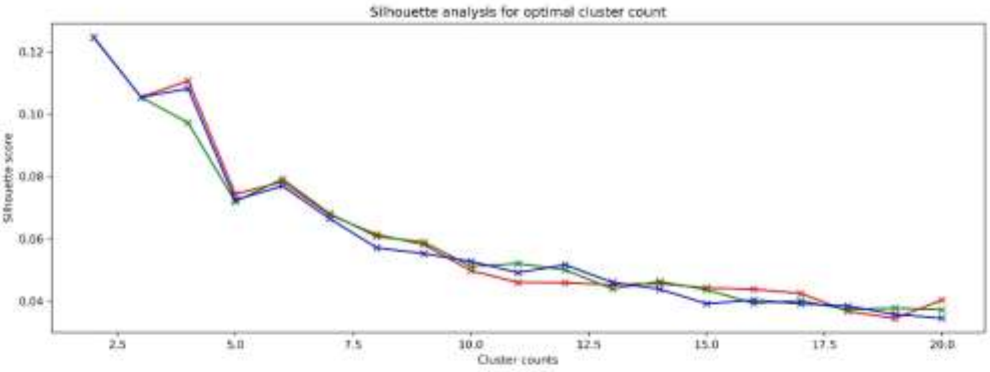

K-Means clustering:

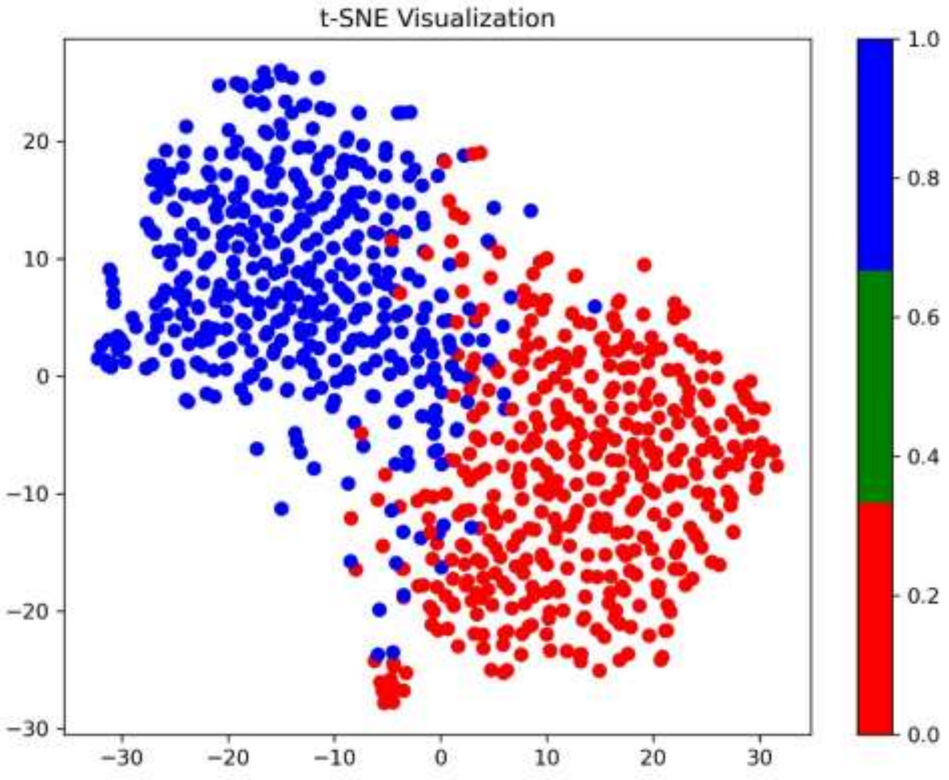

GMM Silhouette and BIC:

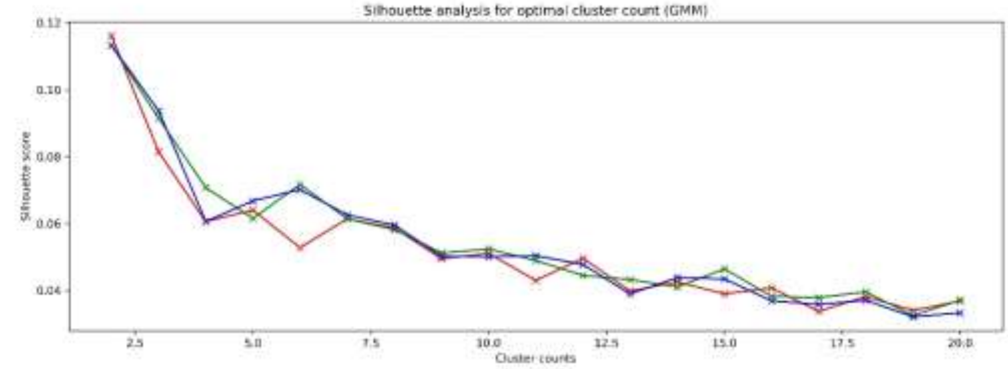

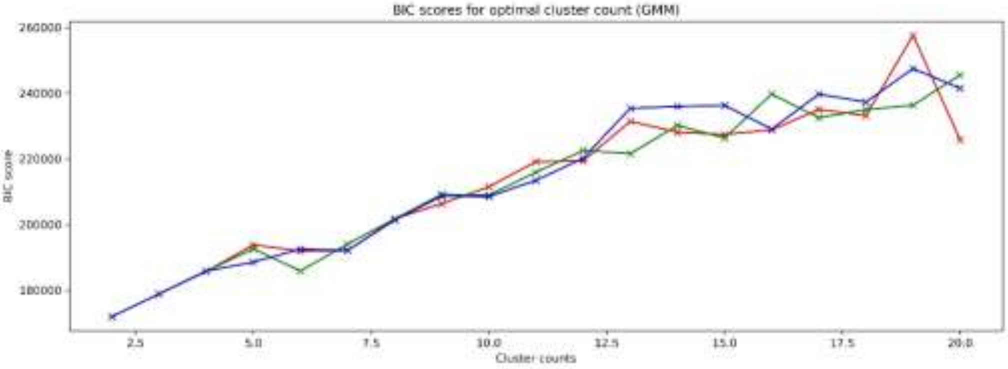

GMM Clustering:

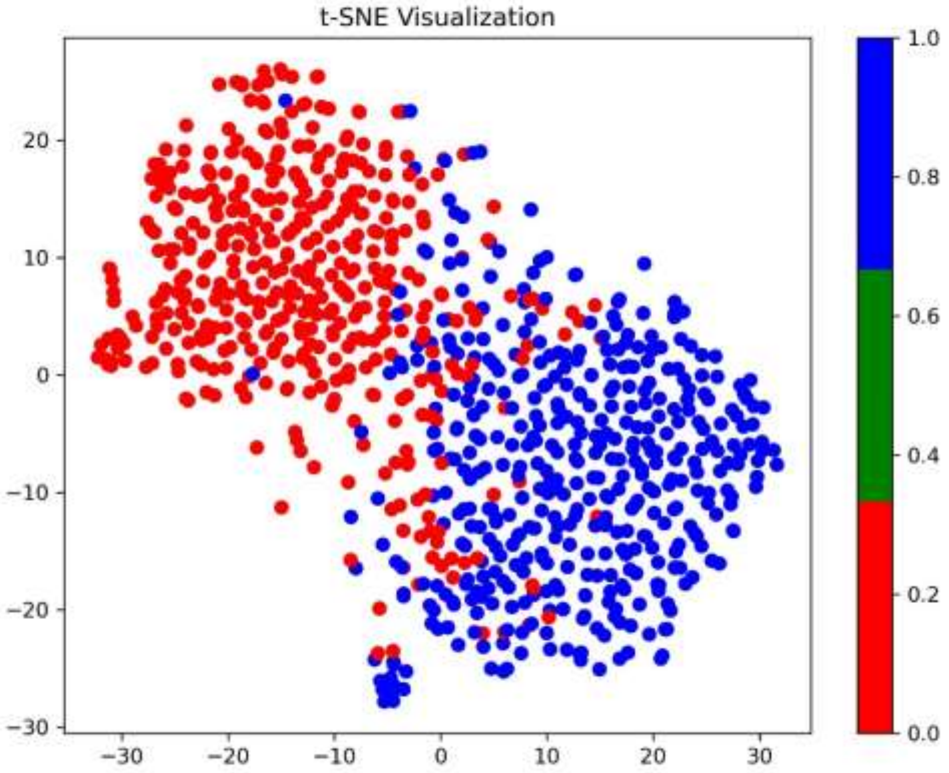

DBSCAN epsilon:

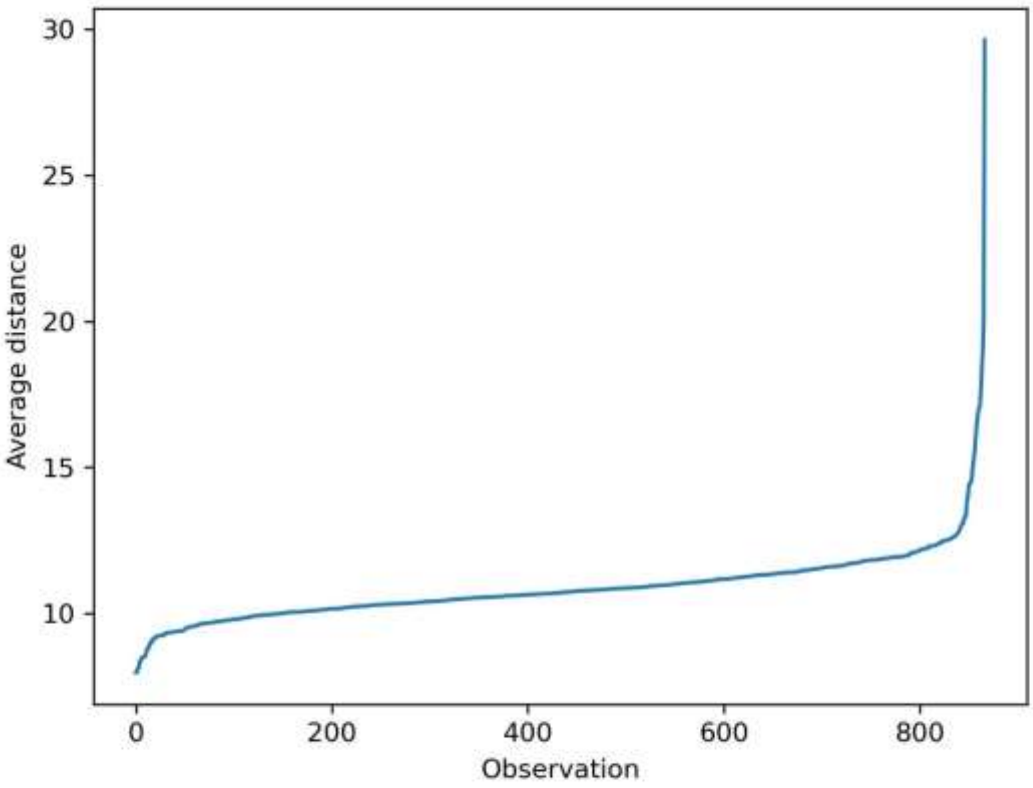

DBSCAN – best based on Davies Bouldin

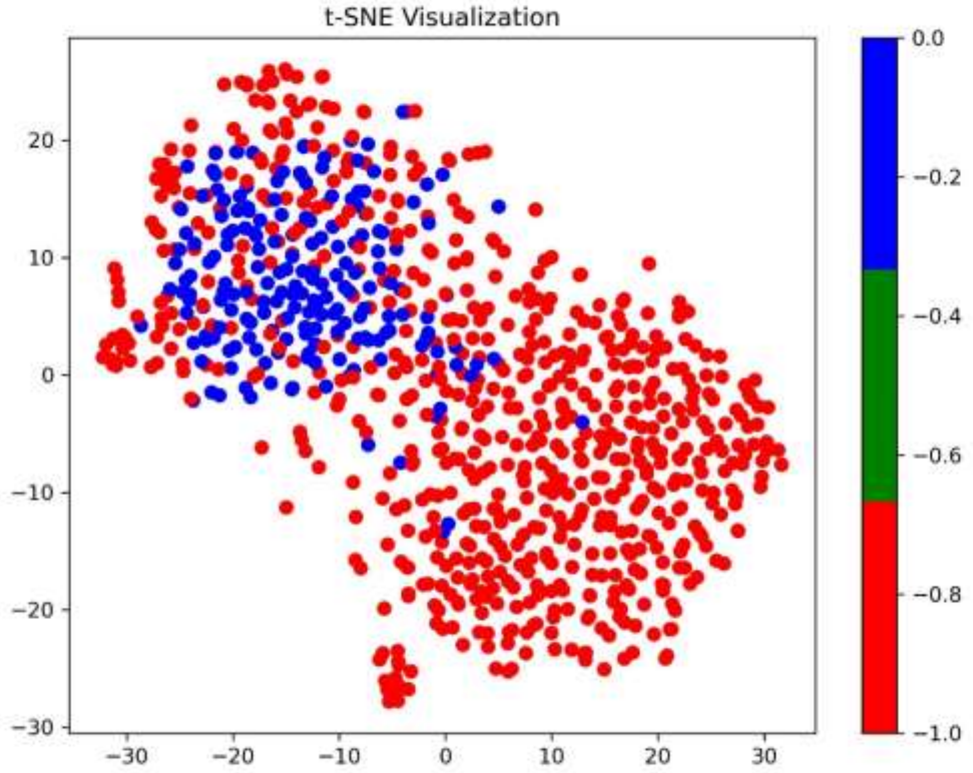

DBSCAN – Best based on Silhouette:

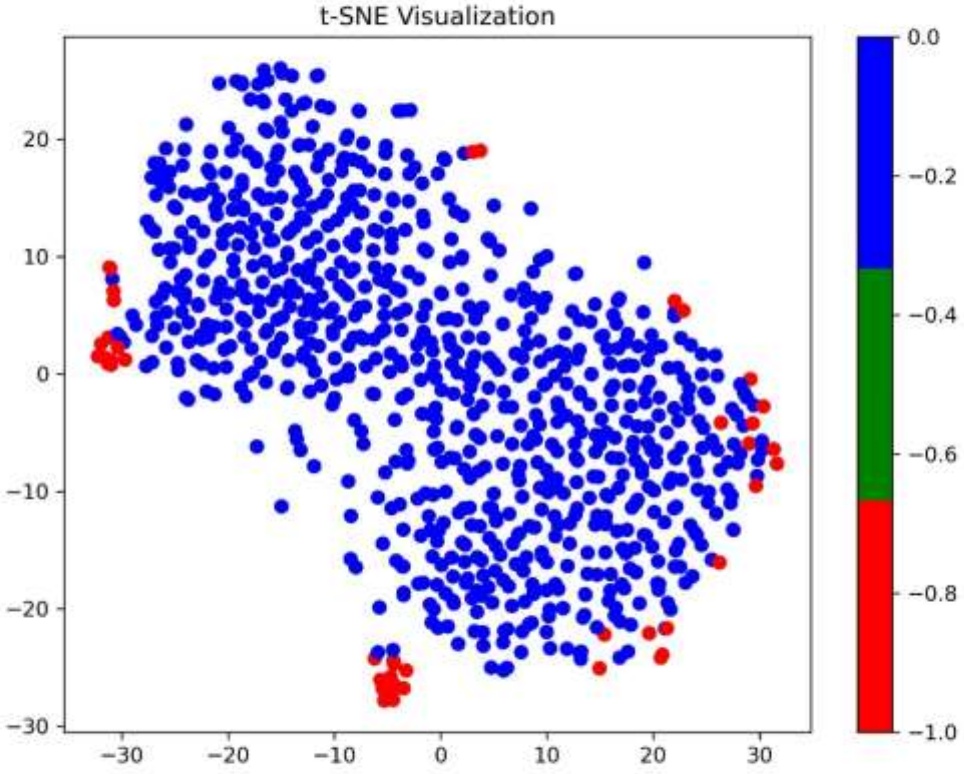

Best model – K-Means with k = 2

Left Temporalis

Data visualization:

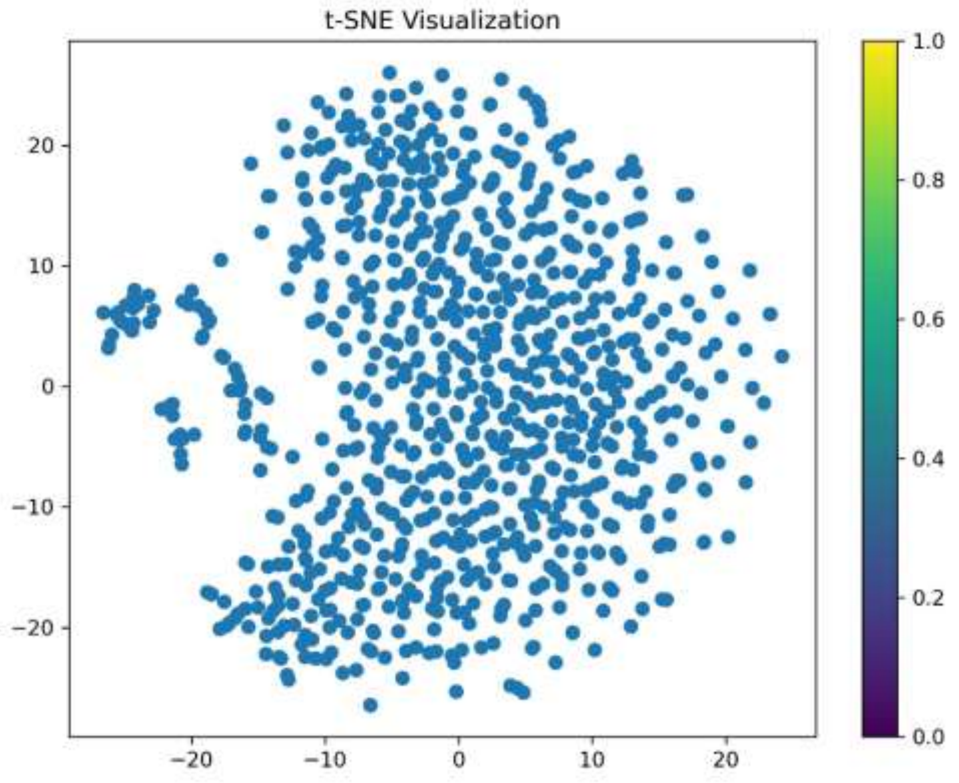

K-Means Elbow and Silhouette:

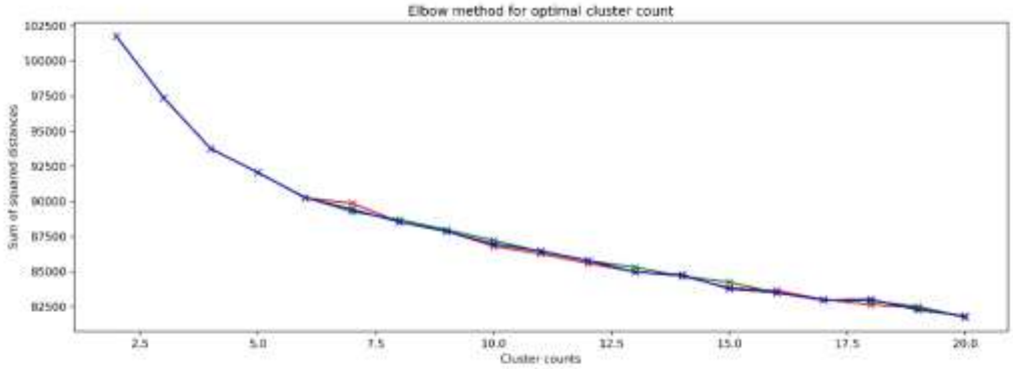

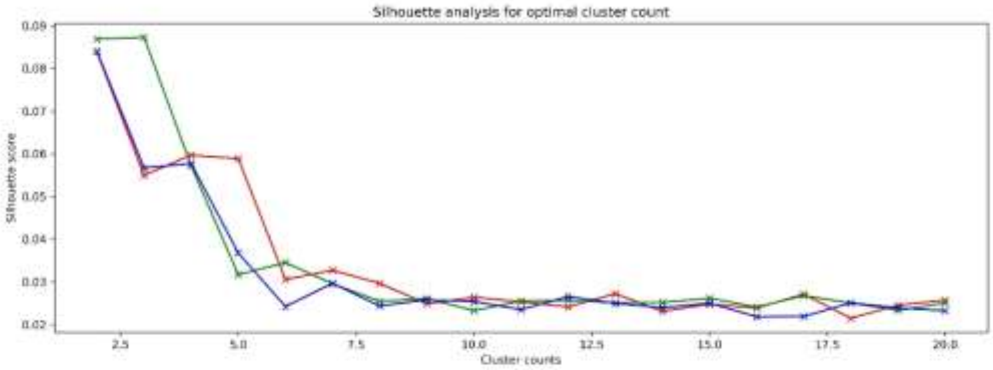

K-Means clustering:

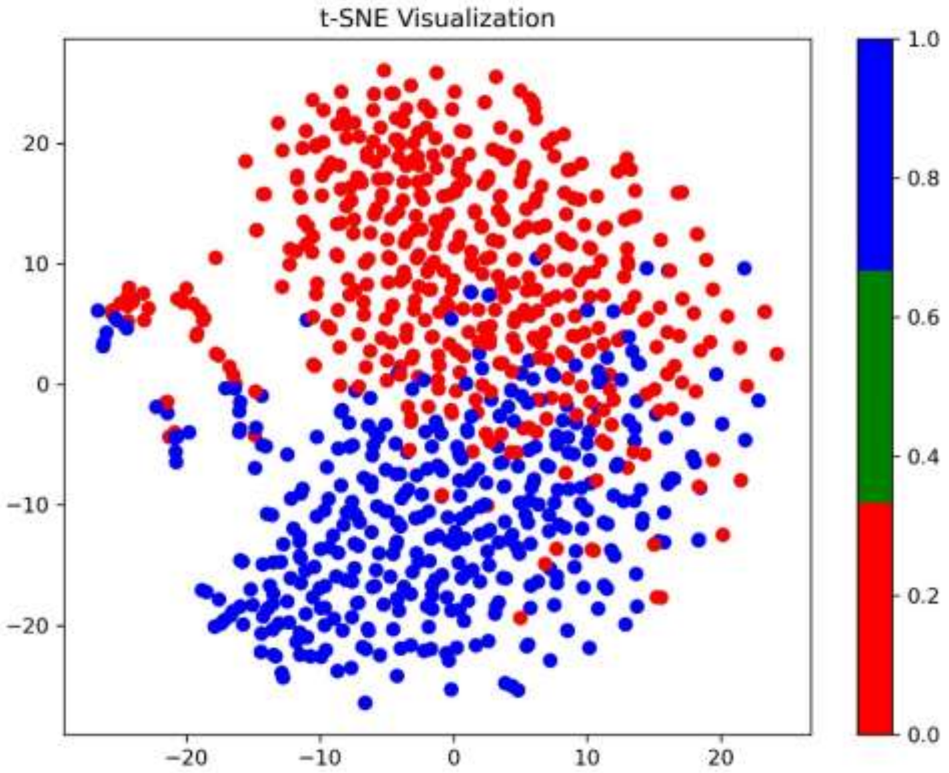

GMM Silhouette and BIC:

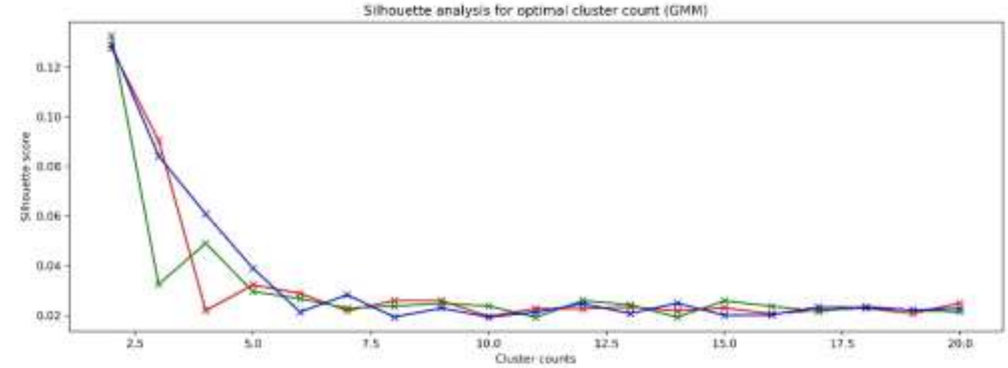

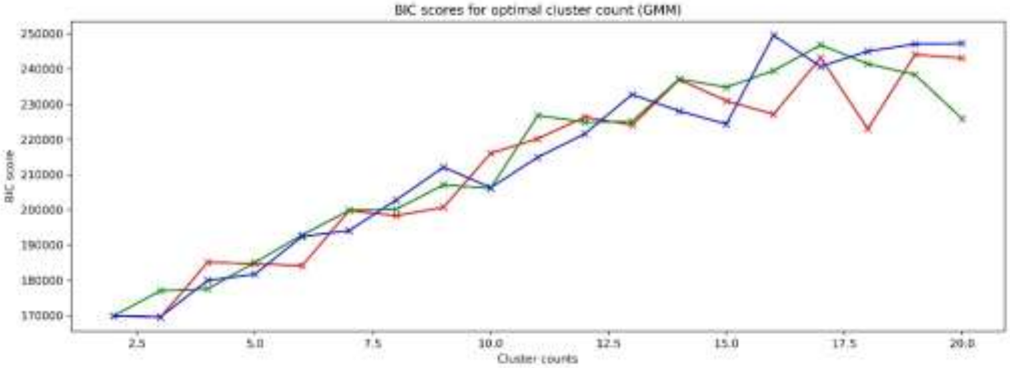

GMM Clustering:

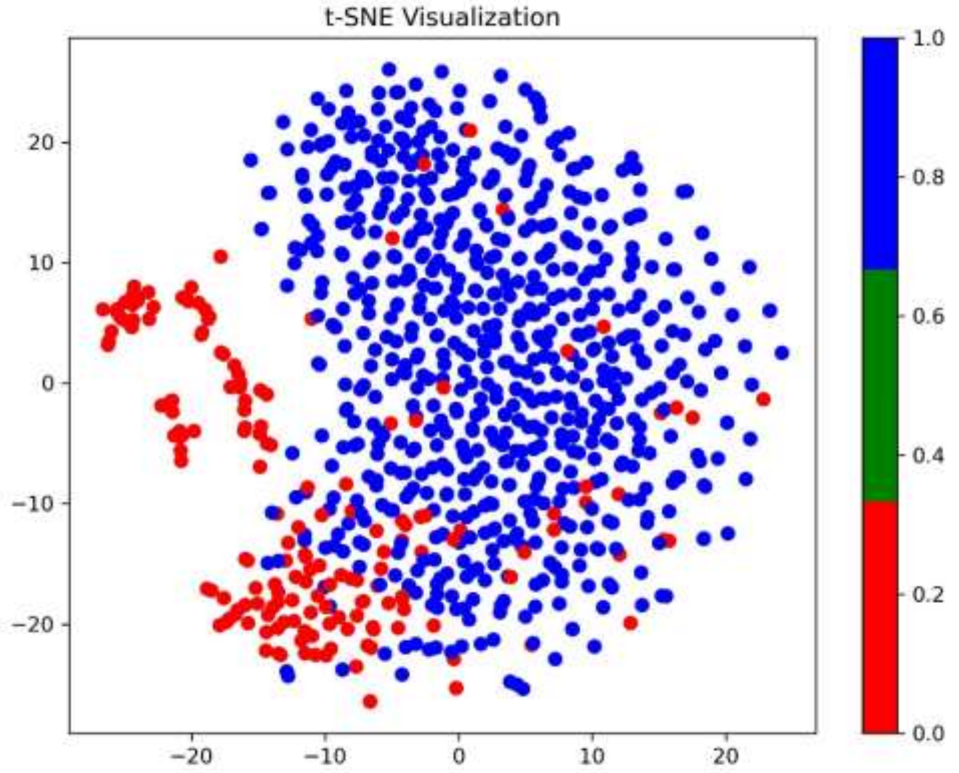

DBSCAN epsilon:

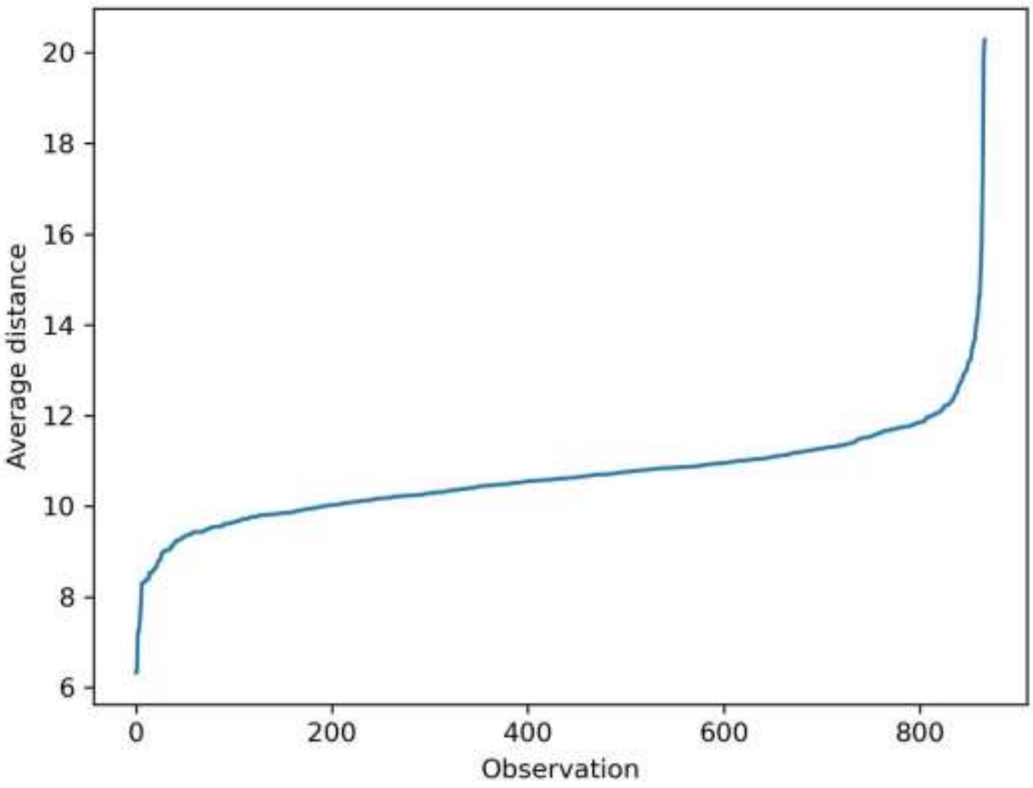

DBSCAN – best based on Davies Bouldin

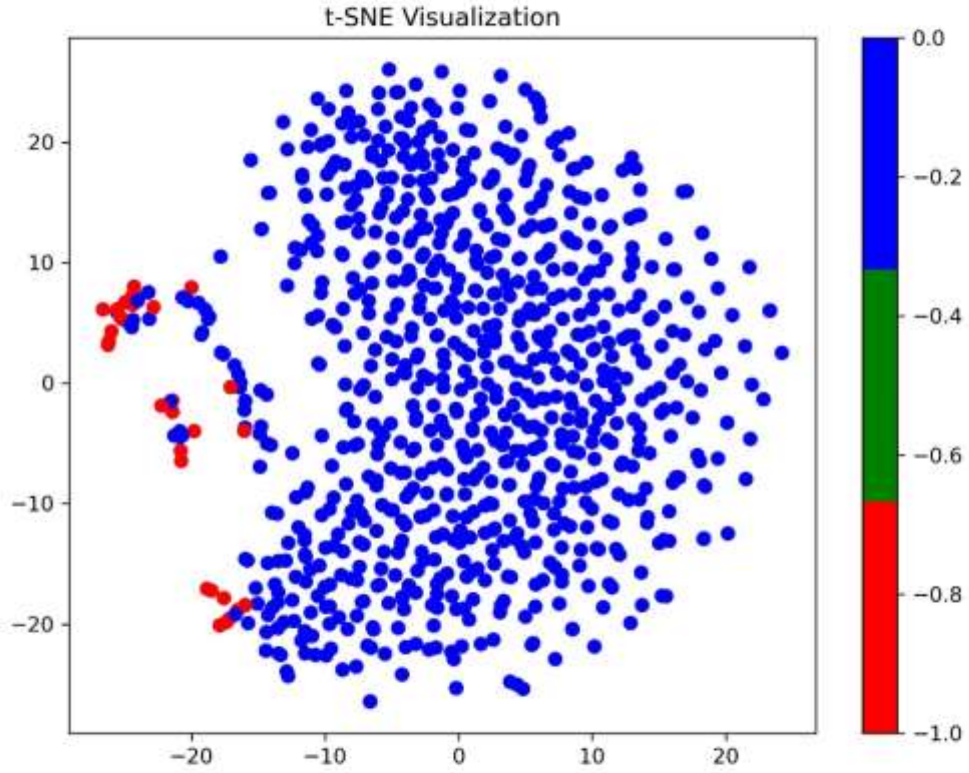

DBSCAN – Best based on Silhouette:

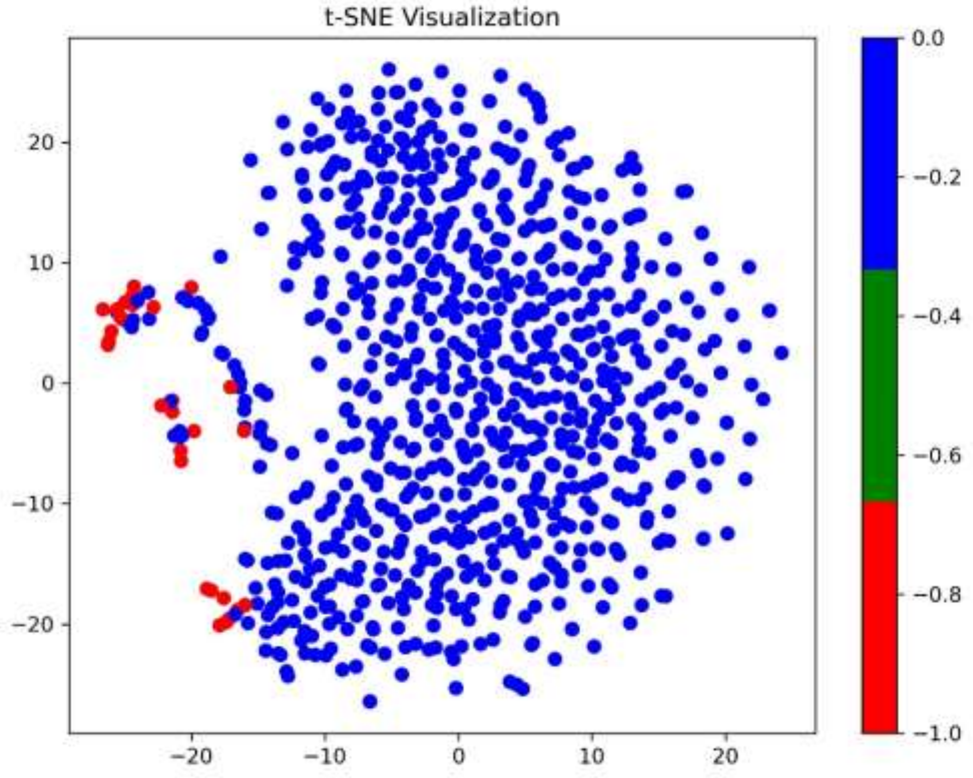

Best model – DBSCAN with Epsilon = 12.8, Min Points = 81.

Right Masseter Muscle

Data visualization:

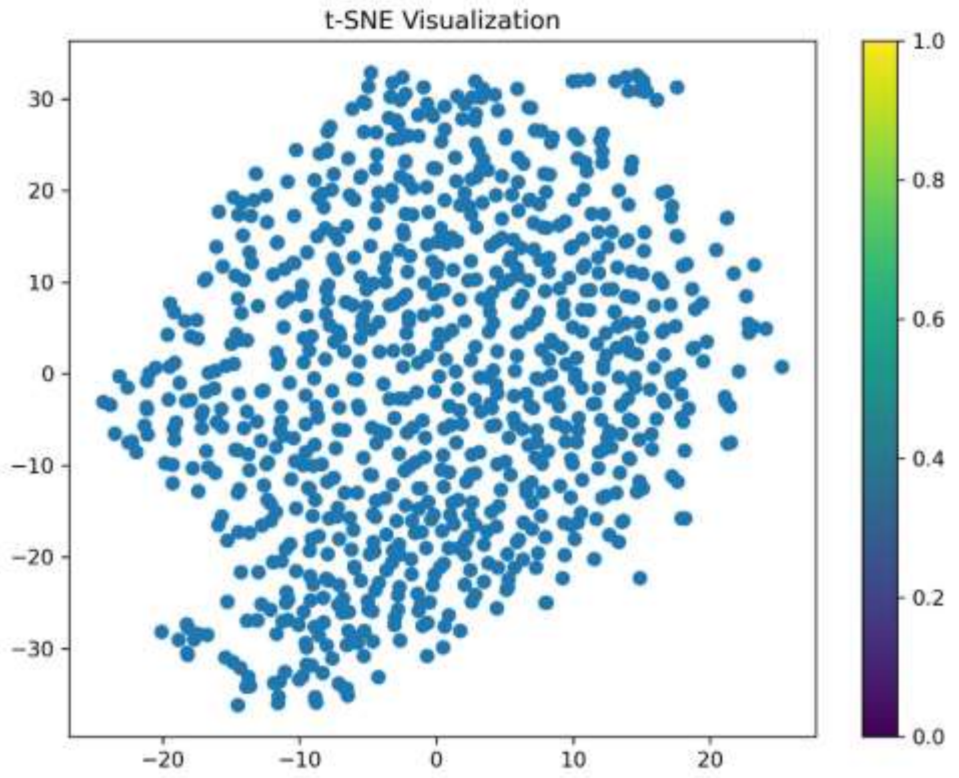

K-Means Elbow and Silhouette:

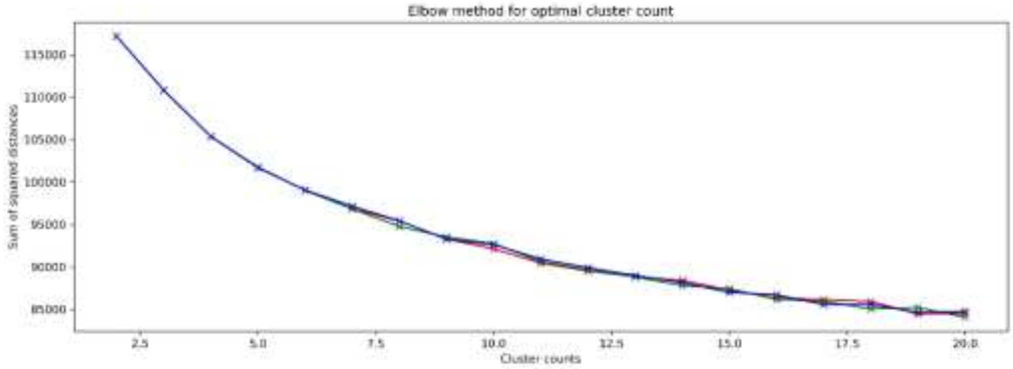

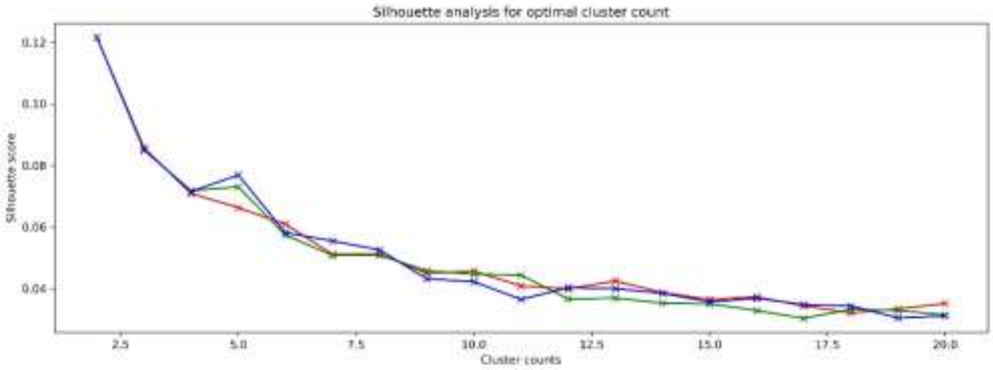

K-Means clustering:

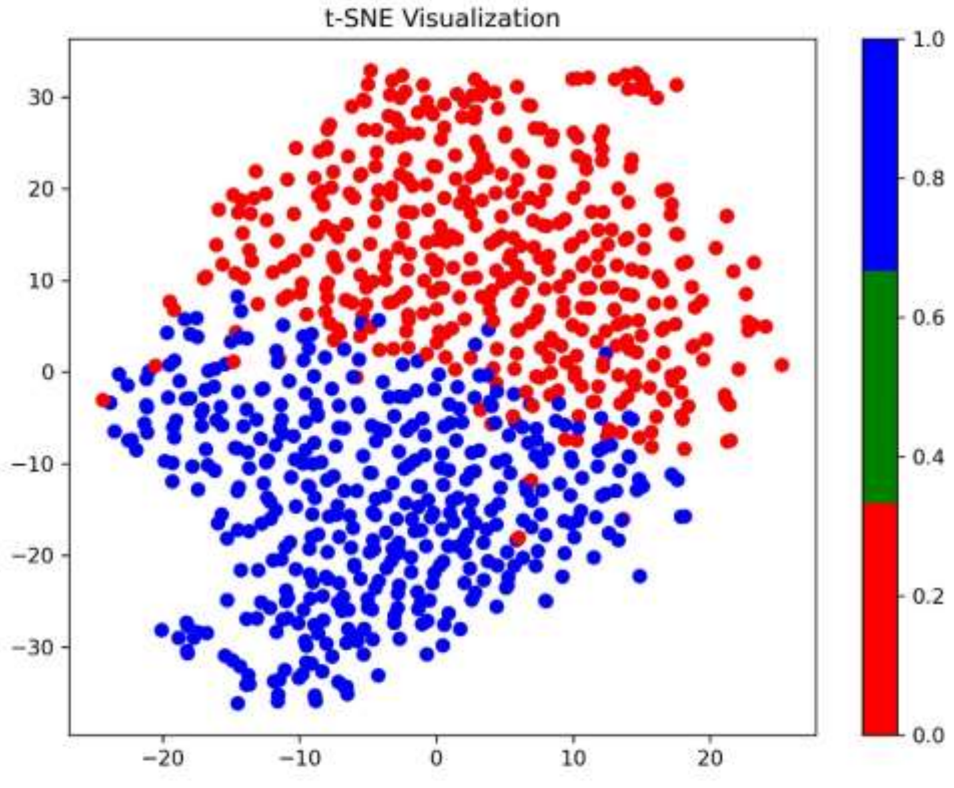

GMM Silhouette and BIC:

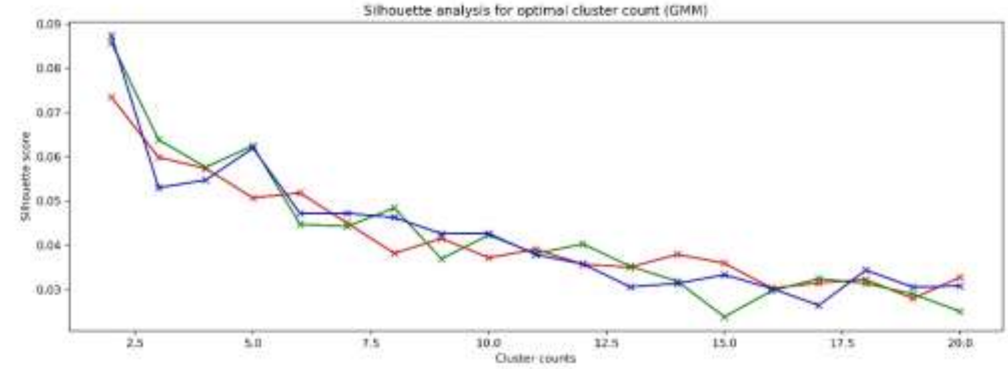

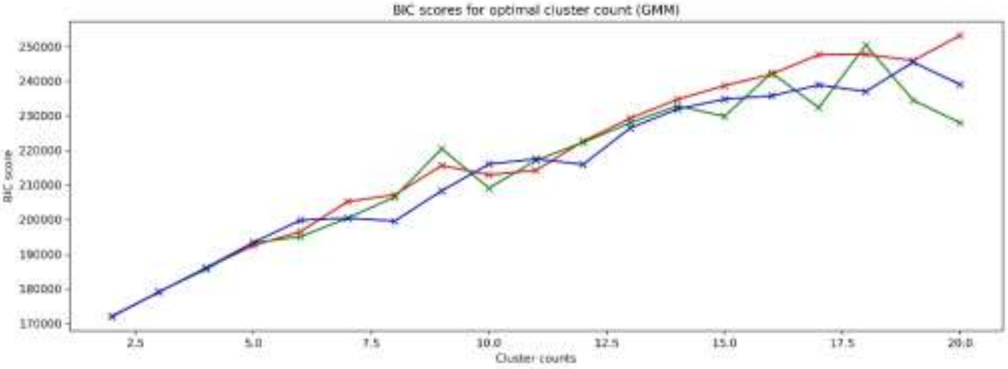

GMM Clustering:

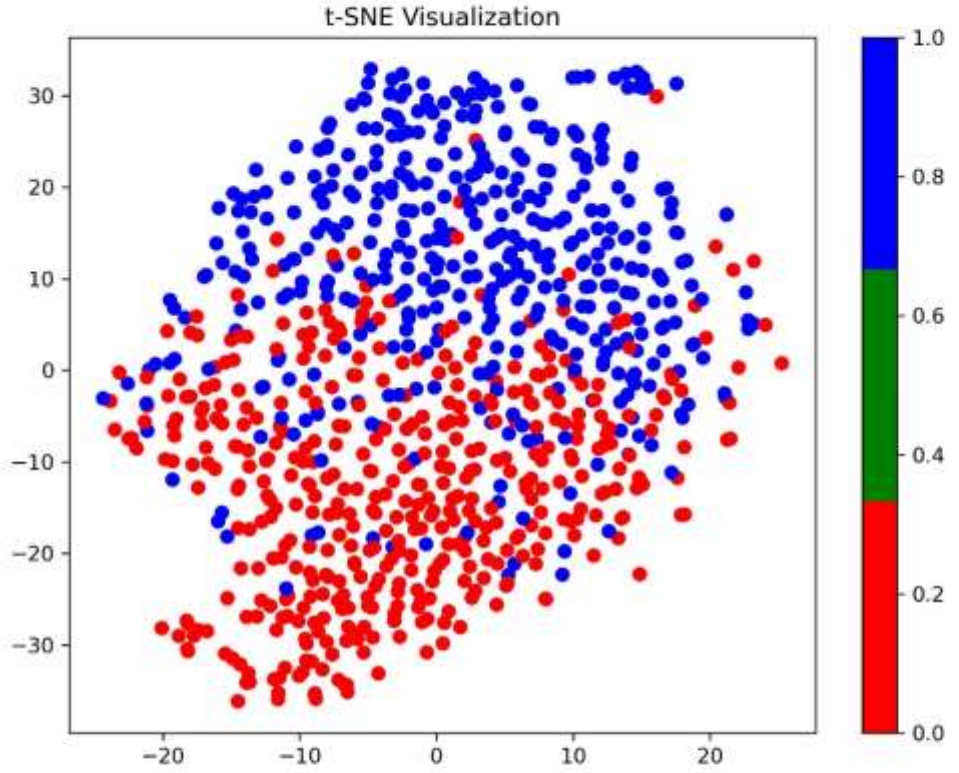

DBSCAN epsilon:

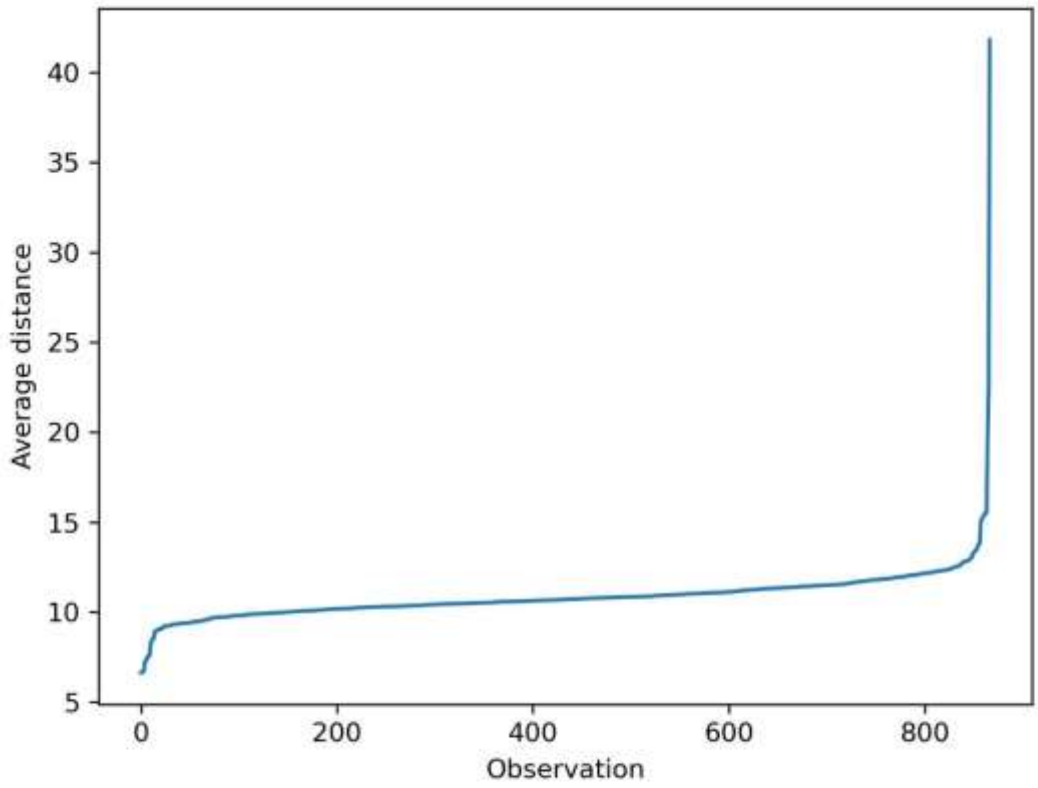

DBSCAN – best based on Davies Bouldin

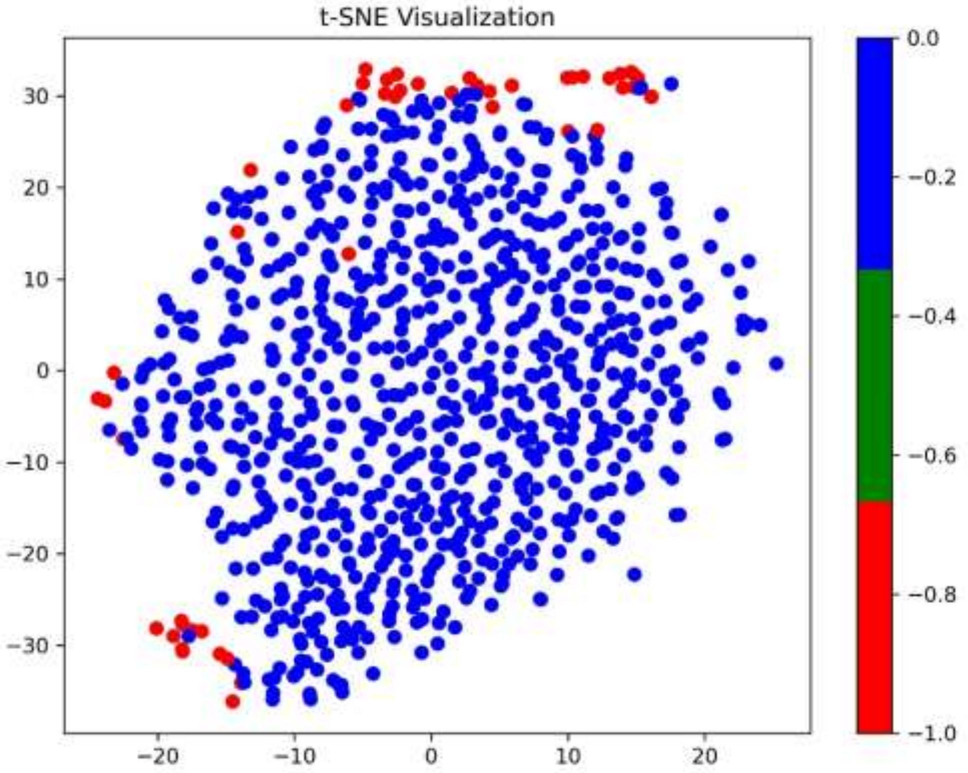

DBSCAN – Best based on Silhouette

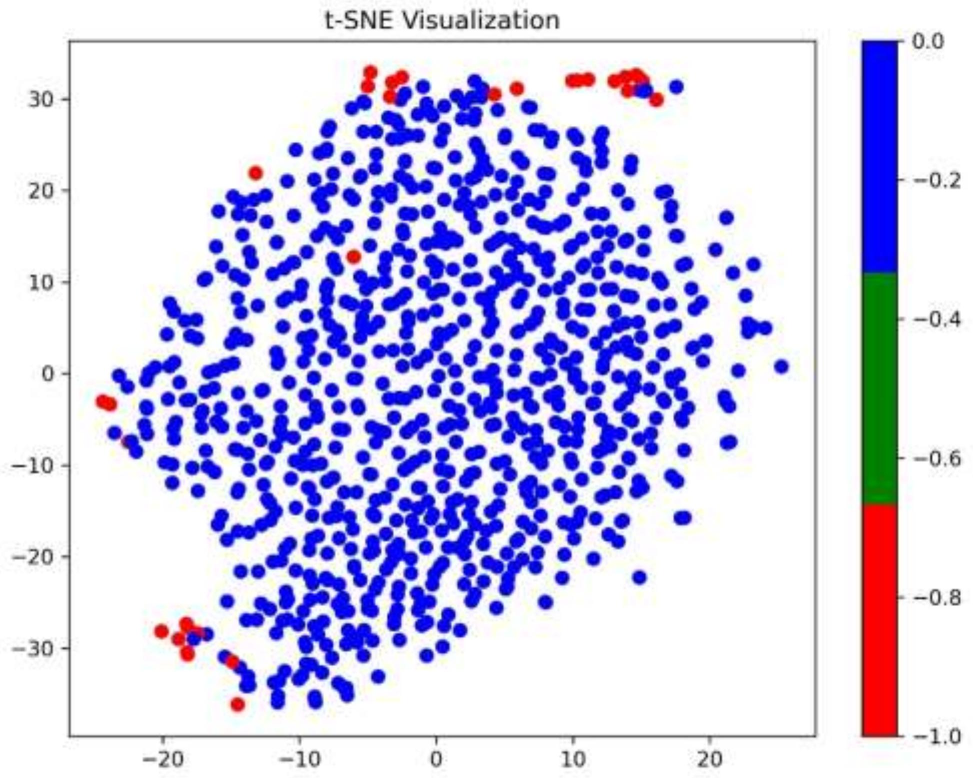

Best model – DBSCAN with Epsilon = 13.8, Min Points = 51.

Left Masseter Muscle

Data visualization:

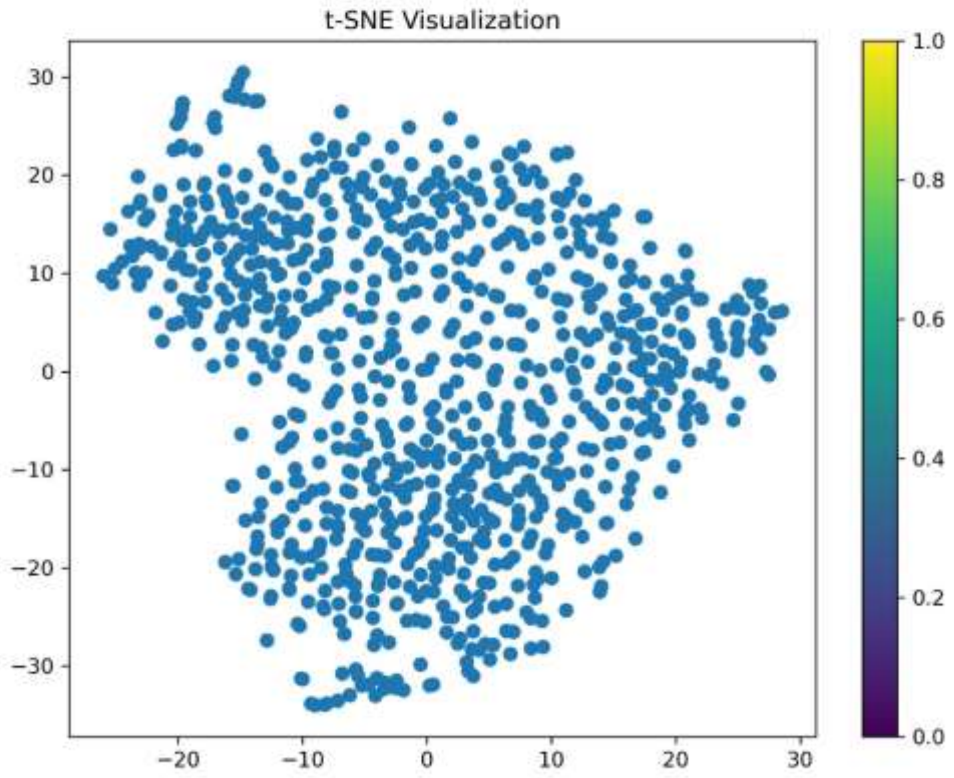

K-Means Elbow and Silhouette:

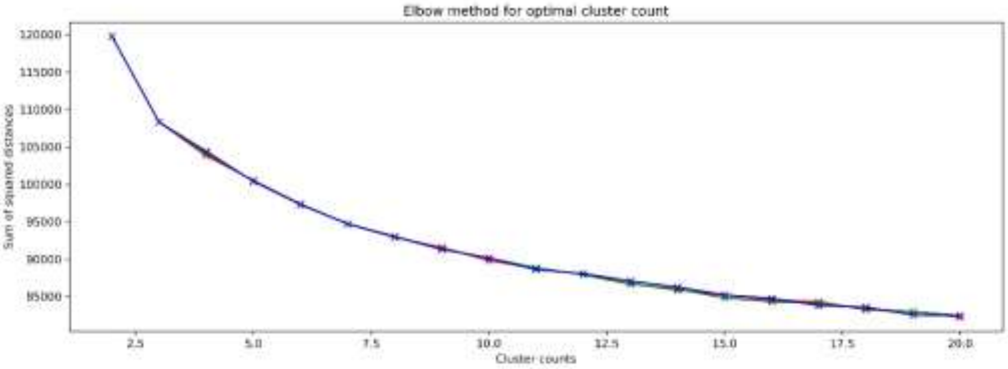

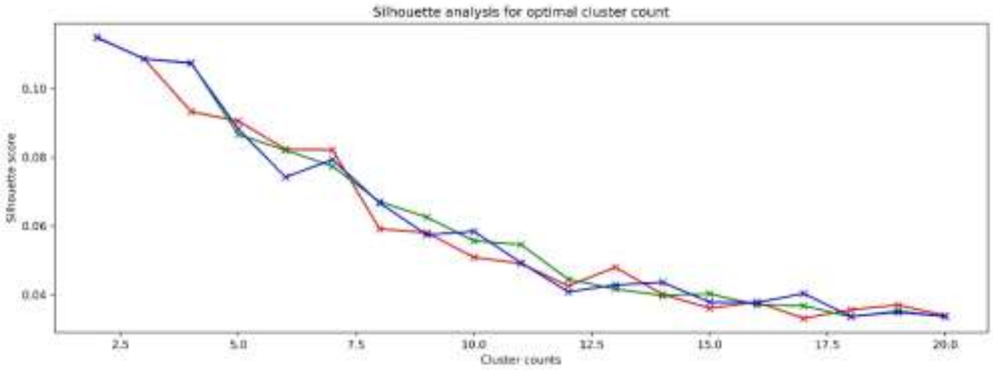

K-Means clustering:

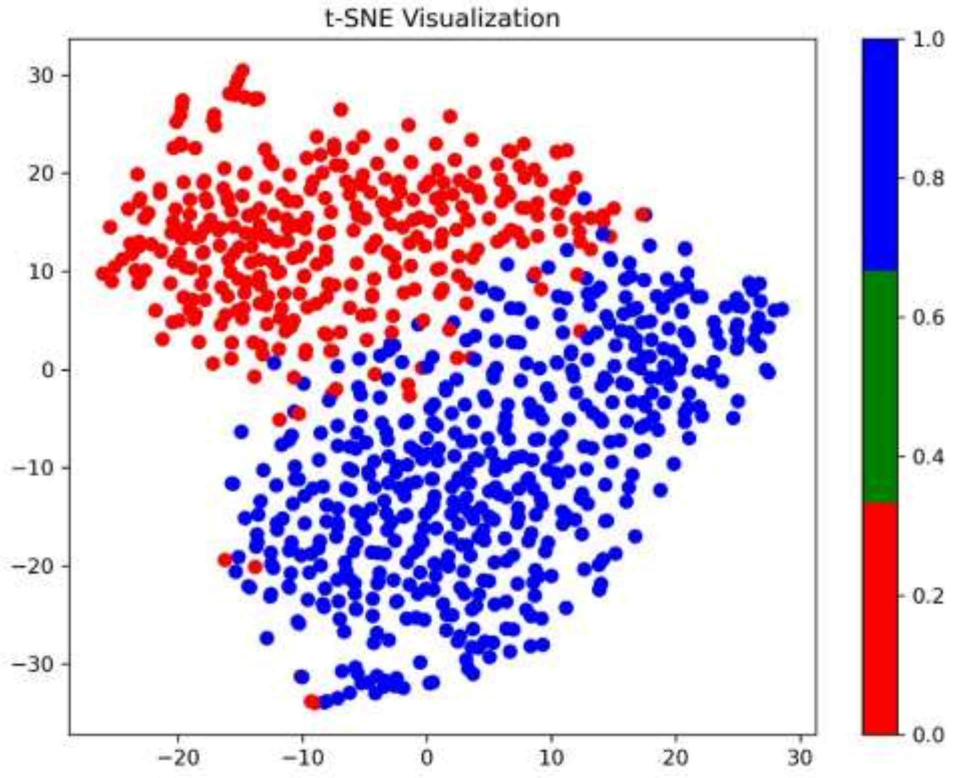

GMM Silhouette and BIC:

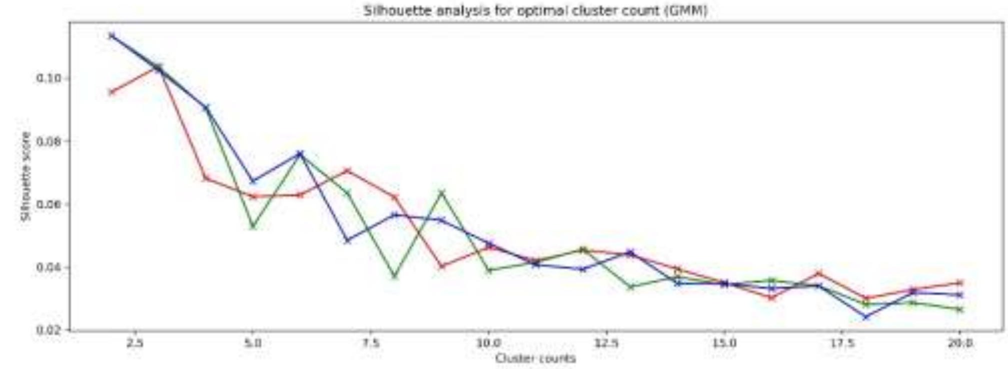

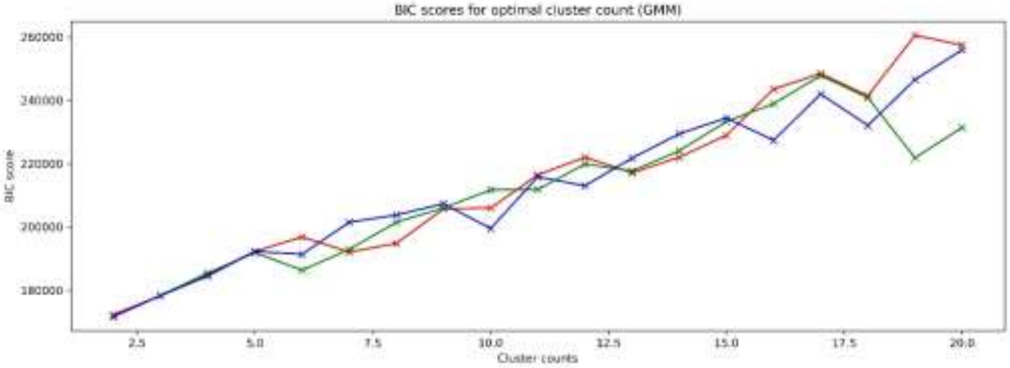

GMM Clustering:

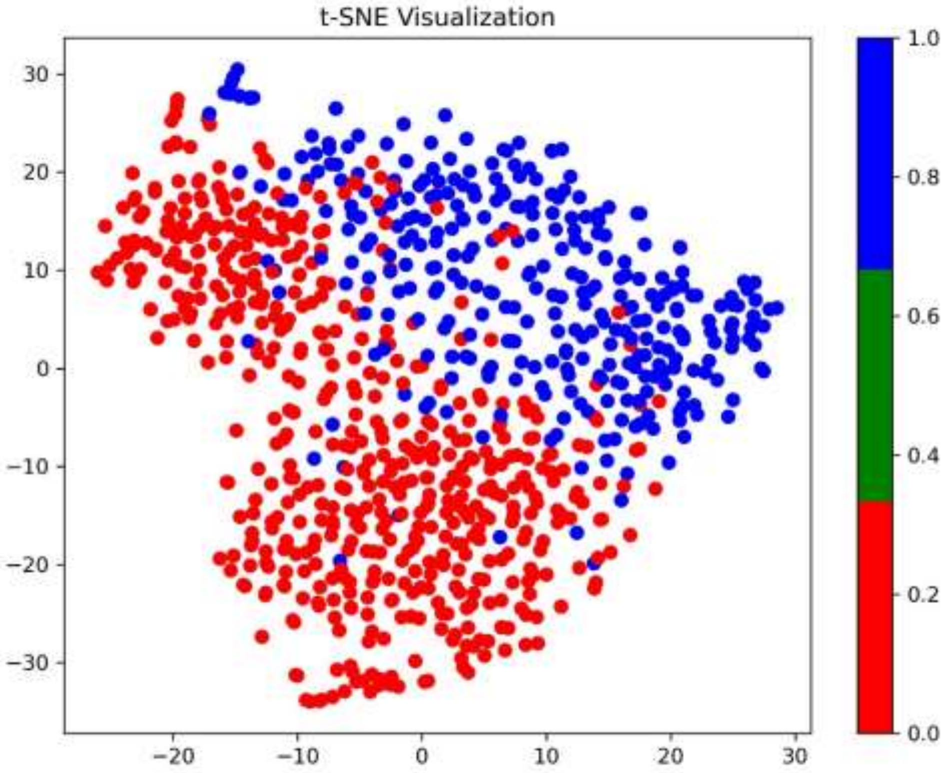

DBSCAN epsilon:

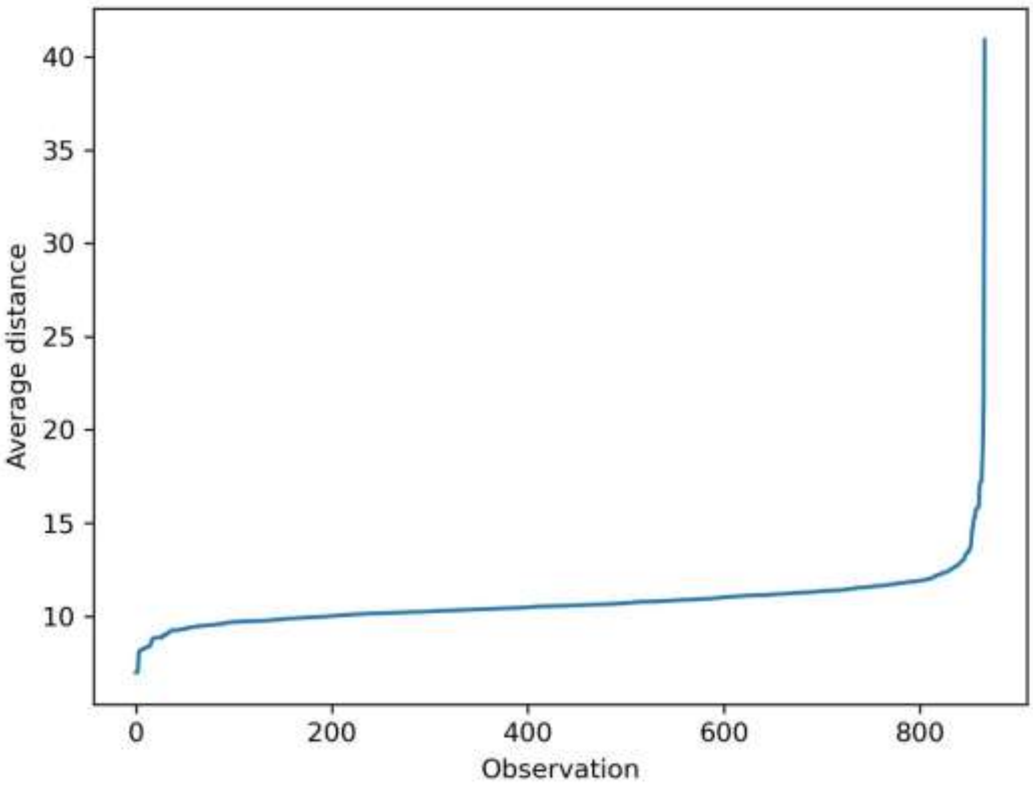

DBSCAN – best based on Davies Bouldin

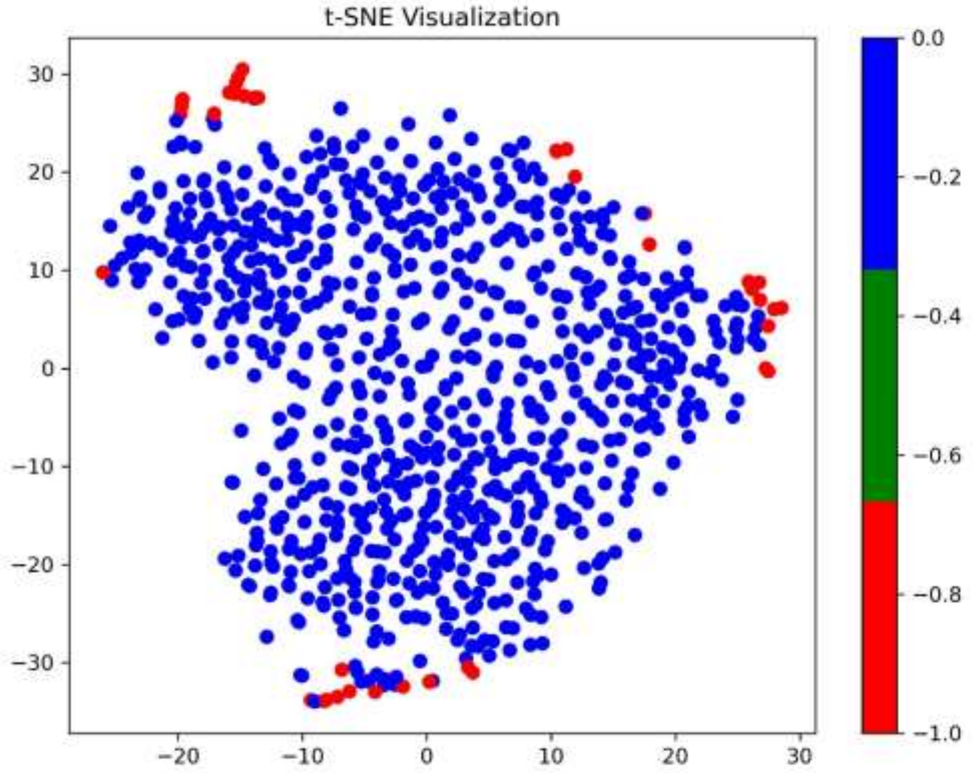

DBSCAN – best based on Silhouette:

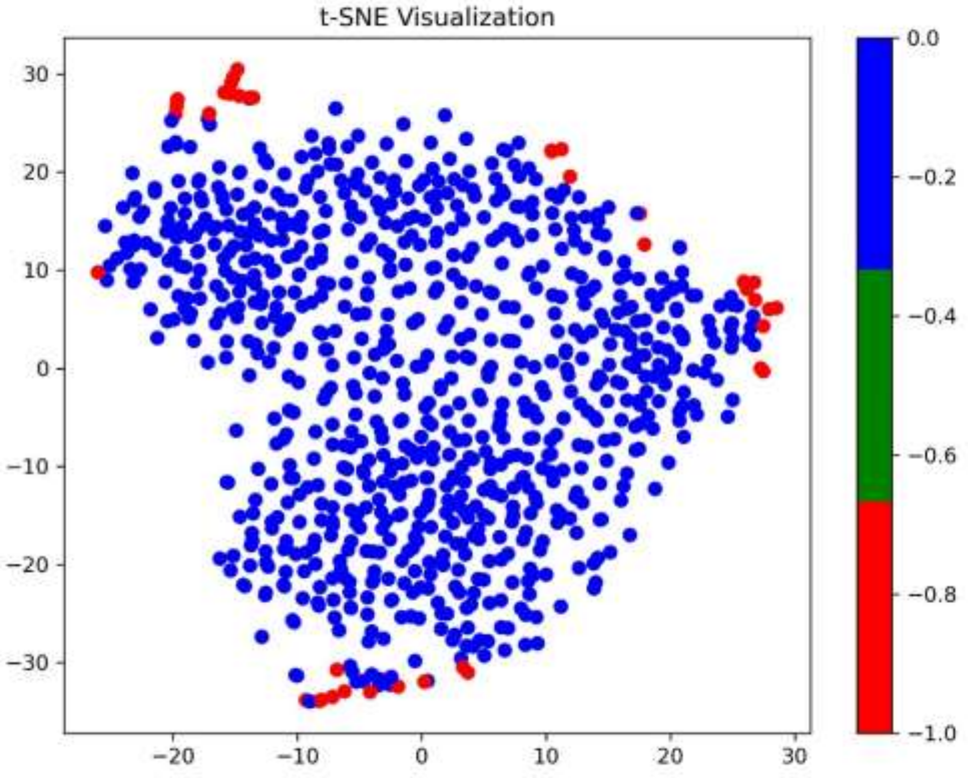

Best model – K-Means with k = 2.

Right Digastric Muscle

Data visualization:

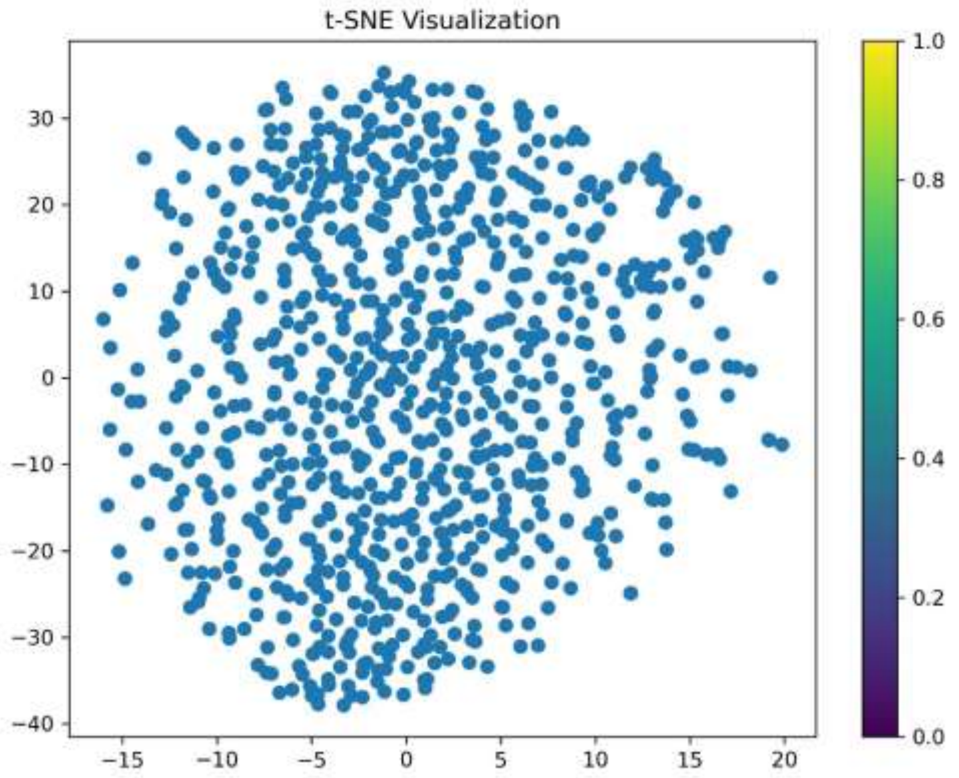

K-Means Elbow and Silhouette:

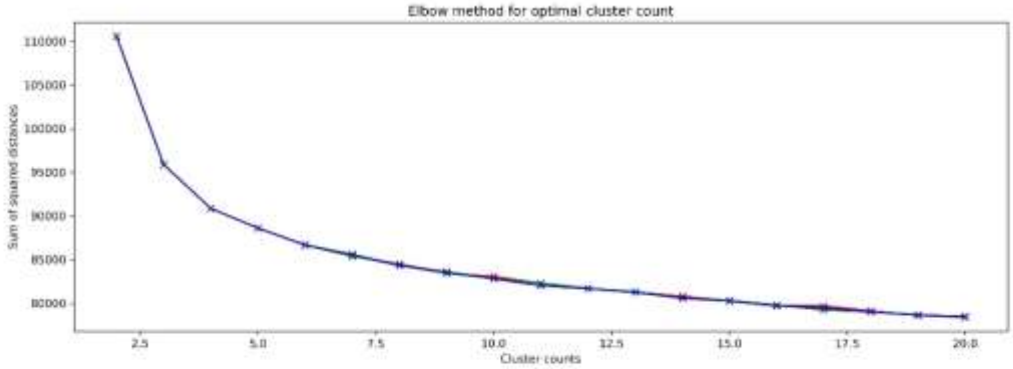

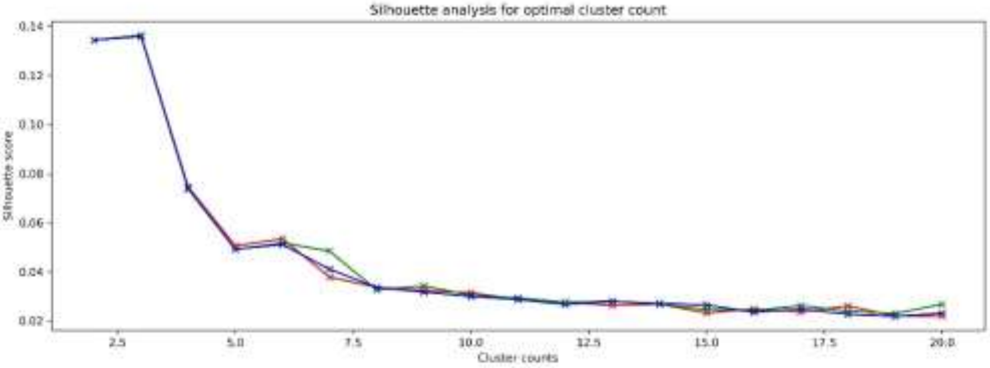

K-Means clustering:

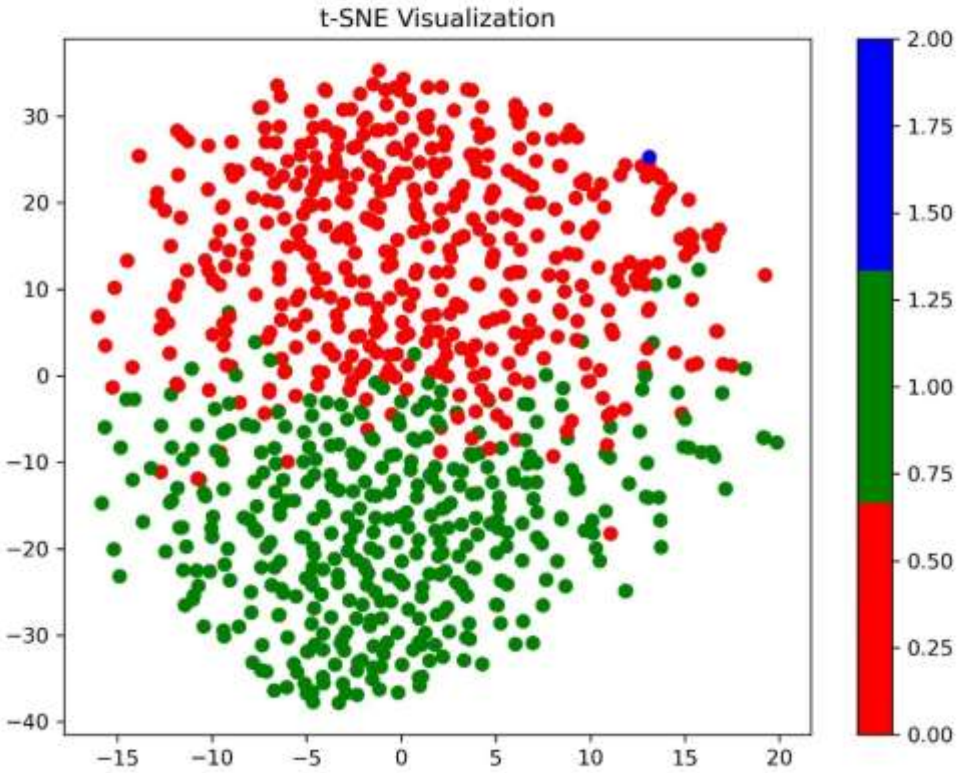

GMM Silhouette and BIC:

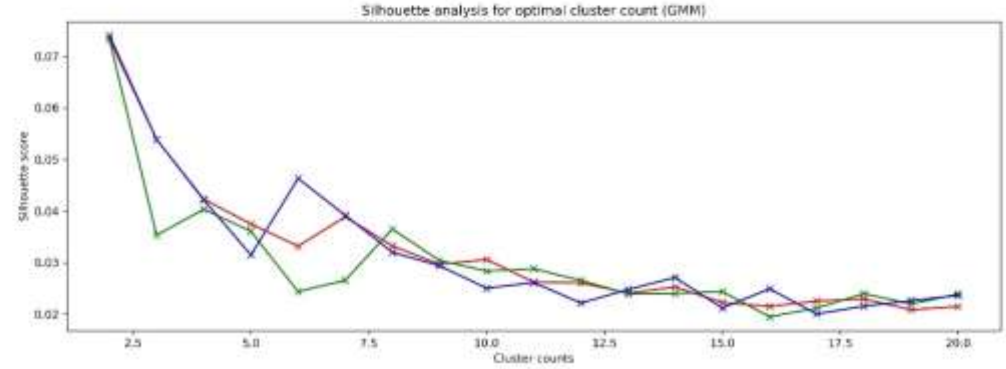

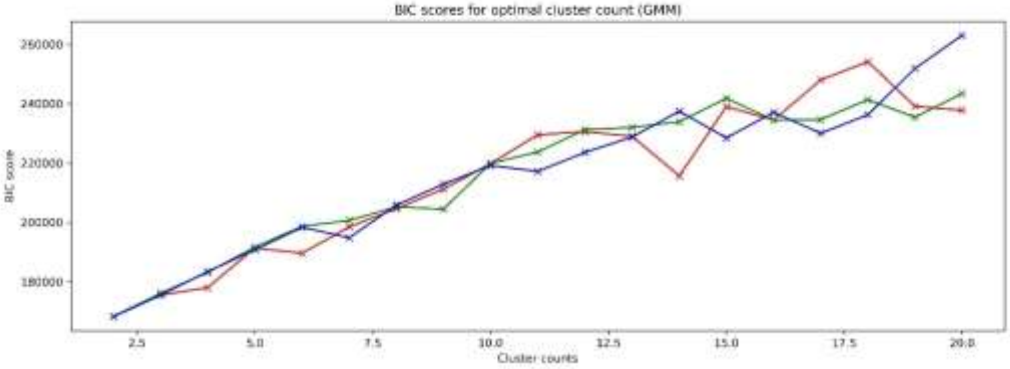

GMM Clustering:

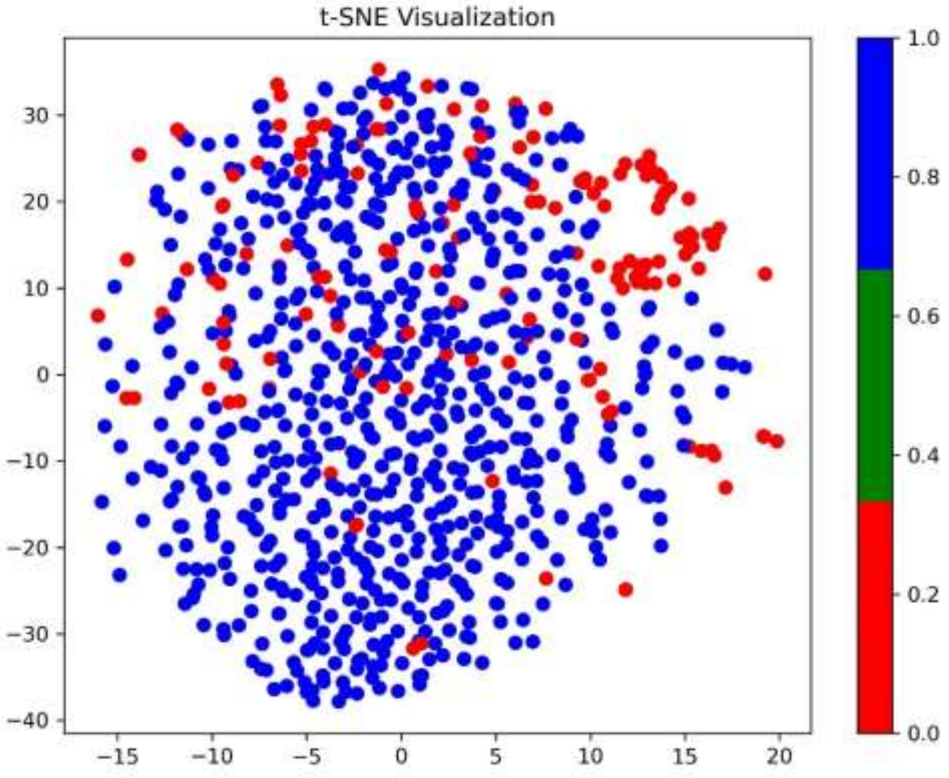

DBSCAN epsilon:

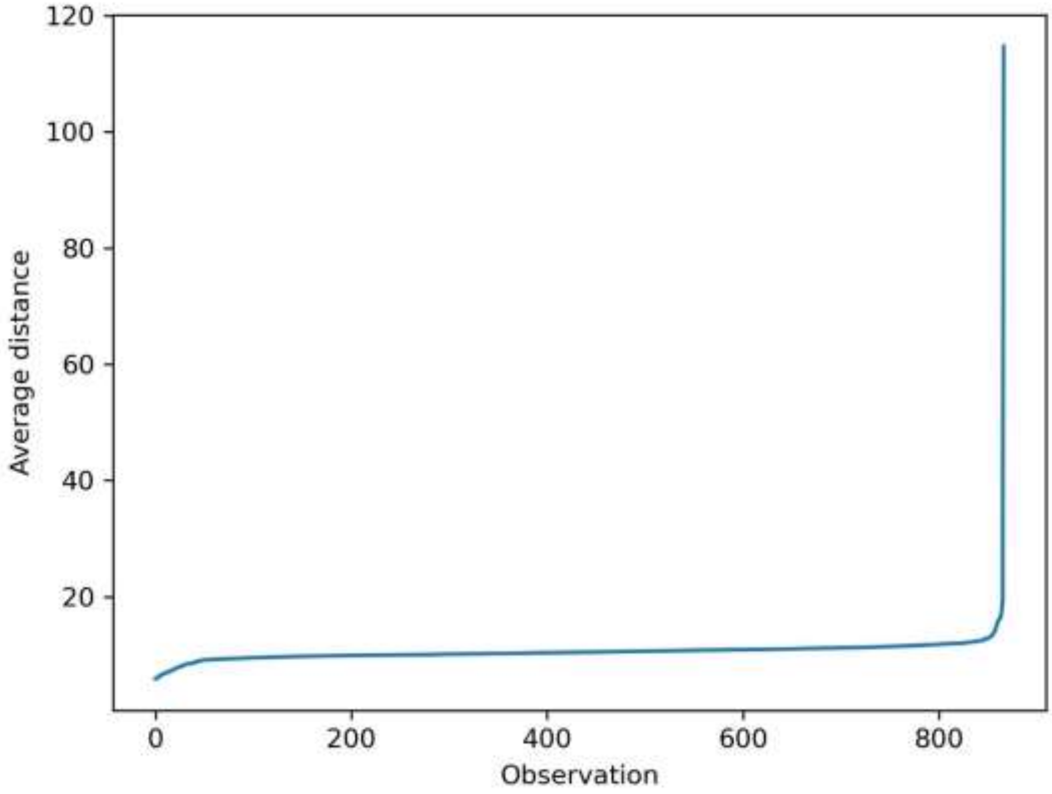

DBSCAN – best based on Davies Bouldin

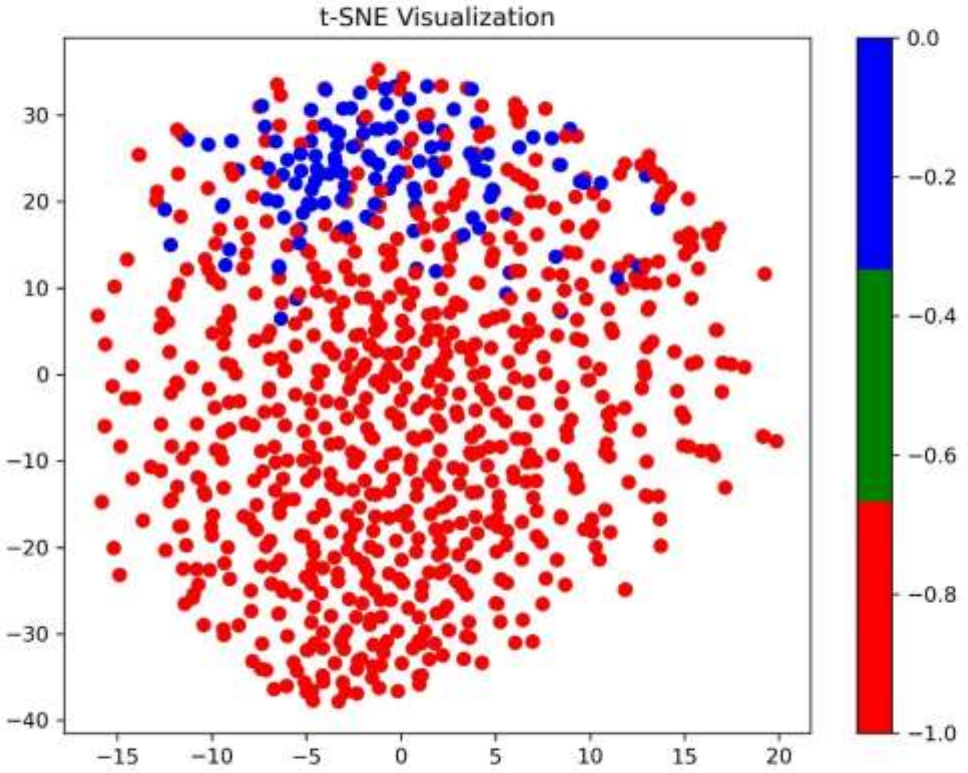

DBSCAN – Best based on Silhouette:

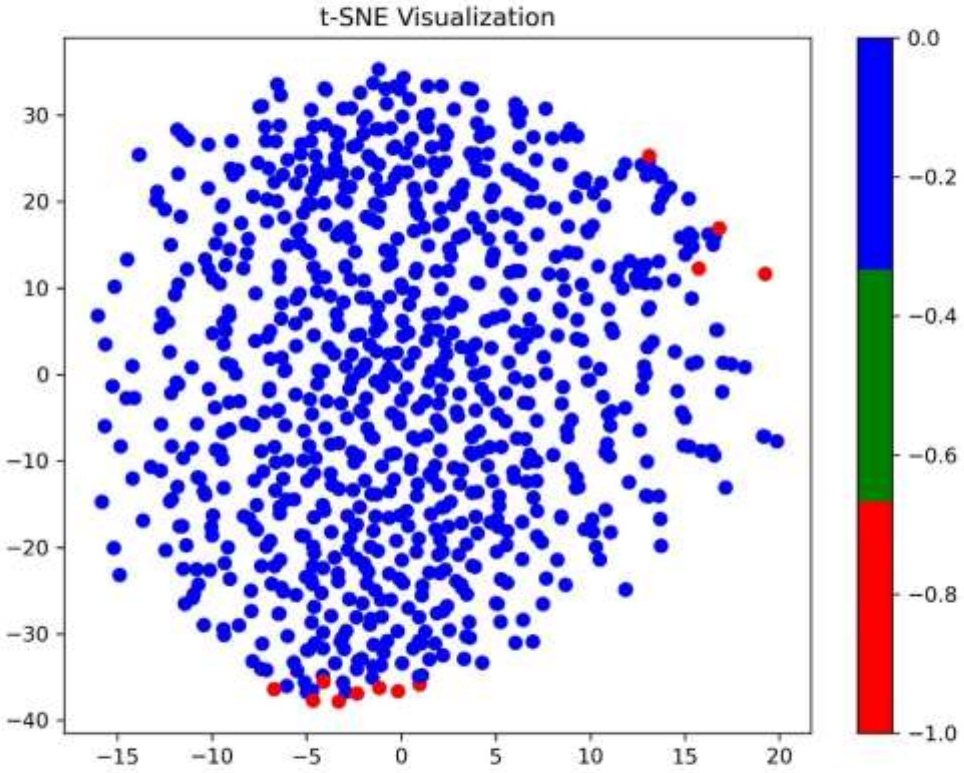

Best model – DBSCAN with Epsilon = 11, Min Points = 61.

Left Digastric Muscle

Data visualization:

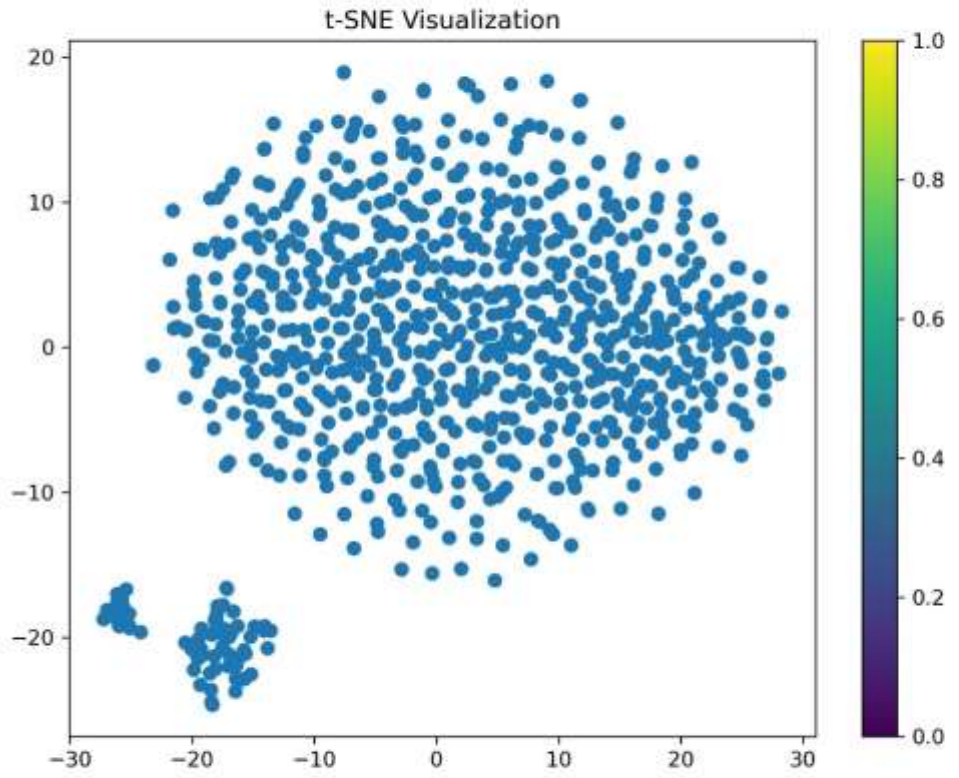

K-Means Elbow and Silhouette:

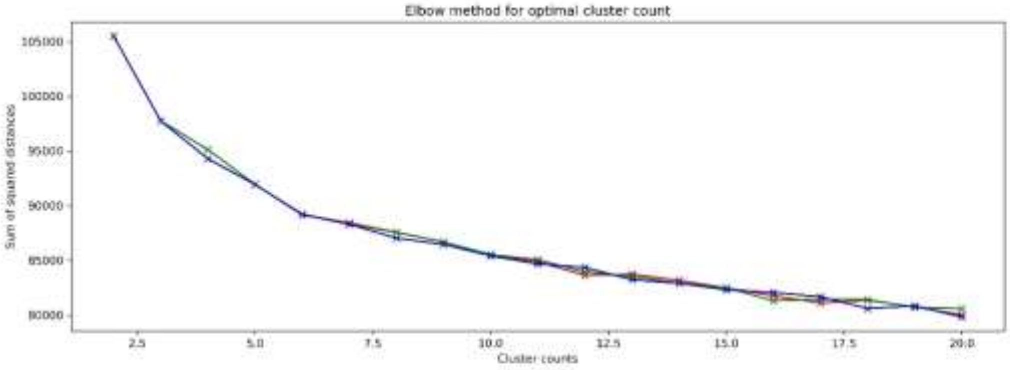

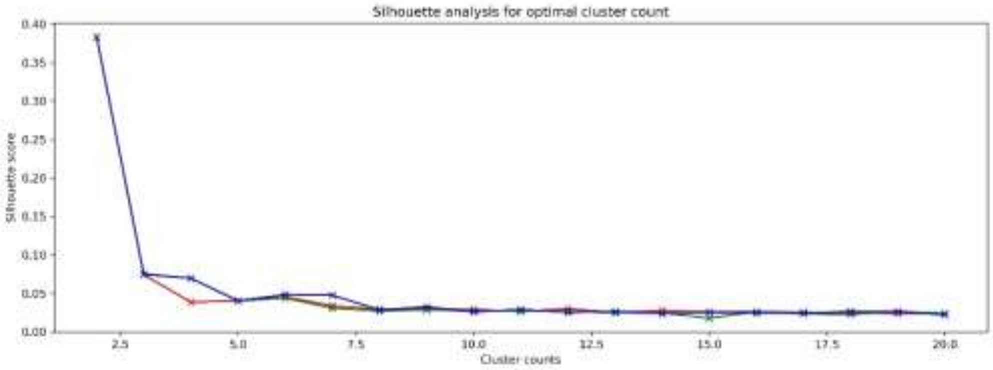

K-Means clustering:

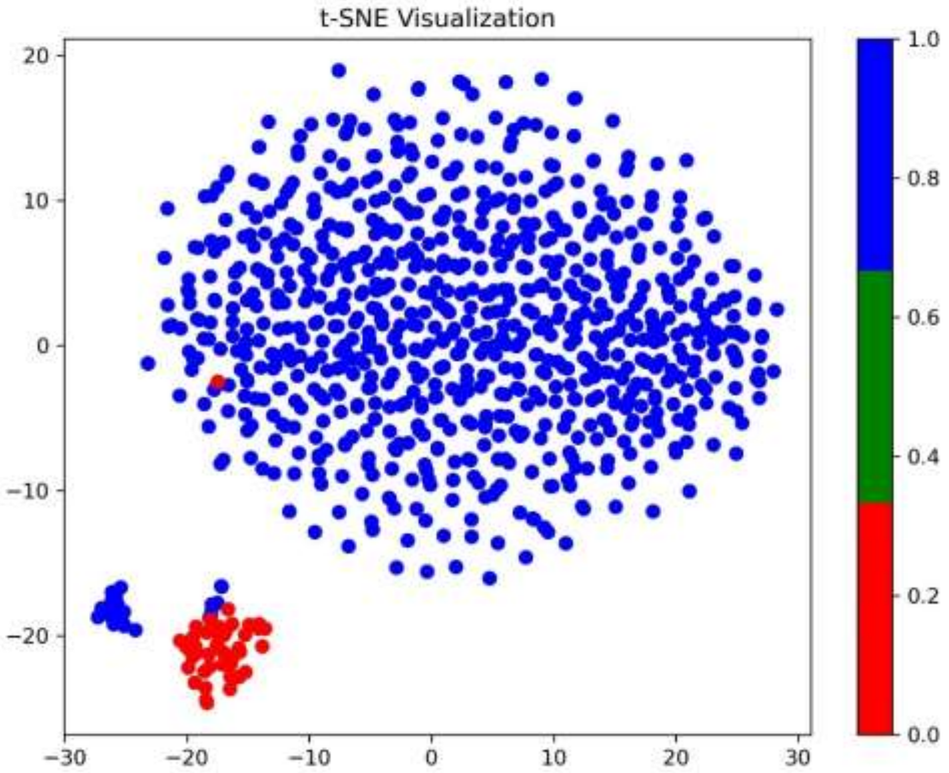

GMM Silhouette and BIC:

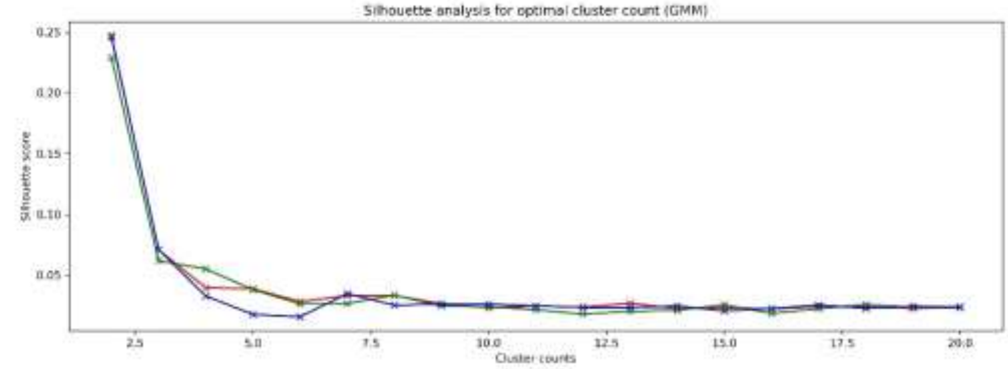

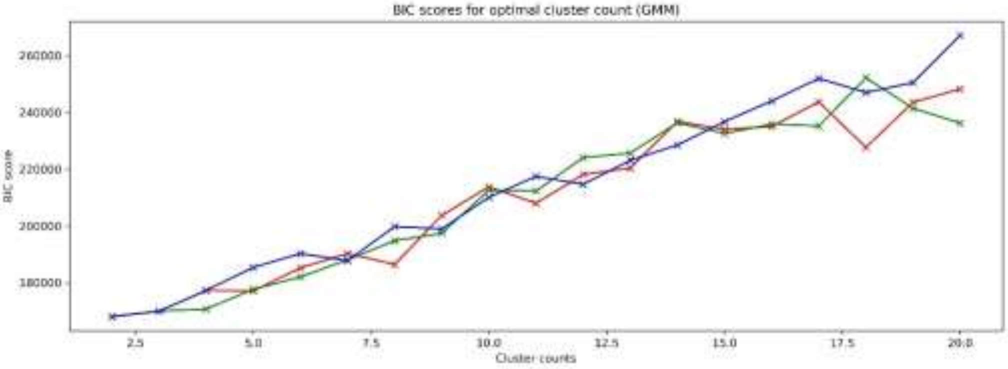

GMM Clustering:

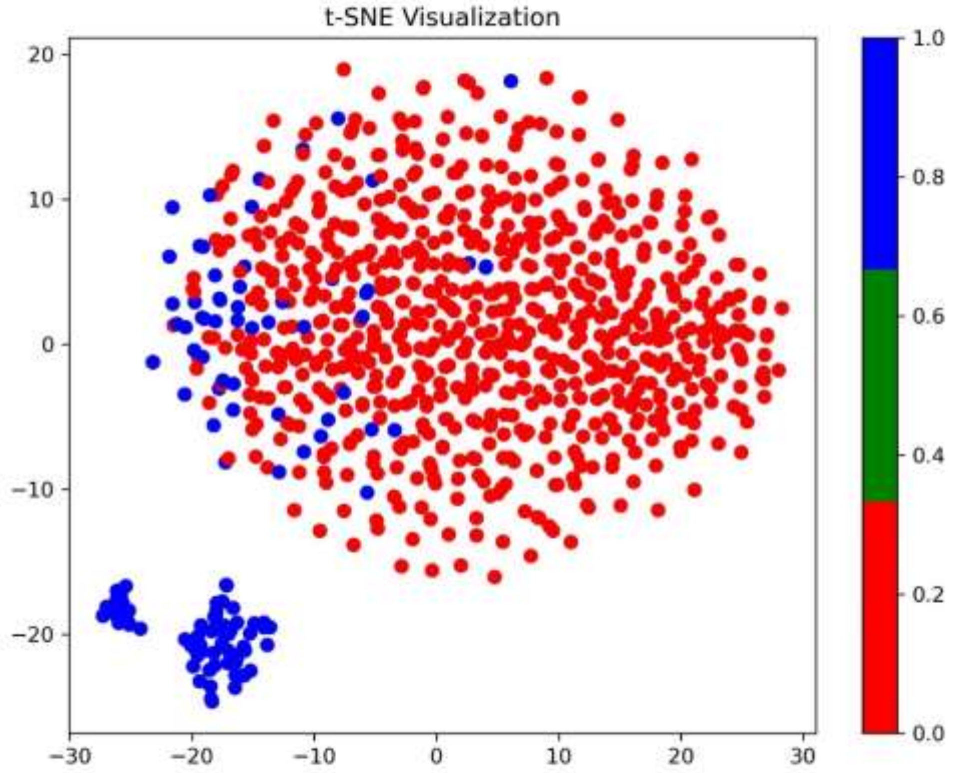

DBSCAN epsilon:

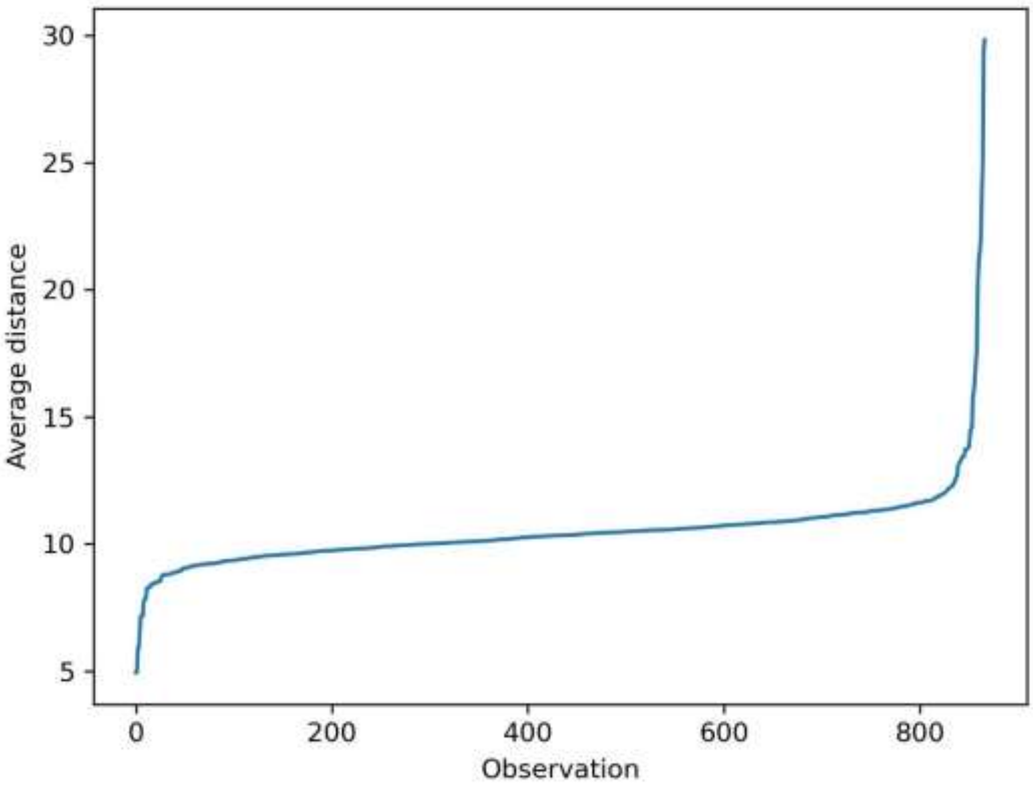

DBSCAN – best based on Davies Bouldin

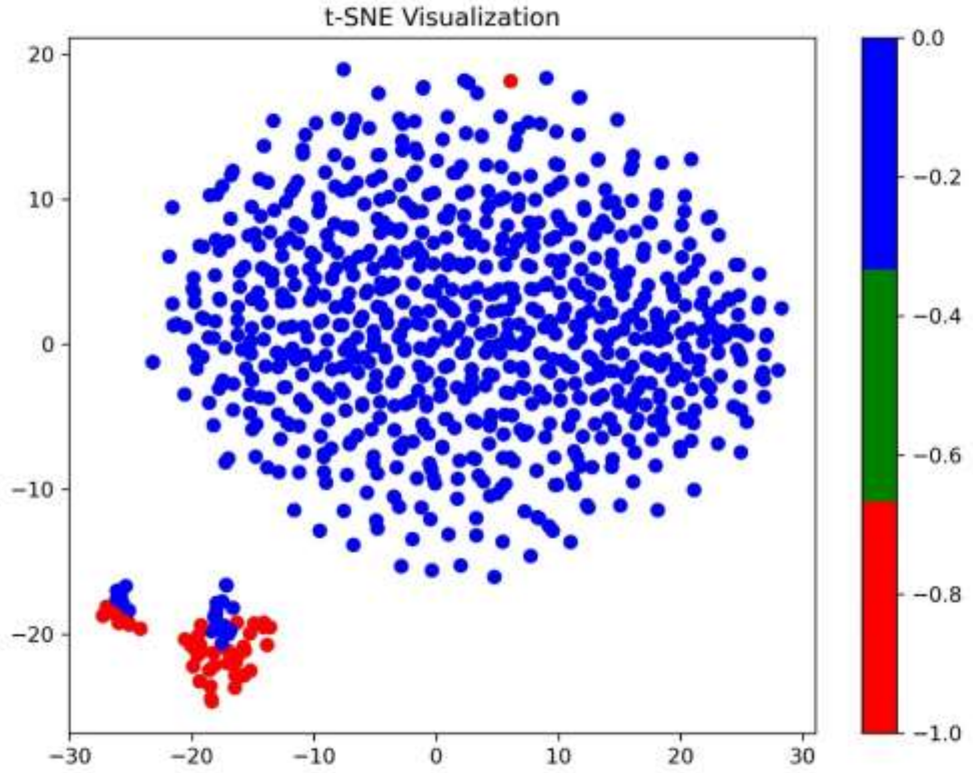

DBSCAN – Best based on Silhouette:

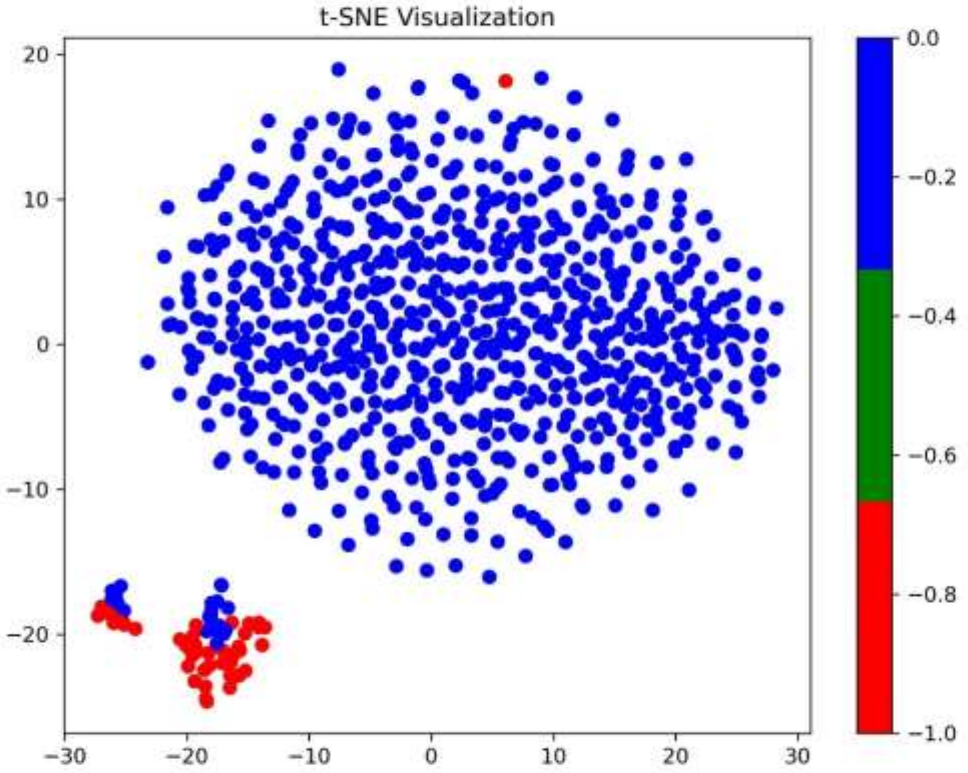

Best model – DBSCAN with Epsilon = 13.8, Min Points = 91.

Maximum Lateral Excursion

Right Temporalis

Data visualization:

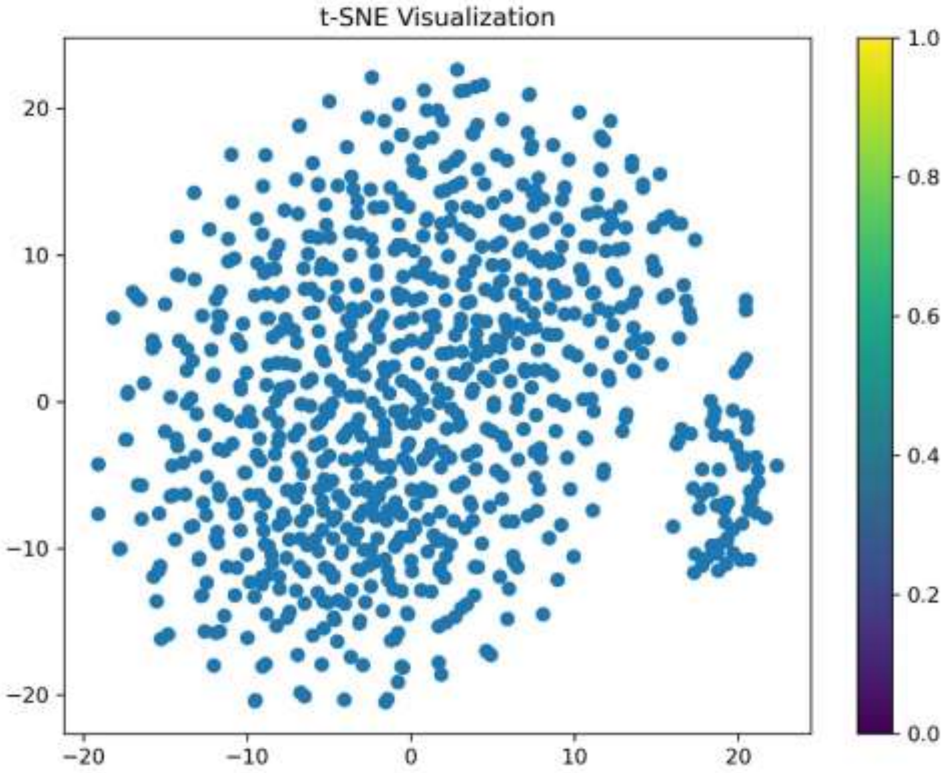

K-Means Elbow and Silhouette:

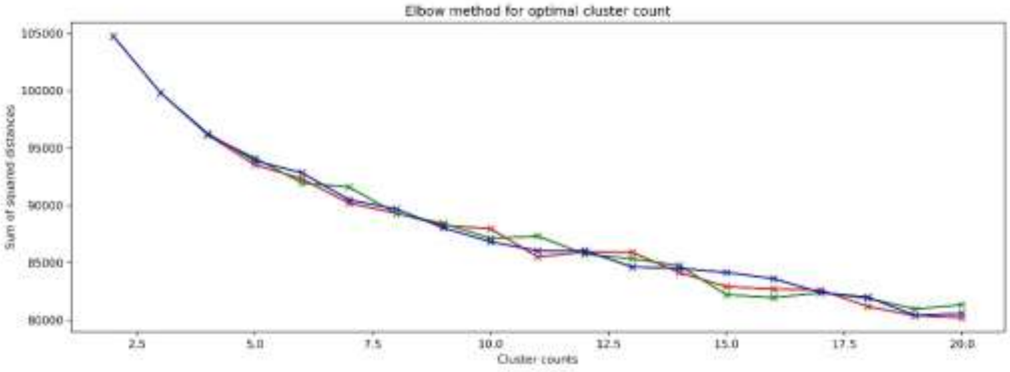

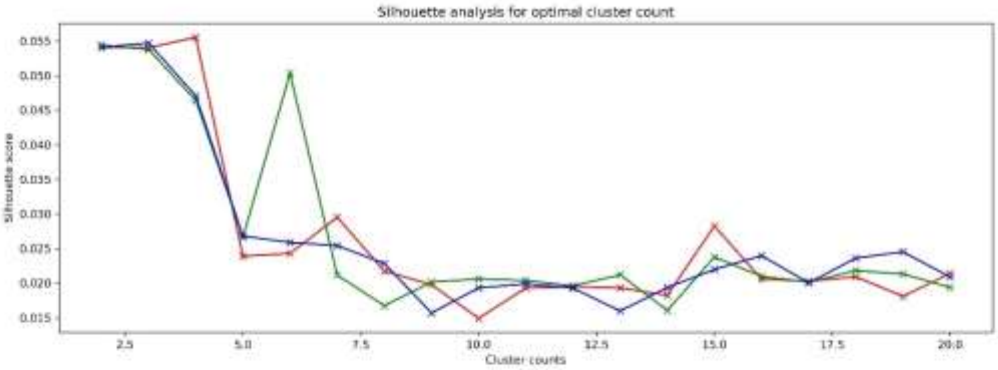

K-Means clustering:

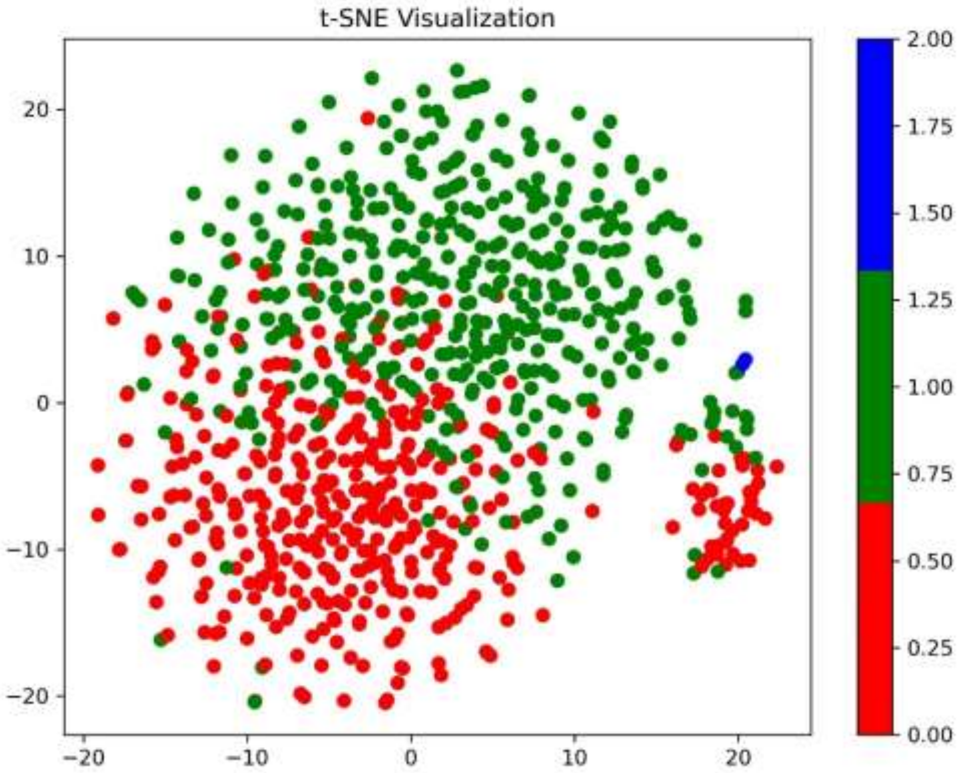

GMM Silhouette and BIC:

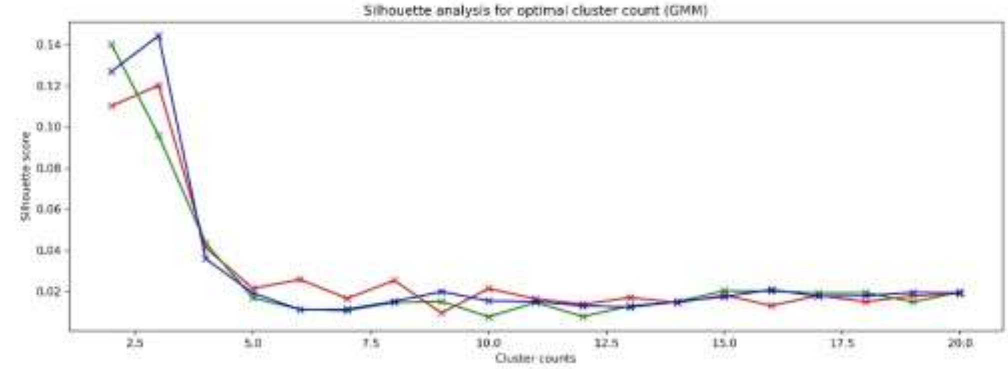

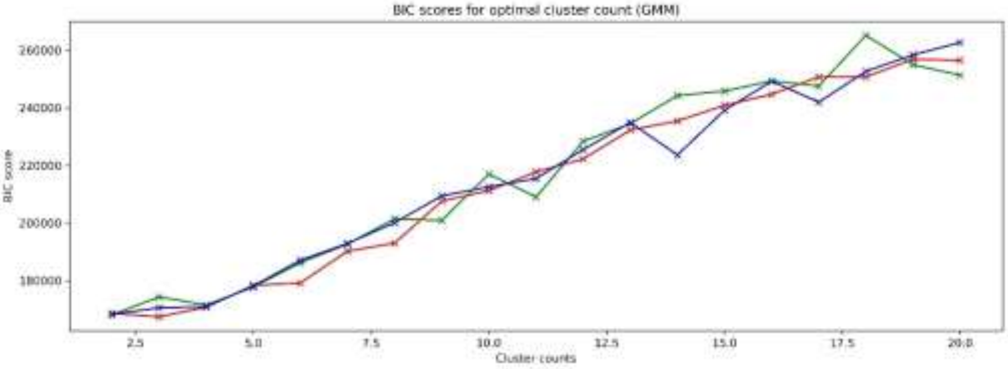

GMM Clustering:

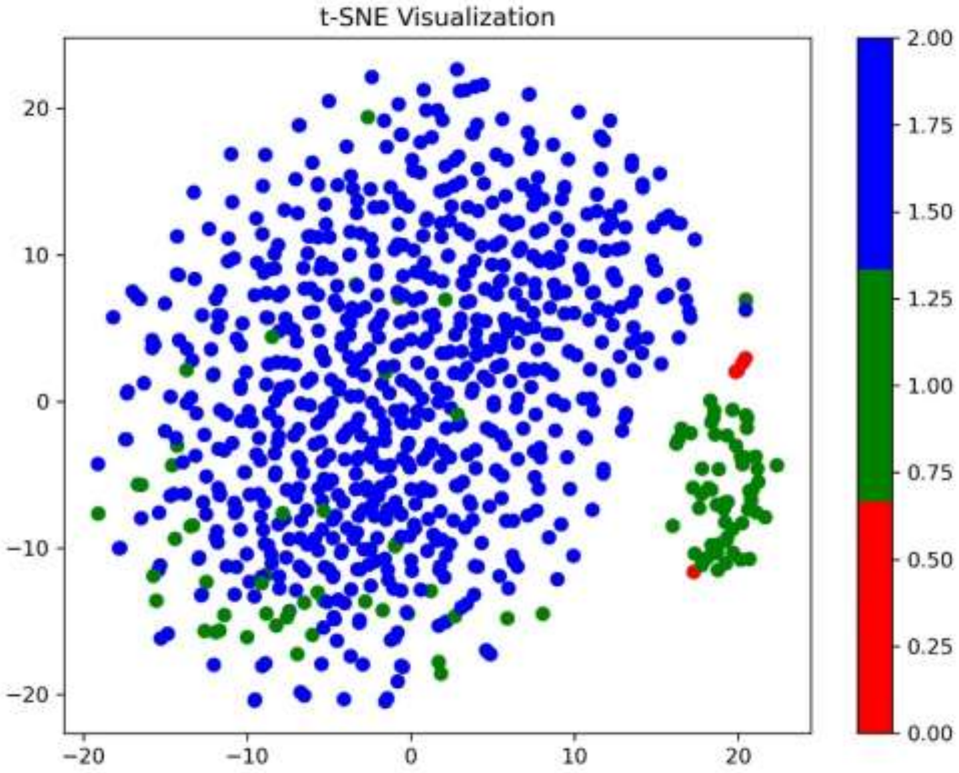

DBSCAN epsilon:

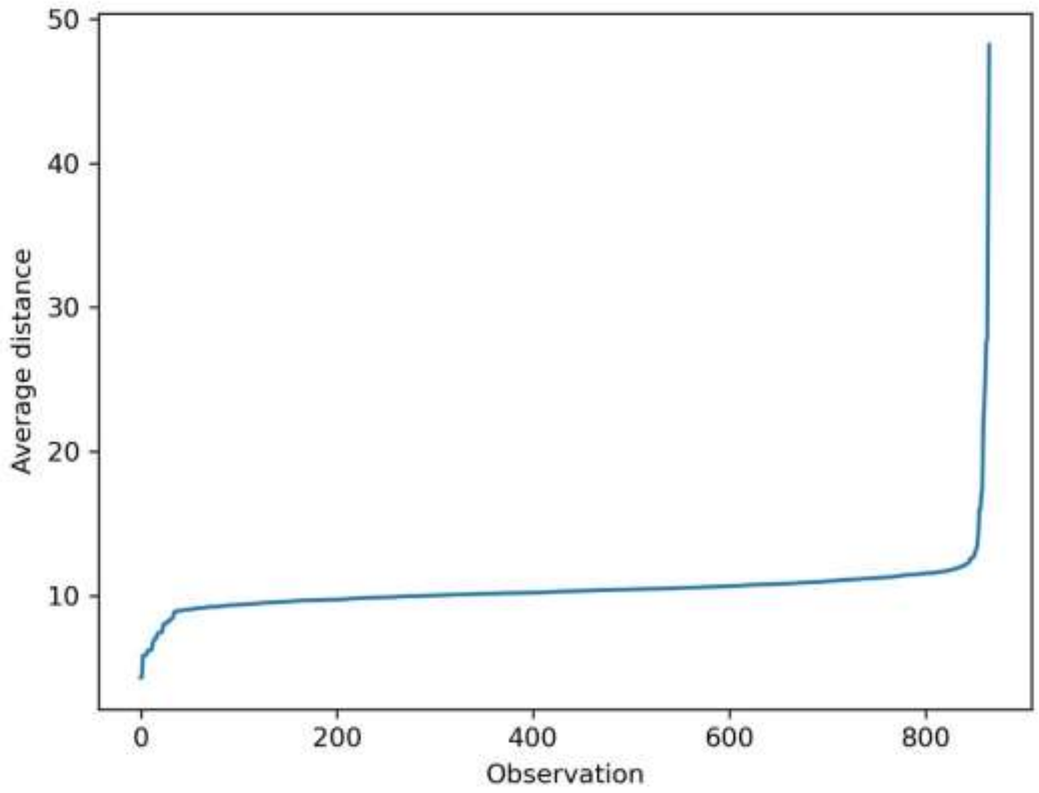

DBSCAN – best based on Davies Bouldin

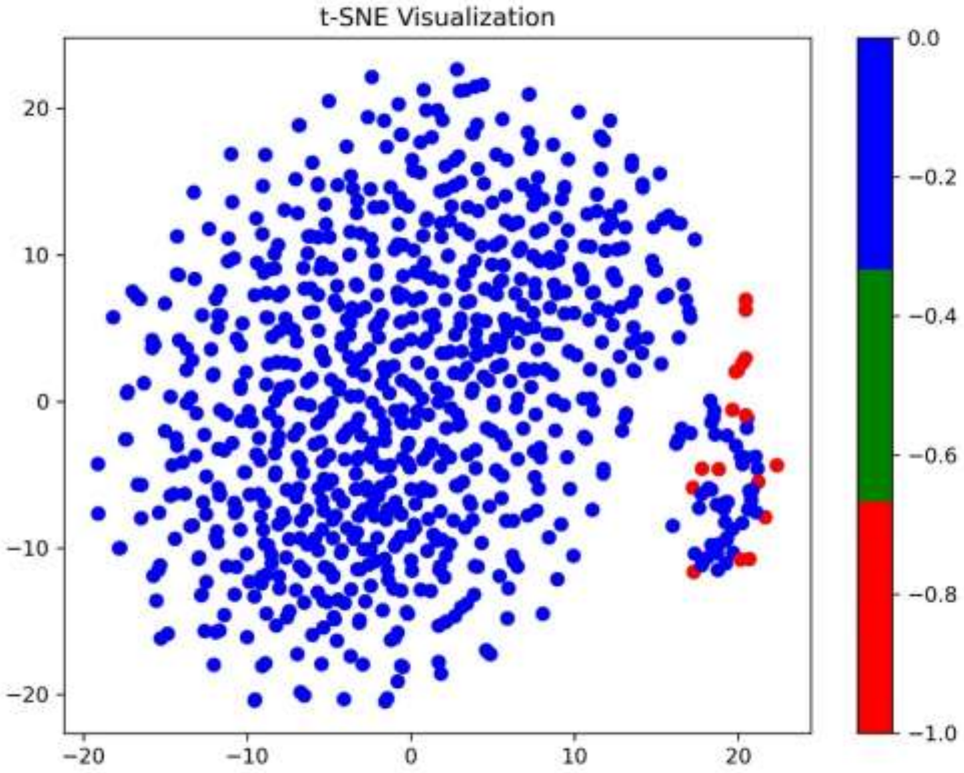

DBSCAN – Best based on Silhouette:

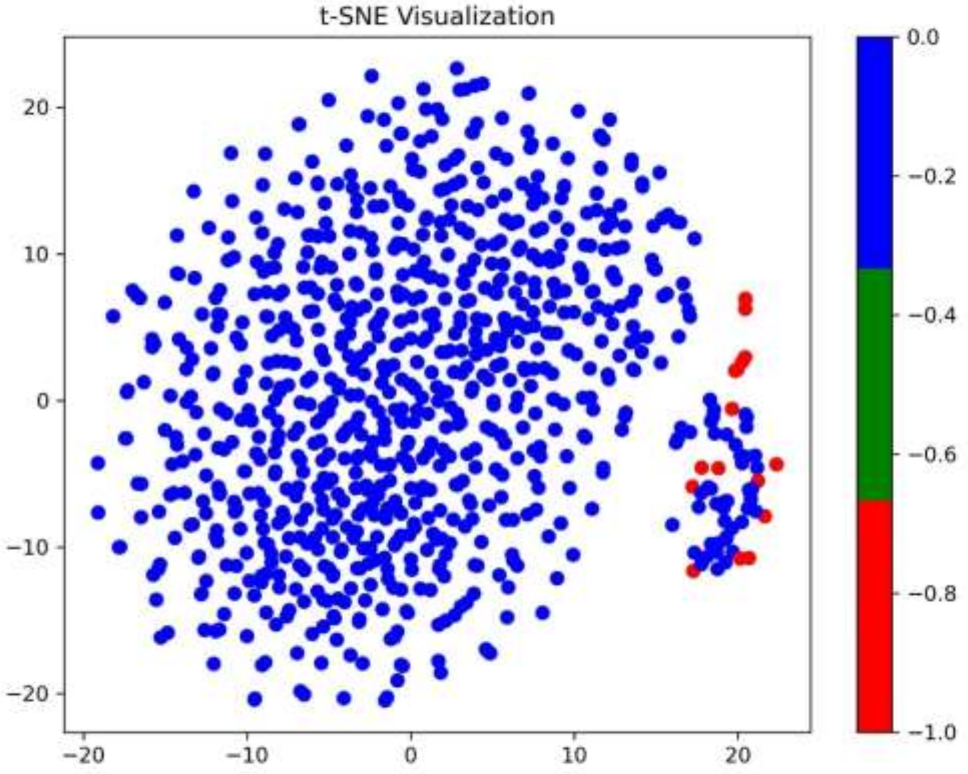

Best model – GMM with k = 3

Left Temporalis

Data visualization:

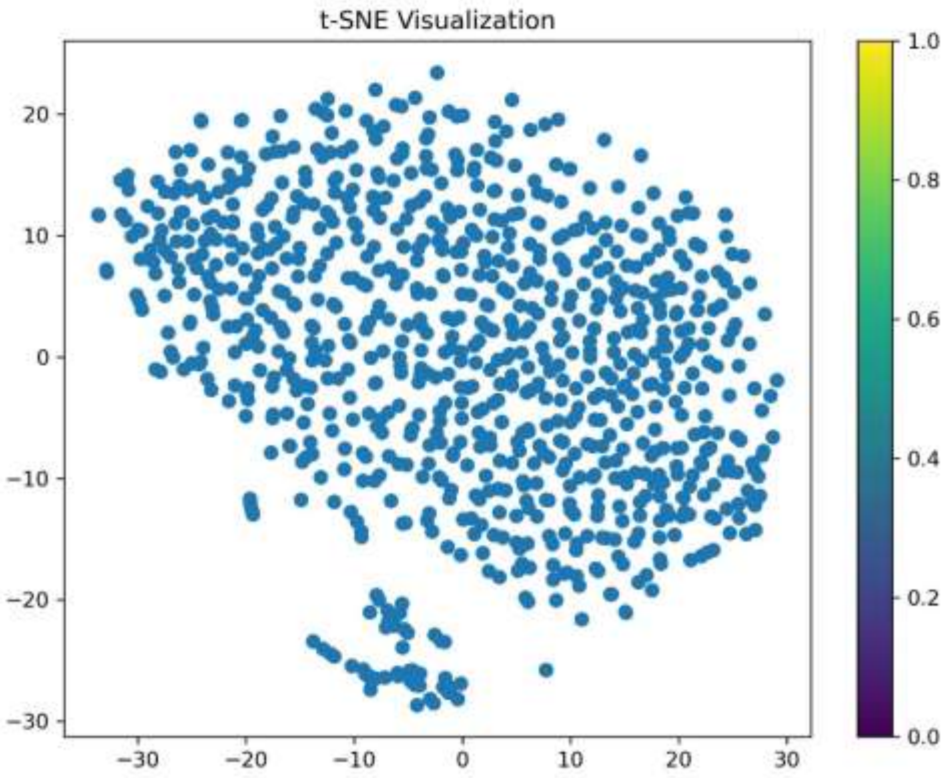

K-Means Elbow and Silhouette:

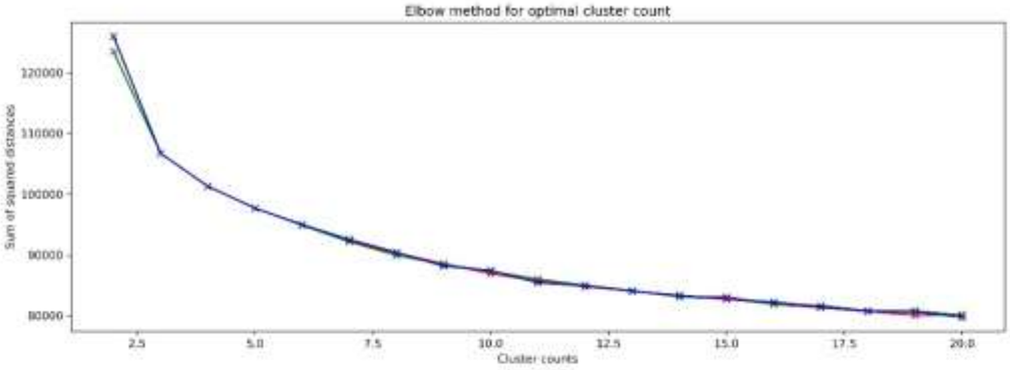

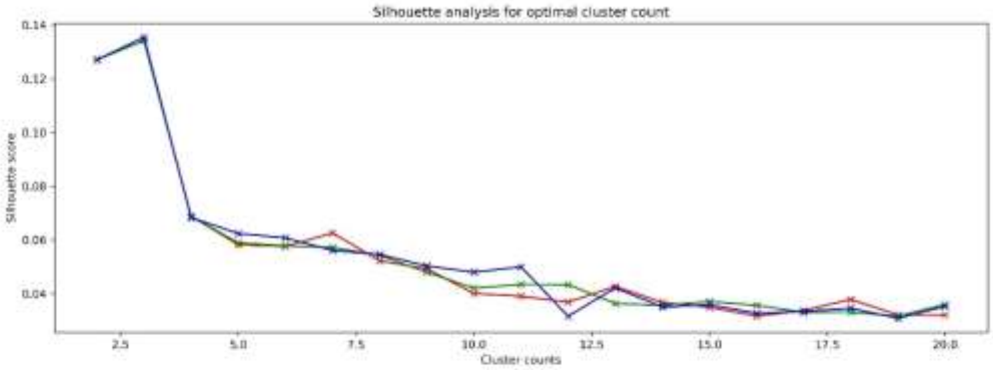

K-Means clustering:

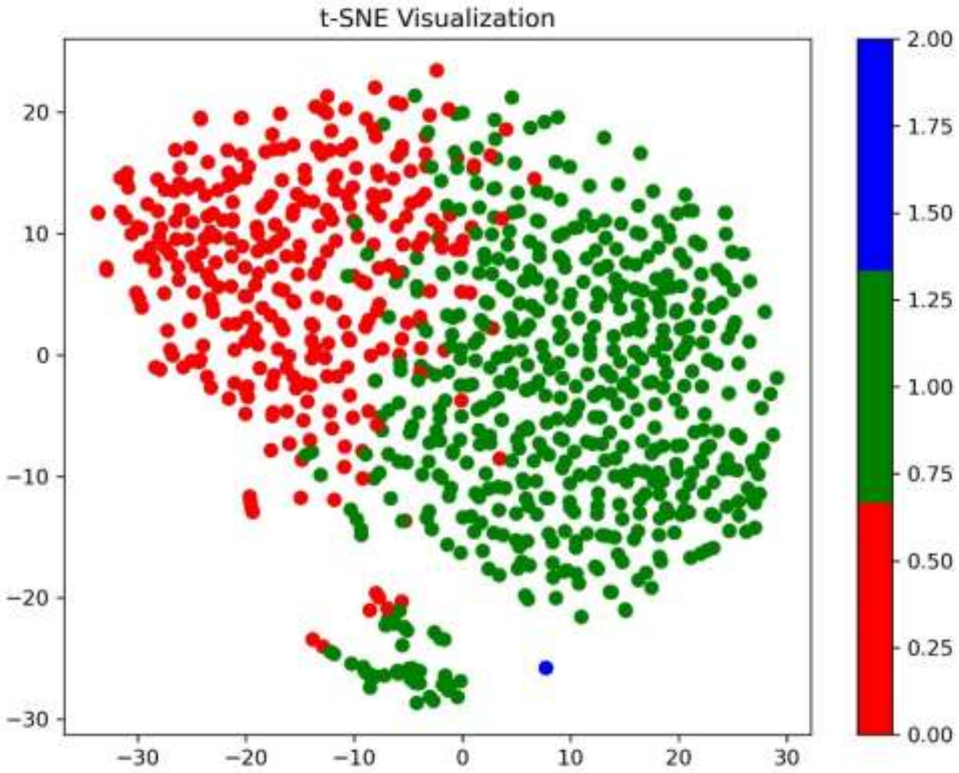

GMM Silhouette and BIC:

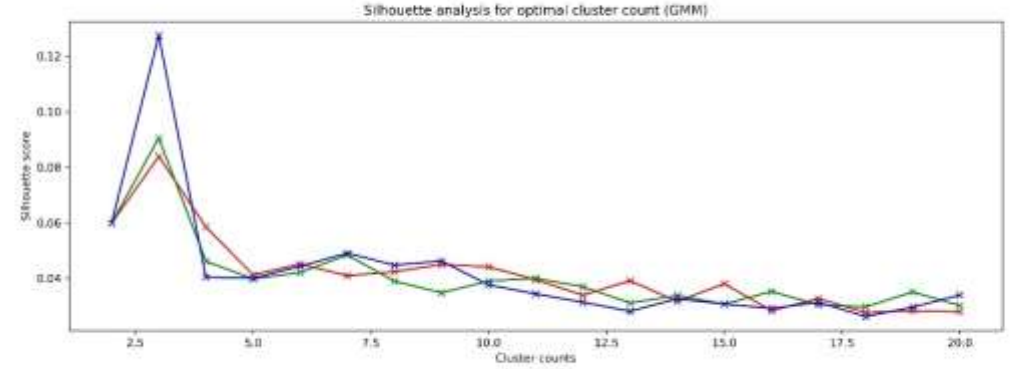

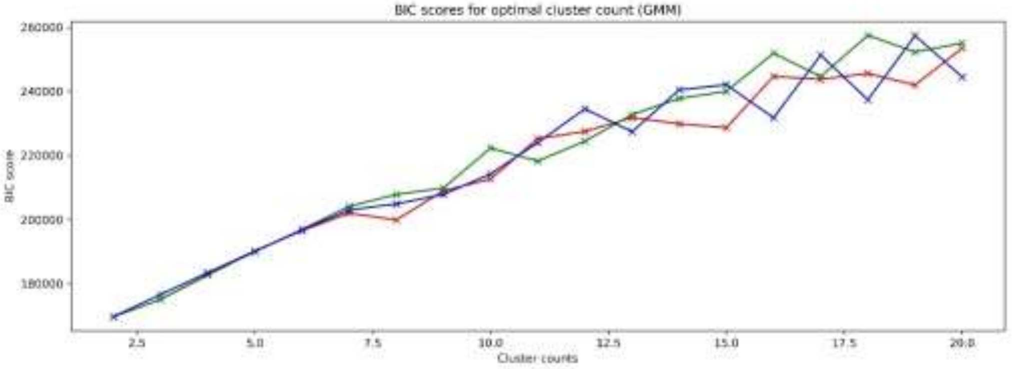

GMM Clustering:

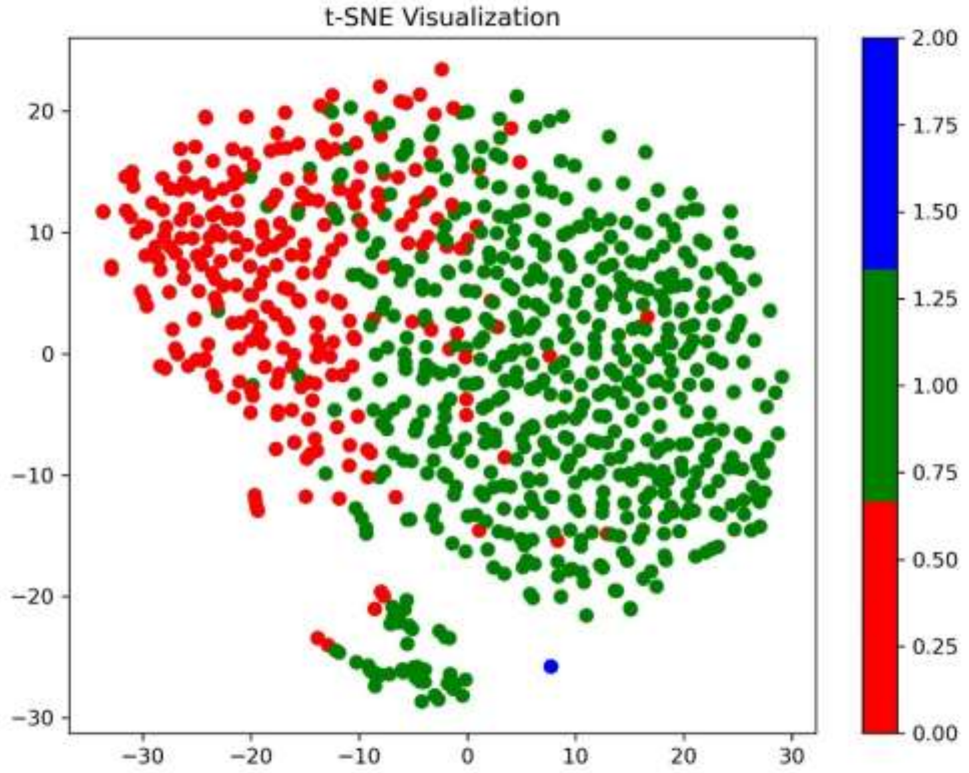

DBSCAN epsilon:

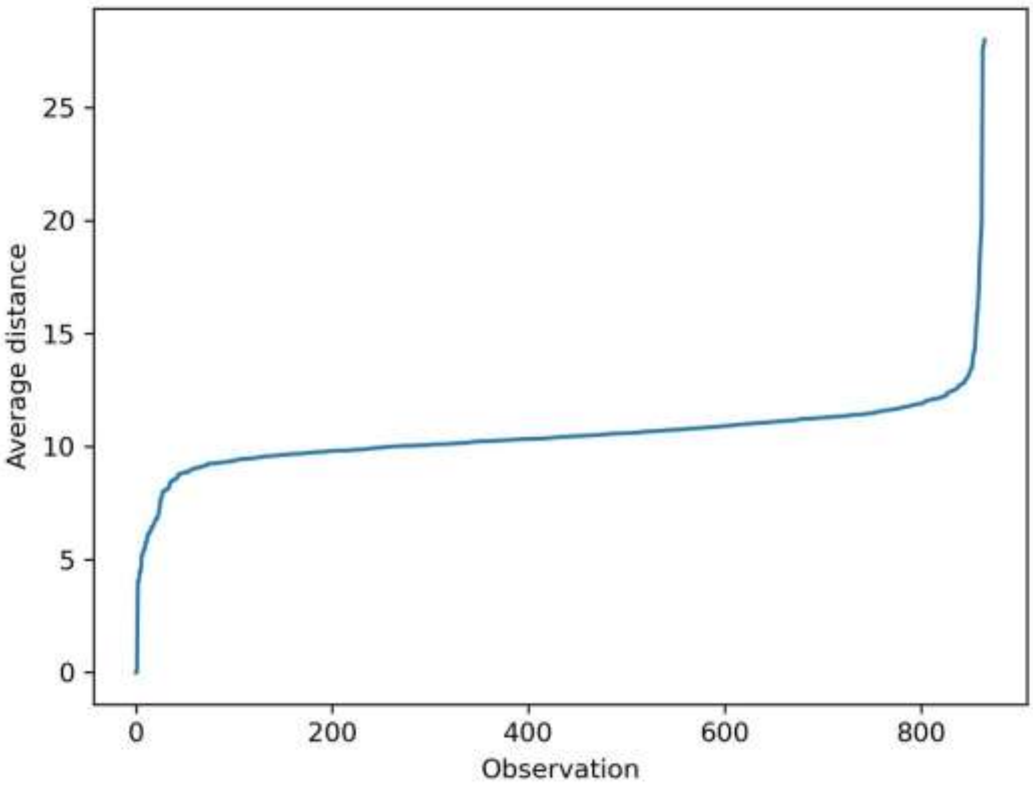

DBSCAN – best based on Davies Bouldin

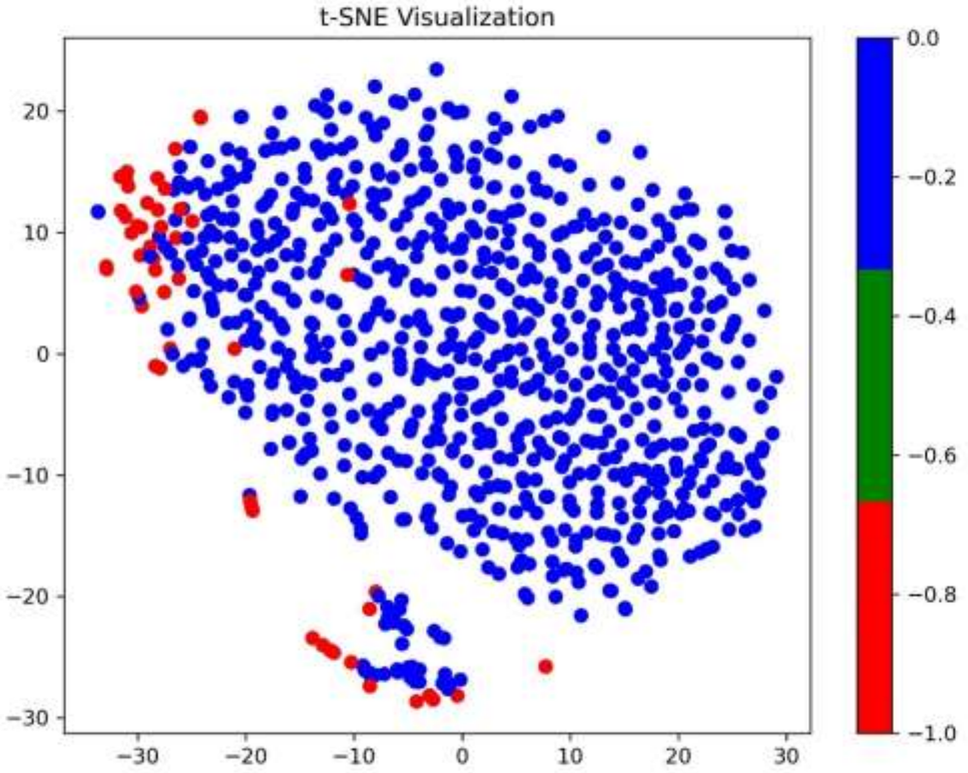

DBSCAN – Best based on Silhouette:

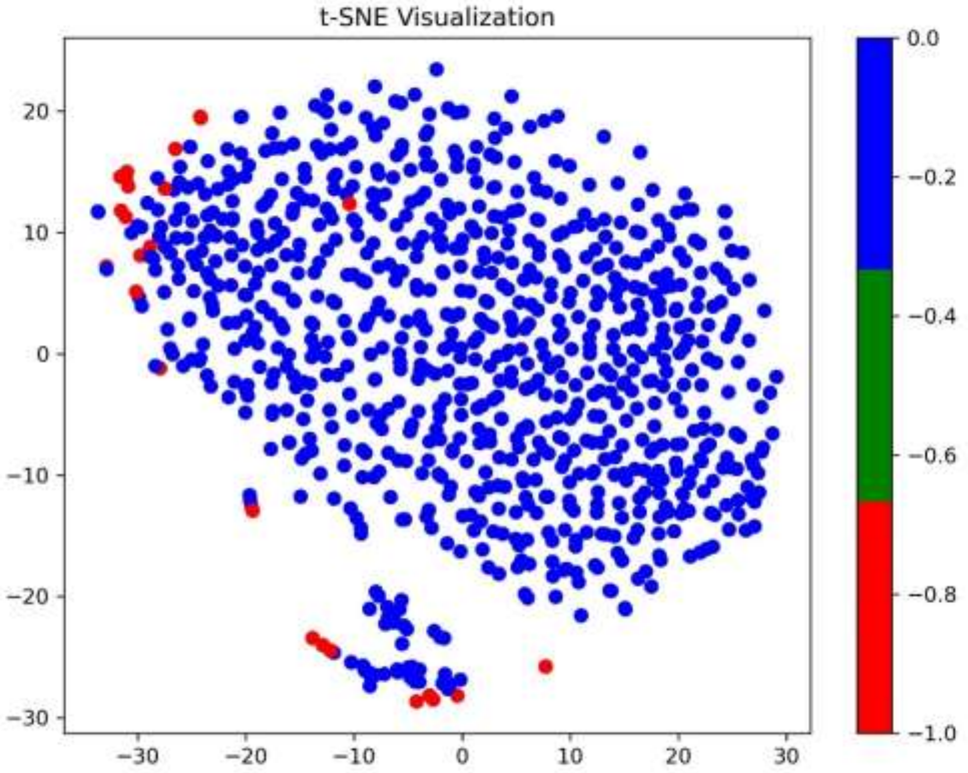

Best model – GMM with K = 3.

Right Masseter Muscle

Data visualization:

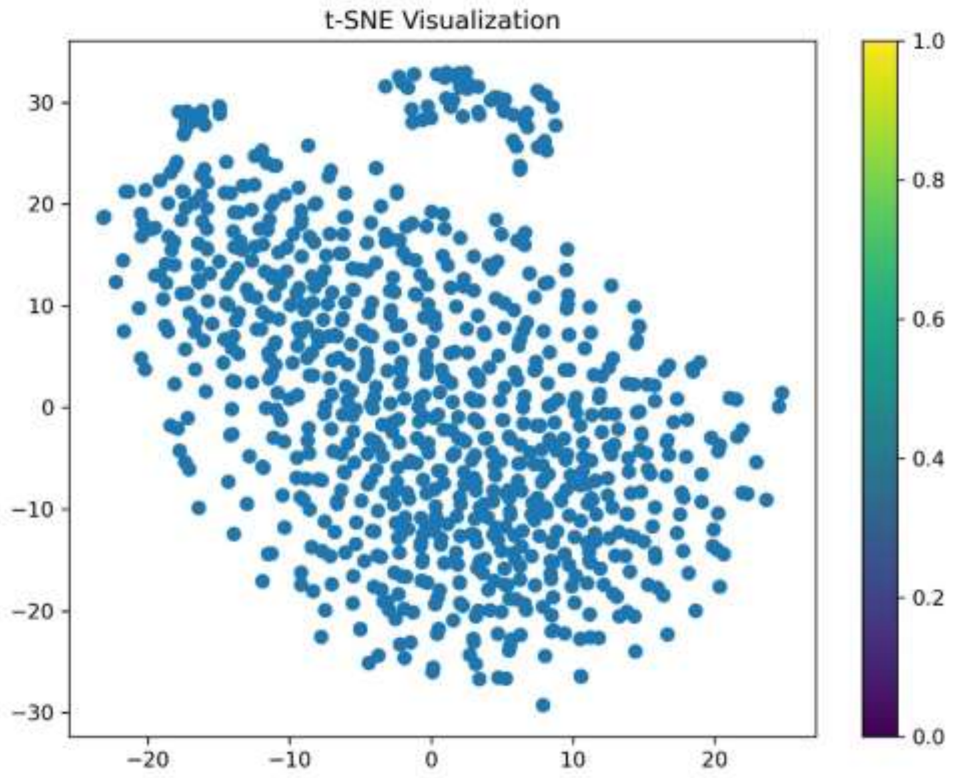

K-Means Elbow and Silhouette:

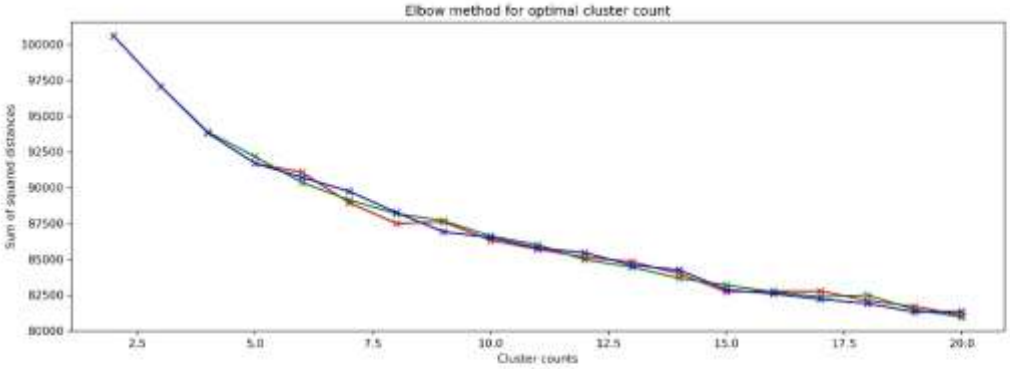

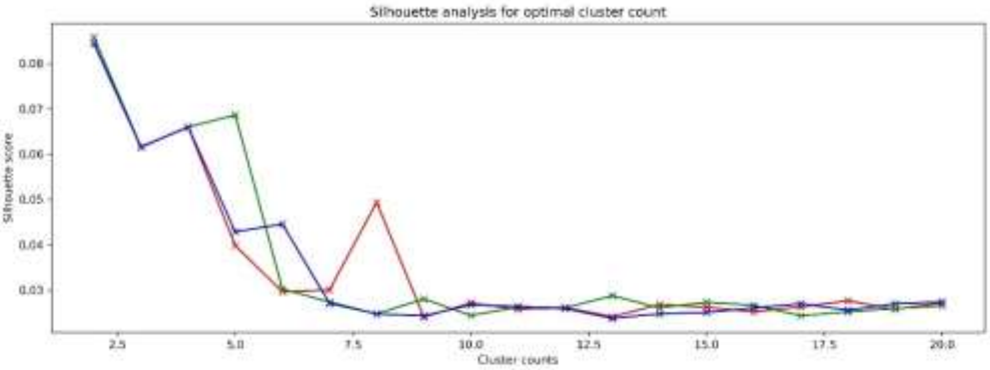

K-Means clustering:

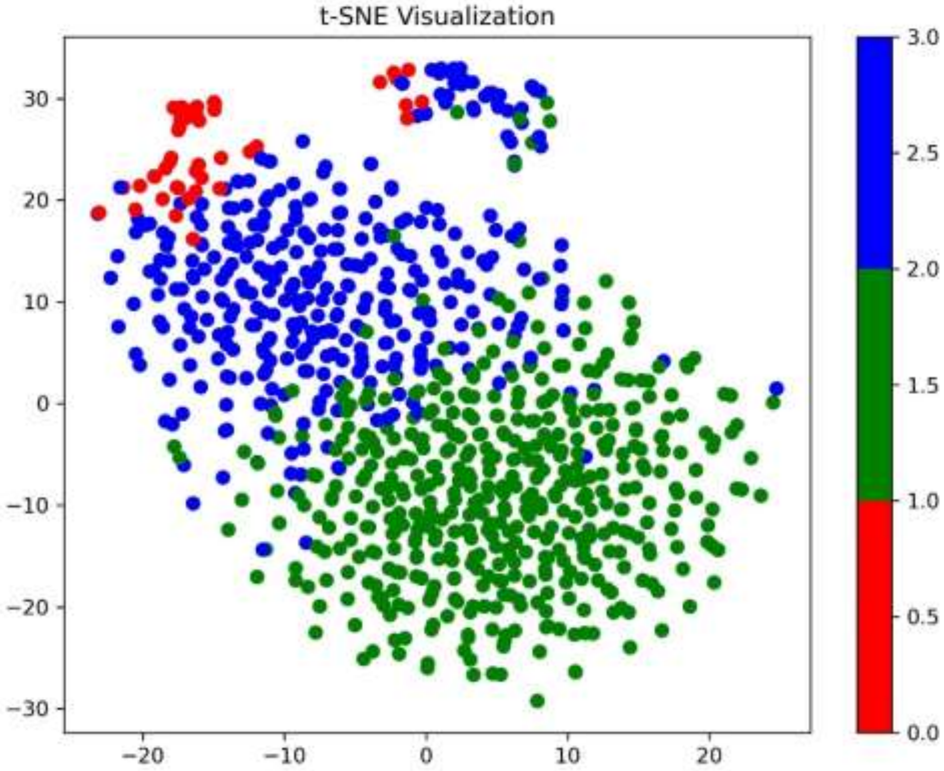

GMM Silhouette and BIC:

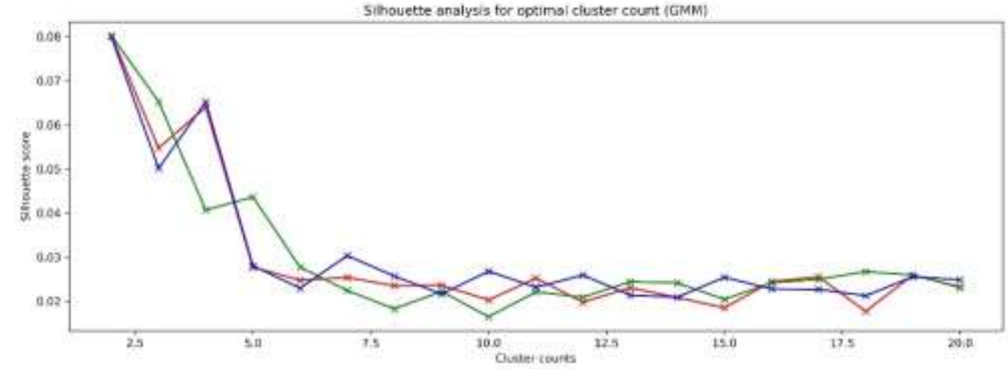

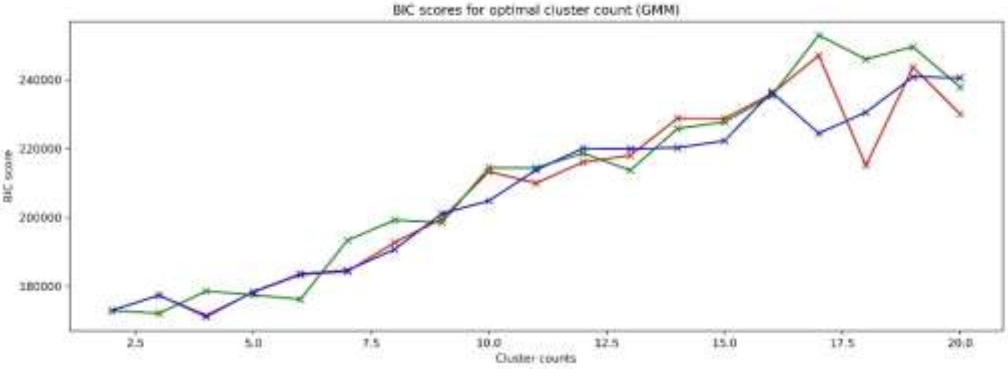

GMM Clustering:

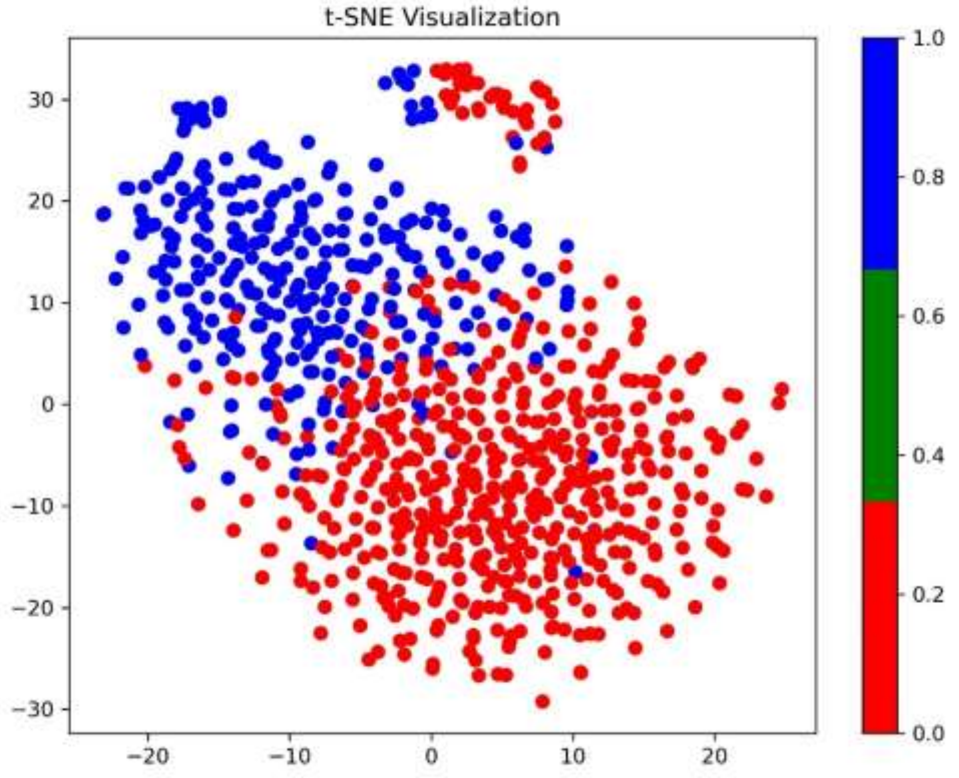

DBSCAN epsilon:

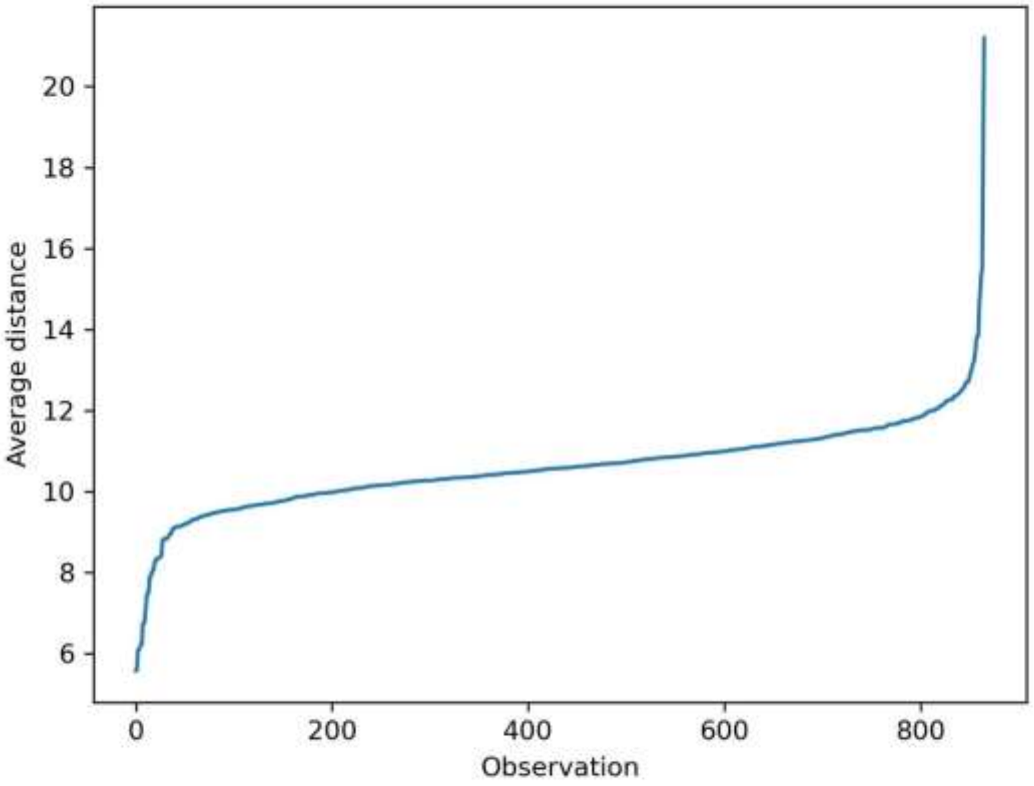

DBSCAN – best based on Davies Bouldin

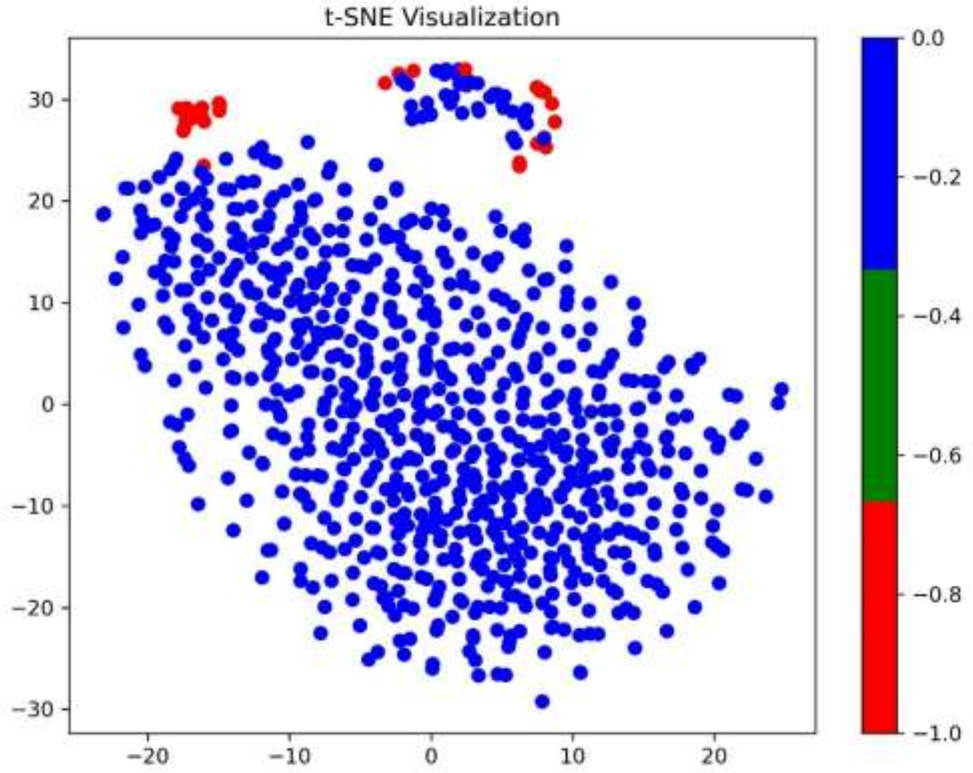

DBSCAN – Best based on Silhouette

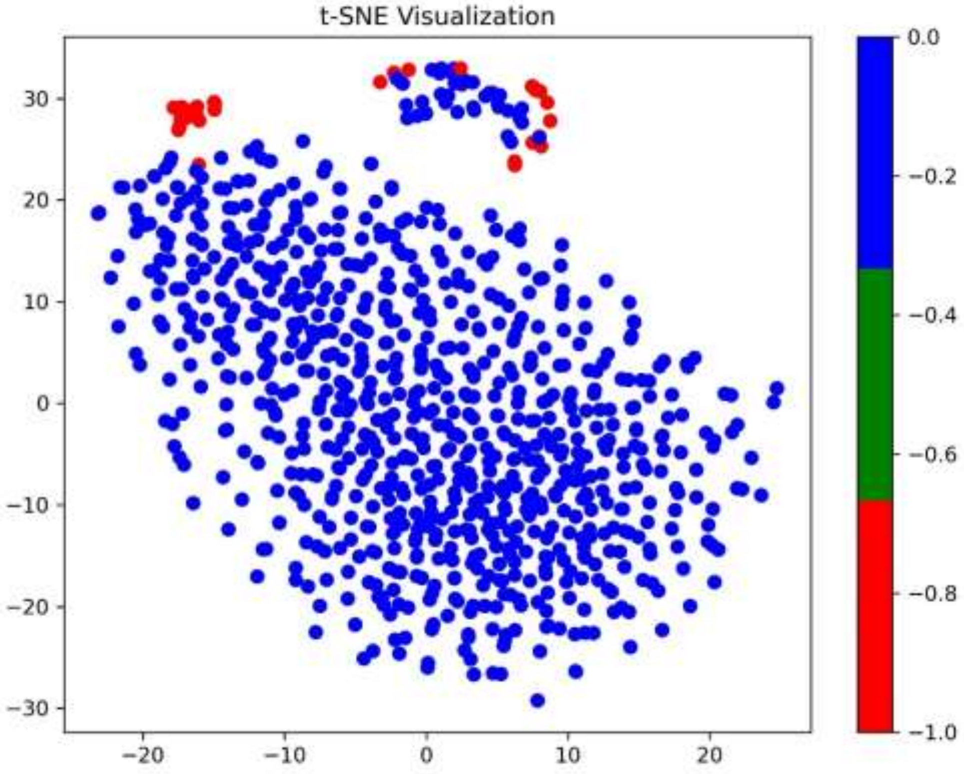

Best model – DBSCAN with Epsilon = 13.8, Min Points = 91.

Left Masseter Muscle

Data visualization:

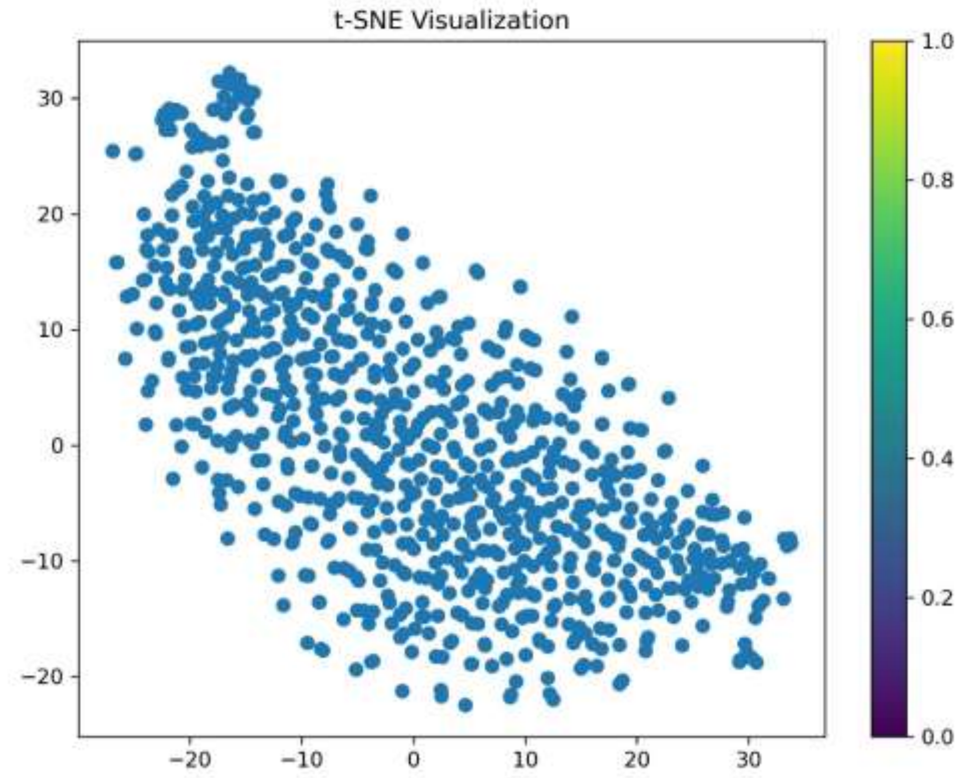

K-Means Elbow and Silhouette:

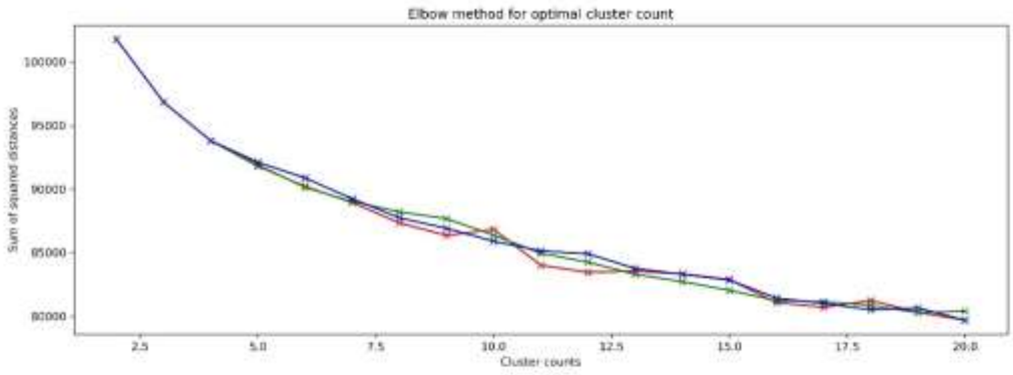

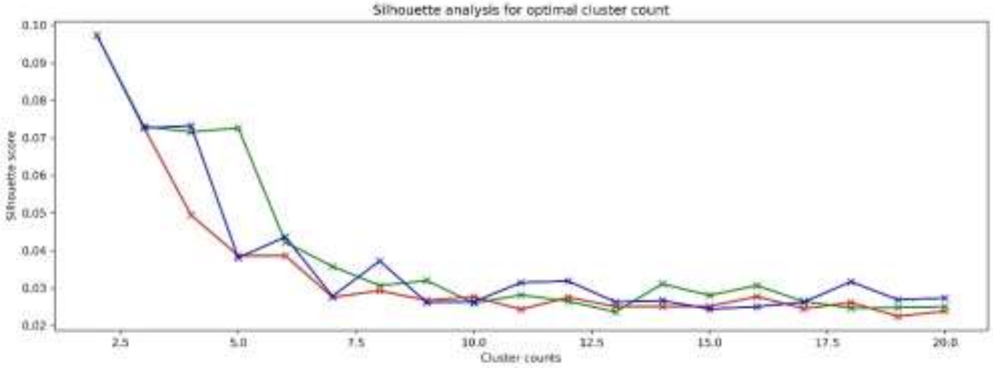

K-Means clustering:

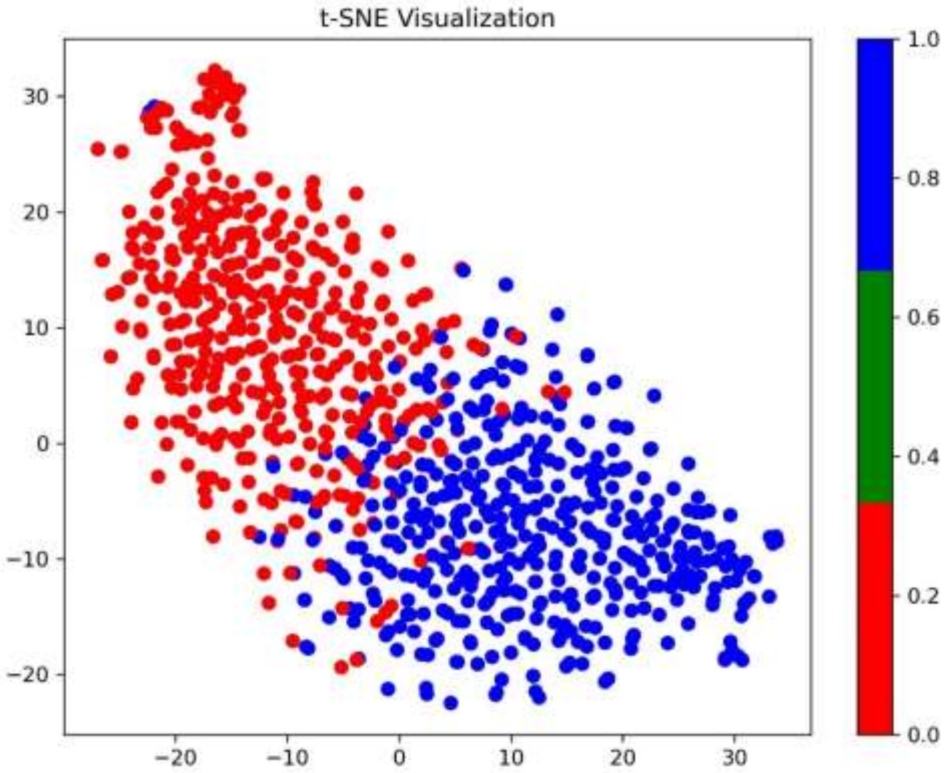

GMM Silhouette and BIC:

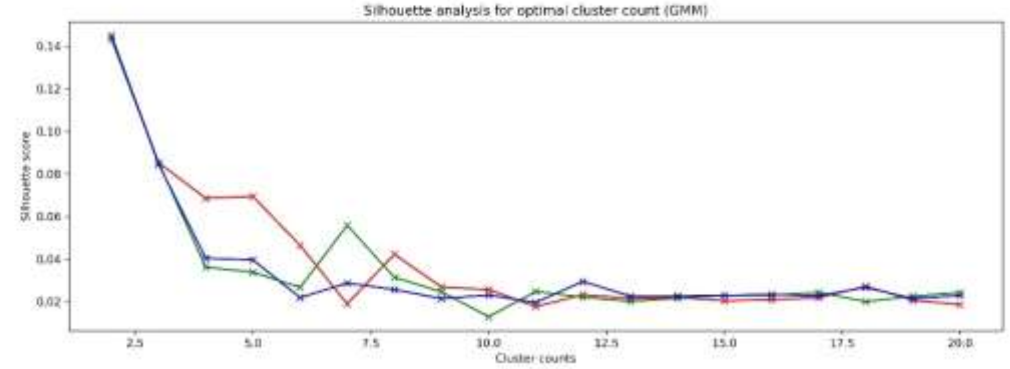

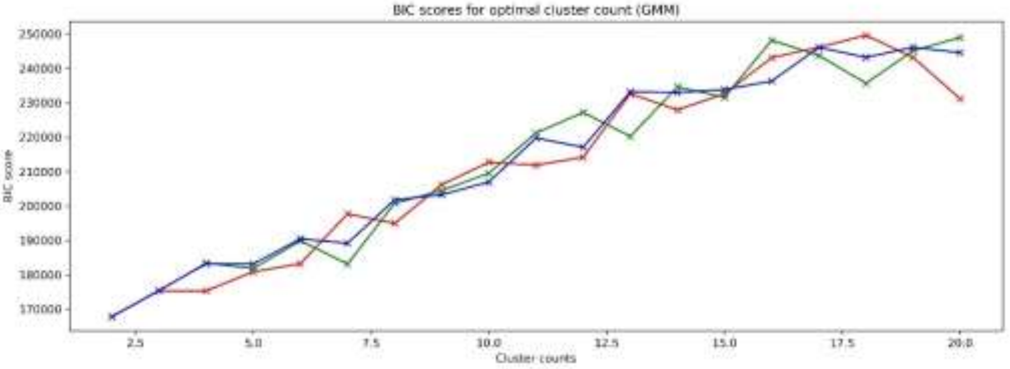

GMM Clustering:

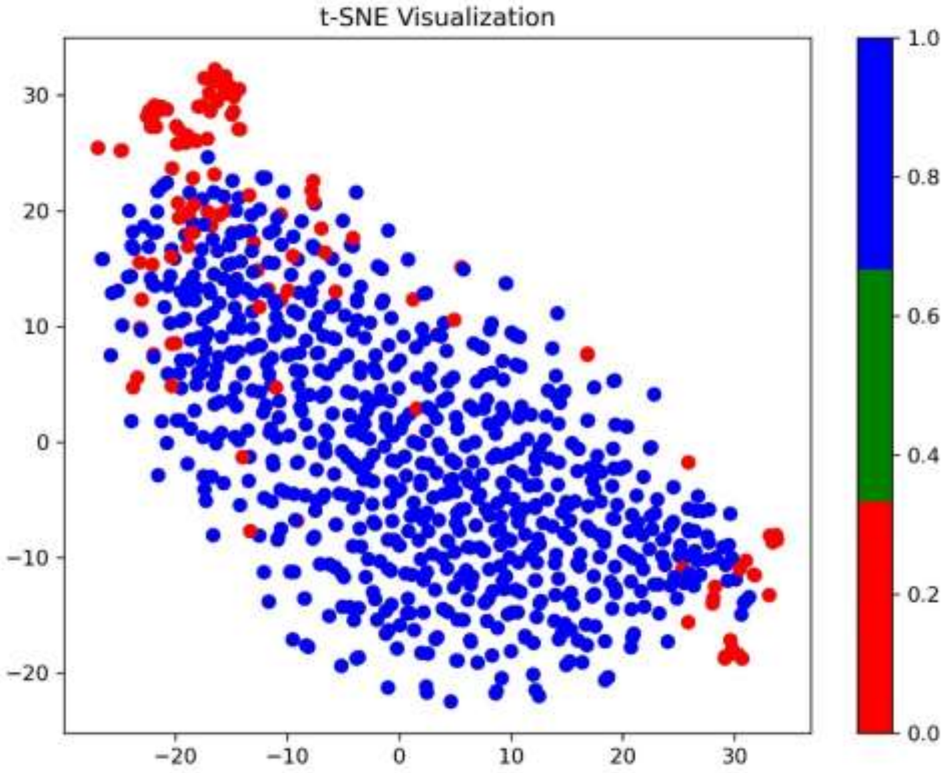

DBSCAN epsilon:

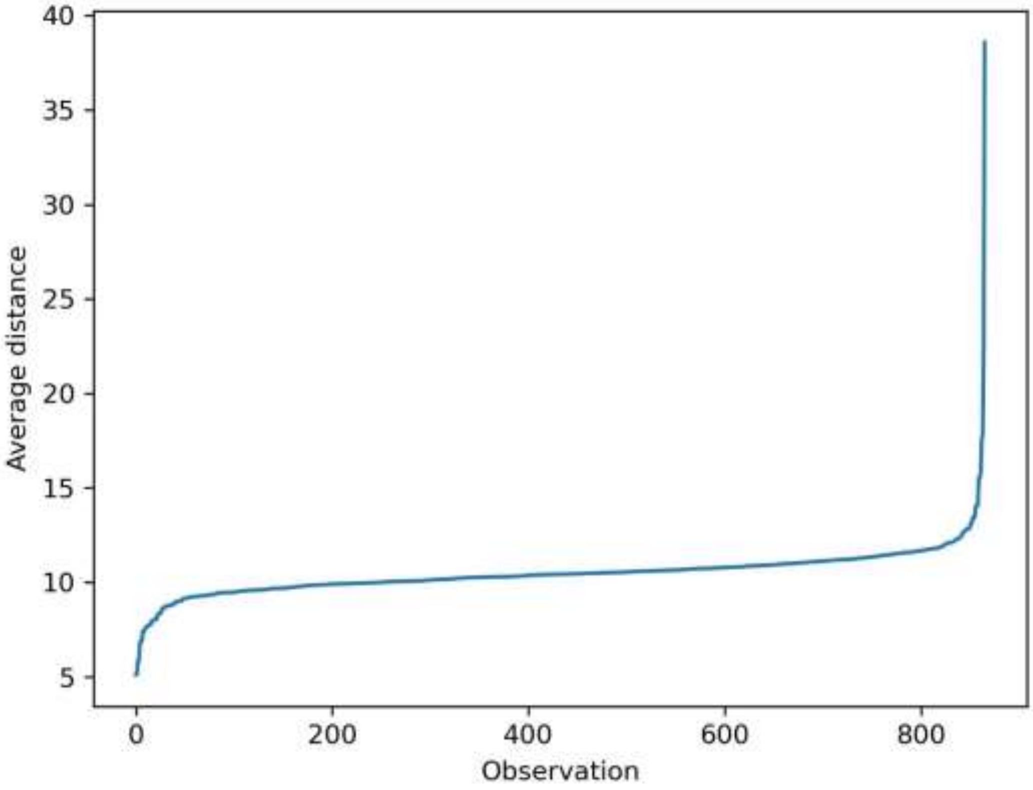

DBSCAN – best based on Davies Bouldin

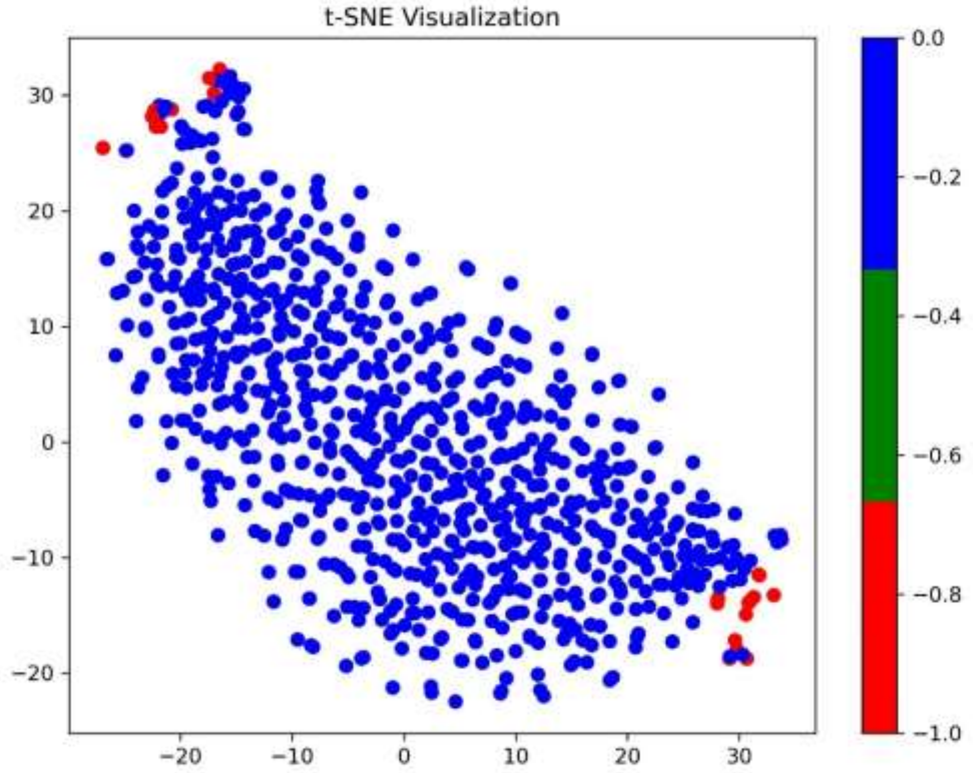

DBSCAN – best based on Silhouette:

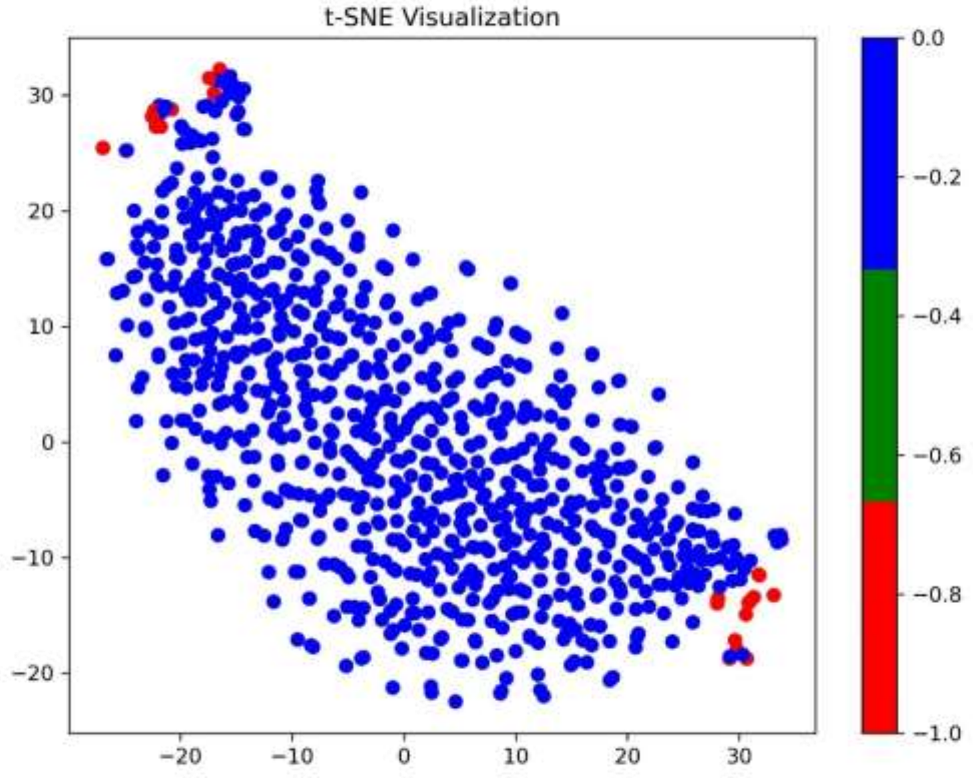

Best model – DBSCAN with Epsilon = 13.8, Min Points = 71.

Right Digastric Muscle

Data visualization:

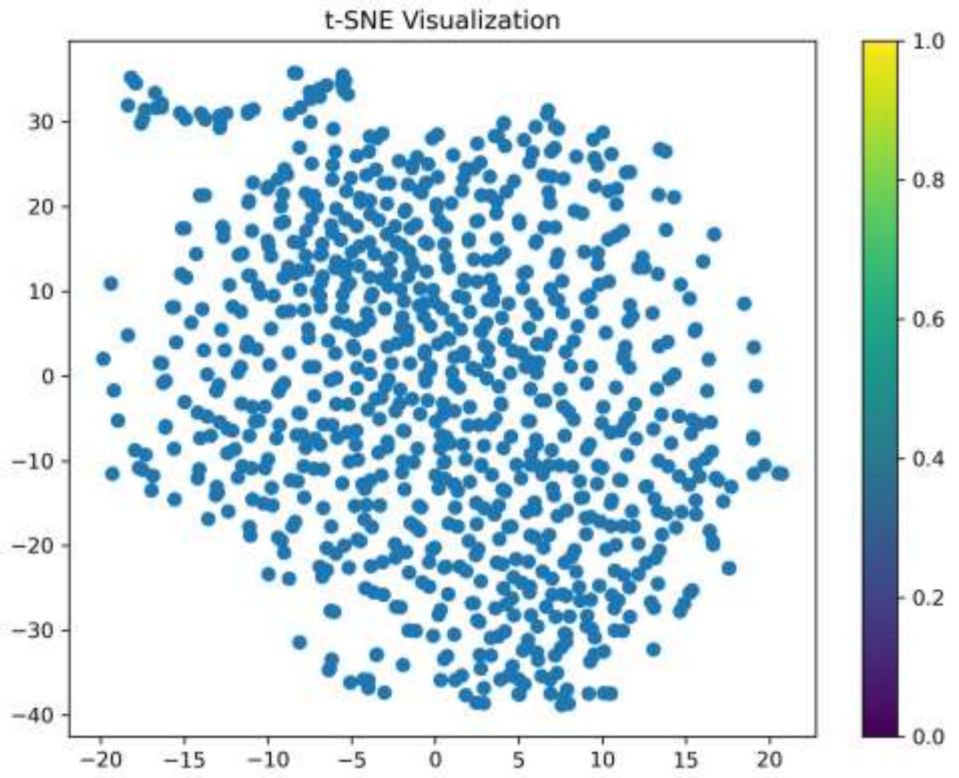

K-Means Elbow and Silhouette:

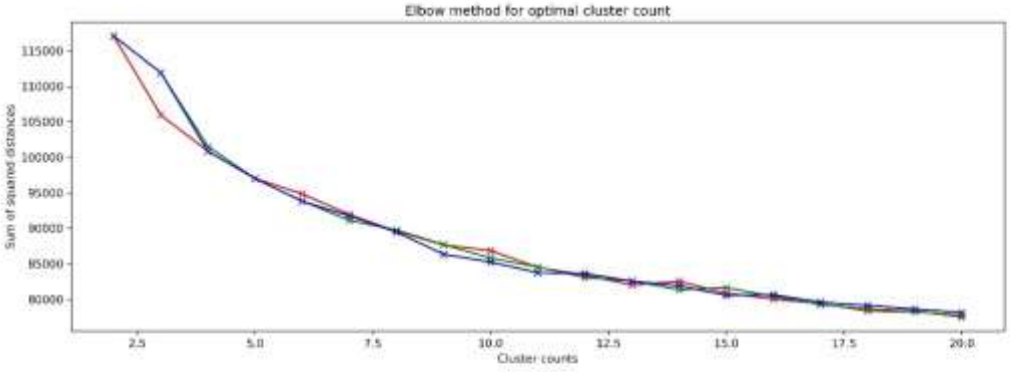

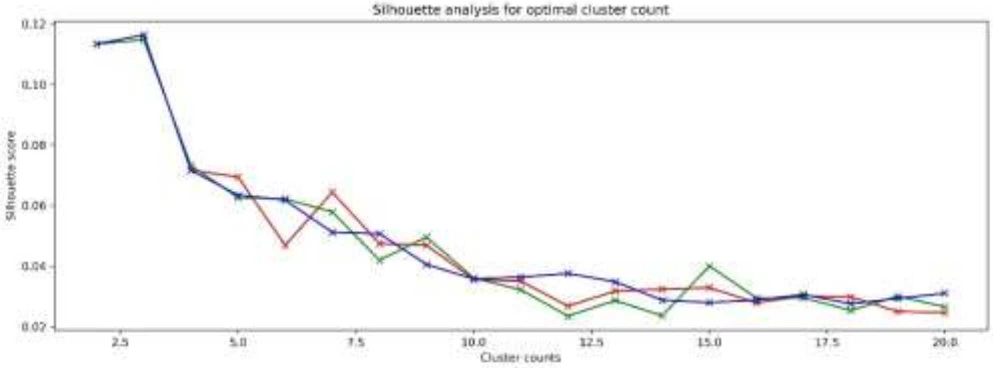

K-Means clustering:

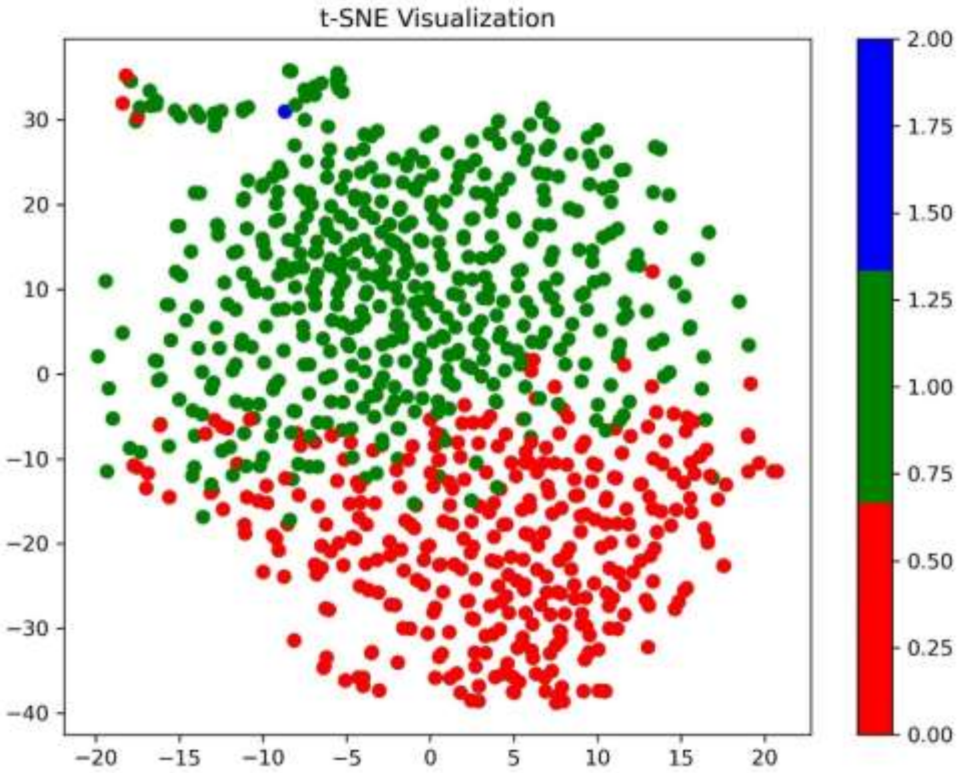

GMM Silhouette and BIC:

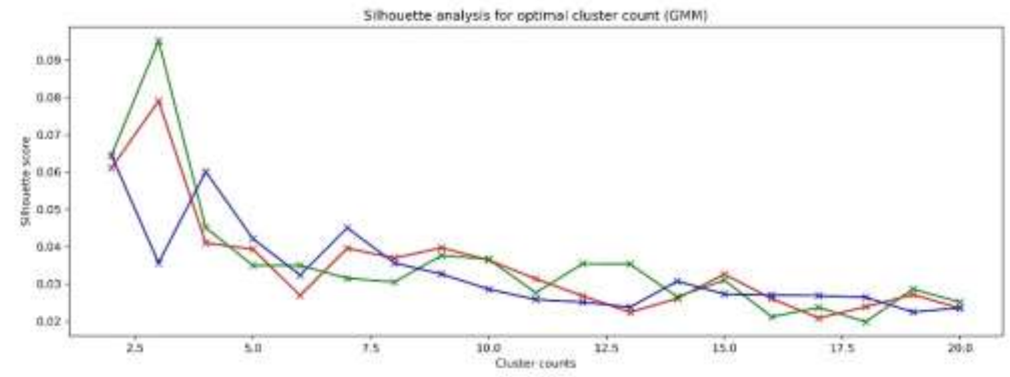

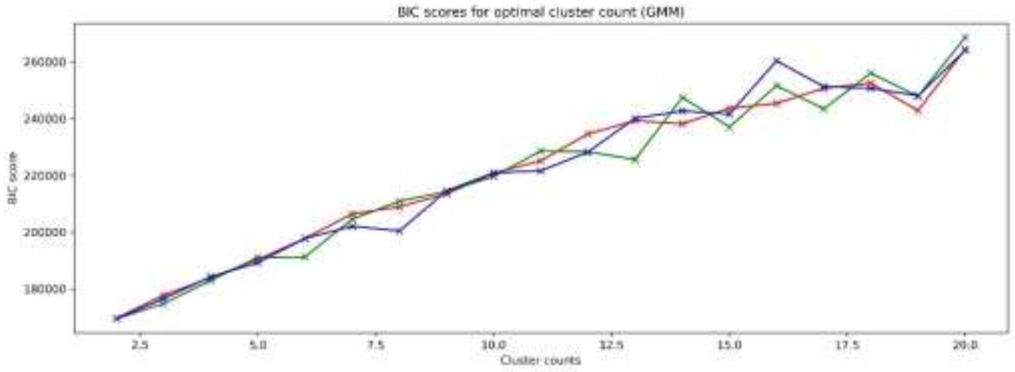

GMM Clustering:

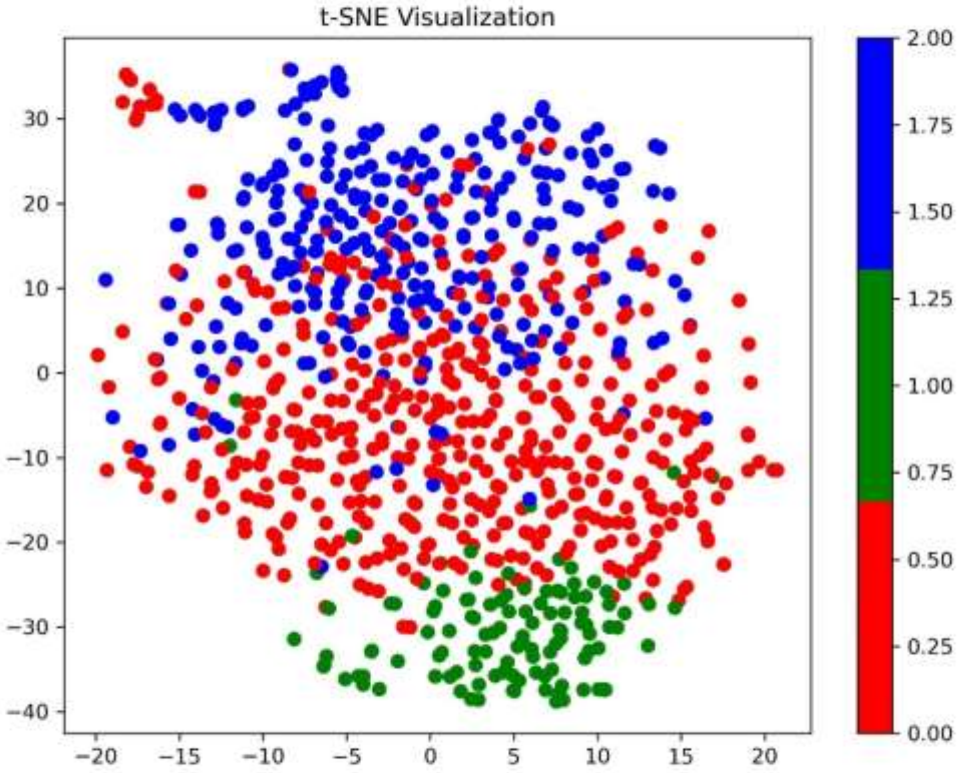

DBSCAN epsilon:

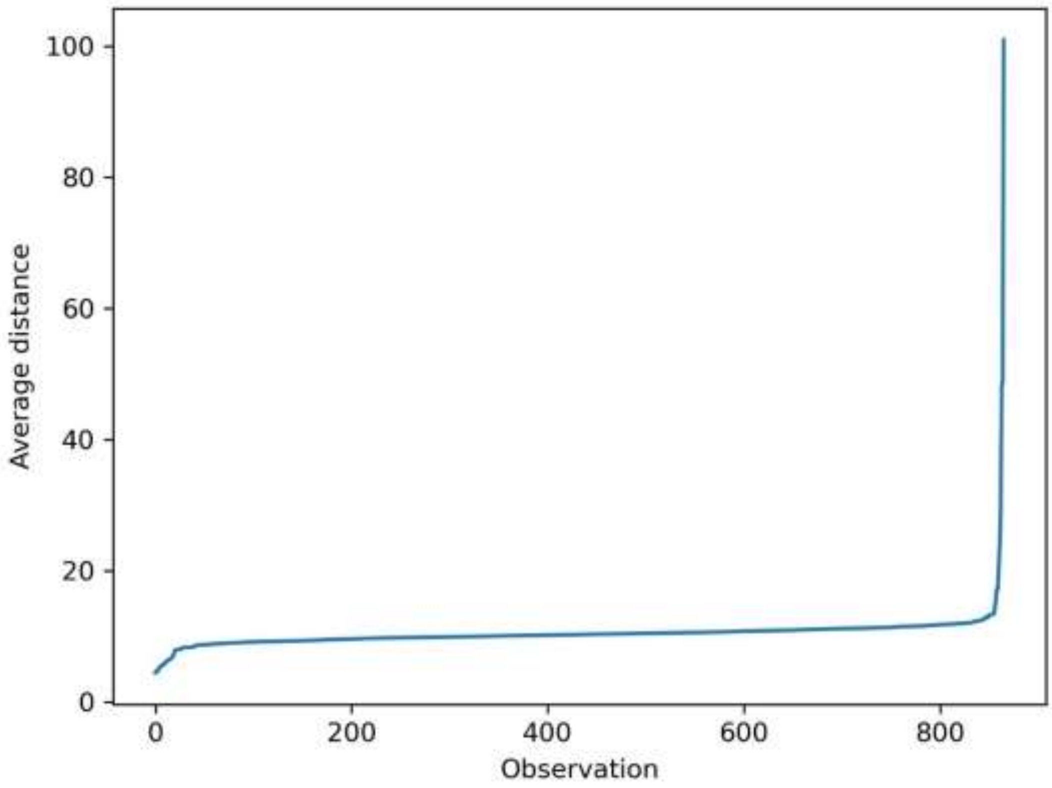

DBSCAN – best based on Davies Bouldin

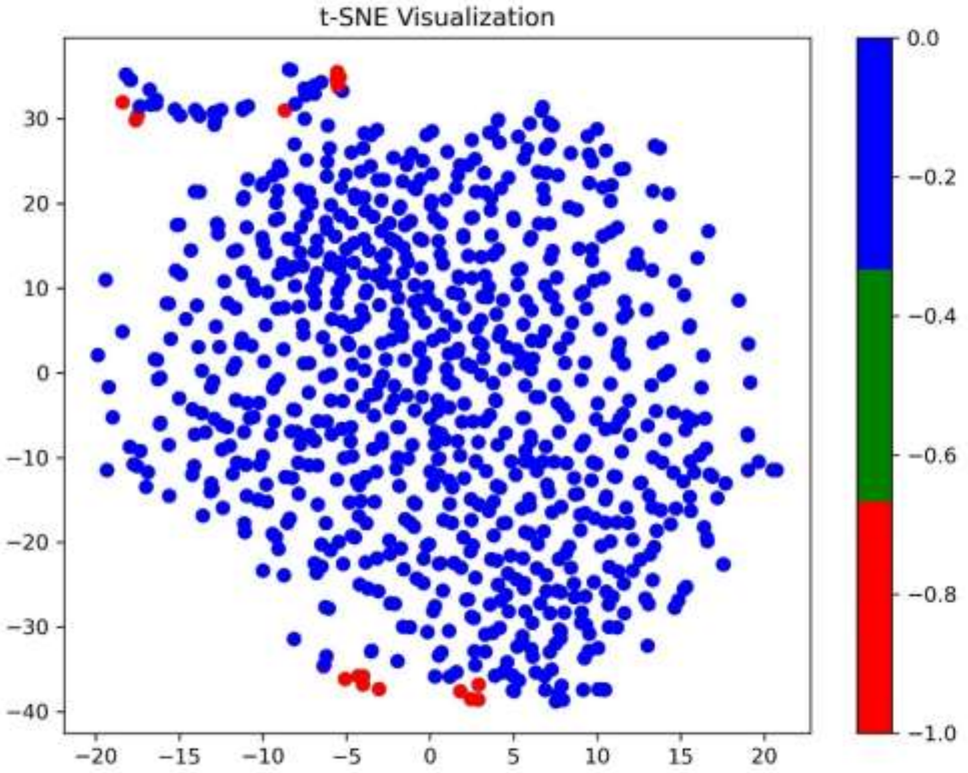

DBSCAN – Best based on Silhouette:

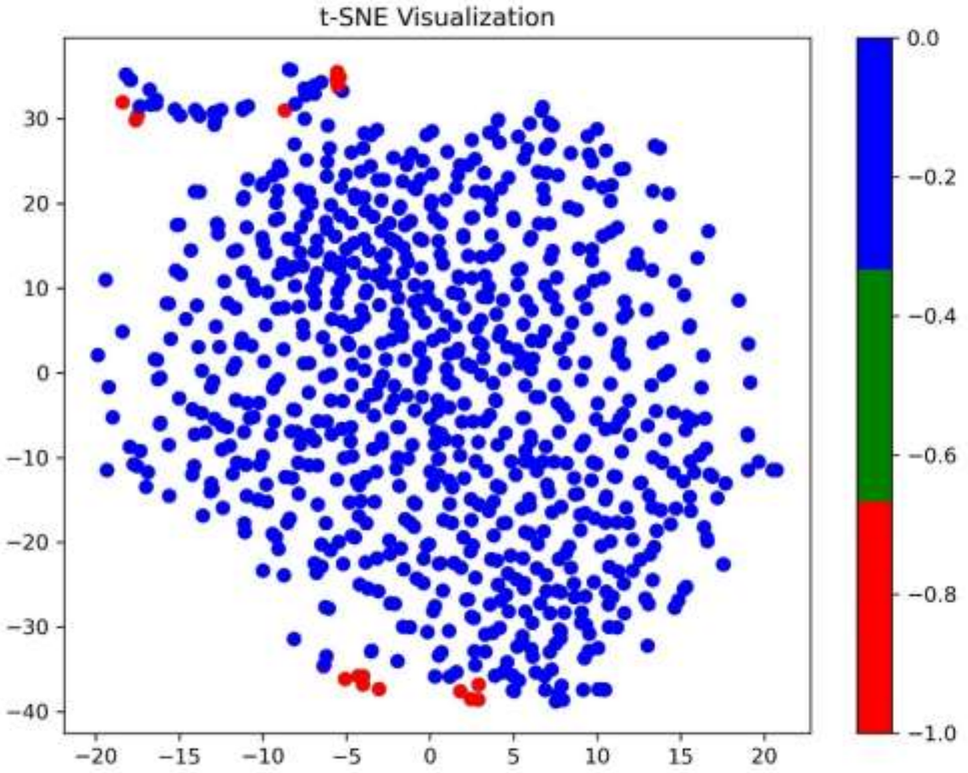

Best model – DBSCAN with Epsilon = 13.8, Min Points = 51.

Left Digastric Muscle

Data visualization:

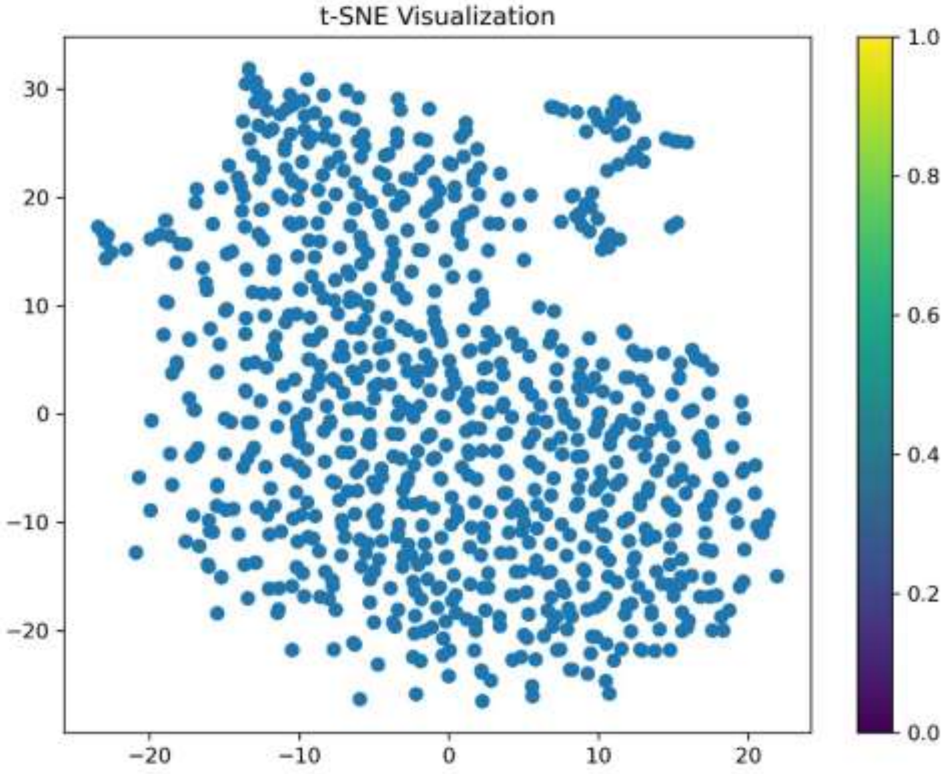

K-Means Elbow and Silhouette:

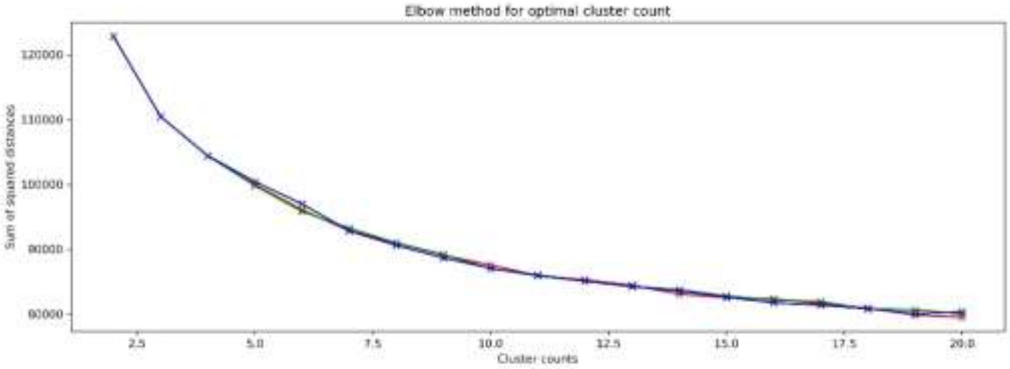

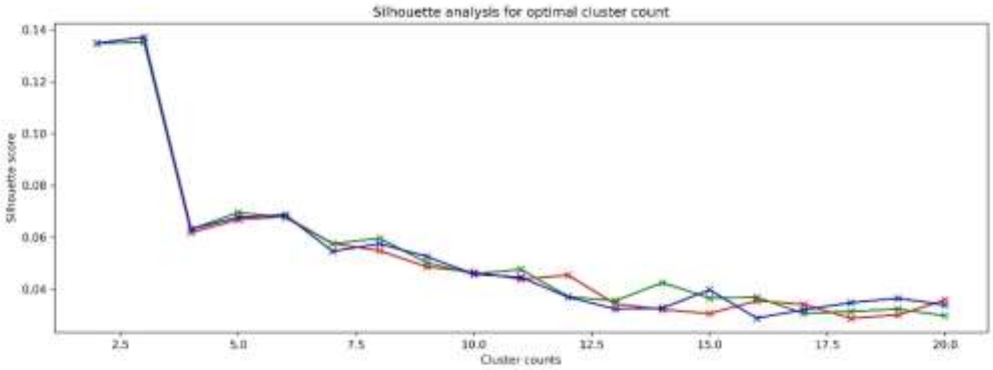

K-Means clustering:

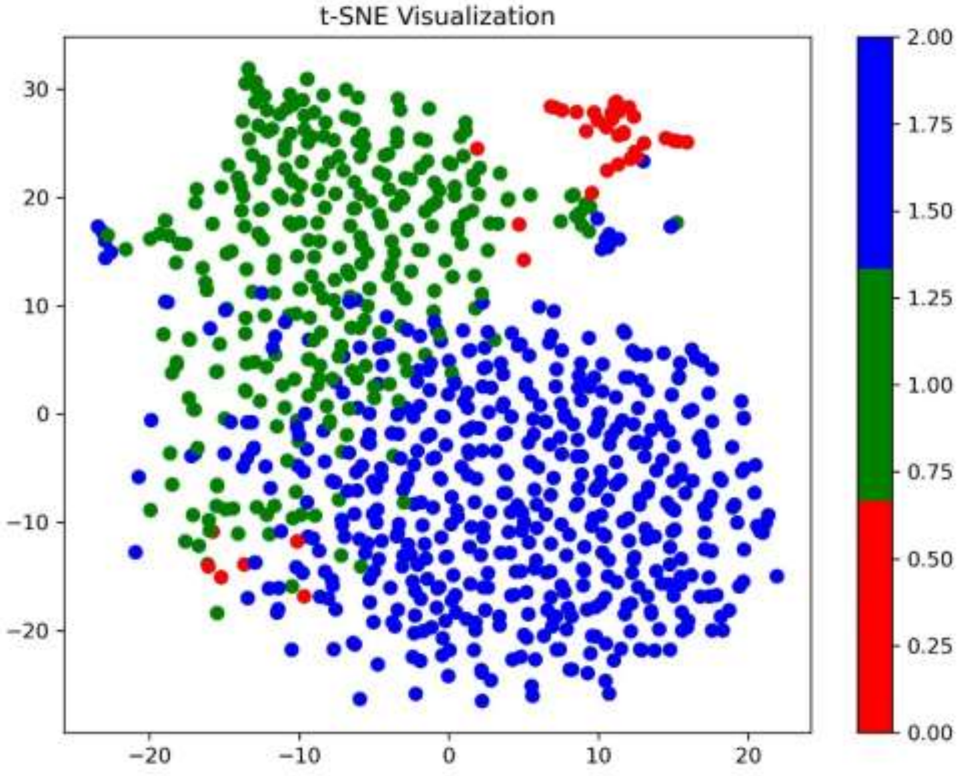

GMM Silhouette and BIC:

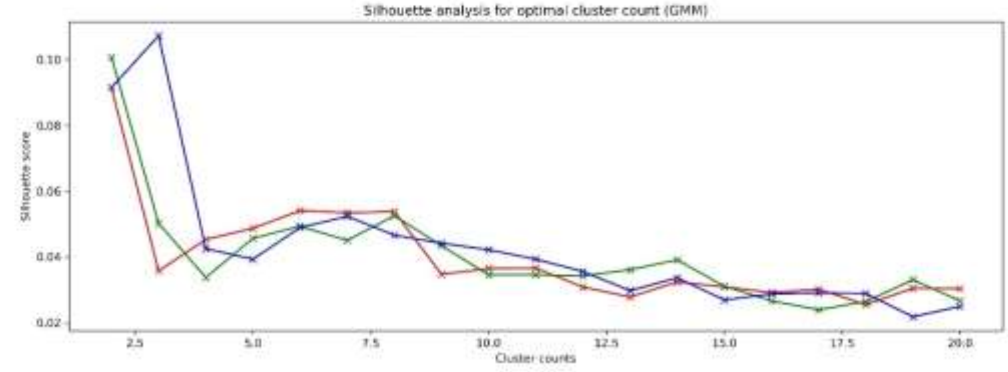

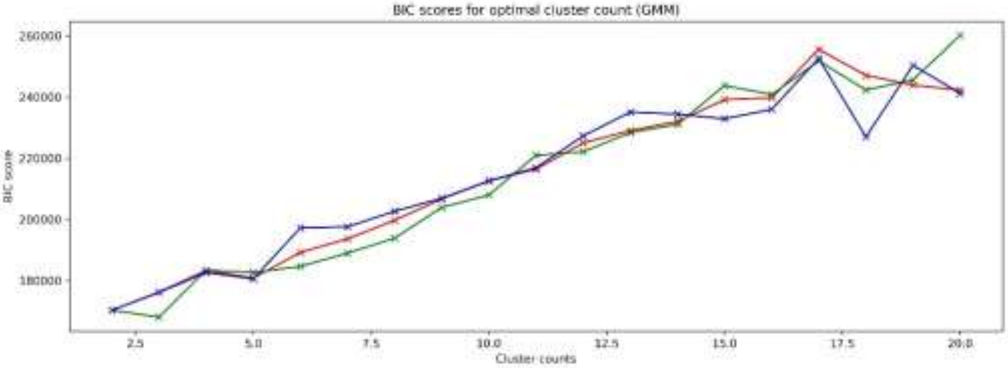

GMM Clustering:

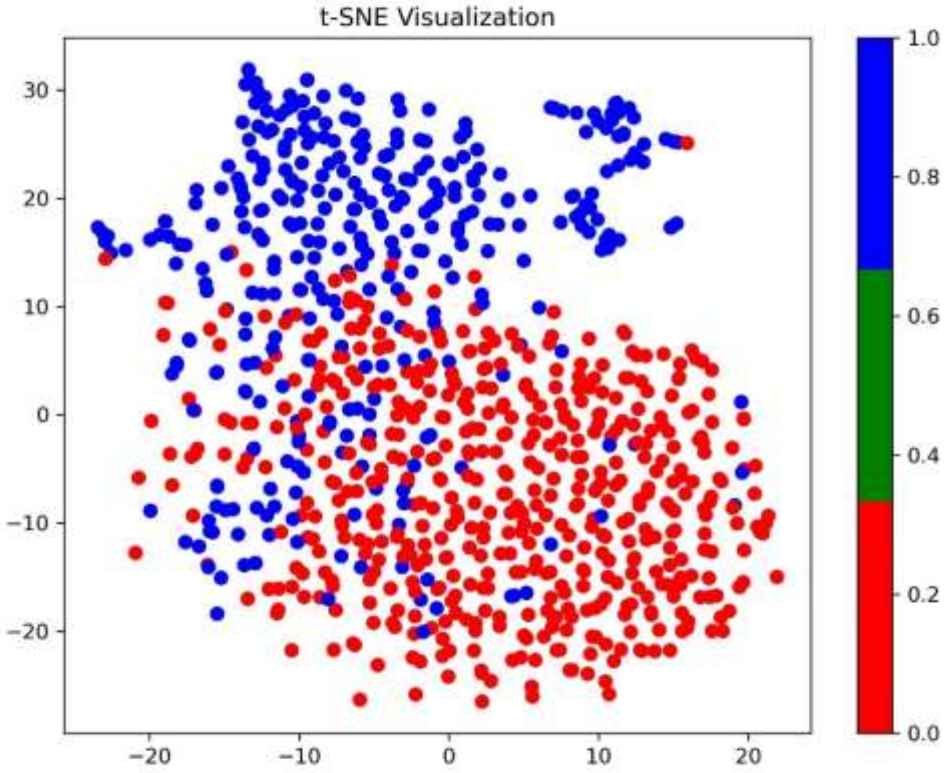

DBSCAN epsilon:

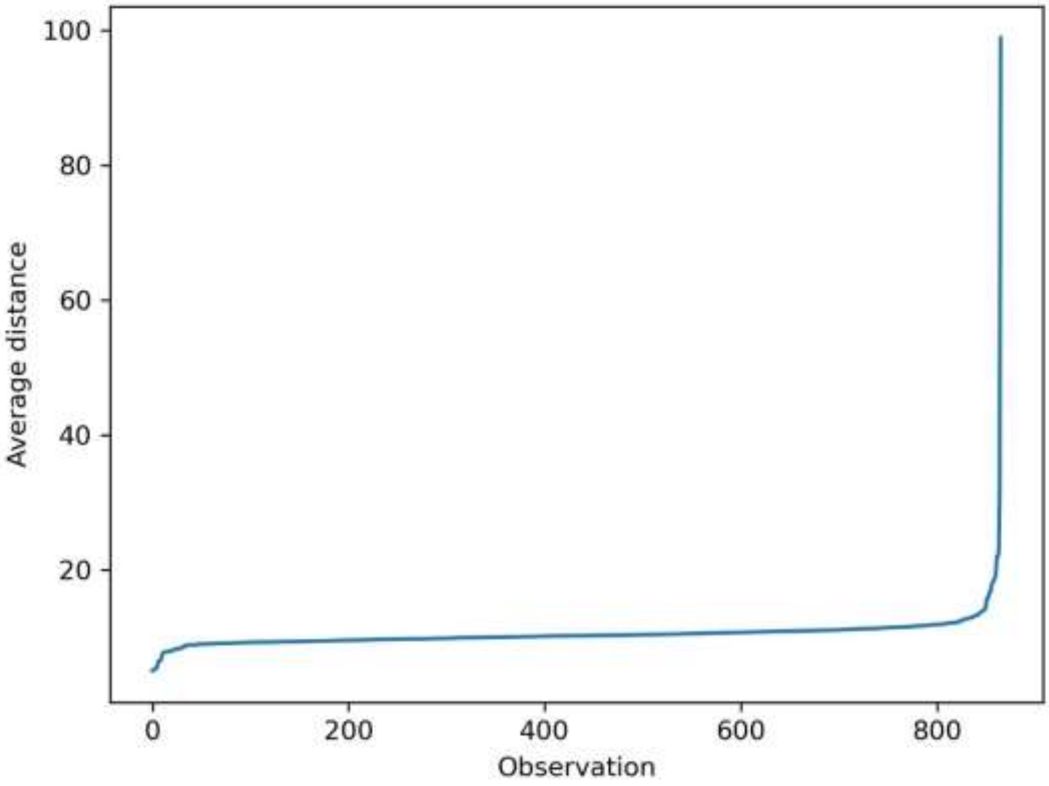

DBSCAN – best based on Davies Bouldin

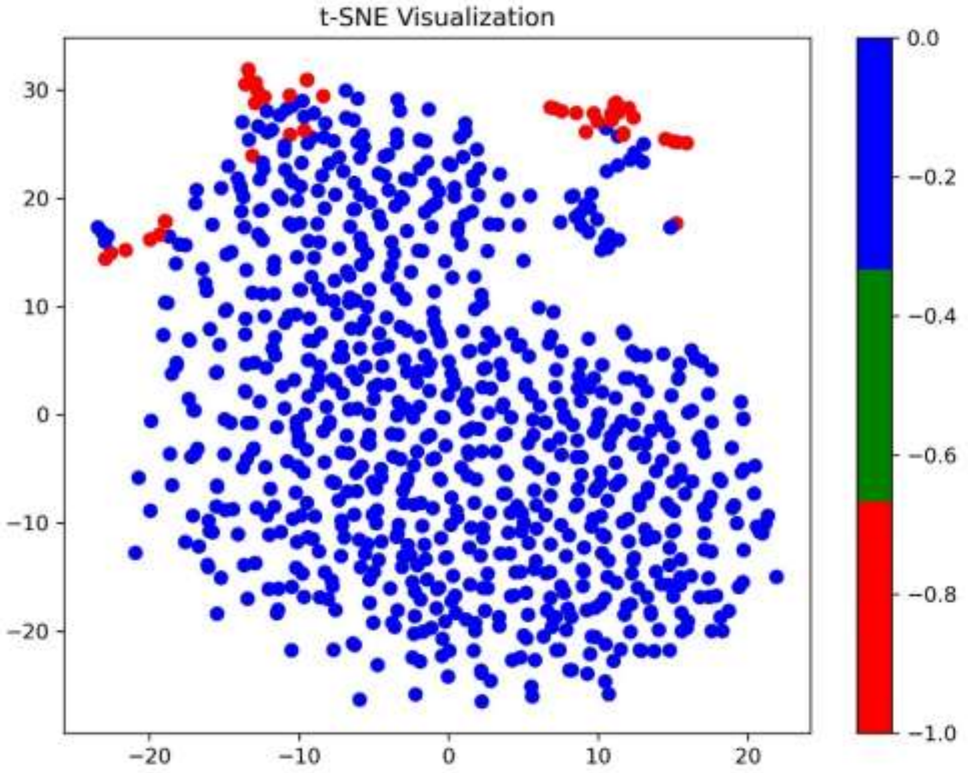

DBSCAN – Best based on Silhouette:

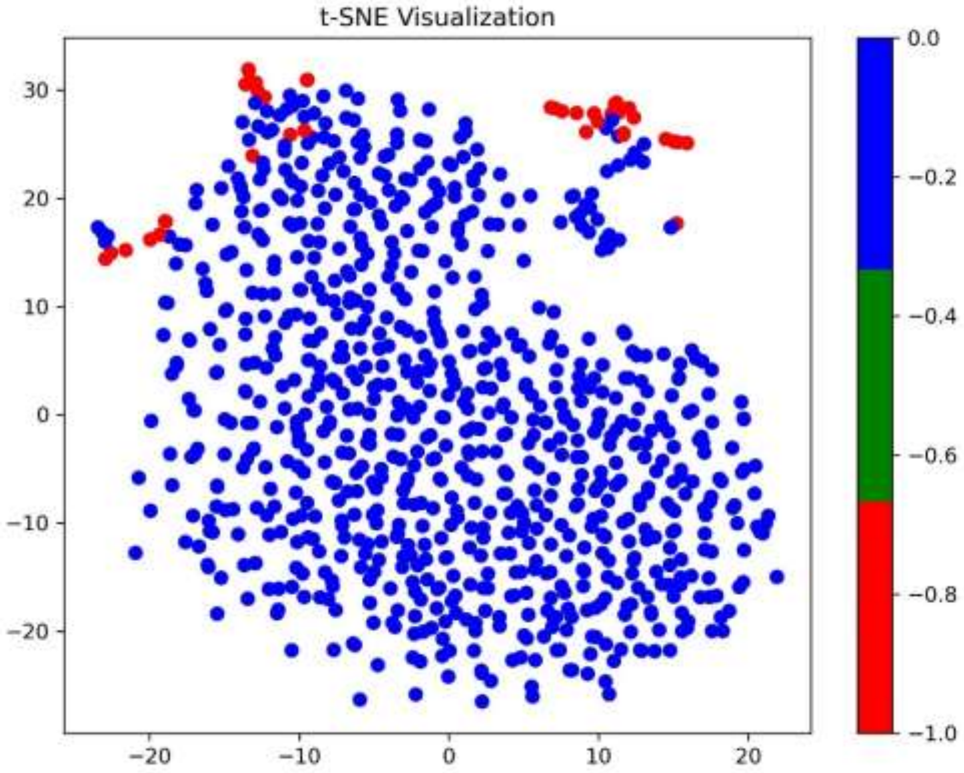

Best model – DBSCAN with Epsilon = 13.8, Min Points = 61.

Normalised signal intensity quotient for muscle activity in different functions

Participant	Maximum Mouth Opening						Maximum lateral excursion						Maximum anterior protrusion						Chewing					
	TA-R	TA-L	MM-R	MM-L	DA-R	DA-L	TA-R	TA-L	MM-R	MM-L	DA-R	DA-L	TA-R	TA-L	MM-R	MM-L	DA-R	DA-L	TA-R	TA-L	MM-R	MM-L	DA-R	DA-L
1	0.22	0.2	0.18	0.19	0.18	0.21	0.14	0.17	0.14	0.13	0.16	0.15	0.1	0.13	0.06	0.08	0.09	0.08	0.25	0.25	0.17	0.25	0.18	0.18
2	0.12	0.12	0.1	0.1	0.11	0.09	0.13	0.17	0.2	0.14	0.16	0.15	0.1	0.14	0.06	0.07	0.09	0.08	0.18	0.13	0.1	0.11	0.11	0.11
3	0.21	0.19	0.17	0.18	0.18	0.17	0.14	0.18	0.15	0.14	0.17	0.16	0.16	0.13	0.1	0.11	0.05	0.1	0.09	0.07	0.05	0.12	0.06	0.08
4	0.15	0.13	0.12	0.13	0.13	0.12	0.14	0.16	0.15	0.15	0.17	0.16	0.11	0.08	0.04	0.05	0.07	0.06	0	0	0	0	0.07	0.05
5	0.14	0.12	0.11	0.11	0.1	0.13	0.15	0.15	0.21	0.14	0.17	0.16	0.14	0.14	0.07	0.08	0.09	0.08	0.19	0.23	0.15	0.23	0.16	0.16
6	0.15	0.13	0.11	0.12	0.11	0.11	0.07	0.05	0	0.06	0.04	0.04	0.15	0.15	0.11	0.12	0.14	0.13	0.1	0.1	0.03	0.04	0.04	0.03
7	0.23	0.22	0.2	0.2	0.2	0.19	0.09	0.12	0.09	0.09	0.11	0.11	0.16	0.16	0.13	0.13	0.15	0.13	0.16	0.16	0.09	0.16	0.1	0.1
8	0.14	0.12	0.1	0.11	0.1	0.1	0.15	0.19	0.15	0.15	0.18	0.16	0.25	0.25	0.19	0.18	0.2	0.19	0.09	0.08	0.13	0.13	0.06	0.08
9	0.16	0.15	0.13	0.14	0.13	0.13	0.29	0.32	0.29	0.29	0.31	0.3	0.23	0.23	0.16	0.17	0.19	0.18	0.04	0.05	0	0.06	0.06	0.07
10	0.2	0.18	0.17	0.18	0.18	0.19	0.04	0.07	0.04	0.04	0.06	0.05	0.13	0.16	0.09	0.1	0.12	0.11	0.09	0.08	0.06	0.13	0.07	0.06
11	0.1	0.09	0.07	0.08	0.07	0.07	0.22	0.27	0.3	0.23	0.25	0.24	0.2	0.16	0.13	0.13	0.15	0.14	0.16	0.16	0.16	0.1	0.1	0.09
12	0.25	0.23	0.21	0.22	0.22	0.21	0.04	0.08	0.04	0.04	0.06	0.06	0.1	0.1	0.06	0.07	0.07	0.08	0.14	0.09	0.07	0.08	0.07	0.09
13	0.17	0.15	0.13	0.14	0.14	0.13	0.09	0.1	0.09	0.09	0.12	0.11	0.21	0.21	0.13	0.14	0.16	0.15	0.04	0.05	0	0	0.06	0.05
14	0.12	0.11	0.09	0.09	0.1	0.08	0.06	0.06	0.06	0.05	0.08	0.07	0.25	0.25	0.18	0.19	0.21	0.19	0.04	0.05	0.07	0.06	0.06	0.05

15	0.11	0.09	0.07	0.08	0.07	0.07	0.17	0.21	0.24	0.17	0.2	0.18	0.21	0.18	0.15	0.15	0.17	0.16	0	0.05	0.07	0	0.06	0.05
16	0.17	0.15	0.13	0.14	0.14	0.13	0.12	0.16	0.13	0.12	0.15	0.14	0.03	0.03	0.08	0.06	0.07	0.06	0	0	0	0.06	0.06	0.07
17	0.15	0.14	0.12	0.12	0.13	0.11	0.08	0.1	0.09	0.09	0.11	0.1	0.12	0.12	0.09	0.09	0.09	0.1	0.24	0.23	0.21	0.22	0.21	0.21
18	0.18	0.16	0.15	0.15	0.14	0.14	0.08	0.12	0.09	0.09	0.11	0.1	0.16	0.12	0.08	0.09	0.09	0.1	0.17	0.16	0.21	0.15	0.15	0.14
19	0.23	0.22	0.2	0.2	0.19	0.19	0.1	0.14	0.11	0.1	0.13	0.12	0.13	0.09	0.07	0.07	0.06	0.07	0.13	0.12	0.17	0.11	0.11	0.12
20	0.18	0.16	0.14	0.15	0.15	0.14	0.09	0.1	0.09	0.09	0.11	0.11	0.2	0.2	0.17	0.17	0.19	0.18	0.14	0.17	0.1	0.17	0.11	0.13
21	0.14	0.13	0.11	0.12	0.11	0.11	0.13	0.16	0.19	0.13	0.15	0.14	0.14	0.14	0.1	0.12	0.13	0.11	0.09	0.07	0.05	0.06	0.06	0.08
22	0.11	0.1	0.08	0.09	0.09	0.1	0.16	0.17	0.23	0.16	0.19	0.18	0.13	0.13	0.1	0.11	0.12	0.11	0	0.05	0	0.06	0.06	0.05
23	0.22	0.21	0.19	0.2	0.2	0.19	0.08	0.11	0.14	0.08	0.1	0.09	0.2	0.17	0.14	0.15	0.16	0.15	0.1	0.08	0.06	0.13	0.07	0.07
24	0.1	0.08	0.07	0.07	0.07	0.09	0.07	0.08	0.07	0.07	0.09	0.09	0.17	0.17	0.15	0.14	0.14	0.15	0.16	0.19	0.12	0.13	0.13	0.13
25	0.08	0.07	0.05	0.06	0.06	0.05	0.06	0.09	0.12	0.06	0.08	0.08	0.24	0.23	0.21	0.21	0.22	0.21	0.04	0	0	0.06	0.07	0.07
26	0.15	0.13	0.12	0.13	0.12	0.12	0.16	0.19	0.22	0.16	0.18	0.17	0.16	0.16	0.11	0.13	0.15	0.13	0.15	0.15	0.08	0.09	0.09	0.09
27	0.13	0.11	0.1	0.11	0.11	0.1	0.06	0.1	0.12	0.06	0.08	0.08	0.13	0.13	0.1	0.11	0.1	0.11	0.04	0.05	0.07	0	0.06	0.07
28	0.09	0.07	0.05	0.06	0.06	0.05	0.05	0.09	0.05	0.05	0.07	0.07	0.09	0.09	0.05	0.07	0.08	0.07	0	0.05	0.07	0	0.06	0.05
29	0.2	0.18	0.16	0.17	0.16	0.16	0.06	0.12	0.15	0.09	0.11	0.1	0.03	0.03	0.06	0.06	0.07	0.06	0	0	0	0.06	0.07	0.07
30	0.15	0.13	0.11	0.12	0.11	0.11	0.08	0.09	0.14	0.08	0.1	0.1	0.18	0.17	0.15	0.15	0.16	0.15	0.12	0.07	0.12	0.12	0.06	0.07
31	0.13	0.11	0.1	0.11	0.1	0.1	0.24	0.24	0.3	0.23	0.26	0.25	0.23	0.26	0.18	0.2	0.22	0.2	0	0	0	0.06	0.06	0.05
32	0.23	0.21	0.19	0.2	0.2	0.19	0.07	0.08	0.07	0.07	0.09	0.09	0.12	0.12	0.05	0.06	0.06	0.07	0	0.05	0.07	0	0.06	0.07

33	0.13	0.11	0.09	0.1	0.1	0.09	0.06	0.06	0.12	0.05	0.08	0.07	0.26	0.23	0.19	0.2	0.2	0.21	0.04	0.05	0.07	0.06	0.06	0.07
34	0.12	0.1	0.09	0.09	0.09	0.11	0.13	0.14	0.14	0.13	0.16	0.15	0.12	0.12	0.09	0.09	0.09	0.1	0.11	0.15	0.15	0.15	0.09	0.1
35	0.13	0.12	0.1	0.11	0.1	0.1	0.12	0.15	0.12	0.12	0.14	0.13	0.14	0.13	0.11	0.1	0.1	0.11	0.04	0	0.07	0.06	0.06	0.07
36	0.1	0.09	0.07	0.08	0.07	0.07	0.15	0.21	0.15	0.15	0.17	0.16	0.13	0.13	0.06	0.08	0.09	0.08	0	0	0	0.06	0.06	0.07
37	0.13	0.12	0.09	0.1	0.09	0.09	0.13	0.16	0.19	0.12	0.15	0.14	0.14	0.17	0.11	0.11	0.12	0.11	0.29	0.29	0.29	0.23	0.23	0.22
38	0.06	0.05	0.03	0.04	0.03	0.05	0.06	0.09	0.06	0.06	0.08	0.07	0.14	0.14	0.1	0.11	0.11	0.12	0.17	0.12	0.1	0.17	0.11	0.1
39	0.18	0.16	0.15	0.15	0.14	0.14	0.05	0.09	0.11	0.05	0.07	0.07	0.12	0.16	0.09	0.09	0.11	0.1	0.19	0.19	0.12	0.19	0.12	0.12
40	0.04	0.06	0.08	0.07	0.07	0.08	0.06	0.03	0	0.06	0.04	0.05	0.03	0.03	0.06	0.06	0.07	0	0.1	0.08	0.13	0.07	0.07	0.09
41	0.12	0.1	0.09	0.09	0.08	0.08	0.14	0.15	0.21	0.14	0.17	0.15	0.12	0.12	0.08	0.1	0.09	0.09	0.16	0.19	0.12	0.13	0.13	0.13
42	0.13	0.11	0.09	0.1	0.09	0.09	0.13	0.16	0.13	0.13	0.15	0.14	0.32	0.32	0.26	0.26	0.25	0.26	0.18	0.18	0.18	0.18	0.12	0.11
43	0.17	0.15	0.13	0.14	0.14	0.13	0.05	0.06	0.11	0.05	0.07	0.07	0.11	0.11	0.06	0.09	0.1	0.08	0	0.05	0	0	0.06	0.07
44	0.16	0.14	0.13	0.14	0.14	0.12	0.16	0.17	0.16	0.16	0.18	0.17	0.21	0.21	0.17	0.18	0.18	0.19	0.23	0.18	0.23	0.23	0.17	0.17
45	0.11	0.1	0.09	0.09	0.09	0.08	0.06	0.03	0.06	0.06	0.04	0.05	0.1	0.1	0.06	0.08	0.07	0.08	0.17	0.12	0.17	0.17	0.11	0.13
46	0.13	0.11	0.09	0.1	0.09	0.09	0.14	0.15	0.14	0.14	0.16	0.16	0.13	0.13	0.1	0.11	0.12	0.11	0.1	0.08	0.06	0.13	0.07	0.09
47	0.2	0.18	0.16	0.17	0.17	0.16	0.18	0.25	0.19	0.18	0.21	0.2	0.14	0.17	0.09	0.12	0.1	0.11	0.04	0.05	0.07	0	0.06	0.05
48	0.15	0.13	0.11	0.05	0.11	0.11	0.09	0.1	0.15	0.09	0.11	0.1	0.13	0.13	0.1	0.11	0.12	0.11	0.04	0.05	0.07	0	0.06	0.07
49	0.1	0.08	0.06	0.07	0.07	0.06	0.03	0.06	0.09	0.03	0.05	0.05	0.16	0.16	0.12	0.13	0.13	0.14	0.04	0	0.07	0	0.06	0.05
50	0.16	0.14	0.12	0.13	0.12	0.12	0.14	0.15	0.21	0.14	0.17	0.16	0.16	0.13	0.08	0.1	0.12	0.1	0.07	0.06	0.04	0.05	0.04	0.06

51	0.23	0.22	0.2	0.21	0.21	0.19	0.18	0.19	0.24	0.18	0.2	0.2	0.16	0.16	0.13	0.13	0.12	0.13	0.16	0.16	0.16	0.1	0.1	0.12
52	0.11	0.09	0.07	0.08	0.08	0.1	0.15	0.19	0.21	0.15	0.17	0.17	0.15	0.15	0.11	0.12	0.14	0.13	0	0.05	0.07	0	0.06	0.07
53	0.14	0.13	0.11	0.12	0.12	0.11	0.12	0.16	0.13	0.13	0.15	0.14	0.14	0.14	0.12	0.11	0.11	0.12	0	0	0.07	0.06	0.06	0.07
54	0.1	0.09	0.06	0.07	0.08	0.06	0.08	0.11	0.14	0.08	0.1	0.09	0.11	0.11	0.07	0.09	0.1	0.08	0.06	0.05	0.1	0.1	0.03	0.03
55	0.13	0.11	0.1	0.11	0.1	0.09	0.04	0.08	0.04	0.04	0.06	0.06	0.13	0.13	0.1	0.11	0.12	0.11	0.15	0.19	0.19	0.19	0.12	0.12
56	0.13	0.11	0.1	0.11	0.11	0.12	0.06	0.03	0.06	0.06	0.04	0.04	0.07	0.07	0.03	0.04	0.04	0.05	0.09	0.13	0.05	0.13	0.06	0.06
57	0.11	0.09	0.08	0.08	0.08	0.07	0.07	0.11	0.07	0.07	0.1	0.09	0.21	0.21	0.17	0.18	0.2	0.19	0.09	0.08	0.06	0.13	0.07	0.06
58	0.2	0.18	0.17	0.17	0.17	0.16	0.12	0.16	0.19	0.12	0.15	0.14	0.11	0.08	0.04	0.05	0.07	0.05	0.17	0.16	0.21	0.21	0.14	0.14
59	0.13	0.11	0.1	0.1	0.09	0.12	0.15	0.16	0.22	0.15	0.18	0.17	0.08	0.08	0.02	0.03	0.02	0.02	0	0.05	0.07	0	0.06	0.07
60	0.3	0.29	0.27	0.28	0.27	0.26	0.05	0.06	0.05	0.05	0.07	0.06	0.16	0.12	0.08	0.09	0.09	0.1	0	0	0.07	0	0.06	0.05
61	0.12	0.11	0.09	0.09	0.1	0.11	0.17	0.2	0.17	0.17	0.19	0.19	0.07	0.07	0.03	0.05	0.04	0.05	0	0	0	0.06	0.07	0.07
62	0.1	0.08	0.07	0.07	0.07	0.06	0.09	0.1	0.15	0.09	0.11	0.11	0.19	0.23	0.17	0.16	0.18	0.17	0	0.05	0.07	0	0.06	0.07
63	0.1	0.08	0.06	0.07	0.07	0.09	0.06	0.1	0.06	0.06	0.08	0.08	0.1	0.1	0.07	0.07	0.07	0.08	0.04	0.05	0.07	0	0.06	0.07
64	0.15	0.14	0.12	0.13	0.13	0.12	0.06	0.03	0.06	0.06	0.04	0.04	0.03	0.03	0.06	0.05	0.07	0.06	0.04	0.05	0	0.06	0.06	0.05
65	0.24	0.22	0.2	0.21	0.2	0.2	0.1	0.14	0.1	0.1	0.12	0.12	0.13	0.13	0.1	0.11	0.12	0.11	0.04	0.05	0	0.06	0.06	0.07
66	0.15	0.13	0.12	0.12	0.11	0.11	0.06	0.09	0.06	0.06	0.08	0.07	0.21	0.21	0.18	0.18	0.18	0.19	0	0.05	0.07	0.06	0.07	0.07

TA-R; Right Temporalis muscle, TA-L; Left Temporalis muscle, MM-R; Right Masseter Muscle, MM-L; Left Masseter Muscle, DA-R; Right Digastric Muscle, DA-L; Left Digastric Muscle

Normalised muscle activity duration quotients for muscles during different exercises

Participant	Maximum Mouth Opening						Maximum lateral excursion						Maximum anterior protrusion						Chewing					
	TA-R	TA-L	MM-R	MM-L	DA-R	DA-L	TA-R	TA-L	MM-R	MM-L	DA-R	DA-L	TA-R	TA-L	MM-R	MM-L	DA-R	DA-L	TA-R	TA-L	MM-R	MM-L	DA-R	DA-L
1	9.47	25.17	73.17	94	87.92	137.67	15.67	134.33	54.67	52.33	147.67	132	70.33	173.33	135.67	96.33	26.67	208.17	398	398	999	398	988	968
2	74.83	78.83	87.67	10	92.58	9	142.17	40.33	182.33	41.67	53.67	38	80.33	183.33	125.67	80.92	16.67	198.17	960	58	437	408	436	406
3	16.7	3.5	47	67.83	14.75	68.83	440.33	559	479.33	554	572.33	556.67	192	20.5	43.75	77.67	6.75	189.5	710	37	532	865	531	16
4	30.87	17.67	32.83	53.67	28.92	54.67	153.83	11.83	51	53.33	42	26.33	141.33	71.17	167.67	122.92	58.67	240.17	0	0	0	0	1386	849
5	24.87	11.67	157.33	59.67	53.58	103.33	33	45.17	227.67	3.67	7.67	83.33	201	201	34.75	68.67	1	180.5	1004	1159	238	1159	237	207
6	89.53	76.33	25.83	5	11.08	4	324.5	182.5	0	224	220	993	115.33	5.83	90.67	45.92	18.33	163.17	116	116	1281	1252	1280	1250
7	23.53	7.83	40.17	61	54.92	62	107.17	11.5	68.17	6.5	66.5	839.5	96.33	13.17	36.42	64.92	0.67	182.17	1189	1189	208	1189	207	177
8	60.95	45.25	2.75	23.58	17.5	24.58	89.67	29	50.67	24	49	26.67	287.67	287.67	51.92	23.42	87.67	93.83	324	423	479	479	907	370
9	105	111.5	61	40.17	46.25	39.17	198.83	317.5	237.83	235.5	330.83	315.17	190	190	119	74.25	10	191.5	155	902	0	1368	1396	1366
10	58.53	45.33	5.17	26	56.58	69.67	127.17	8.5	88.17	13.5	4.83	10.83	43.2	146.2	162.8	118.05	53.8	235.3	267	480	975	422	974	944
11	71.2	58	111	13.33	7.25	14.33	88.17	270.67	412.67	265.67	284	268.33	258	45.5	22.25	6.25	58	123.5	1027	1027	1027	341	369	339
12	12.2	1	51.5	72.33	10.25	73.33	137.33	256	176.33	251	178	595	90	19.5	116	71.25	270	188.5	1119	217	278	249	277	270
13	30.53	14.83	33.17	54	28.58	55	287.83	366	326.83	401.5	328.5	444.5	234.67	234.67	74.33	29.58	34.67	146.83	155	902	0	0	1396	849
14	58.87	43.17	4.83	25.67	56.92	26.67	93	171.17	132	129.67	225	639.33	288.5	288.5	20.5	24.25	88.5	93	155	902	1397	1368	1396	849

15	16.2	0.5	47.5	68.33	62.25	69.33	25	93.67	235.67	88.67	15.67	91.33	177	35.5	58.75	87.25	23	204.5	0	902	1397	0	1396	849
16	174.2	161	110.5	89.67	172.25	88.67	92.67	26	53.67	21	52	825	103	212.5	309	264.25	463	381.5	0	0	0	1368	1396	1366
17	52.53	36.83	11.17	32	50.58	33	173.5	315.5	276.33	351	278	353.67	189	79.5	56.25	27.75	171	89.5	1016	269	226	197	225	195
18	72.2	59	110	12.33	6.25	13.33	45.5	137	57.33	55	59	134.67	228	15.5	81	36.25	235	153.5	407.5	339.5	562.5	805.5	833.5	803.5
19	66.2	50.5	116	18.33	12.25	19.33	150.83	269.5	189.83	264.5	191.5	581.5	279	66.5	43.25	9.33	184	102.5	381	366	536	832	860	313
20	90.2	77	26.5	5.67	88.25	4.67	115.67	37.5	76.67	2	75	848	758	648.5	625.25	596.75	661	479.5	1059	1214	183	1214	182	365
21	132.83	139.33	88.83	68	74.08	67	317.33	436	578	354	358	433.67	242.33	132.83	36.33	75.67	145.33	36.17	297.5	449.5	944.5	915.5	943.5	396.5
22	125.2	109.5	61.5	40.67	123.25	3	96.33	174.5	357	210	228.33	212.67	231	121.5	98.25	64.33	134	47.5	0	902	0	1368	1396	849
23	39.53	23.83	24.17	45	37.58	46	74.67	44	186	38	34	41.67	251.33	38.83	15.58	18.33	51.33	130.17	26	773	1268	129	1267	1237
24	2.05	17.75	184.25	86.58	4	130.25	100.67	178.83	139.67	214.33	141.33	631.67	201	91.5	68.25	39.75	159	77.5	1088	1243	154	125	153	123
25	18.53	2.83	45.17	66	16.58	67	28.67	90	232	85	12	761	112.67	3.17	20.08	54	15.67	165.83	155	0	0	1368	1386	1366
26	67.2	54	115	17.33	11.25	18.33	125.33	244	386	162	166	241.67	113.33	3.83	92.67	47.92	16.33	165.17	1358	1358	39	10	38	8
27	128.53	115.33	53.67	44	126.58	43	29.92	88.75	230.75	6.75	10.75	762.25	142.67	33.17	9.92	24	217.33	135.83	155	902	1397	0	1396	1366
28	58.7	45.5	123.5	25.83	56.75	26.83	85.33	33.33	46.33	28.33	46.67	817.67	234.67	125.17	28.67	68	137.67	43.83	0	902	1397	0	1396	849
29	34.2	21	29.5	50.33	44.25	51.33	108.67	23.67	118.33	28.67	101.67	874.67	103	212.5	235.75	264.25	463	381.5	0	0	0	1368	1386	1366
30	2.2	13.5	61.5	82.33	76.25	83.33	129.17	51	131.5	92.5	2.83	861.5	105.33	4.17	27.42	61.33	8.33	173.17	554	348	554	554	842	295
31	49.2	36	14.5	35.33	29.25	36.33	116.67	38.5	144	80	15.33	0.33	168	271	38	6.75	71	110.5	0	0	0	1368	1396	849
32	34.53	18.83	29.17	50	32.58	51	47.17	31	8.17	10.5	6.5	779.5	280	280	29	15.75	183	101.5	0	902	1397	0	1396	1366

33	183.87	170.67	120.17	99.33	181.92	98.33	90.83	169	351.5	127.5	131.5	641.5	367	154.5	58	102.75	96	14.5	155	902	1397	1368	1396	1366
34	40.7	25	141.5	43.83	38.75	87.5	109	187.17	148	222.67	149.67	225.33	204.33	94.83	71.58	43.08	155.67	74.17	1067	1222	1222	1222	174	373
35	24.25	28.25	19.75	40.58	34.5	41.58	23.67	142.33	62.67	60.33	64.33	140	279.5	170	146.75	118.25	80.5	1	155	0	1397	1368	1396	1366
36	34.95	19.25	147.25	49.58	43.5	50.58	111.67	149	72.67	2	71	4.67	287.5	287.5	21.5	17.83	87.5	94	0	0	0	1368	1396	1366
37	143.2	127.5	79.5	58.67	64.75	57.67	47.42	71.25	213.25	66.25	6.75	68.92	94.75	197.75	38	71.92	2.25	183.75	1392	1392	1392	24	4	26
38	0.13	15.83	63.83	84.67	78.58	128.33	119.87	1.2	80.87	6.2	79.2	852.2	155.33	45.83	50.67	5.92	204.67	123.17	465	437	932	465	931	901
39	41.45	28.25	140.75	43.08	37	44.08	63.33	182	324	177	195.33	669	55.5	158.5	77.25	105.75	41.5	223	620	620	777	620	766	746
40	161.8	177.5	225.5	246.33	163.75	247.33	260.67	142	0	147	128.67	144.33	103	212.5	235.75	264.25	463	0	406	341	561	807	835	288
41	25.7	12.5	156.5	58.83	52.75	59.83	26.33	104.5	287	63	67	142.67	167.33	57.83	38.67	0.67	192.67	111.17	36.33	191.33	1205.67	1176.67	1194.67	1174.67
42	52.2	39	11.5	32.33	26.25	33.33	10.33	129	49.33	124	51	126.67	195	195	40.75	69.25	268	186.5	1348	1348	1348	1348	48	18
43	66.53	53.33	2.83	18	64.58	19	65	13.17	195.67	48.67	24.33	797.33	92.33	17.17	113.67	74.33	4.67	186.17	0	902	0	0	1396	1366
44	21.95	8.75	160.25	62.58	20	63.58	39.67	38.5	0.67	3	1	76.67	87.67	21.83	118.33	73.58	272.33	190.83	179	723	179	179	1217	1187
45	38.5	45	124	26.33	56.25	27.33	260.67	142	221.67	147	220	144.33	256.33	146.83	50.33	89.67	103.67	22.17	1274	372	1274	1274	122	425
46	41.95	28.75	21.75	42.58	36.5	43.58	116.33	38.17	77.33	2.67	75.67	848.67	111.67	2.17	21.08	55	14.67	166.83	1266	519	24	1421	25	572
47	116.2	103	52.5	31.67	114.25	30.67	85.17	175.5	46.17	28.5	44.5	31.17	110	213	96	56.67	250	168.5	155	902	1397	0	1396	849
48	54.87	39.17	8.83	2	23.58	30.67	108.33	186.5	369	222	149	224.67	186	76.5	53.25	19.33	89	92.5	155	902	1397	0	1396	1366
49	22.95	9.75	40.75	61.58	21	62.58	128.33	9.67	132.33	14.67	87.67	860.67	83.67	25.83	122.33	77.58	276.33	194.83	155	0	1397	0	1396	849
50	109.87	96.67	46.17	25.33	31.42	24.33	21.92	56.25	238.75	14.75	18.75	94.42	262	49.5	47	2.25	62	119.5	43.33	703.67	1198.67	1169.67	1197.67	650.67

51	11.2	4.5	52.5	73.33	9.25	74.33	72.67	5.5	188	36	59.33	805	110.75	1.25	22	50.5	249.25	167.75	1421	1421	1421	53	25	572
52	11.45	1.75	52.25	73.08	9.5	116.75	62.67	56	198	51	22	795	1285	1175.5	1079	1123.75	1188	1006.5	0	902	1397	0	1396	1366
53	146.87	133.67	83.17	62.33	144.92	61.33	32.5	150	70.33	145	72	147.67	131.33	21.83	1.42	29.92	228.67	147.17	0	0	1397	1368	1396	1366
54	75.2	59.5	11.5	9.33	73.25	10.33	102.33	221	363	216	143	630	226.33	116.83	20.33	59.67	129.33	52.17	239.5	507.5	394.5	394.5	1001.5	971.5
55	28.2	15	154	56.33	50.25	57.33	111.33	7.33	72.33	2.33	70.67	843.67	178	68.5	45.25	11.33	81	100.5	1106	1261	1261	1261	125	105
56	35.7	22.5	28	48.83	33.75	92.5	260.67	142	221.67	224	220	993	134	24.5	72	27.25	226	144.5	461	616	781	616	780	750
57	62.53	46.83	119.67	22	60.58	23	160.33	279	199.33	197	201	572	60.5	49	145.5	100.75	36.5	218	453	294	789	608	788	758
58	38.95	23.25	143.25	45.58	37	46.58	89.33	29.33	171.33	52.67	48.67	821.67	184	28.5	125	80.25	16	197.5	302	445	457	457	939	909
59	62.87	47.17	119.33	21.67	15.58	65.33	16.33	94.5	277	130	57	716	152	152	83.75	117.67	311	229.5	0	902	1397	0	1396	1366
60	29.45	13.75	34.25	55.08	49	56.08	47.67	30.5	8.67	66	7	68.67	365	152.5	56	100.75	98	16.5	0	0	1397	0	1396	849
61	85.2	69.5	97	0.67	83.25	43	60.33	179	99.33	174	101	672	182.67	73.17	23.33	16	177.33	95.83	0	0	0	1368	1386	1366
62	23.45	10.25	40.25	61.08	55	62.08	80	158.17	340.67	193.67	120.67	652.33	75.5	178.5	57.25	85.75	21.5	203	0	902	1397	0	1396	1366
63	1.8	15	65.5	86.33	3.75	130	37.67	156.33	76.67	151.33	169.67	694.67	219.33	109.83	86.58	58.08	140.67	59.17	155	902	1397	0	1396	1366
64	1.87	11.33	61.83	82.67	0.08	83.67	260.67	142	221.67	147	220	993	103	212.5	235.75	269.67	463	381.5	155	902	0	1368	1396	849
65	5.4	7.8	58.3	79.13	73.05	80.13	135.17	16.5	96.17	21.5	94.5	867.5	227	117.5	94.25	60.33	130	51.5	155	902	0	1368	1396	1366
66	60.95	45.25	121.25	23.58	17.5	24.58	102.92	15.75	63.92	10.75	29.08	13.42	100.5	9	32.25	60.75	259.5	178	0	902	1397	1368	1386	1366

TA-R; Right Temporalis muscle, TA-L; Left Temporalis muscle, MM-R; Right Masseter Muscle, MM-L; Left Masseter Muscle, DA-R; Right Digastric Muscle, DA-L; Left Digastric Muscle
